# New platinum derivatives selectively cause double-strand DNA breaks and death in naïve and cisplatin-resistant cholangiocarcinomas

**DOI:** 10.1016/j.jhep.2025.04.034

**Published:** 2025-11

**Authors:** Irene Olaizola, Mikel Odriozola-Gimeno, Paula Olaizola, Francisco J. Caballero-Camino, Noelia Pastor-Toyos, Mireia Tena-Garitaonaindia, Ainhoa Lapitz, Beatriz Val, Amanda R. Guimaraes, Maitane Asensio, Maider Huici-Izagirre, Colin Rae, David de Sancho, Xabier Lopez, Pedro M. Rodrigues, Elisa Herraez, Oscar Briz, Laura Izquierdo-Sanchez, Aitziber Eleta-Lopez, Alexander M. Bittner, Ana Martinez-Amesti, Teresa Miranda, Sumera I. Ilyas, Chiara Braconi, Maria J. Perugorria, Luis Bujanda, Iván Rivilla, Jose J.G. Marin, Fernando P. Cossío, Jesus M. Banales

**Affiliations:** 1 Department of Liver and Gastrointestinal Diseases, Biogipuzkoa Health Research Institute - Donostia University Hospital -, University of the Basque Country (UPV/EHU), Donostia-San Sebastian, Spain; 2 Department of Organic Chemistry I, Center of Innovation in Advanced Chemistry (ORFEO-CINQA), Faculty of Chemistry, University of the Basque Country (UPV/EHU) & Donostia International Physics Center (DIPC), Donostia-San Sebastian, Spain; 3 National Institute for the Study of Liver and Gastrointestinal Diseases (CIBERehd, "Instituto de Salud Carlos III"), Spain; 4 Department of Biochemistry and Molecular Biology, Faculty of Science and Technology, University of the Basque Country (UPV/EHU), Leioa, Spain; 5 Experimental Hepatology and Drug Targeting (HEVEPHARM), Institute of Biomedical Research of Salamanca (IBSAL), University of Salamanca, Salamanca, Spain; 6 School of Cancer Sciences, University of Glasgow, Glasgow, UK; 7 Polimero eta Material Aurreratuak: Fisika, Kimika eta Teknologia & Donostia International Physics Center (DIPC), Donostia-San Sebastian, Spain; 8 IKERBASQUE, Basque Foundation for Science, Bilbao, Spain; 9 Didactics of Mathematics, Experimental and Social Science, Faculty of Education, Philosophy and Anthropology, University of the Basque Country (UPV/EHU), Donostia-San Sebastian, Spain; 10 CIC nanoGUNE (BRTA), Donostia-San Sebastian, Spain; 11 SGIker, Advanced Research Facilities, University of the Basque Country (UPV/EHU), Donostia-San Sebastian, Spain; 12 Division of Gastroenterology and Hepatology, Mayo Clinic College of Medicine and Science, Rochester, MN, USA; 13 Beatson West of Scotland Cancer Centre, Glasgow, UK; 14 Department of Medicine, Faculty of Medicine and Nursing, University of the Basque Country (UPV/EHU), Donostia-San Sebastian, Spain; 15 Department of Biochemistry and Genetics, School of Sciences, University of Navarra, Pamplona, Spain

**Keywords:** Cancer, Chemotherapy, Chemoresistance, DNA damage

## Abstract

**Background & Aims:**

Patients with cholangiocarcinoma (CCA) have poor prognosis. Current cisplatin-based first-line chemotherapy offers limited survival benefit. Cisplatin induces single-strand DNA breaks, activating DNA repair mechanisms that diminish its effectiveness. Here, we present the design, chemical synthesis, and therapeutic evaluation of a new generation of chemotherapeutic agents (Aurkines) with unique polyelectrophilic properties. These agents cause a high frequency of double-strand DNA breaks, bypassing DNA repair, and promoting cancer cell death.

**Methods:**

Two novel compounds, Aurkine 16 and Aurkine 18, were designed and evaluated for their antitumor effects in both naïve and cisplatin-resistant CCA cells, cancer-associated fibroblasts, healthy cholangiocytes, and *in vivo* models.

**Results:**

Aurkines effectively induced double-strand DNA breaks, leading to increased DNA damage and elevated levels of reactive oxygen species, resulting in greater cytotoxicity than cisplatin in CCA cells. Phosphoproteomic and molecular analysis revealed that cisplatin activates DNA repair pathways, while Aurkines primarily induce apoptosis. Importantly, Aurkines also triggered apoptosis in cisplatin-resistant CCA cells and cancer-associated fibroblasts without harming healthy cholangiocytes. Additionally, Aurkines demonstrated cytotoxicity in other cisplatin-resistant cancers, such as breast and ovarian cancer. This tumor selectivity results from reduced uptake, increased efflux, and compact chromatin structure in normal cells, limiting Aurkine-DNA interactions. *In vivo*, Aurkines inhibited the growth of subcutaneous naïve and cisplatin-resistant CCA tumors, as well as orthotopic tumors in immunocompetent mice, promoting antitumor immune cell recruitment without any adverse events. Transport studies revealed that Aurkines were selectively taken up by OCT1, OCT3, CTR1, and OATP1A2, whereas only CTR1 transported cisplatin.

**Conclusions:**

Aurkines represent promising therapeutic drugs for both naïve and cisplatin-resistant cancers due to their unique polyelectrophilic properties and selective targeting of malignant cells.

**Impact and implications:**

This study introduces a novel therapeutic strategy designed to induce frequent double-strand DNA breaks selectively in both naïve and cisplatin-resistant cancer cells, without evident toxic side effects at therapeutic doses. This approach may form the basis for new strategies to overcome the critical challenge of drug resistance in cancer treatment and has the potential to be a breakthrough not only for the treatment of biliary tumors but also for other cancers.

## Highlights

• Aurkines represent a new generation of chemotherapeutic agents with distinctive polielectrophilic properties.
• Aurkines selectively target DNA in cancer cells, including cholangiocarcinoma.
• Aurkines demonstrate greater efficacy than cisplatin in experimental models of both naïve and cisplatin-resistant CCA.
• Aurkines show no evident systemic toxicity at therapeutic doses.
• Aurkines impact the tumor microenvironment, targeting cancer-associated fibroblasts and boosting immunogenicity.

## Graphical abstract

**Figure:**
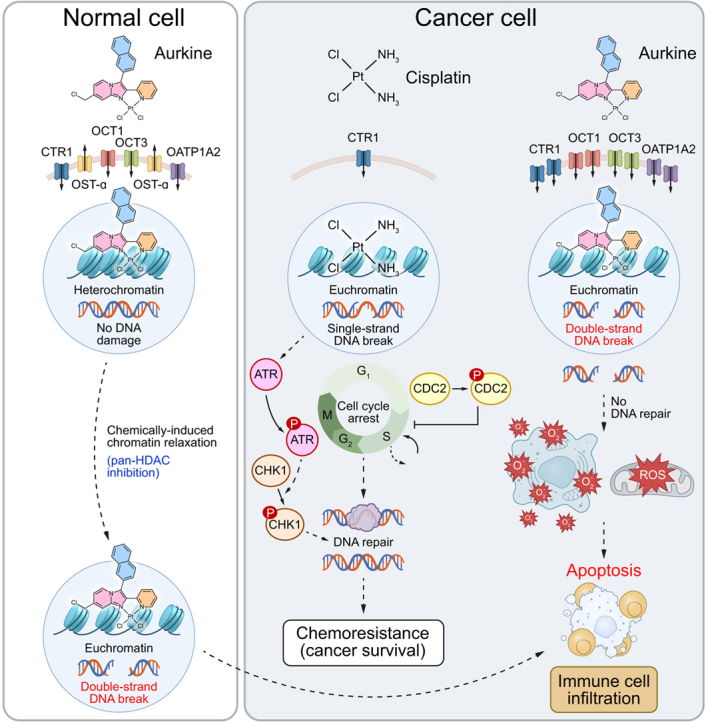

## Introduction

Cholangiocarcinoma (CCA) comprises a heterogeneous group of malignant tumors arising along the biliary tree.^1^ Ranked as the second most frequent primary liver cancer, CCA contributes to 3% of all gastrointestinal malignancies and accounts for 2% of all cancer-related deaths.^2^ However, these statistics are likely underestimated due to errors in disease coding, diagnosis, and data retrieval. Globally, the incidence (0.3-6 cases per 100,000 inhabitants annually) and mortality (1-6 per 100,000 inhabitants annually) of CCA are on the rise, representing a significant health challenge.^1^ Patients with early stage CCA typically lack symptoms, leading to the majority of diagnoses at advanced stages (70%), often when the cancer has already spread.^2^ This delayed diagnosis, coupled with the tumor chemoresistance, contributes to unfavorable outcomes. Current curative strategies for CCA include surgical resection or liver transplantation, although less than 30% of patients are eligible for these interventions and the risk of tumor recurrence is still high.^1^^,^^2^ In unresectable cases, palliative treatment based on systemic therapy remains the only feasible option. Until recently, the first-line treatment for advanced CCA involved a combination of gemcitabine + cisplatin (GemCis),^3^ which evolved into a triple combination with the inclusion of the monoclonal antibodies durvalumab (anti-PD-L1)^4^ or pembrolizumab (anti-PD1).^5^ Nevertheless, the median overall survival achieved with this combined therapy remains modest, with a one-year estimate, and this regimen is still considered palliative.^4^^,^^5^

Cisplatin (CisPt) is a fundamental therapeutic agent in the fight against various cancer types.^6^ Whether used as monotherapy or in combination with other therapies, CisPt currently stands as the primary treatment for different types of solid cancers, including CCA, breast, ovarian, lung, and testicular cancers, among others. However, the use of CisPt is associated with potential side effects, such as myelotoxicity, nephrotoxicity, neuropathy, ototoxicity, and the tendency to induce nausea and vomiting.^7^ Resistance to CisPt still remains a challenge in cancer treatment, with certain tumors exhibiting intrinsic resistance while others gradually acquire resistance over time, despite an initial positive response.^8^

CisPt, cis-[Pt(NH_3_)_2_Cl_2_)], is a platinum (Pt) compound that binds to DNA, inducing mainly single-strand DNA breaks and subsequently causing cancer cell death.^9^^,^^10^ This process includes various steps: (i) cellular uptake through diffusion and facilitated transport; (ii) intracellular activation through interaction with water molecules in the presence of low concentrations (10-20 mM) of chloride ions; this leads to the generation of aqua complexes cis-[Pt(NH_3_)_2_Cl(OH_2_)]^+^ and cis-[Pt(NH_3_)_2_(OH_2_)_2_]^2+^, electrophilic substances that interact with DNA; (iii) nuclear uptake and interaction with nucleophilic centers N7 of the DNA nitrogen bases guanine (G) and, to a lesser extent, adenine (A), forming covalent bonds, preferably with two guanosine residues.^11^ In this context, *in vitro* assays have revealed that CisPt generates 47-50% of adducts on a single strand (*i.e.* intrastrand), comprising two contiguous G units of the cis-[Pt(NH_3_)_2_{d(GpG)}] (or cis-GG) type. Additionally, 23-28% of intrastrand adducts integrate two consecutive A and G units of the cis-[Pt(NH_3_)_2_{d(ApG)}] (or cis-AG) type, while 8-10% of intrastrand adducts are composed of two Pt-G linkages separated by another base X in the cis-[Pt(NH_3_)_2_{d(GpXpG)}] (or cis-GXG) structure. Finally, 2-3% of CisPt-DNA adducts are single-base G linkages.^12^ A parallel *in vitro* exploration involving a CisPt analogue yielded similar outcomes.^13^ *Ex vivo* analyses with human cancer samples mirrored these findings, with cis-GG, cis-AG, and cis-GXG proportions being 65%, 22%, and 13%, respectively.^14^ These data highlight that interstrand crosslinks, leading to double-strand DNA breaks, which are more challenging for cancer cells to repair,^15^ represent a relatively rare event, accounting for less than 5% of crosslinks.^13^^,^^14^ This allows cancer cells to activate their DNA repair mechanisms against single-strand DNA breaks, thereby enabling them to survive and develop resistance to CisPt.^16^

**Fig. 1:**
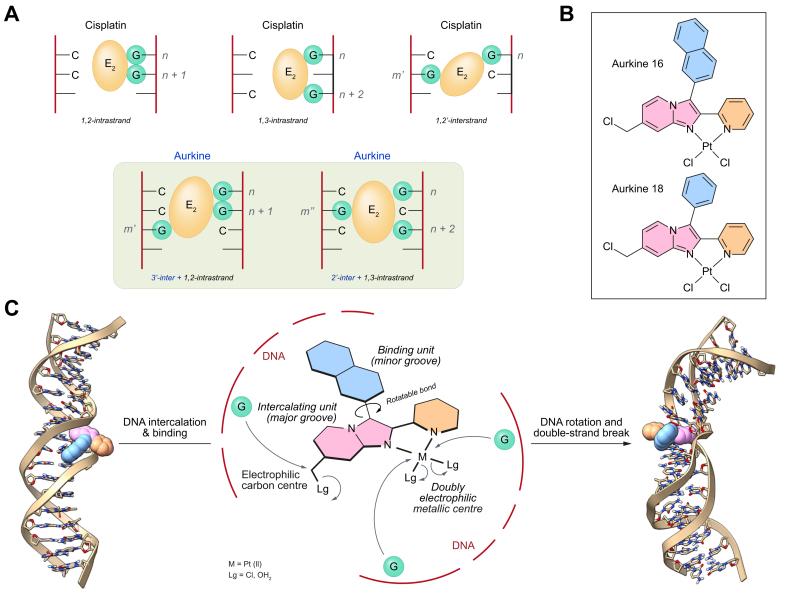
Design, structure, and interaction modes of Aurkines. (A) Interaction of dielectrophilic (E_2_) and trielectrophilic (E_3_) platinum reagents with guanine (G) units. (B) Chemical structures of Aurkines 16 and 18. (C) Molecular dynamics simulations illustrating the interaction of Aurkine 16 with DNA, highlighting both the initial minor groove binding associated with the β-naphthyl moiety (pale blue) and the subsequent major groove intercalation of the heterocyclic groups (pink and orange).

Considering the activity of Pt(II) derivatives and the resistance mechanisms developed by cancer cells, we hypothesize that novel chemotherapeutic agents with marked polyelectrophilic properties could induce interstrand DNA crosslinks. This mechanism would inhibit DNA repair mechanisms, thereby promoting cancer cell death. Considering the interstrand interactions between DNA and dielectrophilic chemotherapeutic agents like CisPt (Fig. 1A, 1^st^ row), we reasoned that adding an extra electrophilic site would create a trielectrophilic agent capable of generating higher rates of double-strand DNA breaks (Fig. 1A, 2^nd^ row, highlighted in green). Thus, we formulated and synthesized a new family of chemotherapeutic agents with pronounced polyelectrophilic characteristics, named Aurkines. Among the synthesized compounds, Aurkine 16 and Aurkine 18 (Fig.1B), emerged as the most promising, exhibiting IC_50_ values for cell viability lower than those of CisPt and showing remarkable selectivity for cancer cells (Fig. S1). These compounds were designed to interact with DNA at multiple sites and were tested therapeutically for CCA. This design included a Pt(II) electrophilic center capable of substituting two chloride groups with two G (or A) units, along with an additional 4-chlorobenzyl carbon-based electrophilic center. Additionally, aryl groups (highlighted in pale blue in Fig. 1B,C) were incorporated to bind and intercalate with the minor and major grooves of DNA, as illustrated in Fig. 1C.

## Materials and methods

### Chemical synthesis of Aurkines 16 and 18

Aurkines 16 and 18 were synthesized as described in the supplementary information - chemical synthesis file.

### Cell cultures

Normal human cholangiocytes (NHCs) were isolated from healthy liver tissue. Three human CCA cell lines were used: HUCCT1 (intrahepatic CCA), EGI-1 (extrahepatic CCA sensitive to CisPt) and EGI-1R (extrahepatic CCA resistant to CisPt). EGI-1R was generated as detailed in the supplementary information. Cancer-associated fibroblasts (CAFs) were isolated from resected intrahepatic CCA. Details of cell isolation, culture, and characterization are provided in the supplementary information.

### Patient-derived organoids (PDOs) from CCA tumors

PDOs from patients with CCA were generated as described in the supplementary information.

### Transcriptomic analysis of human samples

Transcriptomic analyses were performed on CCA tumors and adjacent non-tumor liver tissues using datasets from five distinct publicly available patient cohorts: AHN (dataset: GSE107943), The Thailand Initiative in Genomics and Expression Research (TIGER-LC; dataset: GSE76297), Copenhagen (dataset: GSE26566), Cancer Genome Atlas (TCGA-CHOL) and JOB (dataset: E-MATB-6389). To assess the association between gene expression and tumor mutational profiles, data from the publicly available DONG cohort (OEP001105) was used.

### scRNA-seq analysis of human CCA tissues

Publicly available single-cell RNA sequencing (scRNA-seq) data from human CCA tumors (GSE151530) were analyzed as described in the supplementary information.

### RNA isolation and gene expression

Total RNA was extracted from cultured cells using TRI Reagent® and analyzed by reverse transcription and quantitative real-time PCR, as outlined in the supplementary information. Primer sequences are listed in Table S1.

### Histological analyses

H&E staining was performed to assess tissue morphology, as previously described.^17^ Immunohistochemistry (IHC) was used to evaluate markers of proliferation (Ki67), apoptosis (cleaved caspase-3), DNA damage (p-H2AX) and immune cell populations (CD4 and CD8), as previously detailed.^17^ The antibodies used are listed in Table S2.

### Immunoblotting

Protein expression in CCA cells and NHCs was analyzed using immunoblotting, with antibodies listed in Table S2. Methodological details are provided in the supplementary information.

### ROS detection, cell viability, proliferation, cell cycle, and apoptosis assays

Assays for reactive oxygen species (ROS) detection, cell viability, proliferation, cell cycle, and apoptosis were conducted on NHCs, CCA cells, and CAFs in the presence or absence of CisPt or Aurkines (10 μM or 20 μM), following protocols described in the supplementary information.

### 3D spheroids

3D spheroids were generated and monitored as described in the supplementary information.

### Microscopy and molecular analyses

Atomic force microscopy (AFM), transmission electron microscopy (TEM), pUC18 plasmid mobility, and comet assays were conducted to evaluate DNA interactions and damage, as detailed in the supplementary information.

### Experimental overexpression of human transporters in cells and transport assays

Overexpression of human transporters, along with direct and indirect transport assays, were performed using flow cytometry and HPLC-MS/MS, respectively.

### Mass spectrometry-based high-throughput phosphoproteomic analysis

Mass spectrometry-based high-throughput phosphoproteomic analysis was performed in the EGI-1 CCA cell line following treatment with CisPt, Aurkine 16, or Aurkine 18, as described in the supplementary information.

### Experimental animal models of CCA

Subcutaneous CCA xenografts in immunodeficient mice and orthotopic CCA models in immunocompetent mice were generated as described in the supplementary information.

### Statistical analyses

Statistical analyses are outlined in the supplementary information.

## Results

### Chemical synthesis of Aurkines 16 and 18

These syntheses were planned based on our previous experience on 3-arylimidazo-[1,2-*a*]pyridines.^18^ Basically, a double addition of 2-bromo-1-(pyridine-2-yl)ethan-1-one **(2)** with methyl 2-aminoisonicotinate **(3)** yielded adduct **(4)** in good yield. From this intermediate, both β-naphthyl and phenyl groups were added *via* palladium-catalysed C–C coupling with the corresponding aryl bromides. Then, the electrophilic carbon centers were generated by reduction of the ester group of intermediates **(5)** and **(6)**, followed by chloro-substitution of the alcohol groups. Finally, Aurkines 16 and 18 were obtained in good yields by means of the reaction of chlorides **(7)** and **(8)** with platinum dichloride complexed to two equivalents of dimethyl sulfoxide (Fig. S2).

### Assessment of the triple electrophilicity of Aurkine 16

Molecular dynamics simulations (see supplementary information for further details) of Aurkine 16 on the model sequence 5’-GCACGAACGGACGAACGC-3’ show that aryl groups highlighted in pale blue in Fig. 1C (2-naphthyl in the case of Aurkine 16) promote an initial interaction at the minor groove of DNA. The 2-pyridyl and imidazo-[1,2-*a*]pyridine groups, represented in orange and pink, respectively, induce an additional intercalation step that promotes a severe distortion of the double helix with base-pair eversion. These interactions may constitute the previous stage for the respective substitution reactions.

Computational analysis using density-functional theory on the steps involved in the nucleophilic substitution of the three chlorine atoms of Aurkine 16 by three G residues in the presence of at least two molecules of water show a complex sequential mechanism whose main features are shown in Fig. S3. Previous work from our group on CisPt^19^ and electrophilic carbon atoms of nitrogen mustards^20^ showed that this approach captures the essentials of this process in terms of selectivity and kinetics. Our calculations provide a complex stepwise mechanism that starts with a nucleophilic attack of one molecule of surrounding water to generate a mono-aqua complex **(9)**. This aqua ligand acts as a good leaving group in the subsequent nucleophilic attack by one equivalent of G to yield a mono-G substituted intermediate **(10)**. This latter intermediate is transformed into an activated dication **(11)** *via* nucleophilic substitution by another water molecule. The nucleophilic attack by a second G base yields intermediate **(12)**, whose carbon-centered substitution reaction by the third equivalent of G gives rise to the trisubstituted dicationic species **(13)**, with an overall exergonic reaction profile. The geometries of the critical transition structures **TS1-3** (Fig. S3) are coherent with the expected mechanism, the corresponding activation free energies lying in the range of 10-23 kcal mol^-1^. These results demonstrate that the design criteria of Aurkine 16 and 18 are kinetically and thermodynamically compatible with the mechanisms associated with these transformations.

### Characterization of the binding of Aurkines 16 and 18 with DNA

**Fig. 2:**
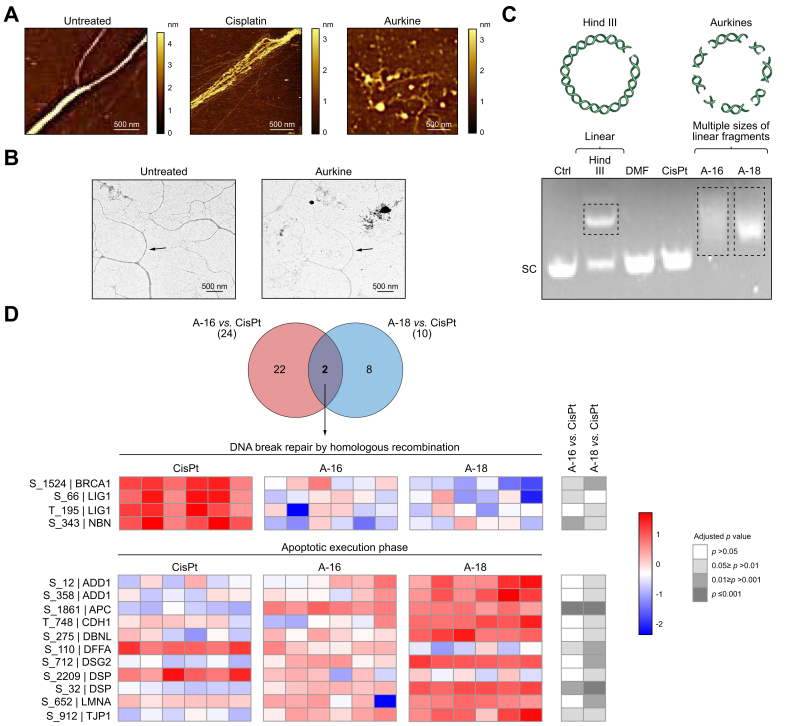
Effect of Aurkine on isolated DNA. (A) Atomic force microscopy (AFM) images of untreated DNA and DNA incubated with CisPt or Aurkine 16 for 10 min. (B) Transmission electron microscopy (TEM) images of untreated DNA, and DNA incubated with Aurkine 16 for 10 min. (C) Agarose gel analysis of pUC18 plasmid after 1 h incubation with vehicle, CisPt, Aurkine 16 or 18. HindIII digestion was used as control. (D) Venn diagram showing the number of canonical pathways with significant adjusted *p* values in both comparisons and a heatmap illustrating the altered protein phosphorylation in EGI-1 cells after a 3-hour exposure to CisPt or Aurkines. Enriched proteins are coloured in red and proteins with lower abundance are displayed in blue. CisPt, cisplatin; DMF, dimethylformamide; SC, supercoiled.

To evaluate the interaction between Aurkine 16 and isolated DNA from *Escherichia coli*, AFM and TEM were employed. AFM images showed that, after 10 min of incubation, CisPt induced some DNA bending, although the main structure of the DNA strand was preserved. In contrast, Aurkine 16 exerted a considerably different effect on DNA, disrupting its strand structure, as shown in Fig. 2A. TEM images further supported these observations, showing a progressive decrease in DNA density over time, consistent with DNA breakage (Fig. 2B). Electrophoretic mobility analysis of the pUC18 plasmid revealed that both Aurkine 16 and 18 induced linear fragments of multiple sizes, which appeared as a diffuse pattern. In contrast, CisPt left the plasmid's supercoiled form unaltered (Fig. 2C). At the molecular level, phosphoproteomic analysis of CCA cells exposed to Aurkines or CisPt for 3 h revealed that CisPt incubation activated DNA repair pathways, while Aurkine primarily promoted apoptotic pathways (Fig. 2D). Consistent with these observations, exposure of CCA cells to CisPt triggered the phosphorylation and activation of key proteins involved in single-strand DNA repair pathways, such as ATR and CHK1 (Fig. S4). This is consistent with the fact that the DNA damage induced by Aurkines exceeds a critical threshold, leading to direct cell death without activating DNA repair mechanisms. These results are in line with the computational data and underscore that Aurkines 16 and 18 operate through a distinct mechanism compared to CisPt.

### Evaluation of the antitumor effect of Aurkines 16 and 18 on human CCA cells

**Fig. 3:**
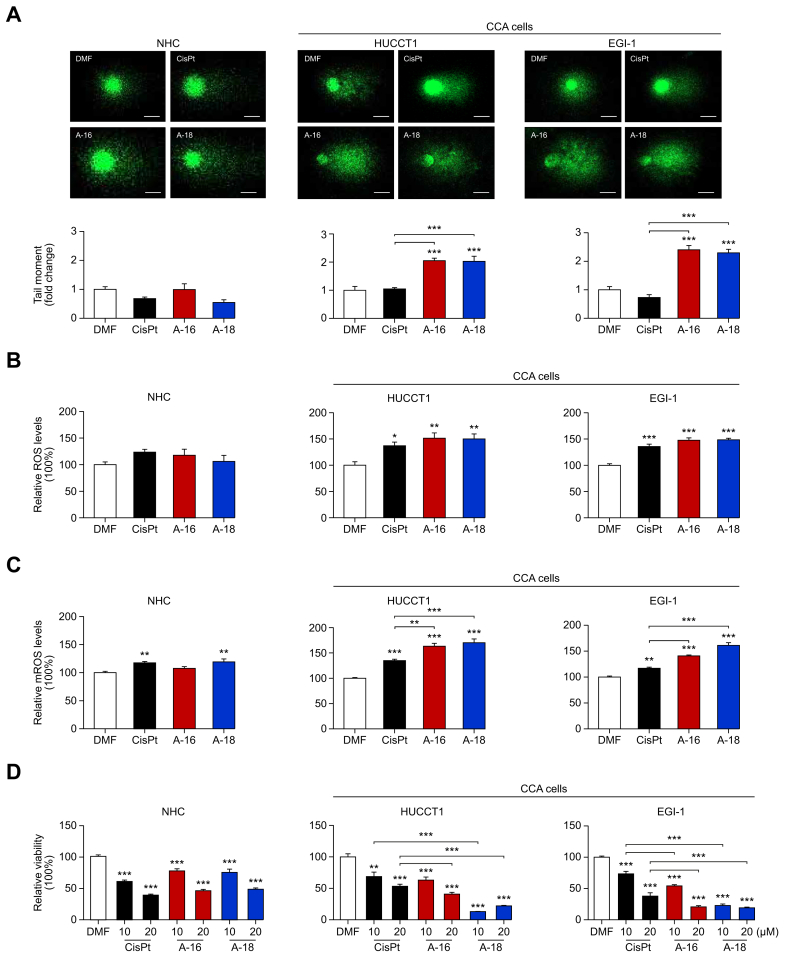
Genotoxic effects of Aurkines 16 and 18 on human CCA cells. (A) DNA damage, (B) ROS and (C) mROS in NHCs and CCA cell lines (HUCCT1 and EGI-1) after 24-hour incubation with vehicle, CisPt, Aurkines 16 or 18 (10 μM). (D) Cell viability of NHCs and CCA cell lines (HUCCT1 and EGI-1) after 48-hour incubation with vehicle, CisPt, Aurkines 16 or 18 (10 μM and 20 μM). One-way ANOVA test or Student’s *t* tests were used. Data are shown as mean ± SEM. ∗*p* ≤0.05, ∗∗*p* ≤0.01, ∗∗∗*p* ≤0.001). CCA, cholangiocarcinoma; CisPt, cisplatin; DMF, dimethylformamide; NHCs, normal human cholangiocytes; mROS, mitochondrial ROS; ROS, reactive oxygen species.

Having confirmed the disruptive impact of Aurkines 16 and 18 on DNA structure, including the generation of double-strand DNA breaks, we analyzed their genotoxic impact *in vitro* using the comet assay. This analysis was conducted on two human CCA cell lines (EGI-1 and HUCCT1) and NHCs. Interestingly, CisPt did not induce significant DNA damage, as evidenced by the "tail moment", in either NHCs or CCA cells at tested concentrations. In contrast, Aurkines 16 and 18 induced remarkable DNA damage, specifically in human CCA cells. This was characterized by an increased relative amount of DNA in the comet tail and a greater migration of the genetic material from the nucleus. Importantly, no DNA damage was observed in NHCs, as they remained entirely unaffected by Aurkine compounds (Fig. 3A).

**Fig. 4:**
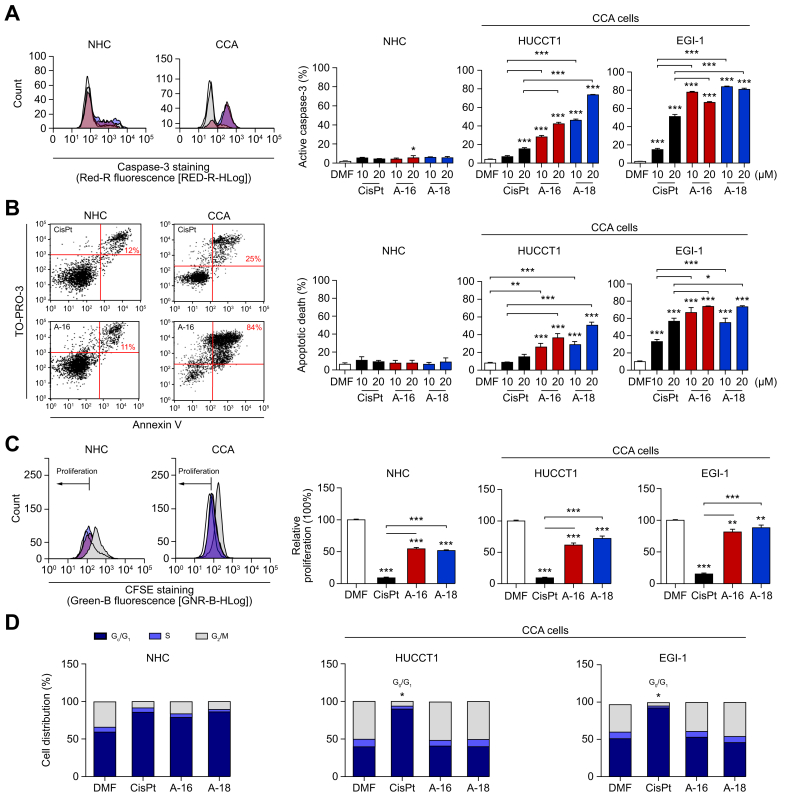
Pro-apoptotic effects of Aurkines 16 and 18 on human CCA cells. (A) % of cleaved caspase-3^+^ cells and (B) Annexin V/TO-PRO™-3 dual staining of NHCs and CCA cell lines (HUCCT1 and EGI-1) after 48-hour incubation with vehicle, CisPt, Aurkines 16 or 18 (10 μM and 20 μM). (C) Cell proliferation and (D) cell cycle analysis in NHCs and CCA cell lines (HUCCT1 and EGI-1) after 24-hour incubation with vehicle, CisPt, Aurkines 16 or 18 (10 μM). One-way ANOVA test or Student’s *t* tests were used. Data are shown as mean ± SEM. ∗*p* ≤0.05, ∗∗*p* ≤0.01, ∗∗∗*p* ≤0.001. CCA, cholangiocarcinoma; CisPt, cisplatin; DMF, dimethylformamide; NHCs, normal human cholangiocytes.

In agreement with the increased DNA damage, flow cytometry analysis demonstrated that both Aurkines 16 and 18 increased total and mitochondrial ROS levels specifically in CCA cells (Fig. 3B,C). This increased oxidative stress correlated with a marked reduction in CCA cell viability following treatment with Aurkines 16 and 18, compared to the milder effects observed with CisPt (Fig. 3D). On the other hand, in contrast to Aurkine 16, Aurkine 18 induced a slight increase in mitochondrial ROS in NHCs, similar to the effect of CisPt (Fig. 3C), all of which resulted in a mild decrease in cell viability (Fig. 3D). To further investigate these antitumor effects, we examined whether the observed reduction in CCA cell viability could be attributed to a decrease in proliferation and/or an increase in apoptosis. Flow cytometry revealed that both Aurkines 16 and 18 selectively targeted cancer cells, promoting greater apoptosis in CCA cells compared to CisPt, while leaving NHCs unaffected (Fig. 4A,B). Of note, while the addition of gemcitabine significantly enhanced the apoptotic effect of CisPt, it provided little additional benefit to the pronounced effect of Aurkines (Fig. S5). Besides, treatment with Aurkines 16 and 18 led to a reduction in the proliferation of surviving CCA cells, although this effect was less pronounced than that of CisPt (Fig. 4C). Moreover, CisPt induced cell cycle arrest in the G_0_/G_1_ phase in both CCA cell lines, associated with p-CDC2 activation, explaining its robust antiproliferative effect on CCA cells. In contrast, Aurkines 16 and 18 did not induce cell cycle arrest in CCA cells, aligning with their milder effect on CCA cell proliferation. In NHCs, both CisPt and Aurkine compounds induced cell cycle arrest in the G1 phase through p-CDC2 activation (Figs. 4D and S6). Nevertheless, the antiproliferative effect of Aurkines 16 and 18 on NHCs was significantly lower than that of CisPt, suggesting milder pharmacodynamic interaction of Aurkines on normal cells compared to CisPt (Fig. 4C). Additionally, Aurkines 16 or 18 also reduced the growth of 3D CCA spheroids (Fig. S7A) and cell viability in PDOs (from two patients with CCA) compared to both vehicle and CisPt (Fig. S7B).

**Fig. 5:**
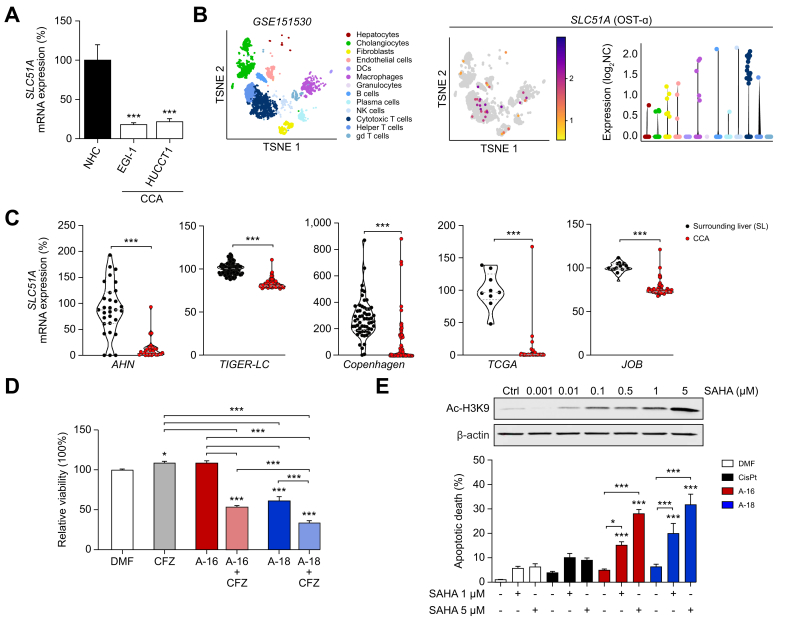
Mechanisms of NHC resistance to Aurkines. (A) *SLC51A* mRNA expression in NHC and CCA cell lines (HUCCT1 and EGI-1). (B) t-SNE plot clustering all the cell populations detected by scRNA sequencing in the GSE151530 dataset, which includes samples from 12 patients with CCA. (C) *SLC51A* mRNA expression levels in CCA tumors compared to SL tissue from the AHN, TIGER-LC, Copenhagen, TCGA and JOB human cohorts. (D) NHC viability after 48-hour incubation with Aurkines 16 and 18 (20 μM), or their combination with the OST-α/β inhibitor (CFZ, 10 μM). (E) Immunoblot of Ac–H3K9 in NHCs after 48-hour incubation with SAHA, with β-actin as loading control. Apoptosis of NHCs after 48-hour incubation with CisPt, Aurkines 16 and 18 (10 μM), or their combination with SAHA (1 μM and 5 μM). One-way ANOVA test or Student’s *t* tests were used. Data are shown as mean ± SEM. ∗*p* ≤0.05, ∗∗*p* ≤0.01, ∗∗∗*p* ≤0.001. CCA, cholangiocarcinoma; CFZ, clofazimine; CisPt, cisplatin; DMF, dimethylformamide; NHCs, normal human cholangiocytes; OST, organic solute transporter; SAHA, suberoylanilide hydroxamic acid; SL, surrounding liver.

To understand the selective toxicity of Aurkines against tumor cells, we investigated the potential role of organic solute export transporters. We observed that the mRNA expression levels of organic solute transporter alpha (*SLC51A*), which encodes the alpha subunit of OST-α/β, a well-characterized exporter of different organic substrates in epithelial cells, were higher in NHCs compared to CCA cells (Fig. 5A). Analysis of publicly available scRNA-seq data from 12 patients with CCA (GSE151530) confirmed the almost complete absence of *SLC51A* (OST-α) expression in CCA cells and other cell types within the tumor (Fig. 5B). Similarly, the expression of *SLC51A* was higher in surrounding liver tissues than in CCA samples (Fig. 5C). Importantly, the role of OST-α/β in NHC resistance to Aurkines was further demonstrated by co-incubating cells with clofazimine (CFZ), an OST-α/β inhibitor, which significantly reduced NHC viability when combined with Aurkines (Fig. 5D). Moreover, differences in DNA packaging between normal and cancer cells may also influence Aurkine efficacy. In fact, the combination of SAHA (suberanilohydroxamic acid), a histone deacetylase pan-inhibitor that induces DNA unpacking, with Aurkines 16 and 18, promoted apoptosis in normal cells (Fig. 5E).

### Evaluation of the antitumor effect of Aurkines 16 and 18 on CisPt-resistant human CCA cells

**Fig. 6:**
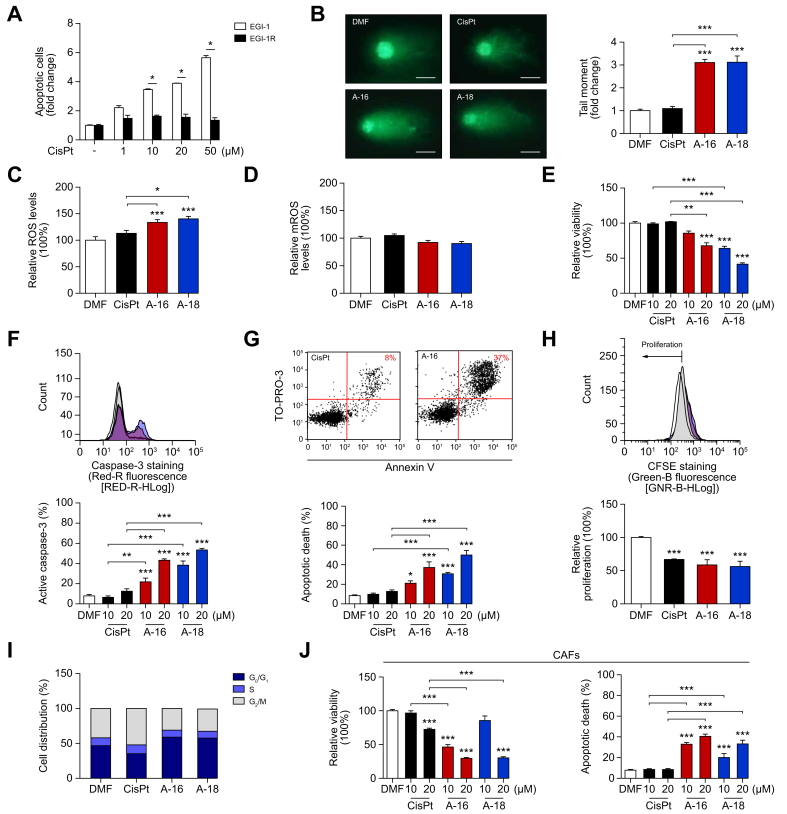
Antitumor effect of Aurkine 16 and 18 on CisPt-resistant CCA cells (EGI-1R) and CAFs. (A) Apoptosis in EGI-1 and EGI-1R cells after 48-hour incubation with increasing CisPt doses. (B) DNA damage, (C) ROS and (D) mROS in EGI-1R cells after 24-hour incubation with vehicle, CisPt, Aurkines 16 or 18 (10 μM). (E) Cell viability, (F) % of cleaved caspase-3^+^ cells and (G) Annexin V/TO-PRO™-3 dual staining of EGI-1R cells after 48-hour incubation with vehicle, CisPt, Aurkines 16 or 18 (10 μM and 20 μM). (H) Cell proliferation and (I) cell cycle analysis in EGI-1R cells after 24-hour incubation with vehicle, CisPt, Aurkines 16 or 18 (10 μM). (J) Cell viability and apoptosis of CAFs after 48-hour incubation with vehicle, CisPt, Aurkines 16 or 18 (10 and 20 μM). One-way ANOVA test or Student’s *t* tests were used. Data are shown as mean ± SEM. ∗*p* ≤0.05, ∗∗*p* ≤0.01, ∗∗∗*p* ≤0.001. CAFs, cancer-associated fibroblasts; CCA, cholangiocarcinoma; CisPt, cisplatin; DMF, dimethylformamide; mROS, mitochondrial ROS; ROS, reactive oxygen species.

Considering that resistance to CisPt remains a major obstacle in chemotherapy response, our focus shifted to evaluating the therapeutic potential of these novel compounds in overcoming CisPt resistance. To accomplish this, we generated a CCA cell line resistant to CisPt (EGI-1R) by subjecting EGI-1 cells to increasing doses of CisPt, until resistance was validated (Fig. 6A). In contrast to CisPt, both Aurkines 16 and 18 induced pronounced DNA damage in EGI-1R cells (Fig. 6B). Additionally, these compounds led to an increase in intracellular ROS levels compared to CisPt (Fig. 6C). Although Aurkines 16 and 18 did not initiate ROS formation in the mitochondria (Fig. 6D), this could be due to high baseline oxidative stress arising as a consequence of repetitive cycles of CisPt exposure. Consistent with these results, Aurkines 16 and 18 markedly reduced EGI-1R cell viability (Fig. 6E), inducing apoptosis, a response not triggered by CisPt (Fig. 6F,G). In the cells that remained alive, both Aurkines 16 and 18 slightly reduced cell proliferation to the same extent as CisPt (Fig. 6H), despite not causing any cell cycle arrest (Fig. 6I).

Notably, Aurkines 16 and 18 also exhibited a dose-dependent reduction in cell viability in an ovarian cancer cell line and two breast cancer cell lines, all resistant to CisPt (Fig. S8A). Moreover, Aurkines 16 and 18 induced apoptosis in the CisPt-resistant ovarian cancer cell line, a response not observed with CisPt (Fig. S8B).

### Evaluation of the effect of Aurkines 16 and 18 on CCA tumor microenvironment

CCA tumors are characterized by an extensive desmoplastic stroma that supports tumor growth and dissemination, contributing to unfavorable patient outcomes and limited treatment response. To explore the potential antitumor effects of Aurkine compounds on the CCA tumor microenvironment (TME), we investigated their impact on CAFs isolated from human CCA tumors – a pivotal component of the TME and a potential mediator of drug resistance. Strikingly, Aurkines 16 and 18 reduced CAF viability and increased their cell death rate (Fig. 6J), while no discernible effects were observed after incubation with CisPt. These findings provide insights into the cytotoxic potential of Aurkine compounds, suggesting their capacity to extend beyond direct effects on cancer cells and influence components of the TME, particularly CAFs.

### Analysis of the activity of Aurkines 16 and 18 on CCA tumors *in vivo*

**Fig. 7:**
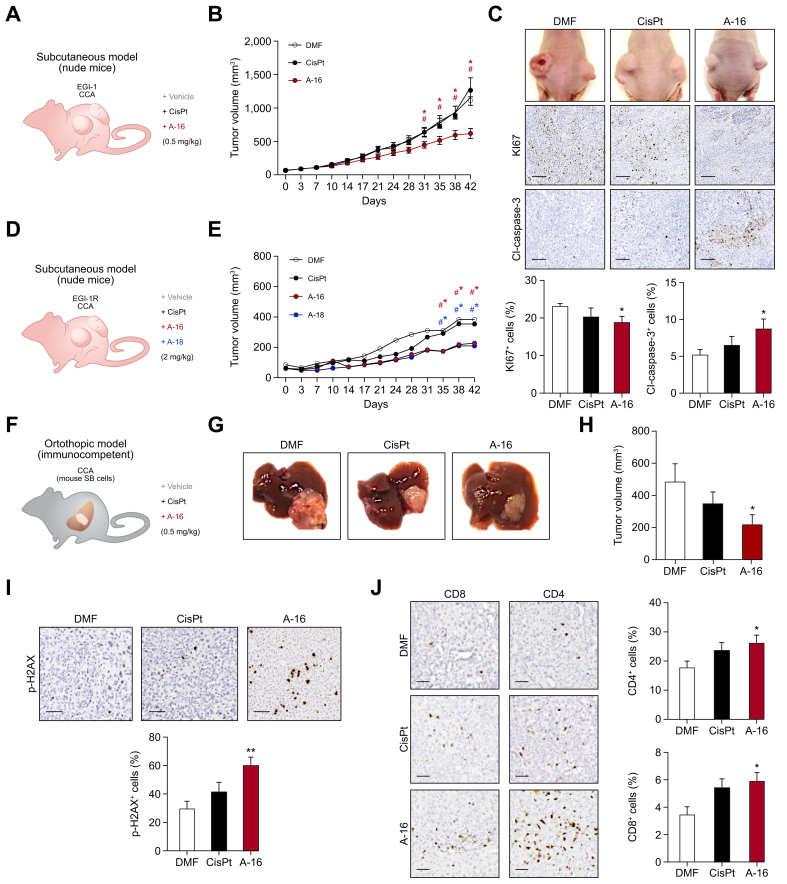
*In vivo* antitumor activity of Aurkines 16 and 18 in experimental murine models of CCA. (A) Schematic representation of the subcutaneous CCA model. (B) Tumor volume growth during treatment with CisPt or Aurkine 16 (0.5 mg/kg) in the subcutaneous CCA model. Group sizes: vehicle control (n = 12), CisPt (n = 11), Aurkine 16 (n = 13). (C) Representative tumor images, along with Ki67 and cleaved caspase-3 staining images and quantification in the subcutaneous CCA model. (D) Schematic representation of the subcutaneous CisPt-resistant CCA model. (E) Tumor volume growth during treatment with CisPt, Aurkines 16 and 18 (2 mg/kg) in the subcutaneous CisPt-resistant CCA model. Group sizes: vehicle control (n = 13), CisPt (n = 10), Aurkine 16 (n = 10), Aurkine 18 (n = 16). (F) Schematic representation of the orthotopic CCA model. (G) Representative macroscopic images of liver tumors from vehicle-, CisPt-, and Aurkine 16-treated animals. (H) Tumor volume at sacrifice following treatment with CisPt and Aurkine 16 (0.5 mg/kg) in the orthotopic CCA model. Group sizes: vehicle control (n = 16), CisPt (n = 16), Aurkine 16 (n = 16). (I) Representative images and quantification of p-H2AX staining in the orthotopic CCA model. (J) Representative images and quantification of CD4 and CD8 staining in the orthotopic CCA model. One-way ANOVA test or Student’s *t* test were used. Data are shown as mean ± SEM. *p* values (B, E): #*p* <0.05, compared to vehicle-treated; ∗*p* <0.05, compared to CisPt-treated. *p* values (C, H-J): ∗*p* ≤0.05, ∗∗*p* ≤0.01, ∗∗∗*p* ≤0.001. Scale bar: 100 μm. CCA, cholangiocarcinoma; CisPt, cisplatin; DMF, dimethylformamide.

To further investigate the therapeutic potential of Aurkines 16 and 18 in CCA, both compounds were tested in immunodeficient subcutaneous and immunocompetent orthotopic murine CCA models. Initially, Aurkines were administered at a 2 mg/kg dose, a dose known to inhibit CCA tumor growth by CisPt. Although both Aurkines 16 and 18, along with CisPt, noticeably delayed tumor growth compared to the vehicle control group, no statistically significant differences were observed among the treatment groups (Fig. S9A and B). Subsequently, the treatment dose of all compounds was reduced to 0.5 mg/kg, a dosage considered ineffective for CisPt (Fig. 7A). Remarkably, while CisPt showed no therapeutic effect on tumor growth at this reduced dosage, Aurkine 16 halted tumor progression over time (Fig. 7B,C). This was accompanied by a decrease in Ki67^+^ proliferative cells and an increase in cleaved caspase-3^+^ apoptotic cells (Fig. 7C). Importantly, no evidence of systemic toxicity was observed (Fig. S9C and D). Notably, in mice subcutaneously injected with CisPt-resistant CCA cells, both Aurkines 16 and 18, at a 2 mg/kg dose, effectively arrested tumor growth, in contrast to vehicle- or CisPt-treated animals (Fig. 7D, E). Furthermore, in an orthotopic CCA model, Aurkine 16 significantly reduced tumor volumes compared to controls (Fig. 7F–H), along with an increase in p-H2AX^+^ cells (Fig. 7I) and enhanced infiltration of CD8^+^ and CD4^+^ T cells into tumors (Fig. 7J). Importantly, no signs of hematological, renal, or hepatic toxicity were observed after treatment with Aurkine (Figs. S10 and S11), a finding further confirmed by toxicological studies with escalating doses in healthy C57BL/6J mice (Figs. S12–14).

### Evaluation of the main transporters involved in the uptake of Aurkines 16 and 18 by cancer cells

**Fig. 8:**
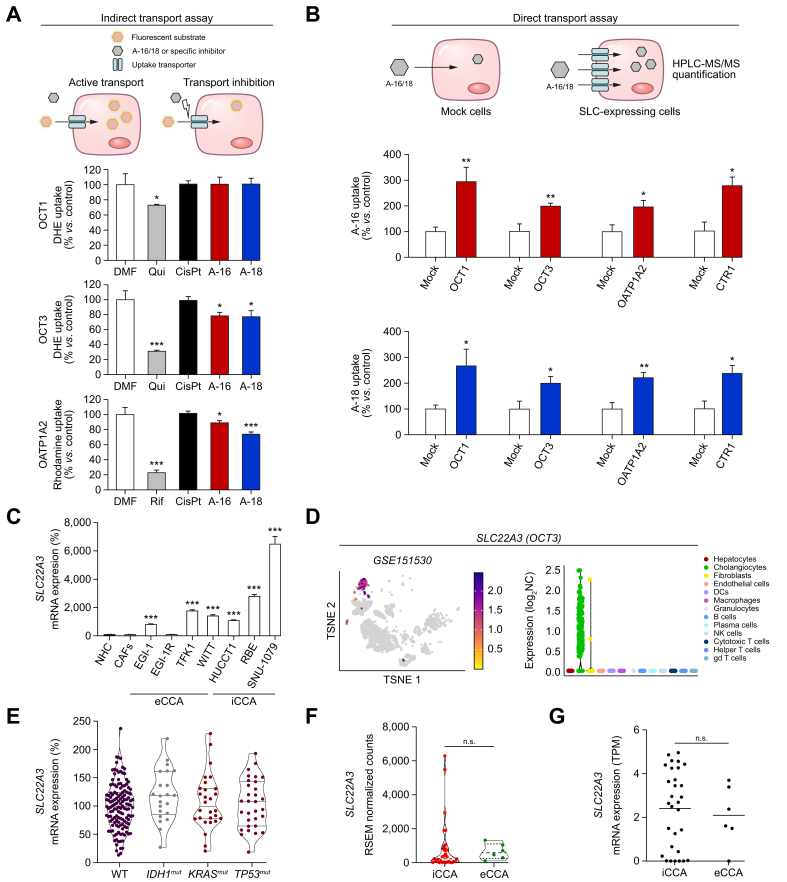
Analysis of transporters involved in the uptake of Aurkines 16 and 18 by cancer cells. (A) Uptake of specific fluorescent substrates by cells with or without experimental overexpression of each transporter, measured by flow cytometry. (B) Intracellular accumulation of Aurkine 16 and 18 in cells with or without overexpression of each transporter, measured by HPLC-MS/MS. (C) Relative mRNA expression (qPCR) of *SLC22A3* in NHCs, CAFs, eCCA (*i.e.* EGI-1, EGI-1R, TFK1, WITT) and iCCA (*i.e.* HUCCT1, RBE and SNU-1079) cells. (D) Expression levels of *SLC22A3* at the single-cell level in human CCA tumors. (E) mRNA expression of *SLC22A3* in CCA tissues, stratified by mutational status (*IDH1, KRAS, TP53* or WT). (F) mRNA expression of *SLC22A3* in CCA tissues, stratified by anatomical origin (iCCA *vs*. eCCA). (G) mRNA expression of *SLC22A3* in CCA cell lines, stratified by anatomical origin (iCCA *vs*. eCCA). One-way ANOVA test or Student’s *t* tests were used. Data are shown as mean ± SEM. ∗*p* ≤0.05, ∗∗*p* ≤0.01, ∗∗∗*p* ≤0.001. CisPt, cisplatin; CTR, copper transporter; DCs, dendritic cells; DMF, dimethylformamide; eCCA, extrahepatic cholangiocarcinoma; iCCA, intrahepatic cholangiocarcinoma; NHCs, normal human cholangiocytes; OATP, organic anion transporting polypeptide; OCT, organic cation transporter; Qui, quinine; Rif, rifampicine; WT, wild-type. This figure was partly generated using Servier Medical Art, provided by Servier, licensed under a Creative Commons Attribution 3.0 unported license.

To elucidate the mechanisms accounting for Aurkines 16 and 18 uptake by cancer cells, cell models with forced expression of plasma membrane transporters involved in the uptake of anionic and cationic drugs by liver cells were used to carry out indirect and direct transport assays. Our observations indicated that Aurkine compounds were transported into the cells by the organic cation transporters, OCT1 and OCT3, the organic anion transporting polypeptide, OATP1A2, and the copper transporter, CTR1 (Figs. 8A,B and S15A). Importantly, none of these transporters, except for CTR1, were found to be involved in the uptake of CisPt (Figs. 8A and S15B). In contrast, OATP1B3 and OATP2B1 did not seem to participate in the transport of Aurkine compounds (Fig. S15A and C). Of note, the expression (mRNA) of *SLC22A3* (OCT3), *SLC22A1* (OCT1), and *SLC31A1* (CTR1) was increased in CCA cell lines compared to NHCs (Figs. 8C and S15D). Among these, scRNA-seq data confirmed that *SLC22A3* (OCT3) is specifically expressed in cholangiocytes within human CCA tumors (Figs. 8D and S16A), regardless of their underlying mutation status (*IDH1, KRAS, TP53*, or wild-type) (Figs. 8E and S16B) or anatomical origin (Figs. 8F,G and S16C,D). Moreover, *SLC22A1* was also found upregulated in CAFs compared to NHCs, which could explain, at least in part, the cytotoxic effect of Aurkines on CAFs (Fig. S15D).

## Discussion

Since CisPt was approved in 1978, the range of platinum (Pt)-based cancer therapies has expanded to include seven additional agents, opening alternative treatment options. These drugs have become foundational in oncology, used in approximately 50% of cancer cases, depending on the type and stage of the cancer.^21^ However, their clinical utility is often limited by potential side effects and the rapid development of drug resistance.^7^ Up to 70% of patients either have intrinsic resistance to CisPt or develop it quickly during treatment, significantly reducing its therapeutic effectiveness.^8^ This challenge has spurred extensive research, evidenced by over 12,000 publications in 2023 alone, underscoring the urgent need for new strategies to overcome CisPt resistance (Source: SCOPUS).

All existing Pt-based chemotherapeutic agents, such as CisPt, oxaliplatin, and carboplatin, operate through a well-understood mechanism.^8^^,^^22^ These compounds primarily bind to DNA, inducing single-strand DNA breaks in about 95% of cases.^12^^,^^13^ However, these breaks are relatively easy for cancer cells to repair, enabling survival and drug resistance. In contrast, interstrand crosslinks that result in double-strand DNA breaks are much more challenging for cancer cells to repair,^15^ though they occur in less than 5% of cases.^12^^,^^13^ To address these limitations, we have designed and synthesized two novel chemotherapeutic agents, Aurkine 16 and Aurkine 18. These innovative compounds feature marked polyelectrophilic properties that promote up to three nucleophilic substitution reactions, thus enhancing formation of interstrand DNA crosslinks, leading to increased double-strand DNA breaks. This approach hampers the activation of DNA repair mechanisms, resulting in greater cancer cell death and a reduced likelihood of resistance. The synthesis of Aurkines 16 and 18 is efficient and cost-effective, starting with readily available reagents and producing high yields, ensuring scalability and resource efficiency. The synthesis process is robust, supporting further exploration and optimization of these compounds. The purification of the final products is straightforward, avoiding complex and costly procedures, enabling synthesis on a gram scale and beyond.

Our molecular studies, coupled with phosphoproteomic analysis, demonstrate that Aurkines and CisPt operate through distinct mechanisms of action. CisPt mainly induces single-strand DNA breaks, which activate DNA repair pathways, leading to cell cycle arrest and reduced cell proliferation. However, as these single-strand breaks are relatively easy to repair, cells often restore their DNA structure, allowing them to survive and avoid cell death. In contrast, Aurkines induce double-strand DNA breaks, a more challenging form of damage for cancer cells to repair. This results in elevated oxidative stress within the cell and mitochondria, triggering caspase activation and ultimately leading to cell death. Notably, Aurkines have shown cytotoxic effects not only in CisPt-sensitive CCA cells but also in CisPt-resistant cancer cells from CCA, ovarian, and breast cancers. This demonstrates their therapeutic potential in overcoming CisPt resistance, a significant challenge in cancer treatment.^23^ These findings have been further validated in various subcutaneous and orthotopic cancer mouse models, demonstrating the superior efficacy of Aurkines compared to CisPt.

The lack of selectivity of chemotherapies for tumor cells remains a major problem, often leading to significant side effects. However, our study highlights the pronounced selectivity of Aurkine compounds for cancer cells and CAFs. These compounds demonstrate minimal toxicity to normal cells, likely due to a combination of different mechanisms, including reduced uptake via OCT1, OCT3 and CTR1, increased efflux via OST-α/β, and the presence of a compact chromatin structure that limits Aurkine-DNA interactions. Additionally, toxicological studies in healthy C57BL/6J mice, along with stable body weight in treated animals, showed no apparent signs of hematological, hepatic, or renal toxicity after Aurkine administration. These findings reinforce the potential safety and efficacy of Aurkines for treating CCA. Pending further translational and clinical research, they could offer a safe alternative to conventional chemotherapy with a potentially wider therapeutic window.

Our research also addresses the critical interaction between tumor cells and the stroma, especially in CCA, where the extensive TME can impact cancer progression and chemoresistance.^24^ Aurkines have demonstrated superior effectiveness compared to CisPt in reducing the viability of CAFs and inducing their apoptosis. This represents a significant advance, as CAFs are known to be resistant to conventional chemotherapy.^25^ By targeting both cancer and stromal cells, Aurkines could disrupt the signals that promote tumor growth, thereby creating a more hostile environment for cancer progression. Furthermore, Aurkines promote the recruitment of CD4^+^ and CD8^+^ T cells into the TME, which could potentially enhance the effectiveness of immunotherapy, which warrants further investigation.

In conclusion, this study provides compelling evidence of the significant therapeutic potential of Aurkines 16 and 18, highlighting their importance not only in treating CCA but also in addressing various other cancers. The research reveals their unique mechanism of action, distinguishing them from CisPt. These compounds show promise in treating both naïve and CisPt-resistant tumors – a major challenge in cancer treatment – and have substantial effects on stromal and immune cells within the tumor microenvironment. The notable absence of toxicity further underscores their safety profile, reinforcing their potential as effective treatments for CCA. Collectively, these findings position Aurkine compounds as an innovative therapeutic approach, supporting further scientific and clinical investigation.

## Abbreviations

A, adenine; AFM, atomic force microscopy; CAFs, cancer-associated fibroblasts; CCA, cholangiocarcinoma; CFZ, clofazimine; CisPt, cisplatin; CTR1, copper transporter 1; DCs, dendritic cells; DMF, dimethylformamide; eCCA, extrahepatic CCA; G, guanine; GemCis, gemcitabine + cisplatin; iCCA, intrahepatic CCA; IHC, immunohistochemistry; mROS, mitochondrial ROS; NHCs, normal human cholangiocytes; OATP, organic anion transporting polypeptide; OCT, organic cation transporter; OST, organic solute transporter; PDO, patient-derived organoids; Pt, platinum; Qui, quinine; Rif, rifampicine; ROS, reactive oxygen species; SAHA, suberanilohydroxamic acid; SC, supercoiled; scRNA-seq, single-cell RNA sequencing; SL, surrounding liver; TEM, transmission electron microscopy; TME, tumor microenvironment; WT, wild type.

## Financial support

This study was competitively funded by the Department of Health of the Basque Government “Euskadi RIS3” [2019222054, 2020333010, and 2021333003 to JMB and FPC] and Instituto de Salud Carlos III (ISCIII) “PI24/00148” to JMB, co-funded by the European Union. Other funding sources not involved in study design and analysis, decision to publish, or preparation of the article include: Instituto de Salud Carlos III (ISCIII) “PI21/00922” to JMB, “PI23/01565 and PI20/00186” to MJP, and “PI23/01850” to PMR, co-funded by the European Union; ISCIII-FIS grant “PI18/01075” to JMB, co-financed by ERDF (FEDER) Funds from the European Commission; ISCIII “PMP22/00054” to JMB, co-funded by the European Union NextGenerationEU/PRTR; ISCIII “FORT23/00026” to JMB; Sara Borrell CD19/00254 to PMR and Miguel Servet CP22/00073 to PMR; CIBERehd (ISCIII). Spanish Ministry of Economy and Competitiveness (MINECO) “Ramón y Cajal” Program “RYC-2015-17755” to MJP; “Diputación Foral Gipuzkoa” (2020-CIEN-000067-01, 2021-CIEN-000029-04-01 and 2023-CIEN-000008-01 to PMR; 2023-CIEN-000045-01 to MJP; 2024-CIE4-000015-01 to LI-S); Department of Education of the Basque Government [IT-1761-22 to LB]; Department of Health of the Basque Country [2023111017 to MJP; 2022111070 to PMR; 2024111044 and 2020111077 to JMB; 2017111010 to JMB], “Euskadi RIS3” [2022333032, 2023333005 and 2024333004 to JMB], and Elkartek (KK-2020/00008 to JMB; KK-2023/00099 to JMB, MJP); La Caixa Scientific Foundation [HR17-00601 to JMB]; Scientific Foundation of the Spanish Association Against Cancer (AECC23/502 to PMR, POSTD246369IZQU to LI-S, PRYGN246692BANA to JMB); AMMF-The Cholangiocarcinoma Charity (EU/2019/AMMFt/001 to JMB and PMR). PSC Partners US (to JMB) and PSC Supports UK (06119JB to JMB). European Research Council, [FPC (ERC-2020-SyG 951281)]; European Commission [ARG (MSCA 101026616 – SN2DNA)]; Spanish Ministry of Science, Innovation and Universities [FPC, IR. MO-G (MCIN/AEI/PID2019-104772GB-I00 and MCIN/AEI/RED2018-102387-T)]; Basque Government [FPC, IR, ARG,MO-G (IT-1553-22)]; Junta de Castilla y Leon (SA074P20 and SA113/P23 to JJGM); Instituto de Salud Carlos III (ISCIII) "PI22/00526" to JJGM; Department of Education of the Basque Country (IT1584-22 to XL and DdS); Spanish Ministry of Science and Universities (MINECO/FEDER): PID2021-127907NB-I00 to XL and DdS; EU MSCA (101072645 - "nanoremedi") to AMB; Spanish Ministry of Science and Innovation (CEX2020-001038-M/MCIN/AEI/10.13039/501100011033, PID2019-104650GB-C22 and PID2023-147987OB-C32 to AMB) and Basque Government - AZPITEK grant to AMB.

## Authors’ contributions

FPC and JMB conceived and coordinated the project. IR and MOG completed the chemical synthesis of the compounds. ARG, DdS and XL performed the computational studies. AEL, AMB, AMA and TM performed the AFM and TEM experiments. IR and IO arranged the organization of chemical and biological data. IO, PO, FJCC, NPT, MTG, MHI, CR and PMR completed the functional experiments. MA, OB, EH and JJGM performed the transport assays. AL and BV performed the bioinformatic analysis. IO, MOG, PO, FJCC, NPT, MTG, AL, BV, MA, ARG, MHI, CR, OB, LIS, PMR, MJP, LB, JJGM, FPC, and JMB completed the experiments as well as the collection, analysis and interpretation of data. IO, FPC and JMB wrote the paper with contributions of all the authors. All authors read and critically revised the manuscript and approved the final version. PMR, MJP, LB, JJGM, FPC, and JMB obtained funding.

## Data availability

Phosphoproteomic data have been deposited in the ProteomeXchange Consortium via the PRIDE (PRoteomics IDEntifications Database) repository under the identifier PXD061935. Single-cell transcriptome data from cholangiocarcinoma tumors were downloaded from the Gene Expression Omnibus (GEO) dataset under accession number GSE151530. Data from the DONG database (OEP001105) were retrieved from the Biosino NODE repository (https://www.biosino.org/node/project/detail/OEP001105). Batch-corrected gene expression data from the DepMap 24Q4 dataset (https://depmap.org/portal) were analyzed for intraductal papillary neoplasm of the bile duct CCA cell lines. Transcriptomic analyses were performed on CCA tumors and adjacent non-tumor liver tissues using publicly available datasets from five independent patient cohorts: AHN (GSE107943), The Thailand Initiative in Genomics and Expression Research (TIGER-LC; GSE76297), Copenhagen (GSE26566), Cancer Genome Atlas (TCGA-CHOL) and JOB (E-MATB-6389). All datasets used in this study are publicly available and can be accessed via the corresponding repositories.

## Conflict of interest

FPC is scientific advisor of Quimatryx Ltd (quimatryx.com). Remaining authors have no conflicts of interest to declare related to this manuscript.

Please refer to the accompanying ICMJE disclosure forms for further details.
