# Supplementary material for: New platinum derivatives selectively cause double-strand DNA breaks and death in naïve and cisplatin-resistant cholangiocarcinomas
Source: J Hepatol. 2025 Nov;83(5):1077–91. doi: 10.1016/j.jhep.2025.04.034 (PMC12547501; doi:10.1016/j.jhep.2025.04.034)
Supplement: Multimedia component 1 [file mmc1.pdf]

Supplementary information – materials and methods

New platinum derivatives selectively cause double-strand DNA breaks and death in naïve and cisplatin-resistant cholangiocarcinomas

Irene Olaizola, Mikel Odriozola-Gimeno, Paula Olaizola, Francisco J. Caballero-Camino, Noelia Pastor-Toyos, Mireia Tena-Garitaonandia, Ainhoa Lapitz, Beatriz Val, Amanda R. Guimaraes, Maitane Asensio, Maider Huici-Izagirre, Colin Rae, David de Sancho, Xabier Lopez, Pedro M. Rodrigues, Elisa Herraez, Oscar Briz, Laura Izquierdo-Sanchez, Aitziber Eleta-Lopez, Alexander M. Bittner, Ana Martinez-Amesti, Teresa Miranda, Sumera I. Ilyas, Chiara Braconi, Maria J. Perugorria, Luis Bujanda, Iván Rivilla, Jose J.G. Marin, Fernando P. Cossio, Jesus M. Banales

Table of contents

Supplementary materials and methods 2

Supplementary figures 19

Supplementary tables 39

Supplementary references 42

Supplementary materials and methods

Cell cultures

Normal human cholangiocytes (NHC) were isolated from healthy liver tissue as previously detailed by our group.[1,2] Three human CCA cell lines were used: HUCCT1 (intrahepatic CCA sensitive to CisPt), EGI-1 (extrahepatic CCA sensitive to CisPt), and EGI-1R (extrahepatic CCA resistant to CisPt). The EGI-1R cell line was developed at the Biogipuzkoa Health Research Institute (BHRI, Spain) by subjecting EGI-1 cells to increasing CisPt concentrations, ranging from 5 μM to 30 μM, over a 48-hour period until resistance was effectively acquired and confirmed. Notably, to sustain the acquired resistance, CisPt (30 μM) was added to cells every two days. NHC, EGI-1 and EGI-1R cells were grown in fully-supplemented DMEM/F-12 medium as previously described,[2] whereas HUCCT1 cells were cultured in RPMI medium supplemented with 10% fetal bovine serum (FBS; Gibco) and 1% penicillin/streptomycin (P/S; Gibco). Cancer-associated fibroblasts (CAFs) were isolated from resected intrahepatic CCA at BHRI (Spain), purified, and cultured in DMEM 1X supplemented with 10% FBS and 1% P/S as previously described.[3] CAF phenotype was characterized analyzing the expression of the positive markers platelet derived growth factor receptor β (PDGFRβ), fibroblast activated protein 1 (FAP1), α-smooth muscle actin (α-SMA) and vimentin. Cytokeratin 19 (CK19) was used as a negative control (positive marker of cholangiocytes).[3] CCA cells (*i.e.,* EGI-1) and hepatic stellate cells (*i.e.,* LX2), were used as controls. Liver tissue was obtained according to the guidelines approved by the Ethics Committee of the hospital and prior signature of the pertinent written informed patient consent. Besides, Chinese hamster ovary (CHO) cells and a liver cancer cell line (HepG2) were used for the transport assays. CHO cells were grown in DMEM medium supplemented with 1% GlutaMAX™ and 0.43 mM L-proline, while HepG2 cells were cultured in DMEM medium supplemented with 2.2 g/L

sodium bicarbonate and 110 mg/L sodium pyruvate. All cell lines were tested for mycoplasma and were negative all along the experiments.

Establishment of Patient-derived Organoids (PDOs) from tissues

Human tissues were obtained from patients undergoing surgical resection at Glasgow Royal Infirmary, collected under approval of the Ethical Committee. All tissues were collected with informed consent and pathology reports confirmed the presence of cancer. Patient BB220682 was diagnosed with distal cholangiocarcinoma (T3N2R0) and underwent surgical resection without prior neo-adjuvant systemic treatment. This tumor stage indicates a locally advanced disease with regional lymph node involvement but negative surgical margins. Patient BB220452 had a duodenal adenocarcinoma (T4N2R1) and also underwent surgical resection without prior systemic therapy. This case represents a more aggressive disease with deeper invasion (T4), regional lymph node metastases (N2), and microscopically positive surgical margins (R1), indicating residual tumor presence.

For establishment of organoids, tissue digestion was adapted these two protocols. [4,5] Briefly, tissue was minced on ice using a scalpel and then incubated in digestion solution (collagenase, dispase and Y-27632 in basal medium (Advanced DMEM/F12, supplemented with HEPES buffer, Glutamax, Penicillin/Streptomycin, Amphotericin). Tissue was washed twice with cold basal medium then incubated in TrypLe solution (ThermoFisher Scientific, UK) and DNase I (Merck Life Sciences, UK). After washing in cold basal medium, cells were resuspended in growth factor-reduced Matrigel (Corning, UK) and domes placed in 24-well plates, then covered with organoid medium when Matrigel solidified. Medium was replaced every 3-4 days and organoids passaged by mechanical disaggregation. Presence of tumor cells was confirmed by hematoxylin and eosin (H&E) staining of fixed organoids. For drug screening, organoids were dissociated to single cells by TrypLe, then 10^4 cells in organoid medium containing 2% Matrigel added to Matrigel-coated wells of black-sided clear-bottom 96-well plates. After re-formation of organoids,

drugs were added and 72 hours later cell viability was measured by CellTiter-Blue® Assay (Promega, UK). The cell viability was normalized to that of the controls containing only vehicle. Each drug was tested at least in triplicate and statistical significance ($p<0.05$) was tested using student's t-test.

Transcriptomic analysis of human samples

Transcriptomic analyses were performed on CCA tumors and adjacent non-tumor liver tissues using datasets from five distinct publicly available patient cohorts: AHN (dataset: GSE107943),[6] The Thailand Initiative in Genomics and Expression Research (TIGER-LC; dataset: GSE76297),[7] Copenhagen (dataset: GSE26566),[8] Cancer Genome Atlas (TCGA-CHOL)[9] and JOB (dataset: E-MATB-6389).[10]

GSE107943 provided expression profiling by high-throughput sequencing using Illumina NextSeq 500 (GPL18573) and included 27 paired intrahepatic CCA (iCCA) tumor and adjacent normal liver samples. The TIGER-LC cohort (GSE76297) used microarray expression profiling with the [HTA-2_0] Affymetrix Human Transcriptome Array 2.0 (GPL17586) and contained 90 paired iCCA and adjacent normal liver samples. GSE26566 utilized microarray expression profiling with the Illumina humanRef-8 v2.0 expression beadchip (GPL6104) and included 104 CCA tumor samples and 59 surrounding normal liver samples. RNA sequencing data from TCGA-CHOL were obtained from the FireBrowse portal (Broad Institute of MIT & Harvard, USA; https://gdac.broadinstitute.org/) and comprised 36 CCA tumor samples, including 30 iCCA and 6 extrahepatic CCA (eCCA), which were further classified into 4 perihilar CCA (pCCA) and 2 distal CCA (dCCA). The E-MTAB-6389 dataset, retrieved from https://www.ebi.ac.uk/biostudies/arrayexpress/studies/E-MTAB-6389, contained 78 iCCA tumor samples and 31 non-tumor liver samples.

Data from the Gene Expression Omnibus (GEO) datasets (GSE107943, GSE76297, and GSE26566) were analyzed using GEO2R. The datasets from TCGA and E-MTAB-6389 were exported as batch-corrected, normalized versions for further analysis.

Gene expression and tumor mutational profile analysis

To assess the association between gene expression and tumor mutational profiles, data from the DONG database (OEP001105)[11] available in the Biosino NODE repository (https://www.biosino.org/node/project/detail/OEP001105) were analyzed. Specifically, RNA-seq transcript per million (TPM) values (Analysis ID: OEZ00008243) and whole exome sequencing (WES) data (Analysis ID: OEZ00008242) were used. Gene expression data were compared across four patient subgroups: iCCA tumors with *KRAS* mutation ($KRAS^{mut}$, $TP53^{wt}$, $IDH1^{wt}$), iCCA tumors with *TP53* mutation ($KRAS^{wt}$, $TP53^{mut}$, $IDH1^{wt}$), iCCA tumors with *IDH1* mutation ($KRAS^{wt}$, $TP53^{wt}$, $IDH1^{mut}$), and iCCA tumors wild-type for *KRAS, TP53* and *IDH1* ($KRAS^{wt}$, $TP53^{wt}$, $IDH1^{wt}$).

CCA cell line analysis

Batch-corrected gene expression data from the DepMap[12] Public 24Q4 dataset (https://depmap.org/portal) were analyzed for intraductal papillary neoplasm of the bile duct CCA cell lines. Intrahepatic CCA cell lines included SNU1079, HUH28, HUCCT1, KKU055, KKU213, ICC10, ICC106, ICC108, ICC12, ICC137, ICC15, ICC2, ICC3, ICC4, ICC5, ICC6, ICC7, ICC8, ICC9, HKGZCC, OZ, RBE, SG231, SSP25, TKKK, YSCCC, CCLP1, CCSW1, ICC16 and ICC18. Extrahepatic CCA cell lines analyzed were SNU245, SNU1196, EGI1, KKU100, TFK1 and ECC2.

Single-cell RNA sequencing analysis (scRNA-seq) of human iCCA tissue

Single-cell transcriptome profiling from CCA tumors was downloaded from Gene Expression Omnibus (GEO) dataset under accession number GSE151530.[13] This CCA tumor dataset

comprises the transcriptional profile of single cells obtained from 14 fresh liver tumor biopsies from 12 patients with iCCA.

Regarding data processing, the dataset was processed with Seurat R package version 5.1.0 and R version 4.3.3. Quality control metrics were computed, and cells with fewer than 500 detected genes, more than 4,000 detected genes, or over 20% mitochondrial gene expression were excluded. Normalization was performed using the SCTransform method, which regresses out the effects of mitochondrial content and RNA counts to minimize technical variability. Dimensionality reduction was applied to the analyzed data using Principal Component Analysis (PCA). For this, the top 2,000 variable genes were selected using the FindVariableFeatures function (vst method) in Seurat. Based on an elbow plot, the first 30 principal components (PCs) were retained for downstream analyses, including clustering and visualization. Unsupervised clustering was performed using the Leiden algorithm at multiple resolutions. For subsequent analyses, clusters generated at a resolution of 0.4 were selected as optimal. Dimensionality reduction techniques, including UMAP and t-SNE, were applied for visualizing the clusters.

Cell type annotation of clusters was based on the expression of canonical marker genes as follows: Hepatocytes (HPX, LBP, SERPINA10); Cholangiocytes (*KRT19*, *KRT7, FXYD2*); Fibroblasts (*COL1A1, DCN*); Endothelial cells (*FCN2, VWF, CDH5*); Dendritic Cells (DCs) (*IRF8*); Macrophages (*CD68, CD163, CSFIR*); Granulocytes (*FXYD2, IRF8*); B cells (*MS4A1, CD79A*); Plasma cells (*FCRL5, IGHM*); Natural Killer (NK) cells (*KLRD1, GNLY*); Cytotoxic T Cells (*CD3D, TRAC*); Helper T Cells (*IL7R, MAL*); γδ (gd) T cells (*TYMS*).

RNA isolation and gene expression

RNA was extracted from cell cultures using TRI Reagent® (Sigma-Aldrich). Reverse transcription of 1 µg RNA from cell samples was performed utilizing a mixture containing DNAse I Amplification Grade (Invitrogen – Thermo Fisher Scientific), M-MLV Reverse Transcriptase (Invitrogen),

RNaseOUT Recombinant Ribonuclease Inhibitor (Invitrogen – Thermo Fisher Scientific), Random Primers (Invitrogen – Thermo Fisher Scientific), and dNTPs (GE Life Sciences). The gene expression (mRNA) of specific primers sequences (Table S1) was determined by real-time quantitative polymerase chain reaction (qPCR) using iQ SYBR Green Supermix (Bio- Rad) in a CFX96 Touch Real-Time PCR Detection as previously described. Expression of *Glyceraldehyde-3-phosphate dehydrogenase (GAPDH)* was used as a housekeeping control for data normalization and gene expression was determined using the ΔCT method.

Histological analyses

Tissue samples were collected and fixed in 4% paraformaldehyde for 24 hours. Next, tissues were processed using the MTM tissue processor (Slee Medical GmbH), embedded in paraffin (Gibco – Thermo Fisher Scientific) and cut using the HM355S microtome (Gibco – Thermo Fisher Scientific) in sections at a thickness of 4-5 μm.

Immunohistochemistry (IHC) was performed in paraffin-embedded mouse liver tissue or tumoral sections. In order to remove the paraffin, slides were incubated in xylene and rehydrated in graded series of ethanol as previously described. Next, sections were placed on a 0.6% H2O2 (Sigma-Aldrich) in methanol (Applichem Panreac) solution for 15 minutes to block endogenous peroxidases. Following antigen retrieval with antigen unmasking solution (Vector Laboratories), slides were blocked using first the Avidin/Biotin Blocking Kit (Vector Laboratories) and later, a 20% swine bovine serum in DPBS 1X blocking buffer. Primary antibodies (Table S2) were incubated overnight at 4ºC. After washing the antibodies with DPBS 1X, slides were incubated with the appropriate biotinylated secondary antibodies. Vectastain ABC Reagent (Vector Laboratories) followed by 3,3 diaminobenzidine (DAB) peroxidase substrate Kit (Vector Laboratories) was used for antigen visualization. Slides were counterstained with Mayer's hematoxylin (Sigma-Aldrich), dehydrated and mounted as previously described. Representative pictures were taken in an Axioscan 7 (Zeiss) and images were analysed with QuPath software.

Immunoblotting

Whole cell lysates of cultured human cholangiocytes were extracted using radioimmunoprecipitation (RIPA) lysis buffer. Alterations in protein expression were evaluated through immunoblotting, using 10-30 µg of protein from the cellular extracts, which were separated using 7-12.5% SDS-PAGE and subsequently transferred to a nitrocellulose membrane (Bio-Rad). These membranes were blocked either with 5% BSA/TBS-0.1%Tween for phosphorylated proteins or 5% skim milk powder/TBS-0.1%Tween for non-phosphorylated forms. Membranes were then incubated with the relevant primary antibody (Table S2) overnight at 4ºC. Subsequently, horseradish peroxidase-conjugated secondary antibody was applied to the membranes and incubated for 1 hour at room temperature. Next, antibodies were exposed using the Novex® ECL HRP Chemiluminescent Substrate Reagent Kit (Invitrogen), and the emitted chemiluminescence was visualized and captured in the iBright CL1500 Imaging System (Invitrogen – Thermo Fisher Scientific). Protein signal quantification was performed using ImageJ (National Institutes of Health, USA).[14] β-actin protein levels were utilized to normalize protein loading.

Cell viability, proliferation, cell cycle and apoptosis

Cell viability was assessed using the Cell Proliferation WST-1 Assay (Roche) according to the manufacturer's instructions. Cells were seeded at a density of 2.5×10^3 cells per well in a collagen-coated 96-well plate in their respective culture media and incubated overnight at 37ºC. The next day, cells were exposed to two different concentrations (10 µM and 20 µM) of CisPt, Aurkines, or the vehicle solution for 48 hours. Finally, 10 µL of WST-1 reagent were added to each well, incubated at 37ºC for 1 hour and the signal was measured at 450 nm in a Halo LED 96® multiplate reader (Dynamic Scientific Ltd., UK).

Early apoptosis was evaluated by measuring caspase-3 activity (PhiPhiLux®-G_2D_2 kit, Oncoimmunin) through flow cytometry. Cells were seeded at a density of $2.5x10^4$ cells per well in a collagen-coated 24-well plate. After 24 hours, cells were incubated with two concentrations (10 μM and 20 μM) of CisPt, Aurkines, or the vehicle solution for 48 hours. Subsequently, cells were collected and stained with a substrate solution (10 μM) for 45 minutes at 37ºC. Fluorescence was then measured by flow cytometry using the *Guava easyCyte 8HT* Flow Cytometer (Merck Millipore).

Late apoptosis was evaluated using FITC Annexin V (BioLegend) and TO-PRO™-3 iodide (Invitrogen Thermo Fisher Scientific) by flow cytometry. Cells were seeded at a density of $2.5x10^4$ cells per well in a collagen-coated 24-well plate. After 24 hours, cells were incubated with two concentrations (10 μM and 20 μM) of CisPt, Aurkines, or the vehicle solution for 48 hours. Subsequently, cells were collected and stained with FITC Annexin V (BioLegend) for 15 minutes at room temperature and TO-PRO™-3 iodide (Invitrogen – Thermo Fisher Scientific) for 15 minutes at 4ºC. Fluorescence was measured by flow cytometry using the *Guava easyCyte 8HT* Flow Cytometer (Merck Millipore). Puromycin (2 μg/mL) was used as a positive control for cell death.

Cell proliferation rates were determined through flow cytometry, using the CellTrace™ CFSE Cell Proliferation Kit (Invitrogen – Thermo Fisher Scientific) in accordance with the manufacturer's instructions. Briefly, $2x10^4$ cells were collected, suspended in a 0.1% BSA in PBS solution, and labelled with CFSE at a 5 mM concentration. Afterwards, 5 volumes of cold medium were added, and the tubes were incubated for 5 minutes on ice. Subsequently, cells were washed three times with cold media, resuspended in their respective culture media, and seeded in a collagen-coated 12-well plate. The cells were allowed to attach overnight, followed by incubation with CisPt, Aurkines or vehicle solution (10 μM) for 24 hours (the vehicle solution was used as control). The cells were then trypsinized, centrifuged, and resuspended in DPBS 1X (Gibco – Thermo Fisher

Scientific) before being placed in a U-bottom 96-well plate, following the flow cytometer manufacturer's instructions. Fluorescence was measured using the *Guava easyCyte 8HT* Flow Cytometer (Merck Millipore).

The cell cycle distribution was assessed through flow cytometry utilizing TO-PRO™-3 iodide (Invitrogen – Thermo Fisher Scientific). Approximately $1x10^5$ cells were harvested, washed with DPBS 1X, and fixed with ice-cold 70% ethanol overnight at -20ºC. After centrifugation at 8,000 rpm for 5 minutes, the cell pellets were stained with a DPBS 1X solution containing TO-PRO™-3 iodide (Invitrogen – Thermo Fisher Scientific) and RNAse A (Sigma), followed by a 30-minute incubation at 37ºC in the dark. Subsequently, the samples were transferred to a U-bottom 96-well plate following the manufacturer's instructions and analyzed using the *Guava easyCyte 8HT* Flow Cytometer (Merck Millipore).

Reactive oxygen species (ROS) detection

To study cellular oxidative stress, the CellROX™ Deep Red Reagent (Invitrogen – Thermo Fisher Scientific) was utilized following the manufacturer's guidelines. Briefly, $2x10^5$ cells were seeded overnight in thin collagen-coated 12-well plates with their corresponding culture media. The next day, cells were incubated with CisPt, Aurkines, or the vehicle solution (10 µM) for 24 hours. Subsequently, cells were trypsinized and incubated with 25 µM CellROX™ Deep Red Reagent (Invitrogen) for 1 hour at 37ºC. Fluorescence was then measured using the *Guava easyCyte 8HT* Flow Cytometer (Merck Millipore).

To evaluate mitochondrial oxidative stress, the MitoSOX™ Red Reagent (Invitrogen) was employed following the manufacturer's instructions. Initially, $2x10^5$ cells were seeded overnight in thin collagen-coated 12-well plates with their corresponding culture media. The next day, cells were incubated with CisPt, Aurkines, or the vehicle solution (10 µM) for 24 hours. After this incubation period, the medium was removed, and cells were incubated with 1 µL of MitoSOX

reagent per well in 250 μL of their respective culture media for 20 minutes at 37ºC. Subsequently, cells were trypsinized, washed twice with DPBS 1X, and finally transferred to a U-bottom 96-well plate for analysis using the *Guava easyCyte 8HT* Flow Cytometer (Merck Millipore).

3D Spheroids

To generate CCA spheroids, $3x10^3$ human CCA cells were seeded in 50 μL of complete spheroid medium (DMEM F12 + Glutamax + P/S + EGF + B27 + Insulin) in a 96-well U-bottom plate (Greiner bio-one). The plate was then centrifuged at 400 g for 10 minutes and maintained at 37ºC with 5% CO2 for 24 hours to allow spheroid formation. The following day, once the spheroids had formed, 50 μL of normal medium (DMEM-F12 + P/S + Glutamax) was added to each well. Subsequently, CisPt or Aurkines at a concentration of 10 μM (or vehicle solution as control) were added in 100 μL of medium to each well. Calcein was used to stain the spheroids. The size of the CCA spheroids was measured at baseline (0 hours) and 48 hours after treatment using ImageJ software version 1.50 (NIH, Bethesda, MA, USA), and photographs were taken during the process using the Axio Observer 7 fluorescent microscope.

Atomic Force Microscopy and Transmission Electron Microscopy

DNA samples were prepared from lyophilized lambda phage DNA, methylated, from *E. coli* host strain W3110 (Mw. 31.5 x 10^3 kDa, 48 kb, from Sigma-Aldrich). DNA solutions (0.167 mg mL^{-1} in (N-morpholino)propanesulfonic acid, MOPS, 10 mM) were prepared. These samples were deposited on a freshly cleaned silicon oxide surface for AFM, and on a freshly cleaned and glow discharged carbon grid for TEM. The silicon wafer (100 orientation and thickness 0.52 mm) was cut into pieces by a wafer dicing saw (Disco DAD321). The pieces were thoroughly cleaned by sonication in an ultrasonicator (VWR Ultrasonic Cleaner) for 5 min in a sequence of four solvents: isopropanol (LC-MS chromasolv®, Sigma-Aldrich), acetone (ACS reagent > 99.5 %, Sigma-Aldrich), ethanol (absolute, Sigma-Aldrich) and water (18 M *Ohm* cm, < 5 ppb total organic content,

Millipore). Afterwards, the wafer was freshly cleaned with an oxygen plasma (Femto plasma system, Diener).

A droplet of DNA solution was deposited on the substrate (2 µL for carbon grid and 5 µL for silicon wafer), softly blown with a nitrogen stream (focused to the solid-liquid air line) and dragged along until it evaporated. Then, a droplet between 2-5 µL of a 10 µM Pt (II) compound solution in 10 mM MOPS was deposited on the DNA/carbon grid or DNA/silicon sample. The Pt(II) incubation time varied between 2 and 10 min. The samples were dried with a nitrogen stream. DNA surface topographies were imaged with an atomic force microscopy (Agilent AFM 5500) in air, in AC mode with an oscillation frequency of 63 kHz. The images were obtained at 512 points per lines at 0.5-1 lines/second. Topography images were flattened, and profiles were analyzed with Gwyddion 2.55 (www.gwyddion.net). For TEM analysis, DNA was imaged by high-angle annular dark-field scanning transmission electron microscopy (HAADF-STEM), with a TECNAI G2 20 TWIN apparatus, equipped with a LaB6 filament, operated at 120 kV.

pUC18 plasmid mobility

The pUC18 plasmid (Thermo Fisher, ref. SD0051) was obtained from a transfected *E. coli* clone and purified using Miniprep (Qiagen). A total of 300 ng of DNA was incubated in 25 µL of 10 mM Tris pH 8.0 with 50 µM of the drug at 37°C for 1 hour. Subsequently, agarose gel electrophoresis was performed using a 1% agarose gel containing SYBR™ Safe DNA Gel Stain (Thermo Fisher, ref. S33111) in TAE buffer (40 mM Tris-HCl, 1 mM EDTA, 20 mM acetic acid, pH 8.0) at 80 V for 1 hour.

Comet assay

Comet slides (Abcam) were prepared using the following procedure. Initially, comet agarose (Abcam) was heated in a water bath at 90-95ºC until liquefied, and then cooled down to 37ºC for 20 minutes. Subsequently, 75 µL of comet agarose was added per well onto the 3-well comet slide

to create a base layer and incubated at 4ºC for 15 minutes. Cells, whether incubated with vehicle solution, CisPt, or Aurkines at 10 μM, were then trypsinized, collected, and centrifuged at 700 *g* for 2 minutes. The supernatant was then discarded, and cells were resuspended to $1x10^5$ cells/mL in ice-cold PBS. Cell samples were then mixed with comet agarose (1/10 ratio) and 75 μL of this mixture was added per well onto the comet agarose base layer. After a 15-minute incubation at 4ºC, the slide was first placed in Lysis Buffer for 45 minutes at 4ºC in the dark, and then in Alkaline Solution for 30 minutes at 4ºC in the dark. The slide was then placed within an electrophoresis chamber with cold alkaline electrophoresis solution, at 300 mA and 1 volt/cm for 30 minutes. After the electrophoresis step, the slide was rinsed in dH_2O three times, treated with cold 70% ethanol for 5 minutes, and air-dried. Finally, the slide was stained with Vista Green DNA Dye for 15 minutes at room temperature. The stained slide was then observed using a confocal microscope, specifically the ZEISS LSM 900 model.

Sample preparation for phosphoproteomic analysis

Approximately 500 μg of protein per sample was processed using the FASP protocol described in the previous section. Phosphopeptides were enriched using the High-Select™ TiO_2 Phosphopeptide Enrichment Kit (Thermo) according to the manufacturer's instructions. The enriched samples were then directly loaded onto the mass spectrometer for analysis.

Mass spectrometry-based high throughput phosphoproteomic analysis

Samples were analyzed using a timsTOF Pro mass spectrometer with PASEF (Bruker Daltonics), coupled online to an Evosep ONE liquid chromatograph (Evosep). Samples were directly loaded onto the Evosep ONE in a solution containing approximately 5% trifluoroacetic acid (TFA) and resolved using the 30 samples-per-day protocol (44-minute runs).

Total protein identification and quantification were performed using DIA-NN software v1.8.1.[15] Database searches were conducted against *Homo sapiens* entries from

UniProt/SwissProt, with precursor and fragment mass tolerances of 20 ppm and 0.05 Da, respectively. Carbamidomethylation of cysteines was set as a fixed modification, while oxidation of methionines and phosphorylation of serine, threonine and tyrosine were considered variable modifications. A false discovery rate (FDR) of <1% at the peptide level was applied as a significance threshold. Proteins not detected with at least two peptides and in at least 75% of samples within at least one experimental group were excluded from further analysis. Protein abundances were $\log_2$-transformed, and missing values were imputed using quantile regression-based imputation (QRILC). The imputed protein quantification data were normalized by quantile normalization in R studio (R version 4.2.1). Group comparisons were conducted by first assessing normality using the Shapiro-Wilk test. If data followed a normal distribution, a *t*-test was performed; otherwise, the Wilcoxon rank-sum test was used. P-values were adjusted for multiple testing using the FDR method.

Phosphopeptide samples were analyzed using MSFragger v4.1 and IonQuant v1.10.27 via FragPipe v22.0,[16] with default settings optimized for phosphoproteomic analysis. Phosphosites not detected in at least 75% of samples within at least one experimental group were excluded. Phosphosite abundances were $\log_2$-transformed, and missing values were imputed using QRILC. Normalization was performed by quantile normalization in R Studio (R version 4.2.1). Phosphorylation site expression levels were compared following the same statistical workflow as total protein analysis: normality was assessed using the Shapiro-Wilk test, followed by a *t*-test for normally distributed data or a Wilcoxon rank-sum test otherwise. P-values were adjusted for multiple testing using the FDR method. Phospho log ratios from comparisons, along with adjusted p-values for all identified phosphosites, were analyzed using QIAGEN Ingenuity Pathway Analysis (QIAGEN IPA).[17]

The mass spectrometry data have been deposited in the ProteomeXchange Consortium via the PRIDE (PRoteomics IDEntifications Database) repository[18] under the identifier PXD061935.

Experimental overexpression of human transporters in cells

To conduct transport assays, the CHO cell line and a human liver cancer cell line (HepG2) were employed. To establish stable monoclonal cell lines expressing human drug transporters, lentiviral transduction was carried out for both CHO and HepG2 cell lines, and cells were subjected to monoclonal selection. Specifically, CHO cells were transduced to express OATP1A2, OATP2B1, OATP1B3, and OCT3, while HepG2 cells were transduced to express OCT1. Single cell clones were obtained by the limited dilution method. As controls, CHO-mock and HepG2-mock cells were utilized.

Indirect transport assay

Cells were plated in a 12-well plate at a density of $2x10^4$ cells per well and cultured for four days. After this period, the cells were trypsinized and suspended first in culture medium and then in PBS. Subsequently, 50 µL of cells were combined with 50 µL of a "transport" medium (containing 96 mM NaCl, 5.3 mM KCl, 1.1 mM KH_2PO_4, 0.8 mM $MgSO_4$, 1.8 mM $CaCl_2$, 11 mM glucose, and 50 mM HEPES at pH 7.40). Within this medium, a specific known fluorescent substrate at a concentration of 1 µM was introduced along with the corresponding specific inhibitor or the Aurkine compound at 10 µM, and the mixture was incubated at 37ºC for 15 minutes (Table S3). The loading phase was concluded by diluting the mixture with 900 µL of ice-cold uptake medium, and the level of intracellular fluorescence was assessed using a FACSCalibur flow cytometer. Propidium iodide staining (5 µg/mL) was applied to identify and exclude dead cells from the data analysis.

Direct transport assay

Cells were initially seeded into 12-well plates at a density of $2x10^4$ cells per well, and the experiments were conducted the following day. The culture medium was replaced with fresh medium containing either Aurkines 16 or 18. After a 60-minute incubation period, the uptake process was halted by washing the plates four times with 1 mL of ice-cold culture medium devoid of FBS. Subsequently, cells were lysed using distilled water supplemented with 5 µM prednisolone (utilized as an internal standard). The concentration of Aurkines in the lysates was quantified using HPLC-MS/MS on a 6420 Triple Quad LC/MS system from Agilent Technologies, Santa Clara, CA, USA. The results were adjusted based on the protein content.

Experimental animal models of CCA

The therapeutic efficacy of Aurkines 16 and 18 was assessed *in vivo*, in subcutaneous xenograft CCA mouse models. All experimental protocols received approval from the Ethical Committee for Animal Experimentation of the BHRI (CEEA18/020, CEEA21/011 and CEEA24/08) and were conducted in accordance with the institution's regulations for the ethical treatment of laboratory animals.

<u>Xenograft CCA models</u>

CCA (EGI-1) cells (1×10^6 cells) and CCA (EGI-1R) cells resistant to CisPt ($5x10^6$)were subcutaneously injected in the dorsal flanks of seven-week-old male immunodeficient CD-1 nude mice (Crl:CD1-Foxn1nu; strain 086, homozygous) (Charles River). Once tumors were well-established (average size: ~50 mm3), mice were homogenously distributed into control and treatment groups (CisPt, Aurkine 16 and Aurkine 18). All of the treatments were intraperitoneally administered once a week during 5 weeks, at 2 mg/kg or 0.5 mg/kg dose. Tumor size were measured using a caliper every 2-3 days during 5 weeks. The formula V = (D x d2)/2 was

employed to calculate tumor volume (V), where "D" means the largest diameter measured and "d" represents the shortest diameter.

Orthotopic CCA model

Orthotopic xenografts were established by injecting 5 × 10^5 CCA (SB) mouse cells, resuspended in 50% Matrigel/saline, into the left lobule of the liver of immunocompetent C57BL6/J mice following laparotomy, as previously described.[19] One week later, mice were randomized into control and treatment groups (CisPt or Aurkine 16). Treatments were administered intraperitoneally (0.5 mg/kg) once per week for one month. After 28 days, mice were sacrificed by exanguination. After sacrifice, tumor size was measured with a caliper and tumor volume (V) was calculated using the following formula: $V = (D \times d^2)/2$ (where "D" represents the largest diameter measured and "d" the shortest".

Toxicological study

Healthy C57BL/6J mice were treated once per week for one month with either vehicle (DMF), CisPt, Aurkine 16, or Aurkine 18 at doses of 0.5 mg/kg (used in the subcutaneous model with CisPt-sensitive CCA cells) and 2 mg/kg (used in the subcutaneous model with CisPt-resistant CCA cells).

A comprehensive panel of hematological markers was measured, including:

- Red blood cell parameters: erythrocytes, hemoglobin, hematocrit, mean corpuscular volume (MCV), mean corpuscular hemoglobin (MCH), mean corpuscular hemoglobin concentration (MCHC), and red cell distribution width (RDW).
- Platelet parameters: platelet count and immature platelet fraction (IPF).

- White blood cell parameters: total white blood cells (WBCs), neutrophils, lymphocytes, monocytes, eosinophils, basophils, immature granulocytes, nucleated red blood cells (NRBCs), reticulocytes, and immature reticulocyte fraction (IRF).

Additionally, to further assess potential nephrotoxicity or hepatotoxicity, several biochemical parameters were measured in serum, including alanine aminotransferase (ALT), aspartate aminotransferase (AST), gamma-glutamyl transferase (GGT), alkaline phosphatase (ALP), creatinine, urea, albumin, glucose, triglycerides, and bilirubin.

Statistical analysis

Statistical analyses were performed using the GraphPad Prism 9.2.0 software (GraphPad Software). Once the normality assessment with Shapiro-Wilk test was carried out, the statistical difference between two data sets was determined using the parametric paired or unpaired Student´s *t*-test or the non-parametric Mann-Whitney test. For comparison between more than two data sets, one-way analysis of variance (ANOVA) with Tukey´s *post hoc* test or Kruskal-Wallis with Dunn´s *post hoc* test was implemented for the analysis of normally and non-normally distributed data, respectively. Data are indicated as mean ± standard error of the mean (SEM), and differences of $p < 0.05$ were considered statistically significant.

Supplementary figures

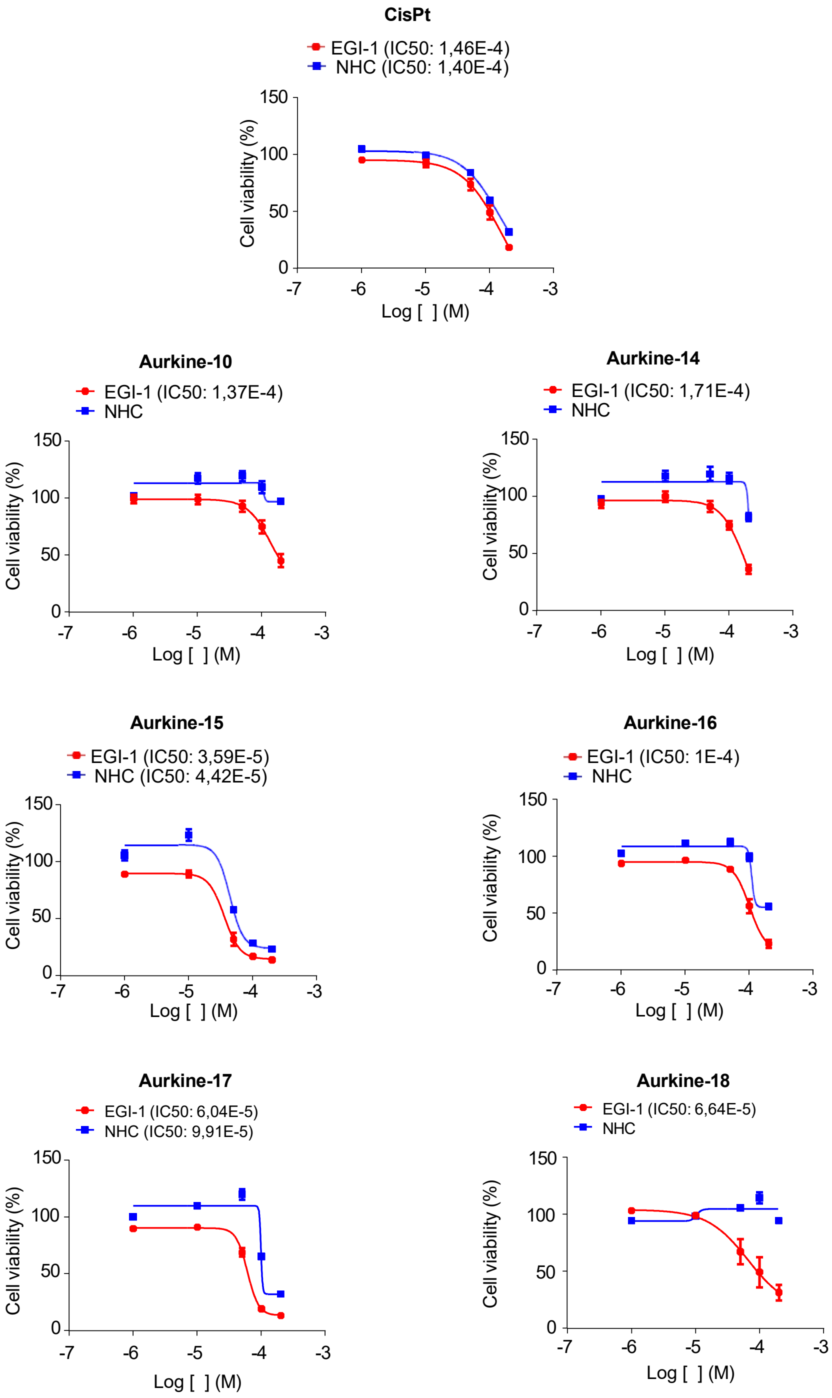

Fig. S1. Dose-response curves and IC50 values of Aurkines on eCCA (EGI- 1) cells and NHCs. Dose-response curves (0.1 μM, 1 μM, 5 μM, 10 μM, 20 μM) and IC50 values of the Aurkine compounds were determined for the eCCA cell line (EGI-1) and NHCs. Curve fitting and IC50 calculation were performed using the log(inhibitor) vs. normalized response with variable slope equation in GraphPad 9.2.0. Abbreviations: CisPt, cisplatin; NHCs, normal human cholangiocytes.

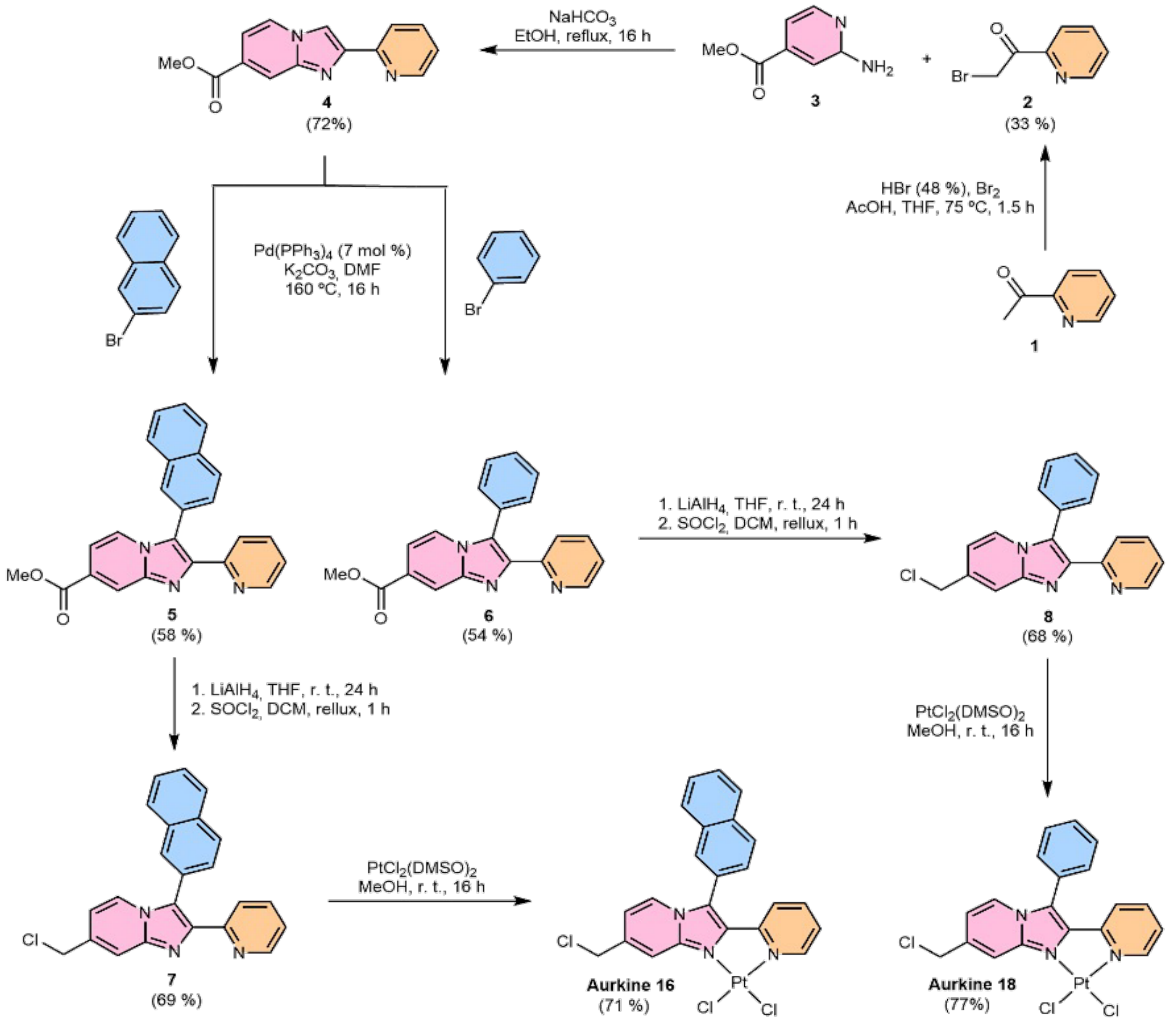

Fig. S2. Chemical synthesis of Aurkines 16 and 18. THF: tetrahydrofuran; DMSO: dimethylsulfoxide; AcOH: acetic acid; EtOH: ethanol.

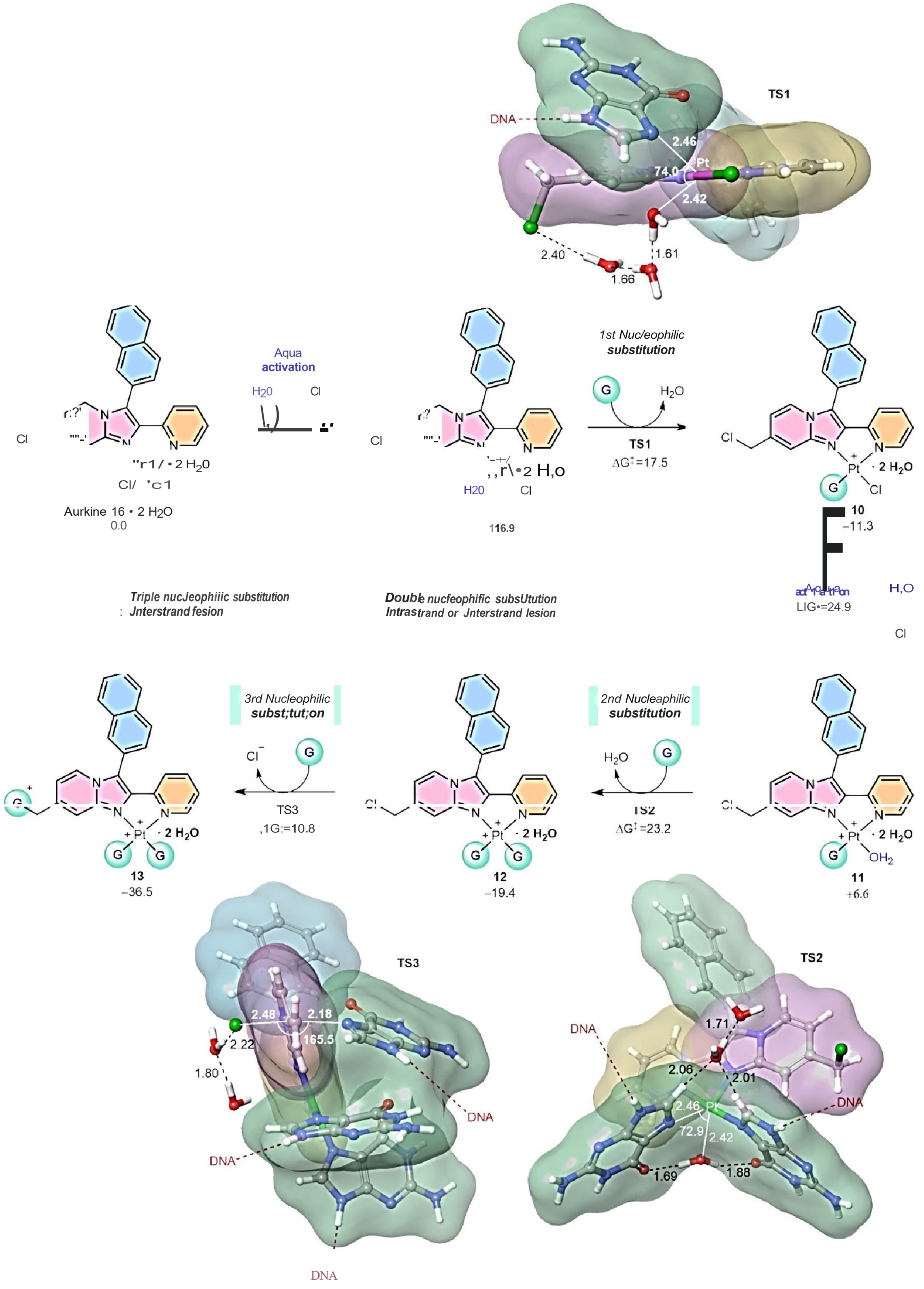

TS1
DNA
2.46
Pt
74.0
2.42
2.40
1.61
1.66
Aqua
activation
H20
Cl
Cl
Aurkine 16 • 2 H2O
0.0
116.9
1st Nuc/eophilic
substitution
G
H2O
TS1
ΔG‡=17.5
Cl
N
N
N
Pt
· 2 H2O
G
Cl
10
–11.3
H,O
LIG•=24.9
Cl
Triple nucJeophiiic substitution
Jnterstrand fesion
Double nucfeophific subsUtution
Intrastrand or Jnterstrand lesion
3rd Nucleophilic
subst;tut;on
Cl−
G
TS3
,1G:=10.8
2nd Nucleaphilic
substitution
H2O
G
TS2
ΔG‡=23.2
· 2 H2O
OH2
13
–36.5
12
–19.4
11
+6.6
TS3
2.48
2.18
165.5
2.22
1.80
DNA
DNA
DNA
TS2
DNA
1.71
2.06
2.01
2.46
Pt
72.9
2.42
DNA
1.69
1.88

Fig. S3. DFT analysis of Aurkine 16 activation and electrophilicity with DNA. All calculations were performed at the B3LYP/D3-BJ(PCM=water)/6-31+G(d,p)&LANL2DZ(Pt) level of theory**.** The figure displays the formation of aqua intermediates and substitution adducts with up to three guanine molecules, with either none or two water molecules. The numbers beneath the intermediates indicate the relative Gibbs free energies, while those under the reaction arrows denote activation energies ($\Delta G^{\ddagger}$) in kcal/mol calculated at 298 K. Transition structures (TSs) illustrate to the nucleophilic substitution reactions by guanine units, with bond distances and angles in Å and degrees, respectively.

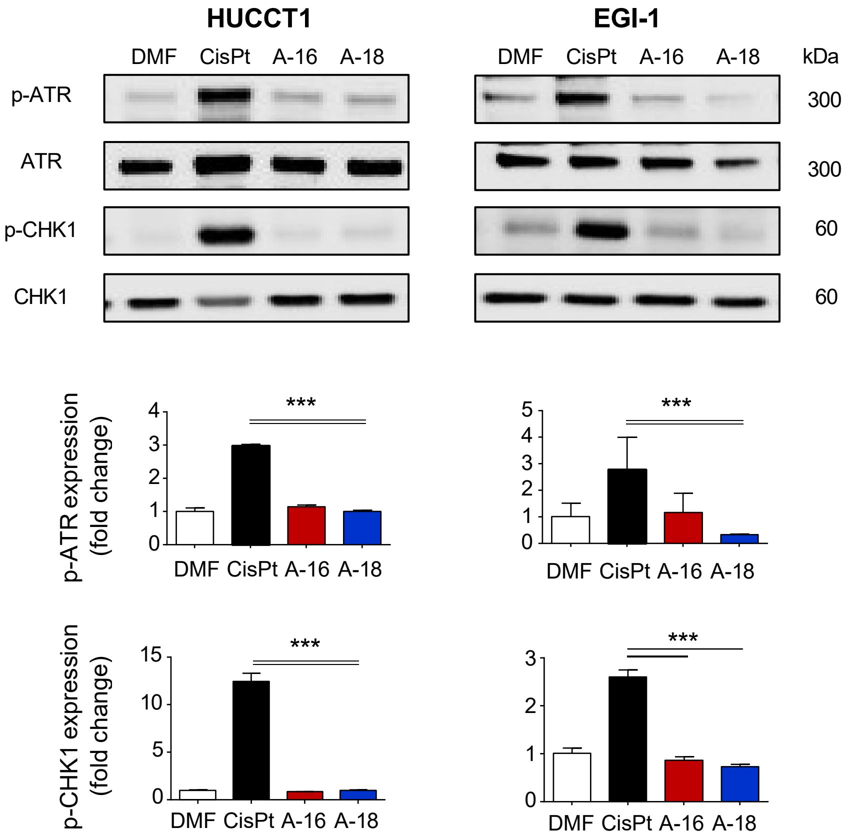

Fig. S4. Single-strand DNA repair pathway activation. Immunoblots and quantification of single-strand DNA repair proteins after 48-hour incubation with vehicle, CisPt, Aurkine 16 or 18 in CCA cell lines (HUCCT1 and EGI-1). β-actin served as a loading control. Student's t-test was used. Data are shown as mean ± SEM. p-values: * ($p \leq 0.05$), ** ($p \leq 0.01$),
*** ($p \leq 0.001$). Abbreviations: ATR, ataxia telangiectasia and RAD3-related protein; CisPt, Cisplatin; CHK1, checkpoint kinase 1; CCA, cholangiocarcinoma; DMF, dimethylformamide.

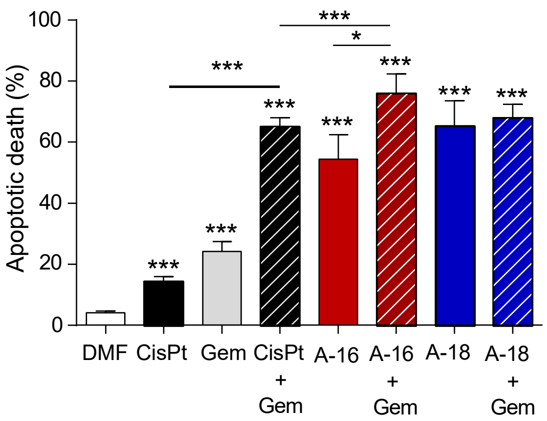

Fig. S5. Antitumor effect of Aurkines combined with Gemcitabine on EGI-1 CCA cells. Annexin V/TO-PRO[TM]-3 dual staining of EGI-1 CCA cells after 48-hour incubation with CisPt, Aurkines 16 or 18 (10μM) alone or in combination with Gemcitabine (1 μM). Student's t-test was used. Data are shown as mean ± SEM. p-values: * (p ≤ 0.05), ** (p ≤ 0.01), *** (p ≤ 0.001). Abbreviations: CCA, cholangiocarcinoma; CisPt, Cisplatin; DMF, dimethylformamide; Gem, gemcitabine; NHC.

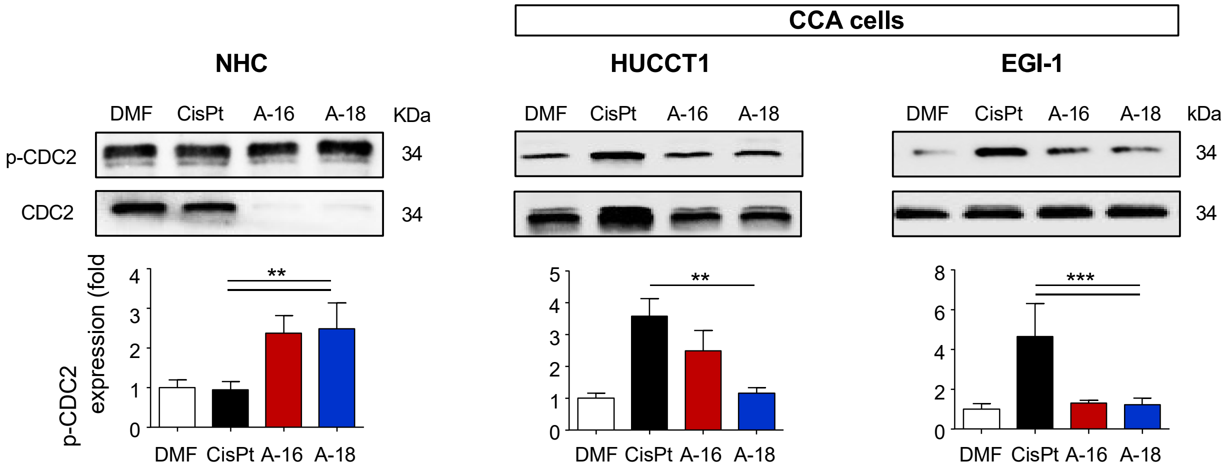

Fig. S6. p-CDC2 quantification. Immunoblot and quantification of p-CDC2 in NHC and CCA cell lines (HUCCT1 and EGI-1) following treatment with vehicle, CisPt, Aurkines 16 or 18 (10 μM), with β-actin as loading control. Student's t-test was used. Data are shown as mean ± SEM. p-values: * (p ≤ 0.05), ** (p ≤ 0.01), *** (p ≤ 0.001). Abbreviations: CisPt, Cisplatin; CCA, cholangiocarcinoma; DMF, dimethylformamide; NHC, normal human cholangiocytes.

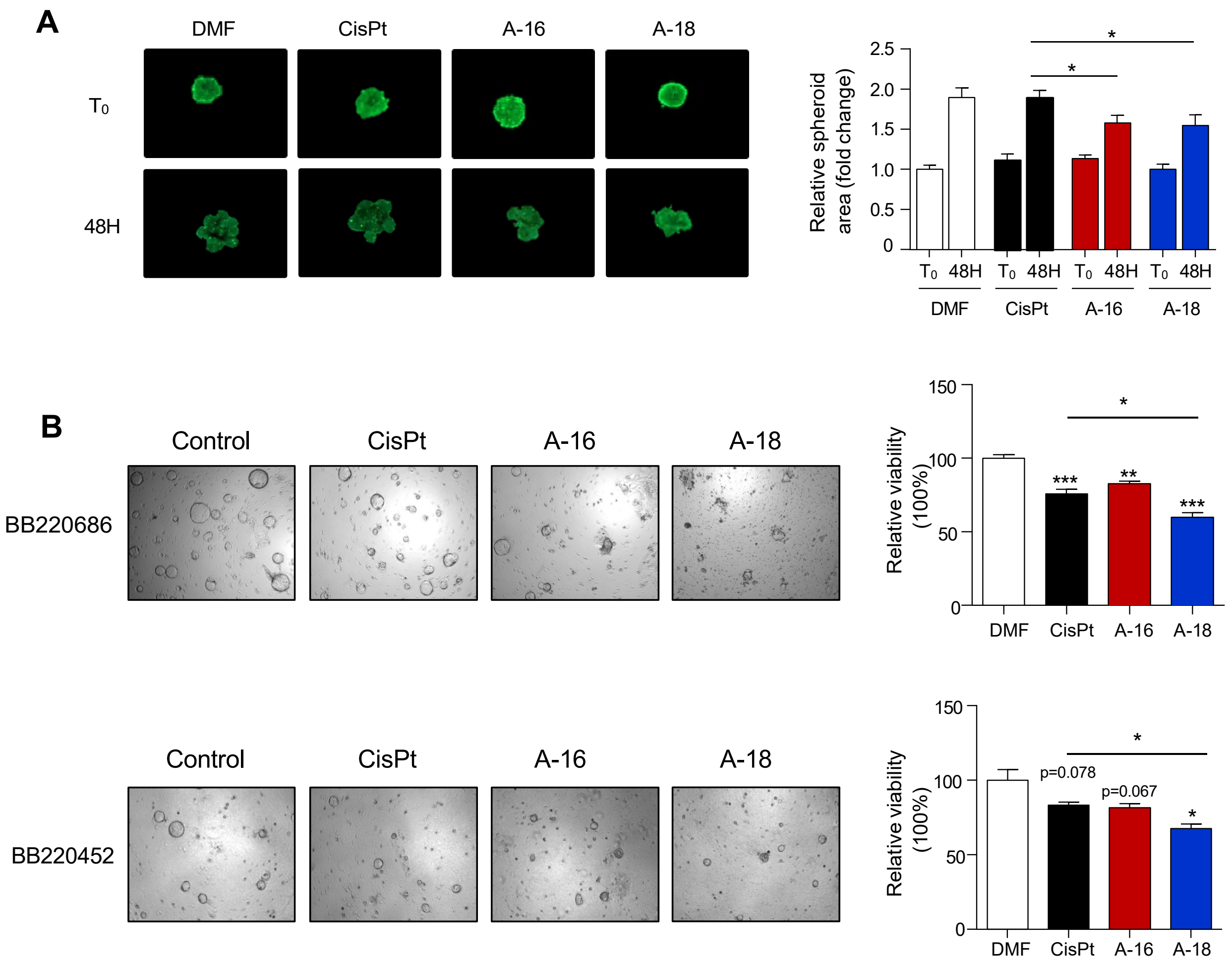

Fig. S7. Antitumoral effects of Aurkines on 3D models. (A) 3D spheroid formation of EGI-1 CCA cells after 48-hour incubation with vehicle, CisPt, Aurkines 16 or 18 (10 μM). (B) Cell viability of patient-derived organoids (PDOs) of two CCA patients after 72-hour treatment with CisPt, Aurkine 16 or Aurkine 18 (20 μM). Student's t-test was used. Data are shown as mean ± SEM. p-values: * ($p \leq 0.05$), ** ($p \leq 0.01$), *** ($p \leq 0.001$). Abbreviations: CisPt, Cisplatin; CCA, cholangiocarcinoma; DMF, dimethylformamide; PDO, patient-derived organoids.

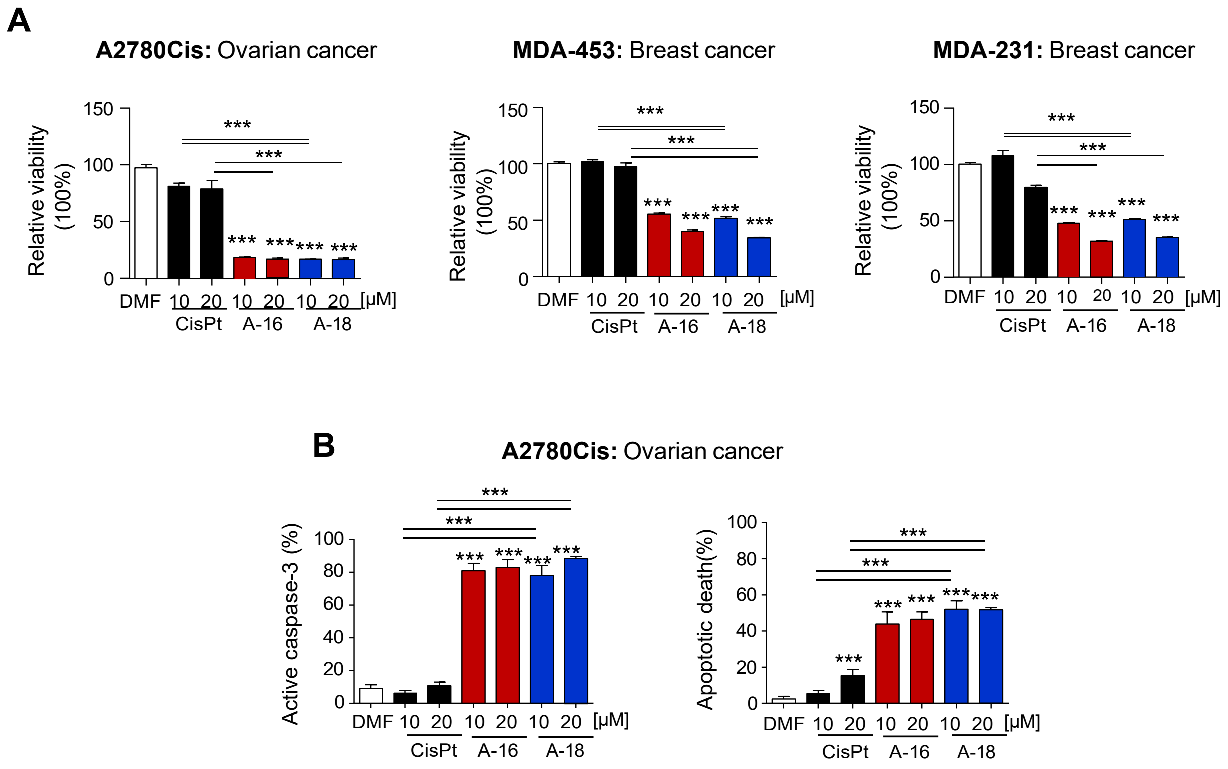

Fig. S8. Antitumor effect of Aurkines 16 and 18 on CisPt-resistant human cancer cell lines. (A) Cell viability of the ovarian cancer cell line (A2780Cis) and the breast cancer cell lines (MDA-453 and MDA-231) after 48-hour incubation with vehicle (DMF), CisPt, Aurkines 16 or 18 (10 μM and 20 μM). (B) % of cleaved caspase-3$^+$ cells and Annexin V/ TO-PRO^{TM}-3 dual staining of A2780Cis cells after 48-hour incubation with vehicle, CisPt, Aurkine 16 or 18 (10 μM and 20 μM). Student's t-test was used. Data are shown as mean ± SEM. p-values: * ($p \leq 0.05$), ** ($p \leq 0.01$), *** ($p \leq 0.001$). Abbreviations: CisPt, cisplatin; DMF, dimethylformamide.

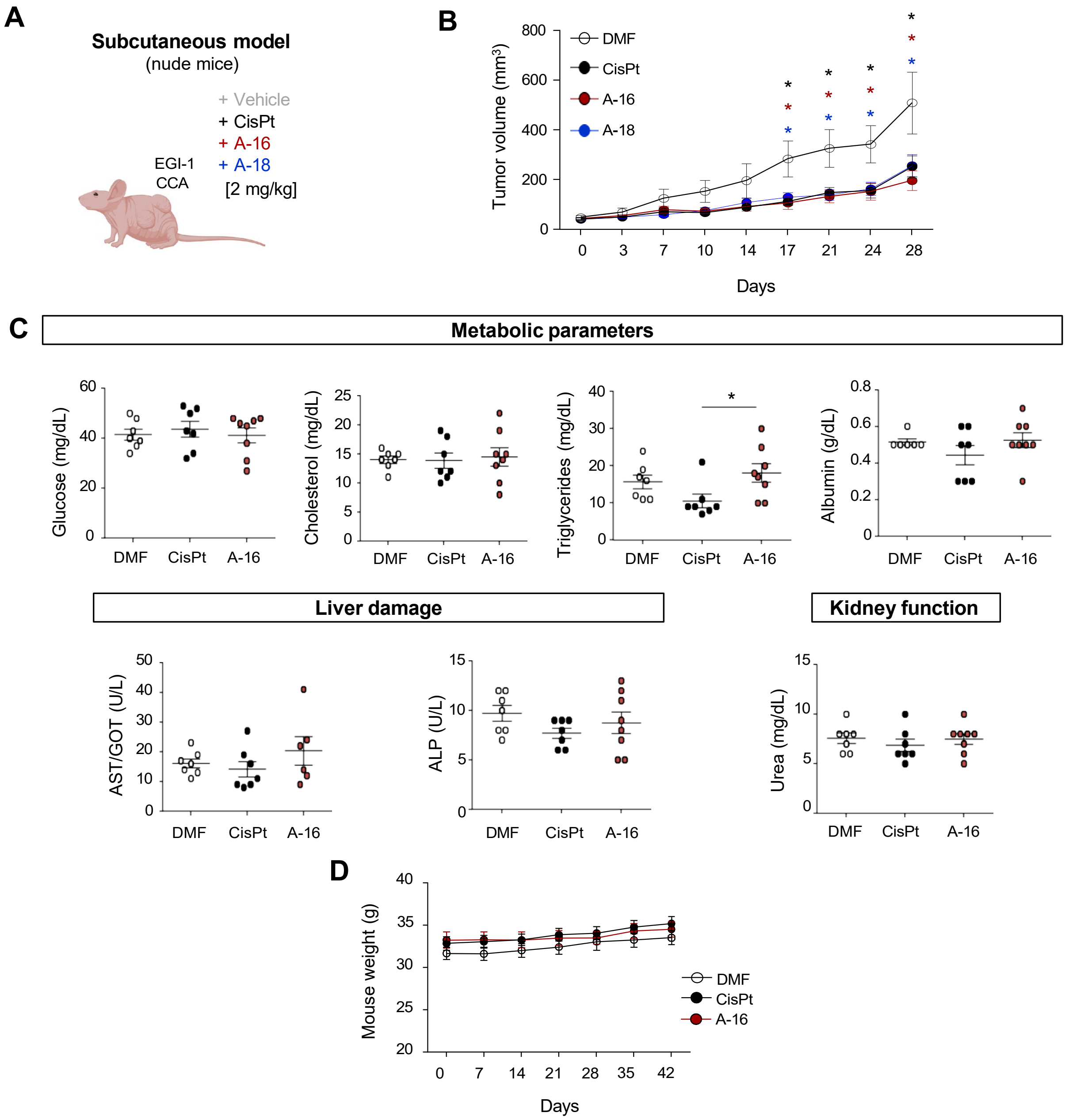

Fig. S9. *In vivo* antitumor activity of Aurkines 16 and 18 on CCA. (A) Schematic representation of the subcutaneous CCA model. (B) Tumor volume growth during treatment with CisPt, Aurkine 16 or Aurkine 18 (2 mg/kg). Group sizes: vehicle control (n = 8), CisPt (n = 8), Aurkine 16 (n = 8), and Aurkine 18 (n = 12). (C) Biochemical parameters in serum of mice treated with CisPt, Aurkine 16 or vehicle (DMF) (0.5 mg/kg). (D) Mouse weight over time. One-way ANOVA test or Student's t-tests were used. Data are shown as mean ± SEM. p-values: * ($p \leq 0.05$), ** ($p \leq 0.01$), *** ($p \leq 0.001$). Abbreviations: ALP, alkaline phosphatase; AST, aspartate aminotransferase; CisPt, cisplatin; DMF, dimethylformamide.

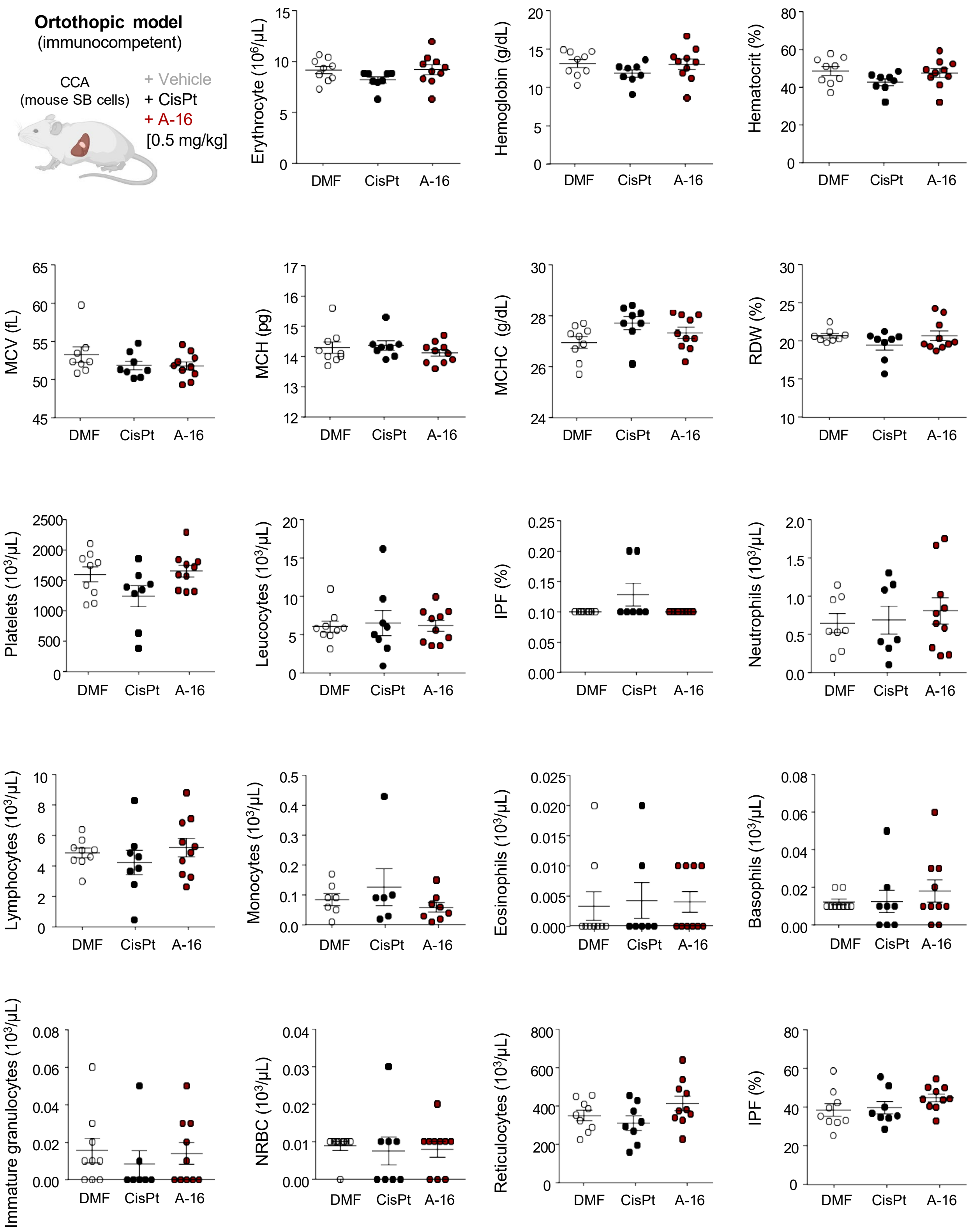
Orthotopic model
(immunocompetent)
CCA
(mouse SB cells)
+ Vehicle
+ CisPt
+ A-16
[0.5 mg/kg]
Erythrocyte (10^6/µL)
Hemoglobin (g/dL)
Hematocrit (%)
MCV (fL)
MCH (pg)
MCHC (g/dL)
RDW (%)
Platelets (10^3/µL)
Leucocytes (10^3/µL)
IPF (%)
Neutrophils (10^3/µL)
Lymphocytes (10^3/µL)
Monocytes (10^3/µL)
Eosinophils (10^3/µL)
Basophils (10^3/µL)
Immature granulocytes (10^3/µL)
NRBC (10^3/µL)
Reticulocytes (10^3/µL)
IPF (%)
DMF
CisPt
A-16

Fig. S10. Red and white blood cell parameters in the orthotopic CCA model after treatment with vehicle solution, CisPt or Aurkine 16. A comprehensive panel of hematological markers was evaluated in orthotopic xenografts after treatment with vehicle solution (n=9), CisPt (n=8) or Aurkine 16 (n=10) at a dose of 0.5 mg/kg, administered once per week for one month. Student's t-test were used. Data are shown as mean ± SEM. p-values: * ($p \leq 0.05$), ** ($p \leq 0.01$), *** ($p \leq 0.001$). Abbreviations: IPF: immature platelet fraction; IRF, immature reticulocyte fraction; MCH, mean corpuscular hemoglobin; MCHC, mean corpuscular hemoglobin concentration; MCV, mean corpuscular volume; NRBCs, nucleated red blood cells; RDW: red cell distribution width.

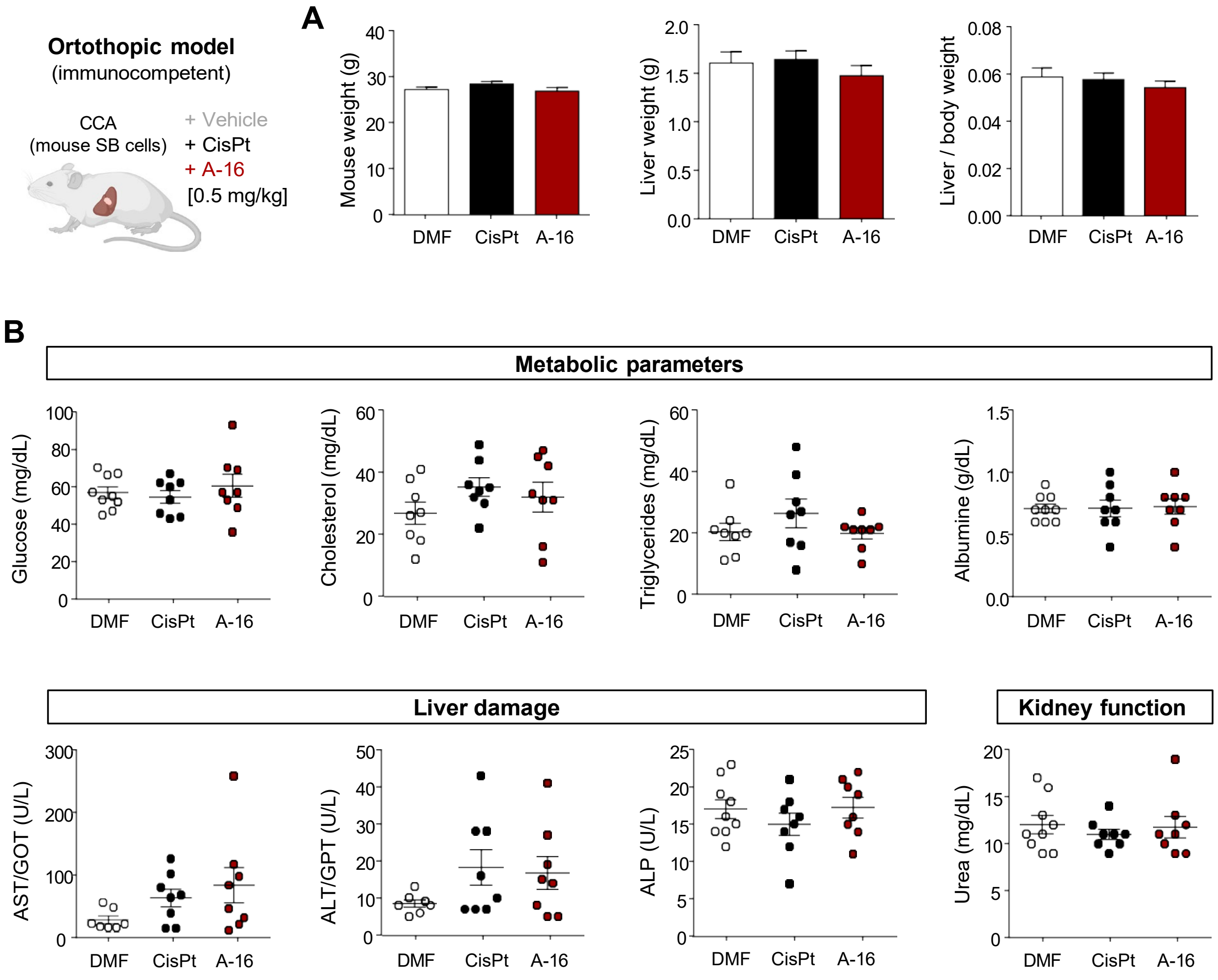

Fig. S11. Biochemical parameters in the orthotopic CCA model after treatment with vehicle solution, CisPt or Aurkine 16. (A) Mouse and liver weight at sacrifice.

(B) A comprehensive panel of biochemical markers was evaluated in orthotopic xenografts after treatment with vehicle solution (n=9), CisPt (n=8) or Aurkine 16 (n=10) at a dose of 0.5 mg/kg, administered once per week for one month. Student's t-test were used. Data are shown as mean ± SEM. p-values: * ($p \leq 0.05$), ** ($p \leq 0.01$), *** ($p \leq 0.001$). Abbreviations: ALP, alkaline phosphatase; ALT, alanine aminotransferase; AST, aspartate aminotransferase; GGT, gamma-glutamyl transferase.

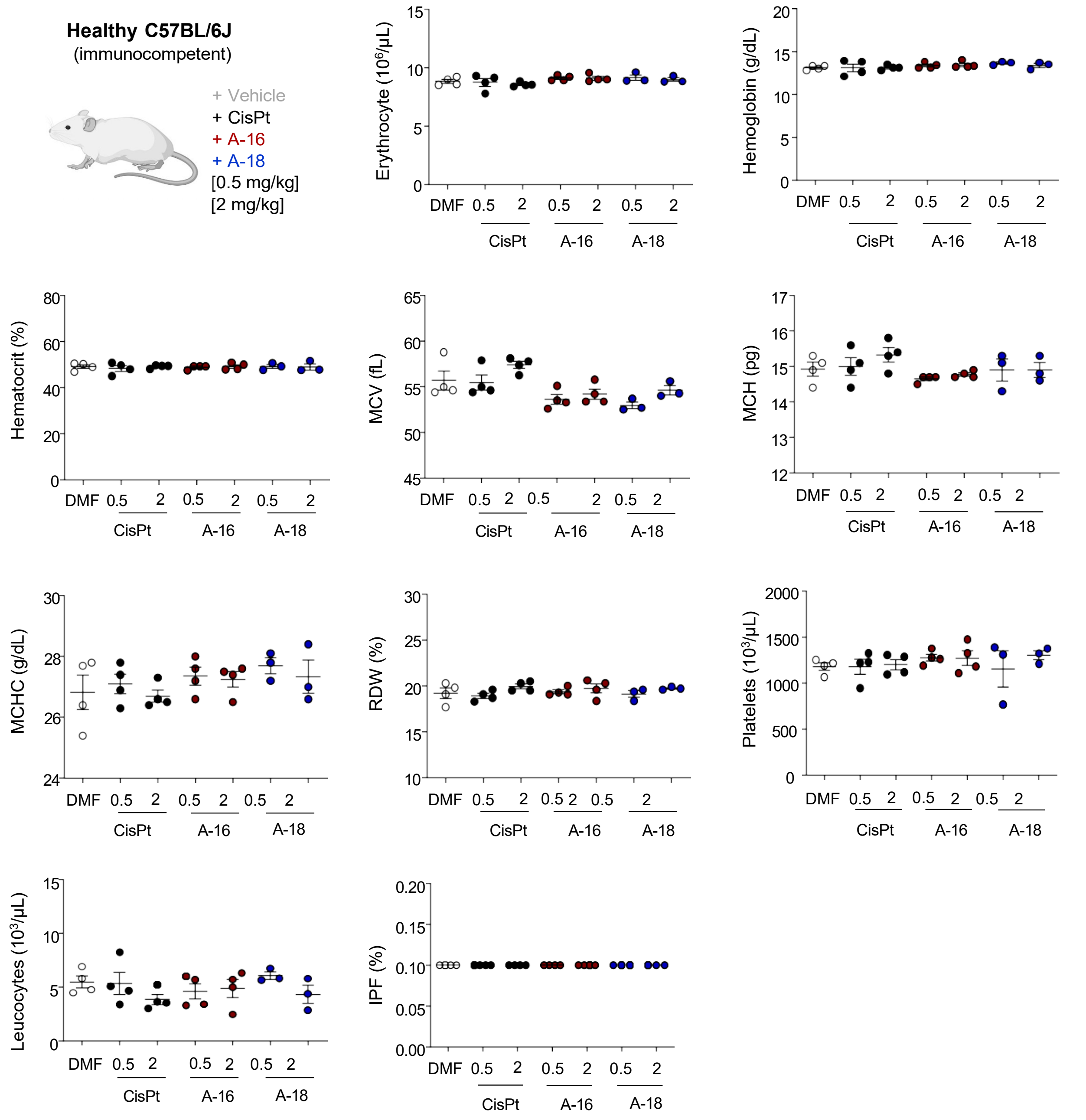

Fig. S12. Red blood cell parameters in healthy mice after treatment with CisPt, Aurkines or vehicle. Healthy C57BL/6J mice were treated once per week with either vehicle (DMF) (n=4), CisPt (n=4), Aurkine 16 (n=4), or Aurkine 18 (n=3) at doses of 0.5 mg/kg and 2 mg/kg for one month. A comprehensive panel of hematological markers was evaluated. Student's t-test were used. Data are shown as mean ± SEM. p-values: * ($p \leq 0.05$), ** ($p \leq 0.01$), *** ($p \leq 0.001$). Abbreviations: IPF: immature platelet fraction; MCH, mean corpuscular hemoglobin; MCHC, mean corpuscular hemoglobin concentration; MCV, mean corpuscular volume.

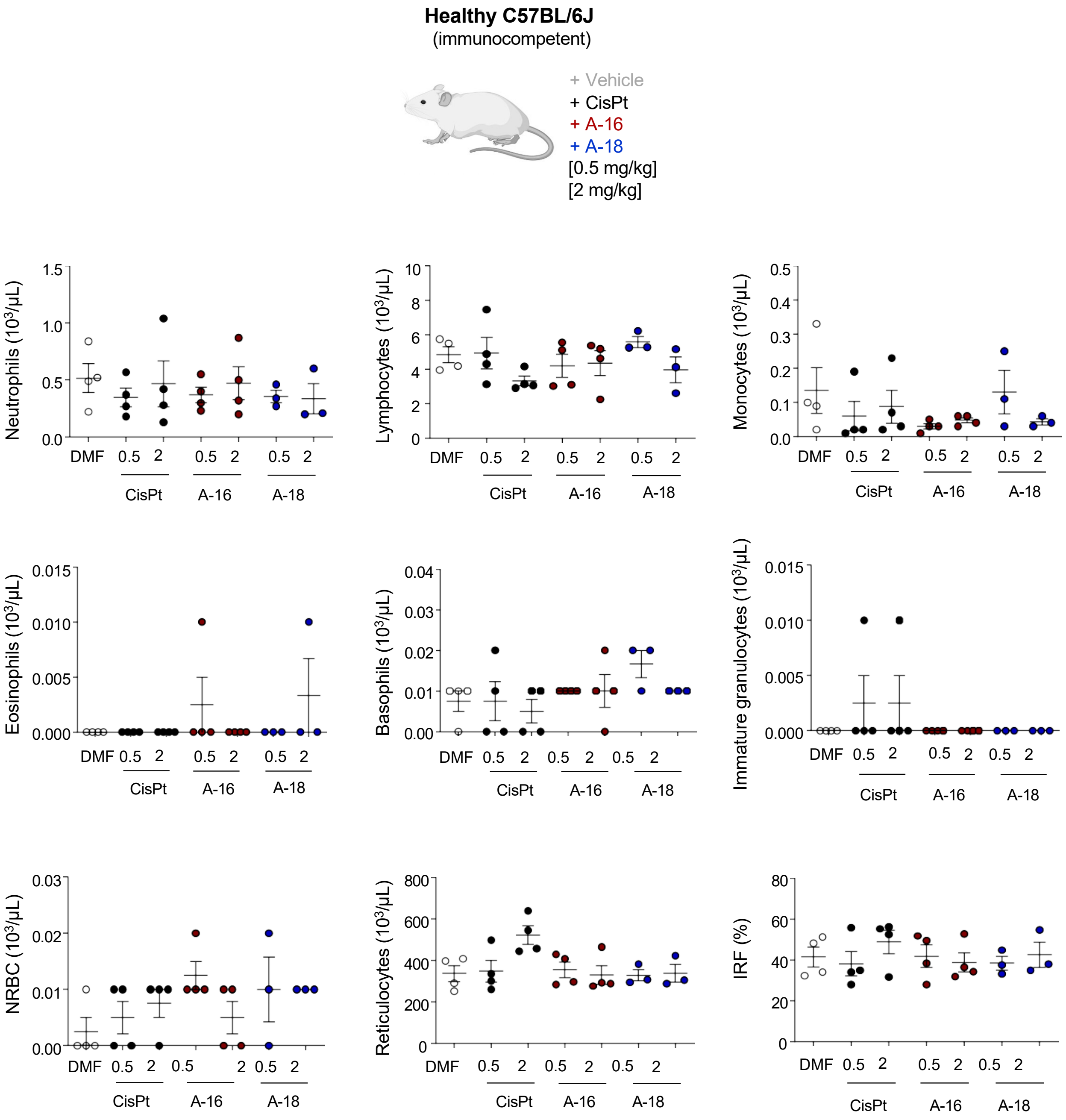

Fig. S13. White blood cell parameters in healthy mice after treatment with CisPt, Aurkines or vehicle. Healthy C57BL/6J mice were treated once per week with either vehicle (DMF) (n=4), CisPt (n=4), Aurkine 16 (n=4), or Aurkine 18 (n=3) at doses of 0.5 mg/kg and 2 mg/kg for one month. A comprehensive panel of hematological markers was evaluated. Student's t-test were used. Data are shown as mean ± SEM. p-values: * ($p \leq 0.05$), ** ($p \leq 0.01$), *** ($p \leq 0.001$). Abbreviations: Abbreviations: IRF, immature reticulocyte fraction; NRBCs, nucleated red blood cells.

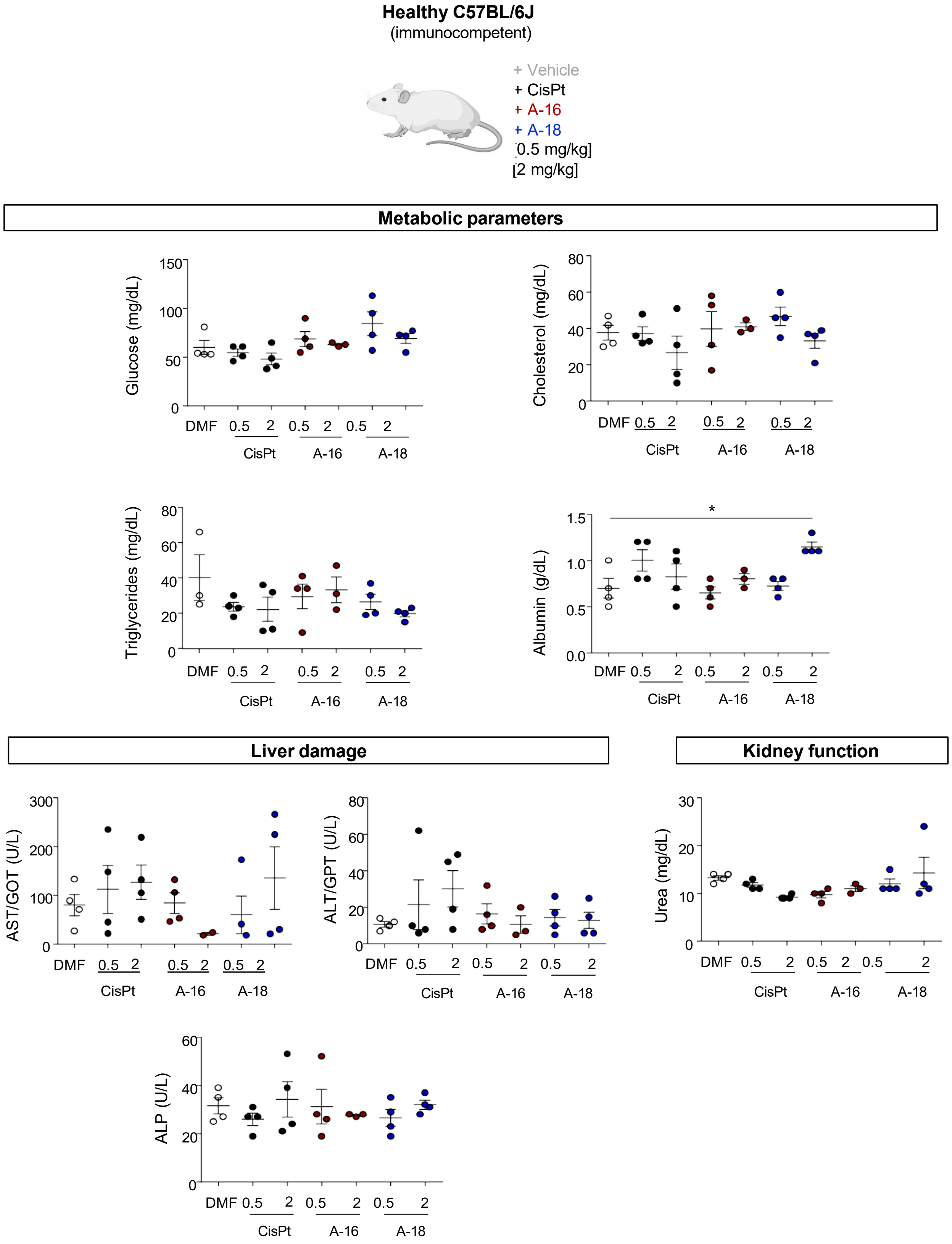
Healthy C57BL/6J
(immunocompetent)
+ Vehicle
+ CisPt
+ A-16
+ A-18
[0.5 mg/kg]
[2 mg/kg]
Metabolic parameters
Glucose (mg/dL)
Cholesterol (mg/dL)
Triglycerides (mg/dL)
Albumin (g/dL)
*
Liver damage
AST/GOT (U/L)
ALT/GPT (U/L)
ALP (U/L)
Kidney function
Urea (mg/dL)
DMF
0.5
2
CisPt
A-16
A-18

Fig. S14. Biochemical parameters in healthy mice after treatment with CisPt, Aurkines, or vehicle. Healthy C57BL/6J mice were treated once per week with either vehicle (DMF) (n=4), CisPt (n=4), Aurkine 16 (n=4), or Aurkine 18 (n=4) at doses of 0.5 mg/kg and 2 mg/kg for one month. A comprehensive panel of biochemical parameters was evaluated in serum. Student's t-test were used. Data are shown as mean ± SEM. p-values: * ($p \leq 0.05$), ** ($p \leq 0.01$), *** ($p \leq 0.001$). Abbreviations: ALP, alkaline phosphatase; ALT, alanine aminotransferase; AST, aspartate aminotransferase; GGT, gamma-glutamyl transferase.

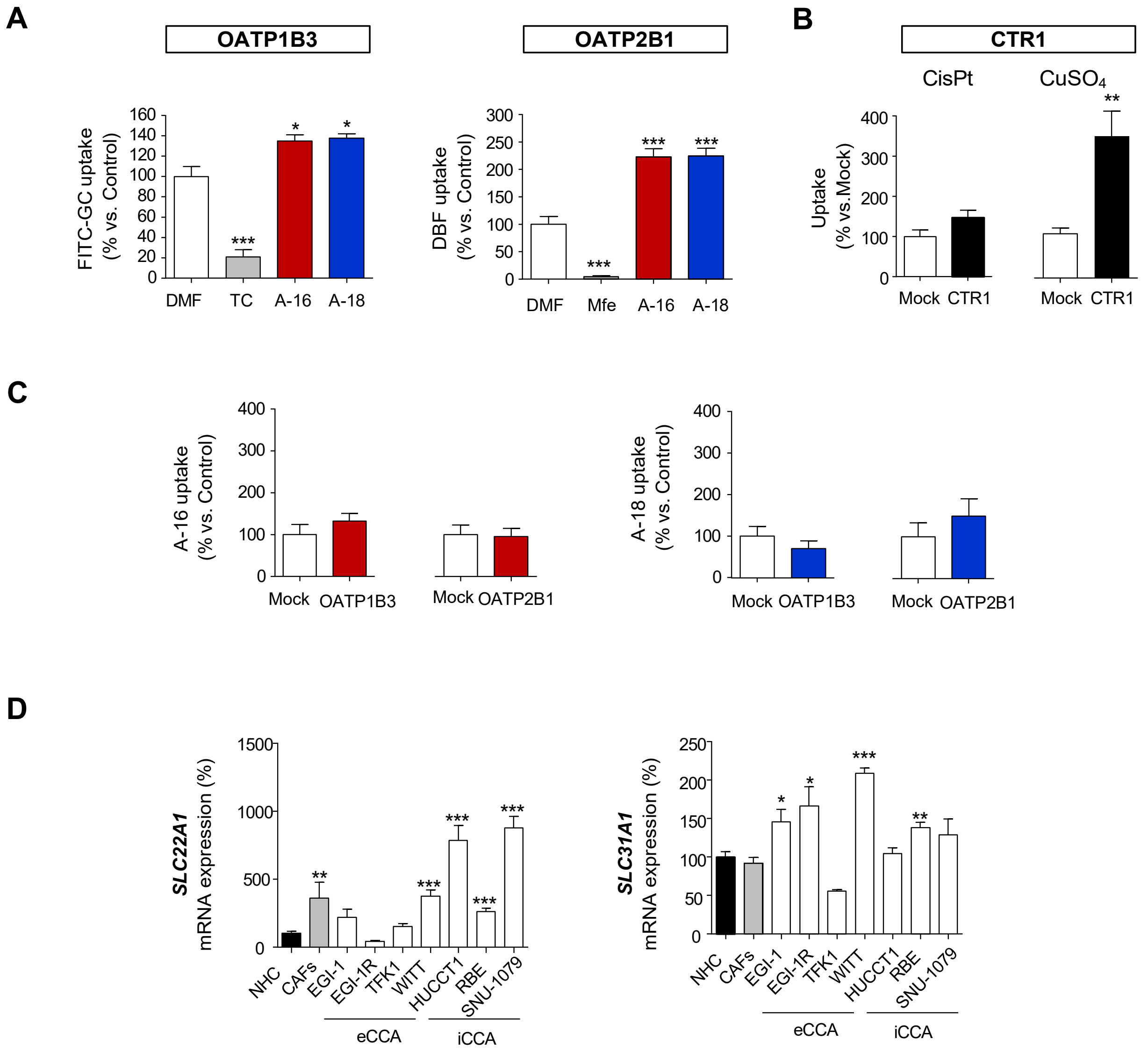

Fig. S15. Analysis of transporters involved in Aurkines 16 and 18 uptake by cancer cells. (A) Uptake of specific fluorescent substrates by cells with or without experimental overexpression of upstream transporters measured by flow cytometry. (B) Atomic absorption spectroscopy (AAS) was employed to determine the accumulation of Pt and Cu after incubating the cells with CisPt or CuSO4. (C) Intracellular accumulation of Aurkine 16 and 18 in cells with or without overexpression of different transporters measured by HPLC-MS/MS. (D) Relative mRNA expression (qPCR) of *SLC22A1* and *SLC31A1* in NHC, CAFs, eCCA (i.e., EGI-1, EGI-1R, TFK1, WITT) and iCCA (i.e., HUCCT1, RBE and SNU-1079) cells. Student's t-test was used. Data are shown as mean ± SEM. p-values: * (p ≤ 0.05), ** (p ≤ 0.01), *** (p ≤ 0.001). Abbreviations: CTR1, copper transport 1; Mfe, mifepristone; NHC, normal human cholangiocytes; OATP, organic-anion-transporting polypeptide; OCT, organic cation transporter; TC, taurocholate.

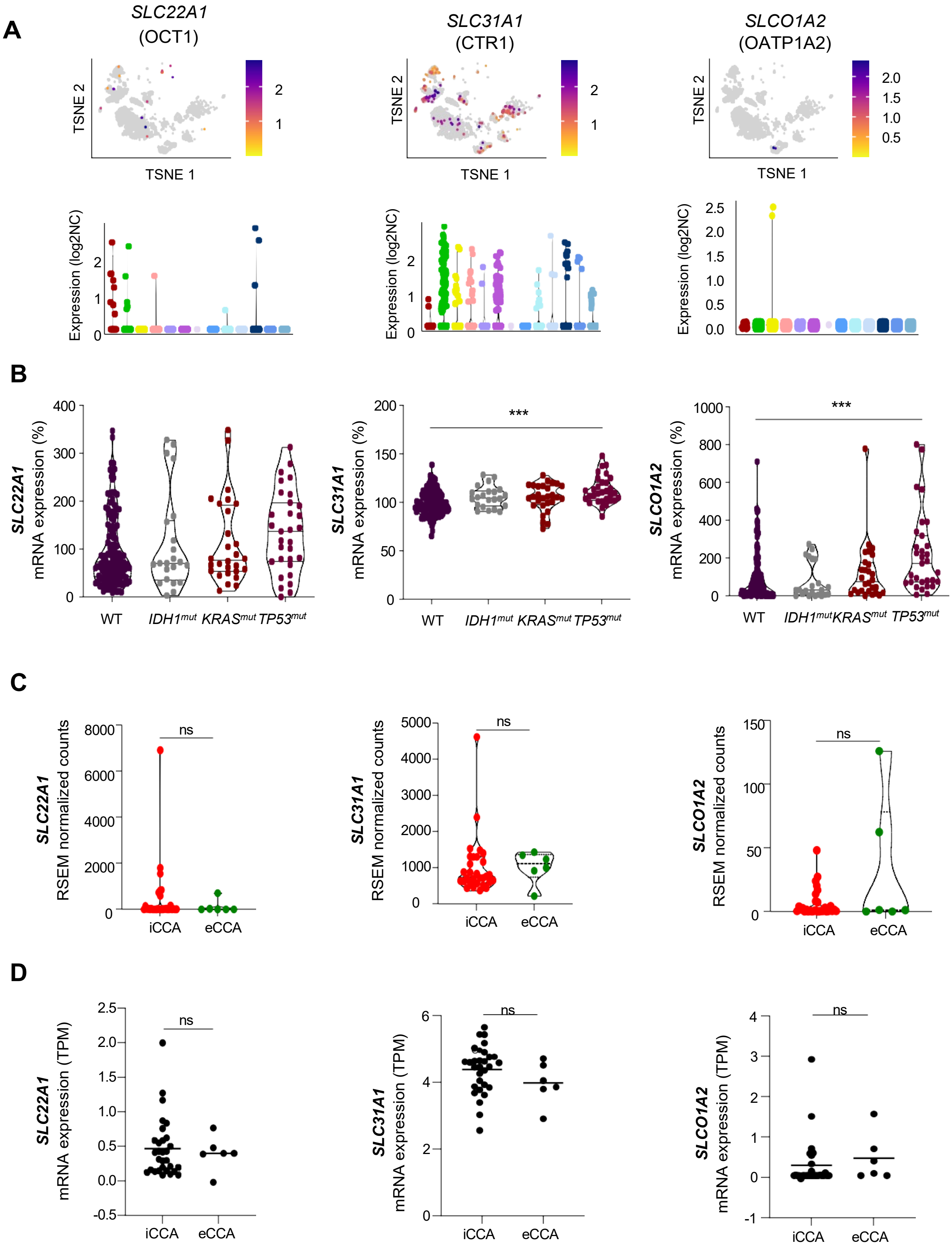
A
SLC22A1 (OCT1)
SLC31A1 (CTR1)
SLCO1A2 (OATP1A2)
TSNE 1
TSNE 2
Expression (log2NC)
B
SLC22A1 mRNA expression (%)
SLC31A1 mRNA expression (%)
SLCO1A2 mRNA expression (%)
WT
IDH1mut
KRASmut
TP53mut
***
C
SLC22A1 RSEM normalized counts
SLC31A1 RSEM normalized counts
SLCO1A2 RSEM normalized counts
ns
iCCA
eCCA
D
SLC22A1 mRNA expression (TPM)
SLC31A1 mRNA expression (TPM)
SLCO1A2 mRNA expression (TPM)

Fig. S16. Expression levels of *SLC22A1, SLC31A1 and SLCO1A2*. (A) Expression levels of *SLC22A1, SLC31A1* and *SLCO1A2* across all the cell populations of human CCA tumors detected by scRNA-seq in the GSE151530 dataset, which includes samples from 12 CCA patients. (B) Expression of *SLC22A1, SLC31A1* and *SLCO1A2* in CCA tissues, stratified by mutational status (*IDH1, KRAS, TP53* or wild type type for these genetic alterations). (C) mRNA expression of *SLC22A1, SLC31A1* and *SLCO1A2* in CCA tissues, stratified by anatomical origin (iCCA vs eCCA). (D) mRNA expression of *SLC22A1, SLC31A1* and *SLCO1A2* in CCA cell lines, stratified by anatomical origin (iCCA vs eCCA). One-way ANOVA test or Student's t-test were used. Data are shown as mean ± SEM. p-values: * ($p \leq 0.05$), ** ($p \leq 0.01$), *** ($p \leq 0.001$). Abbreviations: eCCA, extrahepatic cholangiocarcinoma; iCCA, intrahepatic cholangiocarcinoma; WT, wild type.

Supplementary tables

Table S1. Human primers sequences employed for qPCR (all from Sigma-Aldrich).

Gene	Sequence
SLC22A1 (OCT1)	Forward 5'-GTCGCTTTGCCAGAGACCAT-3'<br>Reverse 5'-CTTCATCCCTCCAACATGACA-3'
SLC22A3 (OCT3)	Forward 5'-ATCGTCAGCGAGTTTGACCTT-3'<br>Reverse 5'-ACCTGTCTGCTGCATAGCCTA-3'
SLC31A1 (CTR1)	Forward 5’-TGCGTAAGTCACAAGTCAGC-3’<br>Reverse 5’-CTGCTACTGCAATGCAGAGG-3’
SLC51A (OST-α)	Forward 5’- TTCCAGGTTCTCCTCATCCTGAC -3’<br>Reverse 5’- CAATTCATCACTTGAGACCTGGTTT-3’
GADPH	Forward 5’-CCAAGGTCATCCATGACAAC-3’<br>Reverse 5’-TGTCATACCAGGAAATGAGC-3’

Abbreviations: CTR1, copper transporter 1; GAPDH, glyceraldehyde-3-phosphate dehydrogenase; OCT1, organic cation transporter 1; OCT3, organic cation transporter 3; OST, organic solute transporter.

Table S2. Antibodies employed for WB and IHC assays.

Antibody	Clone	Company	Reference	Application
Mouse monoclonal anti-ATR	C-1	Santa Cruz Biotechnology	sc-515173	WB
Rabbit monoclonal anti-p-ATR	T1989	Abcam	ab223258	WB
Mouse monoclonal anti-CHK1	2G1D5	Cell Signaling	2360S	WB
Rabbit monoclonal anti-p-CHK1 (Ser345)	133D3	Cell Signaling	2348T	WB
Mouse monoclonal anti-CDC2	n/a	Santa Cruz Biotechnology	sc-54	WB
Mouse monoclonal anti-p-CDC2	pY15.44	Santa Cruz Biotechnology	sc-136014	WB
Mouse monoclonal anti β-actin	AC-74	Sigma-Aldrich	A5316	WB
Rabbit monoclonal anti-CK19	EP1580Y	Abcam	ab52625	IHC
Rabbit monoclonal anti-CD4	EPR19514	Abcam	ab183685	IHC
Rabbit monoclonal anti-CD8	D4W2Z	Cell Signaling	98941	IHC
Rabbit monoclonal anti-KI67	SP6	Abcam	ab16667	IHC
Rabbit polyclonal anti-PCNA	n/a	Abcam	ab18197	IHC
Rabbit monoclonal anti-Cleaved Caspase-3 (Asp175)	5A1E	Cell Signaling	9664	IHC
Rabbit polyclonal anti-phospho-Histone H3 (Ser28)	n/a	Cell Signaling	9713	IHC

n/a: not applicable. Abbreviations: CK19, cytokeratin 19; IHC, immunohistochemistry; p-ATR, phosphorylated ataxia telangiectasia and RAD3-related protein; p-CHK1, phosphorylated chekpoint kinase 1; PCNA, proliferating cell nuclear antigen; WB, western blot.

Table S3. Inhibitor and known fluorescent substrate of each transporter.

Transporter	Fluorescent substrate	Inhibitor
OATP1A2	Rhodamine-123 1 μM	Rifampicin 100 μM
OATP1B3	FITC-GC 1 μM	Taurocholic acid 100 μM
OATP2B1	Dibromofluorescein 1 μM	Mifepristone 20 μM
OCT1	Dihydroethidium 1 μM	Quinine 50 μM
OCT3	Dihydroethidium 1 μM	Quinine 10 μM

Abbreviations: DBF, dibenzylfluorescein; DHE, dihydroethidium; FITC-GC, fluorescent bile acid derivative cholylglycylamido-fluorescein; OATP, organic anion transporting polypeptide; OCT, organic cation transporter

Supplementary references

Author names in bold designate shared co-first authorships.

[1] Merino-Azpitarte M, Lozano E, Perugorria MJ, et al. SOX17 regulates cholangiocyte differentiation and acts as a tumor suppressor in cholangiocarcinoma. J Hepatol 2017;67:72–83.

[2] Erice O, Labiano I, Arbelaiz A, et al. Differential effects of FXR or TGR5 activation in cholangiocarcinoma progression. Biochim Biophys Acta Mol Basis Dis 2018;1864:1335–44.

[3] Olaizola P, Lee-Law PY, Fernandez-Barrena MG, et al. Targeting NAE1-mediated protein hyper-NEDDylation halts cholangiocarcinogenesis and impacts on tumor-stroma crosstalk in experimental models. J Hepatol 2022;77:177–90.

[4] Vlachogiannis G, Hedayat S, Vatsiou A, et al. Patient-derived organoids model treatment response of metastatic gastrointestinal cancers. Science 2018;359:920–6.

[5] **Boj SF, Hwang C II, Baker LA,** et al. Organoid models of human and mouse ductal pancreatic cancer. Cell 2015;160:324–38.

[6] Ahn KS, O'Brien D, Kang YN, et al. Prognostic subclass of intrahepatic cholangiocarcinoma by integrative molecular-clinical analysis and potential targeted approach. Hepatol Int 2019;13:490–500.

[7] **Chaisaingmongkol J, Budhu A,** Dang H, et al. Common Molecular Subtypes Among Asian Hepatocellular Carcinoma and Cholangiocarcinoma. Cancer Cell 2017;32:57-70.e3.

[8] Andersen JB, Spee B, Blechacz BR, et al. Genomic and genetic characterization of cholangiocarcinoma identifies therapeutic targets for tyrosine kinase inhibitors. Gastroenterology 2012;142.

[9] Farshidfar F, Zheng S, Gingras MC, et al. Integrative Genomic Analysis of Cholangiocarcinoma Identifies Distinct IDH-Mutant Molecular Profiles. Cell Rep 2017;18:2780–94.

[10] **Job S, Rapoud D**, Dos Santos A, et al. Identification of Four Immune Subtypes Characterized by Distinct Composition and Functions of Tumor Microenvironment in Intrahepatic Cholangiocarcinoma. Hepatology 2020;72:965–81.

[11] Dong L, Lu D, Chen R, et al. Proteogenomic characterization identifies clinically relevant subgroups of intrahepatic cholangiocarcinoma. Cancer Cell 2022;40:70-87.e15.

[12] **Tsherniak A, Vazquez F**, Montgomery PG, et al. Defining a Cancer Dependency Map. Cell 2017;170:564-576.e16.

[13] **Ma L, Wang L, Khatib SA,** et al. Single-cell atlas of tumor cell evolution in response to therapy in hepatocellular carcinoma and intrahepatic cholangiocarcinoma. J Hepatol 2021;75:1397–408.

[14] Schneider CA, Rasband WS, Eliceiri KW. NIH Image to ImageJ: 25 years of image analysis. Nat Methods 2012;9:671–5.

[15] Demichev V, Messner CB, Vernardis SI, et al. DIA-NN: neural networks and interference correction enable deep proteome coverage in high throughput. Nature Methods 2019 17:1 2019;17:41–4.

[16] **Yu F, Haynes SE,** Teo GC, et al. Fast Quantitative Analysis of timsTOF PASEF Data with MSFragger and IonQuant. Mol Cell Proteomics 2020;19:1575–85.

[17] Krämer A, Green J, Pollard J, et al. Causal analysis approaches in Ingenuity Pathway Analysis. Bioinformatics 2014;30:523–30.

[18] Perez-Riverol Y, Bandla C, Kundu DJ, et al. The PRIDE database at 20 years: 2025 update. Nucleic Acids Res 2025;53:D543–53.

[19] Rizvi S, Fischbach SR, Bronk SF, et al. YAP-associated chromosomal instability and cholangiocarcinoma in mice. Oncotarget 2017;9:5892–905.
