# Supplementary material for: New platinum derivatives selectively cause double-strand DNA breaks and death in naïve and cisplatin-resistant cholangiocarcinomas
Source: J Hepatol. 2025 Nov;83(5):1077–91. doi: 10.1016/j.jhep.2025.04.034 (PMC12547501; doi:10.1016/j.jhep.2025.04.034)
Supplement: Multimedia component 2 [file mmc2.pdf]

Supplementary information – chemical synthesis

New platinum derivatives selectively cause double-strand DNA breaks and death in naïve and cisplatin-resistant cholangiocarcinomas

Irene Olaizola, Mikel Odriozola-Gimeno, Paula Olaizola, Francisco J. Caballero-Camino, Noelia Pastor-Toyos, Mireia Tena-Garitaonandia, Ainhoa Lapitz, Beatriz Val, Amanda R. Guimaraes, Maitane Asensio, Maider Huici-Izagirre, Colin Rae, David de Sancho, Xabier Lopez, Pedro M. Rodrigues, Elisa Herraez, Oscar Briz, Laura Izquierdo-Sanchez, Aitziber Eleta-Lopez, Alexander M. Bittner, Ana Martinez-Amesti, Teresa Miranda, Sumera I. Ilyas, Chiara Braconi, Maria J. Perugorria, Luis Bujanda, Iván Rivilla, Jose J.G. Marin, Fernando P. Cossio, Jesus M. Banales

General Remarks

Unless otherwise noted, reagents and substrates were purchased from commercial suppliers. TLC was performed on 0.25mm silica gel 60 F254 aluminum plates and visualized with UV lamps. Column chromatography was carried out on columns of silica gel 60 (particle size 40-63 μm).A Raney-Nickel cartridge-equipped continuous flow reactor was employed for the hydrogenation reaction. Hydrogen gas was in situ generated by electrolysis of water. Infrared spectra were recorded on an Alpha-Bruker FTIR spectrometer with a single reflection ATR module. Wavenumbers are given in cm^{-1}. High Resolution Mass Spectra (HRMS) analyses were carried out by SGIker services (Central Service of Alava and Bizkaia, University of the Basque Country) and performed on an LQ/QTOF, Agilent mass spectrometer using electrospray ionization (ESI)

mode. NMR spectra were recorded at 400 or 500 MHz for ^{1}H NMR, 101 or 126 MHz for 13C NMR, and 107 MHZ for 195Pt NMR using $CDCl_3$, DMSO-*d6* and DMF-*d7* as solvent and TMS as internal standard. The data are reported as s=singlet, d=doublet, t=triplet and m=multiplet, coupling constant(s) (J) in Hz, integration. ^{13}C NMR spectra were recorded with ^{1}H decoupling. The water signal was suppressed when DMF-d7 was employed as solvent.

Experimental Procedure and Characterization Data

Synthesis of 2-bromo-1-phenylethan-1-one (2) [1]

A mixture of **1** (44.64 mmol, 1 eq.), acetic acid (56 mL), and 48% aqueous HBr (44.64 mmol, 1 eq.) was cooled in an ice-bath and Br_2 (51.04 mmol, 1.1 eq.) was added dropwise. Once the bromine was added the stirring continued firstly at r.t. for 1h and afterwards at 75˚C for 1.5 h under argon. The reaction mixture was cooled to r.t., diluted with THF (90 mL), and stirred at room temperature overnight. The residue was diluted with EtOAc, washed with saturated $NaHCO_3$ solution and H_2O, dried ($MgSO_4$), and concentrated in vacuo. The liquid residue was purified over silica gel chromatography (1:8 EtOAc:Hexane).

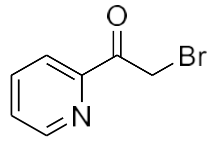

2-bromo-1-(pyridin-2-yl)ethan-1-one (**2**). Yield: 33%, reddish oil. The NMR spectral data were in good agreement with those previously reported.[1] 1H NMR (400 MHz, DMSO-*d6*) D 10.44 (s, 1H), 7.83-7.85 (d, J = 7.6 Hz, 2H), 7.53 (d, J = 8.0 Hz, 2H), 7.36-7.40 (t, J = 7.6 Hz, 2H), 7.25-7.29 (t, J = 7.4 Hz, 1H), 7.19 (s, 1H), 7.10 (d, J = 8.4 Hz, 2H), 2.21 (s, 3H).

Synthesis of Methyl 2-(pyridin-2-yl)imidazo[1,2-a]pyridine-7-carboxylate (4)

2-bromo-1-(pyridin-2-yl)ethan-1-one (**2**) (6.20 mmol, 1.0 eq.) and 2-aminopyridine (**3**) (7.75 mmol, 1.25 eq.) were dissolved in an ethanol solution (20 mL). $NaHCO_3$ (9.67 mmol, 1.56 eq.) was added and the reaction mixture was refluxed overnight. The resulting mixture was concentrated. Then, water was added and the resulting mixture was extracted with DCM. The combined organic layer was washed with brine, dried with $MgSO_4$, and concentrated under vacuum. The resulting solid was purified over silica gel chromatography (1:1 EtOAc:Hexane).

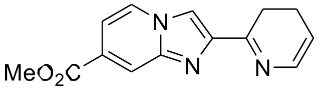

Methyl 2-(pyridin-2-yl)imidazo[1,2-a]pyridine-7-carboxylate (**4**). Yield: 72%, reddish-white solid. m_p= 225-226˚C. FTIR (neat, cm^{-1}) 1715, 1593, 1327, 1228, 1117, 761, 740. ^1H NMR (400 MHz, $CDCl_3$) δ 8.65 (dd, J = 4.8, 0.9 Hz, 1H), 8.40 (s, 1H), 8.37 (s, 1H), 8.26–8.19 (m, 2H), 7.83 (td, J = 7.7, 1.8 Hz, 1H), 7.43 (dd, J = 7.1, 1.7 Hz, 1H), 7.31–7.26 (m, 1H), 3.99 (s, 3H). ^{13}C NMR (101 MHz, $CDCl_3$) δ 165.55, 152.30, 149.45, 147.94, 144.48, 136.89, 126.32, 125.45, 123.09, 120.67, 120.46, 112.26, 112.04, 52.58. HRMS (ESI) for $C_{14}H_{11}N_3O_2$ $[M+H]^+$: calculated 254.0851, found 254.0932.

General procedure for the coupling reactions to synthesize compounds (5) and (6)

Compounds **5** and **6** (1.98 mmol, 1 eq.), $Pd(PPh_3)_4$ (0.139 mmol, 7 mol%) and K_2CO_3 (5.93 mmol, 3 eq.) were dissolved in DMF (15mL) under argon in a sealed microwave tube. The mixture was stirred for 10 minutes, and the corresponding bromo-derivatives (2.96 mmol, 1.5 eq.) were added.

Then, the resulting reaction mixture was heated to 160˚C and stirred for 16h. After completion of the coupling reaction, the solvent was removed, and the mixture dissolved in DCM and filtered. The resulting residue was purified over silica gel chromatography (EtOAc) to obtain the desired products.

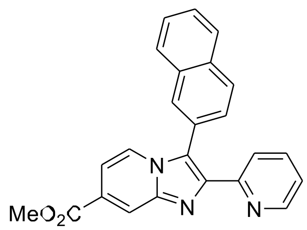

Methyl 3-(naphthalen-2-yl)-2-(pyridin-2-yl)imidazo[1,2-a] pyridine-7-carboxylate (**5**). Yield: 58%, clear brown solid. m_p= 172-173˚C. FTIR (neat, cm^{-1}) 1713, 1331, 1225, 1141, 1088, 816, 755, 737. ^1H NMR (400 MHz, $CDCl_3$) δ 8.59 (ddd, J = 4.8, 1.8, 0.9 Hz, 1H), 8.49 (dd, J = 1.7, 1.0 Hz, 1H), 8.11 (dd, J = 7.2, 1.0 Hz, 1H), 8.10–8.03 (m, 1H), 8.03 (d, J = 8.4 Hz, 1H), 7.97 (dd, J = 7.5, 2.6 Hz, 1H), 7.92 (d, J = 7.4 Hz, 1H), 7.75 (dt, J = 8.0, 1.1 Hz, 1H), 7.66–7.53 (m, 4H), 7.39 (dd, J = 7.4, 1.8 Hz, 1H), 7.19 (ddd, J = 7.5, 4.9, 1.2 Hz, 1H), 4.01 (s, 3H). ^{13}C NMR (101 MHz, $CDCl_3$) δ 165.65, 152.68, 149.71, 144.41, 143.86, 136.15, 136.09, 133.46, 133.36, 130.60, 129.86, 129.27, 129.01, 128.24, 127.97, 127.88, 127.07, 126.66, 126.47, 126.41, 124.63, 123.14, 122.86, 122.51, 122.46, 120.84, 111.96, 111.87, 77.31, 77.20, 76.99, 76.68, 52.59. HRMS (ESI) for $C_{24}H_{17}N_3O_2$ $[M+H]^+$: calculated 380.1321, found 380.1401.

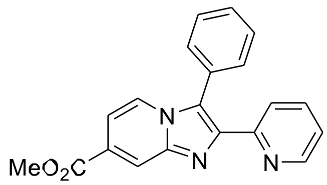

Methyl 3-phenyl-2-(pyridin-2-yl)imidazo[1,2-a]pyridine-7-carboxylate (**6**). Yield: 54%, white solid. m_p= 142-143˚C. FTIR (neat, cm^{-1}) 1716, 1347, 1268, 1225, 1144, 1084, 752, 733, 713. ^1H NMR (400 MHz, $CDCl_3$) δ 8.61 (d, J = 4.9 Hz, 1H), 8.47–8.43 (m, 1H), 8.04 (dd, J = 7.2, 1.0 Hz, 1H), 7.70 (dt, J = 8.0, 1.2 Hz, 1H), 7.63 (td, J = 7.6, 1.8 Hz, 1H), 7.60–7.50 (m, 5H), 7.37 (dd, J = 7.2, 1.7 Hz, 1H), 7.19 (ddd, J = 7.4, 4.8, 1.3 Hz, 1H), 3.99 (s, 3H). ^{13}C NMR (101 MHz, $CDCl_3$) δ 165.63, 152.64, 149.69, 144.13, 143.71, 136.07, 130.57, 129.25, 129.18, 128.97, 126.29, 124.63, 123.08, 122.84, 122.44, 120.77, 111.84, 52.55. HRMS (ESI) for $C_{20}H_{15}N_3O_2$ $[M+H]^+$: calculated 330.1164, found 330.1245.

Procedure for the synthesis of compounds 7 and 8

Compounds **7** and **8** were obtained following the same procedure, which has two steps. The corresponding methyl imidazo[1,2-a]pyridine carboxylate (0.80 mmol, 1 eq.) was dissolved in dry THF (10 mL), to which a 1 M solution of $LiAlH_4$ in THF (1.84 mmol, 2.3 eq.) was added dropwise at 0˚C. The mixture was stirred at room temperature for 2 hours and quenched, adding an excess of MeOH in an ice bath. The solution was concentrated, dissolved in DCM, and washed with $NaHCO_3$ (saturated solution). The resulting residue was purified over silica gel chromatography (1:10 DCM/MeOH).[2] To an ice-cooled solution of the alcohol obtained in the previous step (2.85 mmol, 1 eq.) in 25 mL of DCM, a solution of $SOCl_2$ (5.69 mmol, 2 eq.) in DCM (6 mL) was added dropwise. The reaction mixture was refluxed for 90 min, neutralized with a saturated solution of $NaHCO_3$, and extracted with DCM. The organic layer was dried over $MgSO_4$ and concentrated under reduced pressure. The residue was purified over silica gel chromatography (1:1 EtOAc:Hexane) to obtain the desired product.[3]

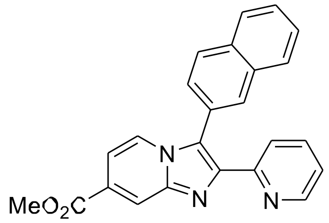

Methyl 3-(naphthalen-2-yl)-2-(pyridin-2-yl)imidazo[1,2-a] pyridine-7-carboxylate (**7**). Yield: 58%, clear brown solid. m_p= 172-173˚C. FTIR (neat, cm^{-1}) 1713, 1331, 1225, 1141, 1088, 816, 755, 737. ^1H NMR (400 MHz, $CDCl_3$) δ 8.59 (ddd, J = 4.8, 1.8, 0.9 Hz, 1H), 8.49 (dd, J = 1.7, 1.0 Hz, 1H), 8.11 (dd, J = 7.2, 1.0 Hz, 1H), 8.10–8.03 (m, 1H), 8.03 (d, J = 8.4 Hz, 1H), 7.97 (dd, J = 7.5, 2.6 Hz, 1H), 7.92 (d, J = 7.4 Hz, 1H), 7.75 (dt, J = 8.0, 1.1 Hz, 1H), 7.66–7.53 (m, 4H), 7.39 (dd, J = 7.4, 1.8 Hz, 1H), 7.19 (ddd, J = 7.5, 4.9, 1.2 Hz, 1H), 4.01 (s, 3H). ^{13}C NMR (101 MHz, $CDCl_3$) δ 165.65, 152.68, 149.71, 144.41, 143.86, 136.15, 136.09, 133.46, 133.36, 130.60, 129.86, 129.27, 129.01, 128.24, 127.97, 127.88, 127.07, 126.66, 126.47, 126.41, 124.63, 123.14, 122.86, 122.51, 122.46, 120.84, 111.96, 111.87, 77.31, 77.20, 76.99, 76.68, 52.59. HRMS (ESI) for $C_{24}H_{17}N_3O_2$ $[M+H]^+$: calculated 380.1321, found 380.1401.

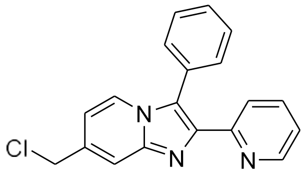

7-(chloromethyl)-3-phenyl-2-(pyridin-2-yl)imidazo[1,2-a] pyridine (**8**). Yield: 68 %, yellow oil. FTIR (neat, cm^{-1}) 3053, 1589, 1508, 1365, 1217, 794, 720, 699. ^{1}H NMR (400 MHz, $CDCl_3$) δ 8.61 (d, J = 4.7 Hz, 1H), 8.01 (d, J = 7.1 Hz, 1H), 7.72–7.68 (m, 1H), 7.67–7.61 (m, 2H), 7.60–7.51 (m, 5H), 7.17 (ddd, J = 7.2, 4.8, 1.5 Hz, 1H), 6.84 (dd, J = 7.2, 1.8 Hz, 1H), 4.67 (s, 2H). ^{13}C NMR (101 MHz, $CDCl_3$) δ 153.03, 149.65, 144.23, 142.54, 136.00, 134.64, 130.72, 129.41, 129.21, 128.97, 123.89, 123.54, 122.71, 122.19, 117.08, 113.07, 45.42. HRMS (ESI) for $C_{20}H_{14}ClN_3$ $[M+H]^+$: calculated 320.0876, found 320.0958.

General procedure for the synthesis of Pt (II) complexes Aurkines 16 and 18 [4]

Chloride **7** or **8** (0.41 mmol, 1.14 eq.) was dissolved in 5 mL of MeOH and $Pt(DMSO)_2Cl_2$ crystals (0.36 mmol, 1 eq.) were added. The mixture was stirred for 16h at r.t. and the yellow precipitate was treated in an ultrasonic bath for 2h, washed with EtOH and ether, and filtered before drying it under vacuum.

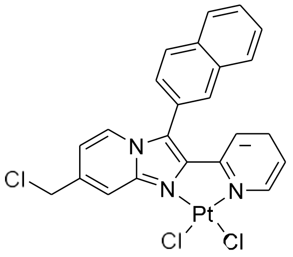

Dichloro(7-(chloromethyl)-3-(naphthalen-2-yl)-2-(pyridin-2 yl)imidazo[1,2-a]pyridine)platinum(II) (**Aurkine 16**). Yield: 71%, yellow solid. mp= > 410 ˚C. FTIR (neat, cm^{-1}) 3032, 1639, 1509, 1245, 1017, 774, 744, 698. ^{1}H NMR (500 MHz, DMF-d7) δ 9.81 (d, J = 5.2 Hz, 1H), 9.46 (s, 1H), 8.75 (s, 1H), 8.58 (d, J = 7.2 Hz, 1H), 8.54 (d, J = 8.4 Hz, 1H), 8.39 (d, J = 8.0 Hz, 1H), 8.32 (d, J = 8.1 Hz, 1H), 8.27 (td, J = 7.9, 1.5 Hz, 1H), 8.15 (dd, J = 8.4, 1.7 Hz, 1H), 8.01–7.89 (m, 2H), 7.82 (td, J = 6.1, 1.5 Hz, 1H), 7.61 (d, J = 8.1 Hz, 1H), 7.54 (dd, J = 7.2, 1.8 Hz, 1H), 5.22 (s, 2H). ^{13}C NMR (126 MHz, DMF) δ 153.26, 148.86, 145.65, 141.56, 141.24, 139.76, 134.37, 133.76, 131.99, 130.22, 128.82, 128.23, 128.15, 127.41, 127.28, 126.75, 125.85, 125.34, 122.46, 121.82, 115.98, 114.58, 44.92. ^{195}Pt NMR (107 MHz, DMF-d7) -2177.42. HRMS (ESI) for $C_{23}H_{16}Cl_3N_3Pt$ $[M+Na]^+$: calculated 657.9955, found 657.9954.

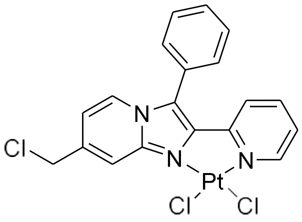

Dichloro(7-(chloromethyl)-3-phenyl-2-(pyridin-2-yl)imidazo [1,2-a]pyridine)platinum(II) (**Aurkine 18**). Yield: 77%, yellow solid. mp= > 410 ˚C. FTIR (neat, cm^{-1}) 3066, 1620, 1510, 1252, 778, 695. ^{1}H NMR (500 MHz, DMF-d7) δ 9.62 (d, J = 5.6 Hz, 1H), 9.26 (s, 1H), 8.20 (dd, J = 7.2, 1.0 Hz, 1H), 8.12 (td, J = 7.8, 1.5 Hz, 1H), 7.92–7.86 (m, 2H), 7.84–7.80 (m, 2H), 7.68–7.58 (m, 1H), 7.39–7.31 (m, 2H), 4.98 (d, J = 0.8 Hz, 2H). ^{13}C NMR (126 MHz, DMF) δ 153.27, 148.92, 145.67, 141.17, 139.64, 131.44, 131.35, 130.37, 126.26, 125.31, 125.06, 121.56, 116.01, 114.65, 44.86. ^{195}Pt NMR (107 MHz, DMF-d7) -2178.24. HRMS (ESI) for $C_{19}H_{14}Cl_3N_3Pt$ $[M+Na]^+$: calculated 607.9799, found 607.9785.

NMR spectra

Compound **4**. **^{1}H NMR ($CDCl_3$)**

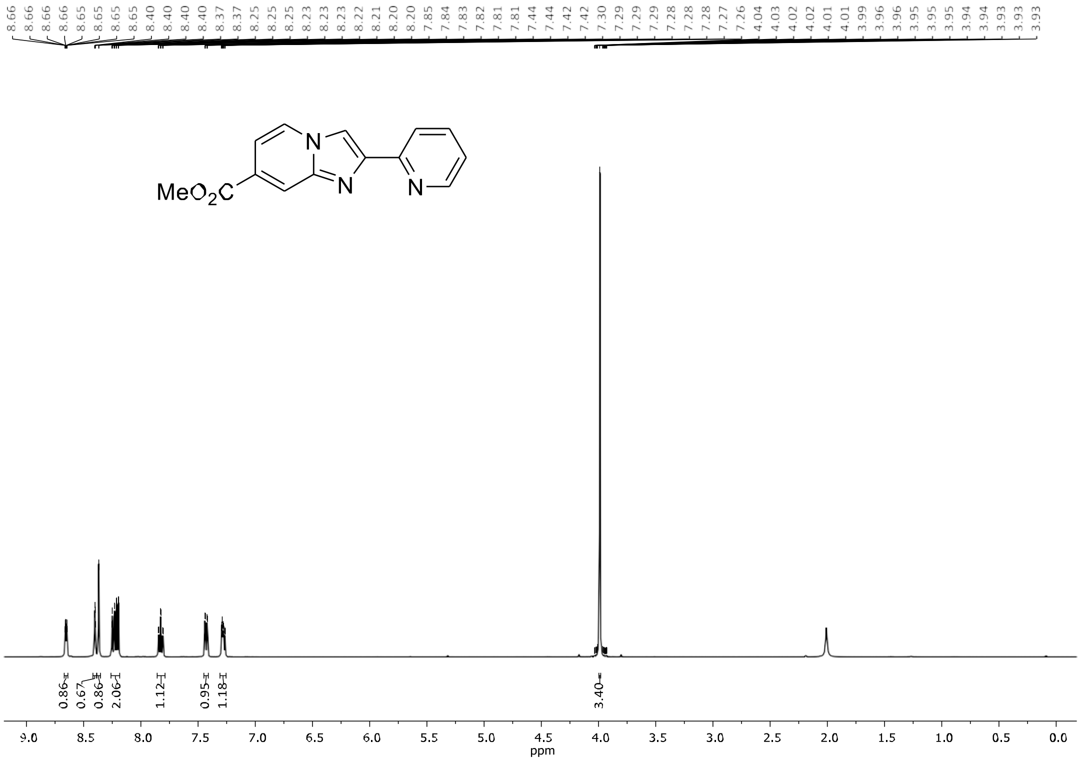

^{13}C NMR ($CDCl_3$)

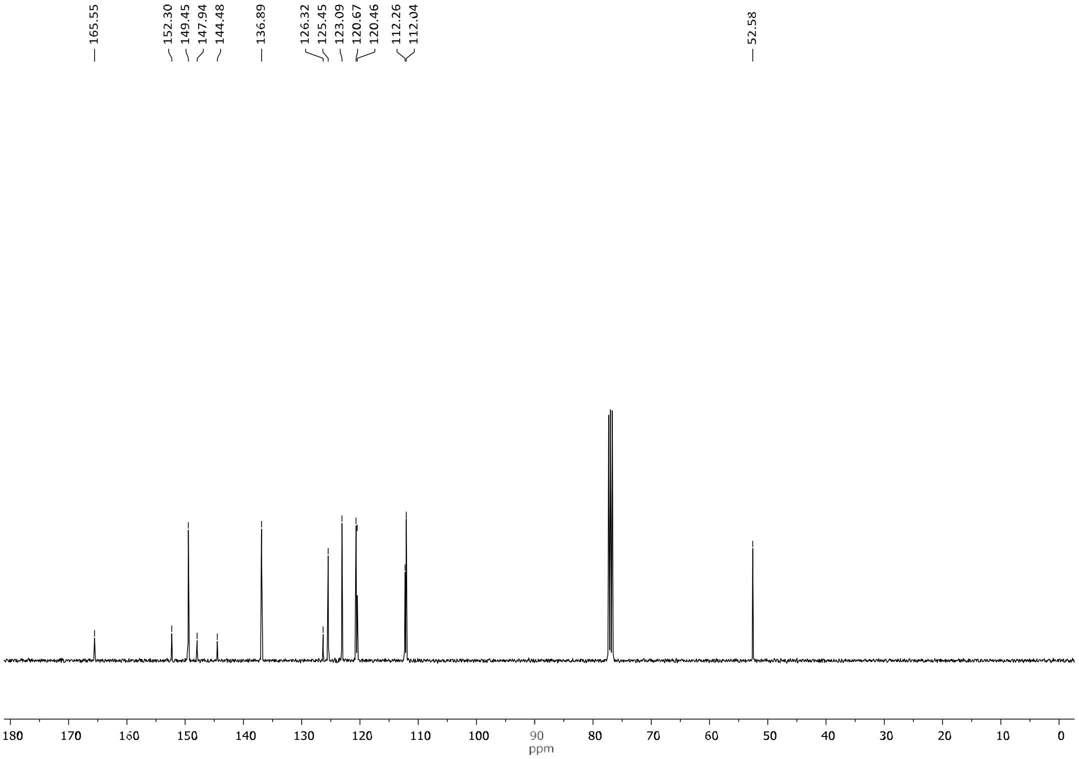

g-COSY ($CDCl_3$)

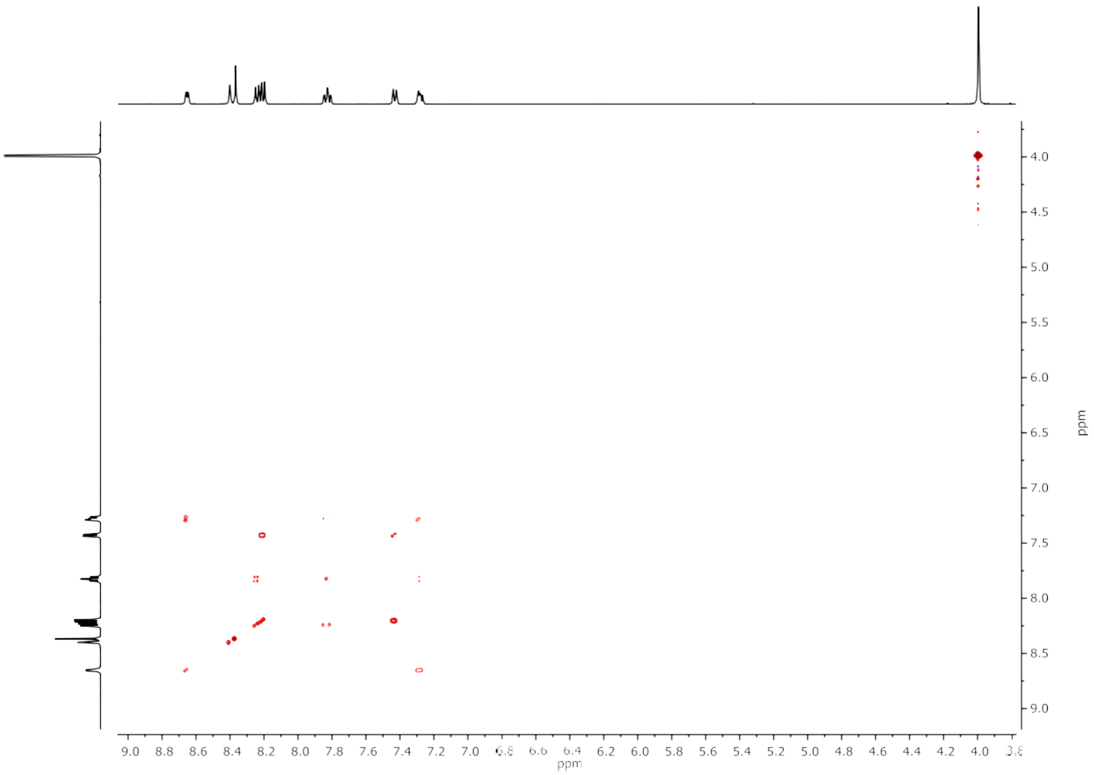

g-HSQC ($CDCl_3$)

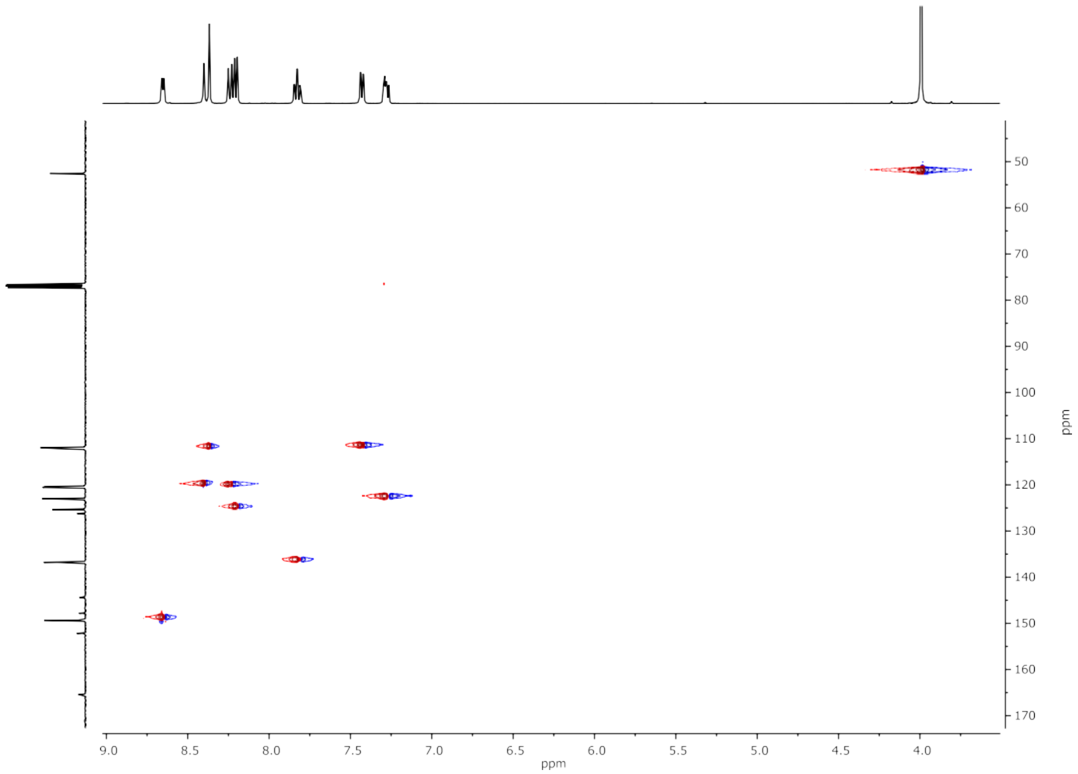

Compound **5**. **^{1}H NMR ($CDCl_3$)**

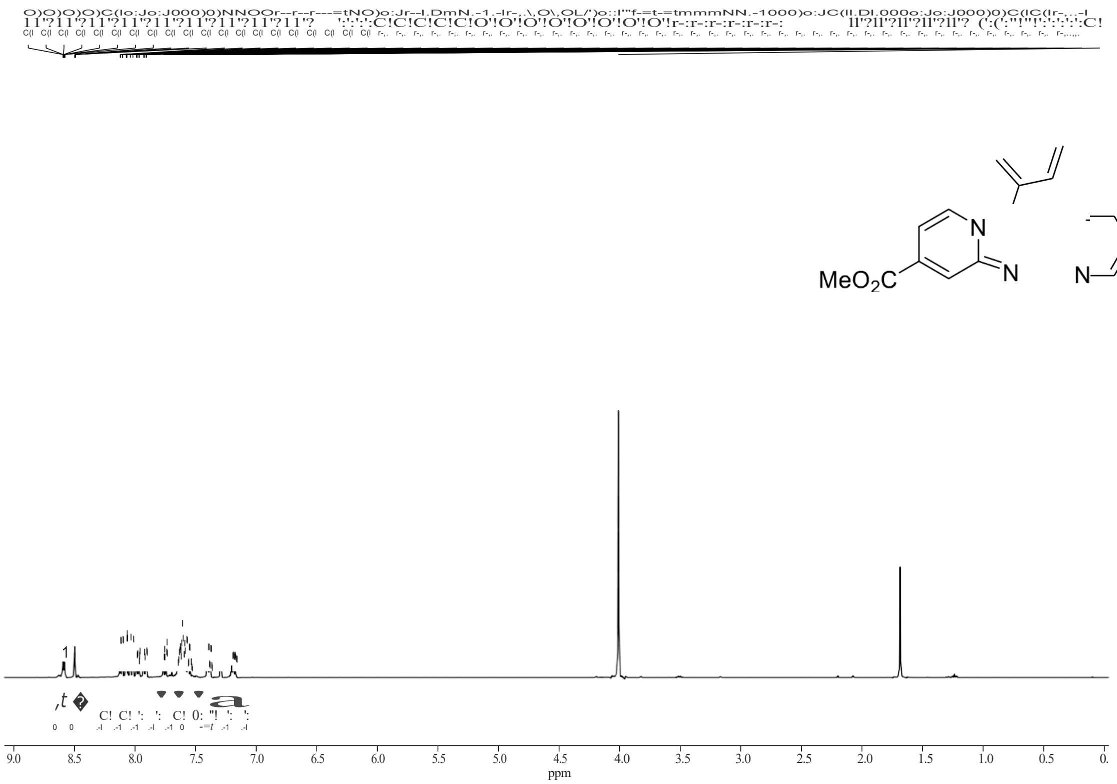

^{13}C NMR ($CDCl_3$)

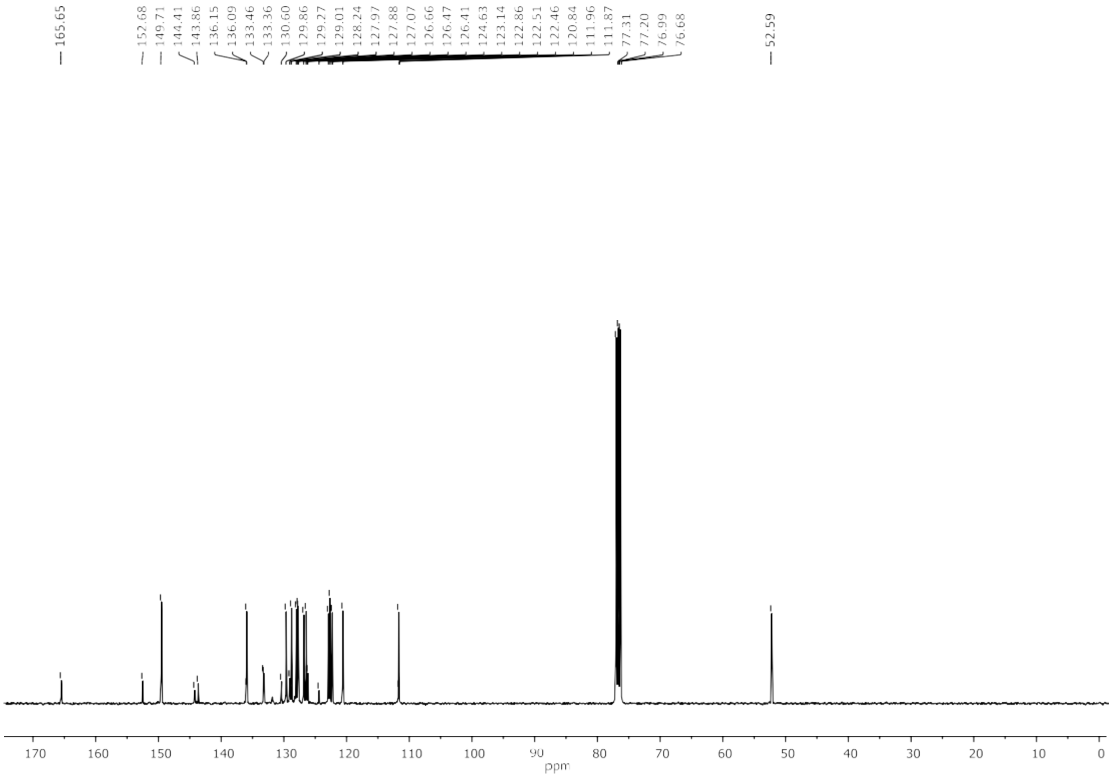

g-COSY ($CDCl_3$)

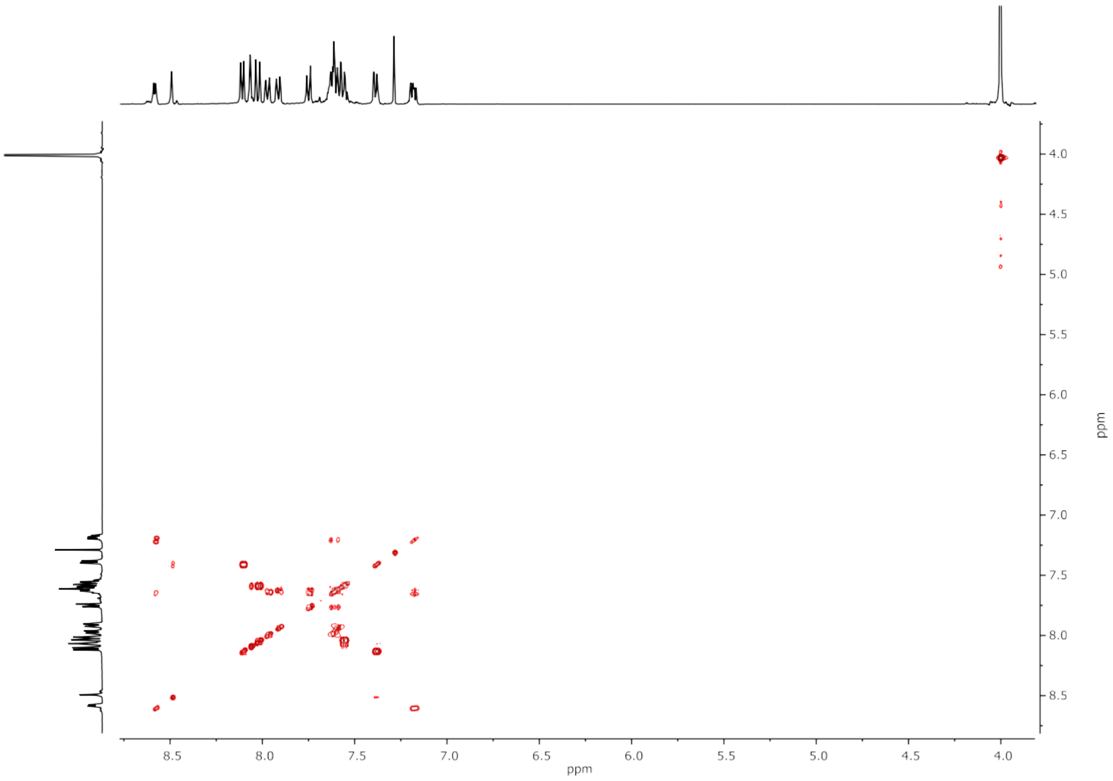

g-HSQC ($CDCl_3$)

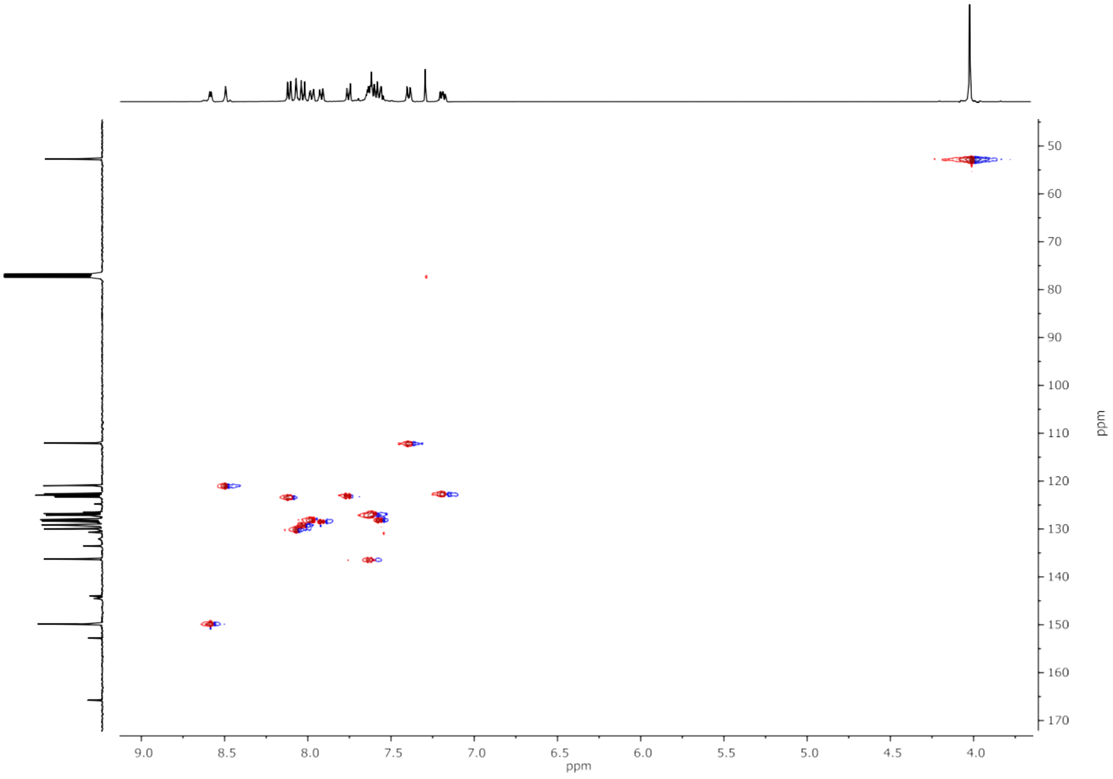

Compound **6**. **^{1}H NMR ($CDCl_3$)**

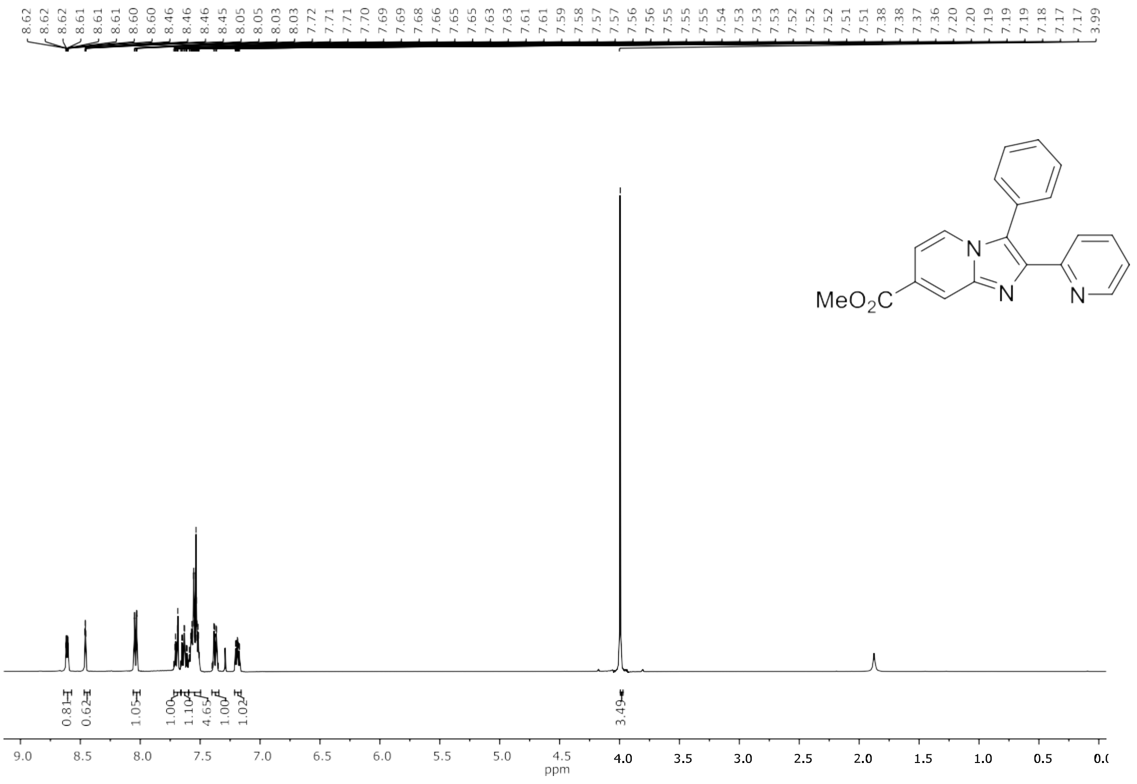

^{13}C NMR ($CDCl_3$)

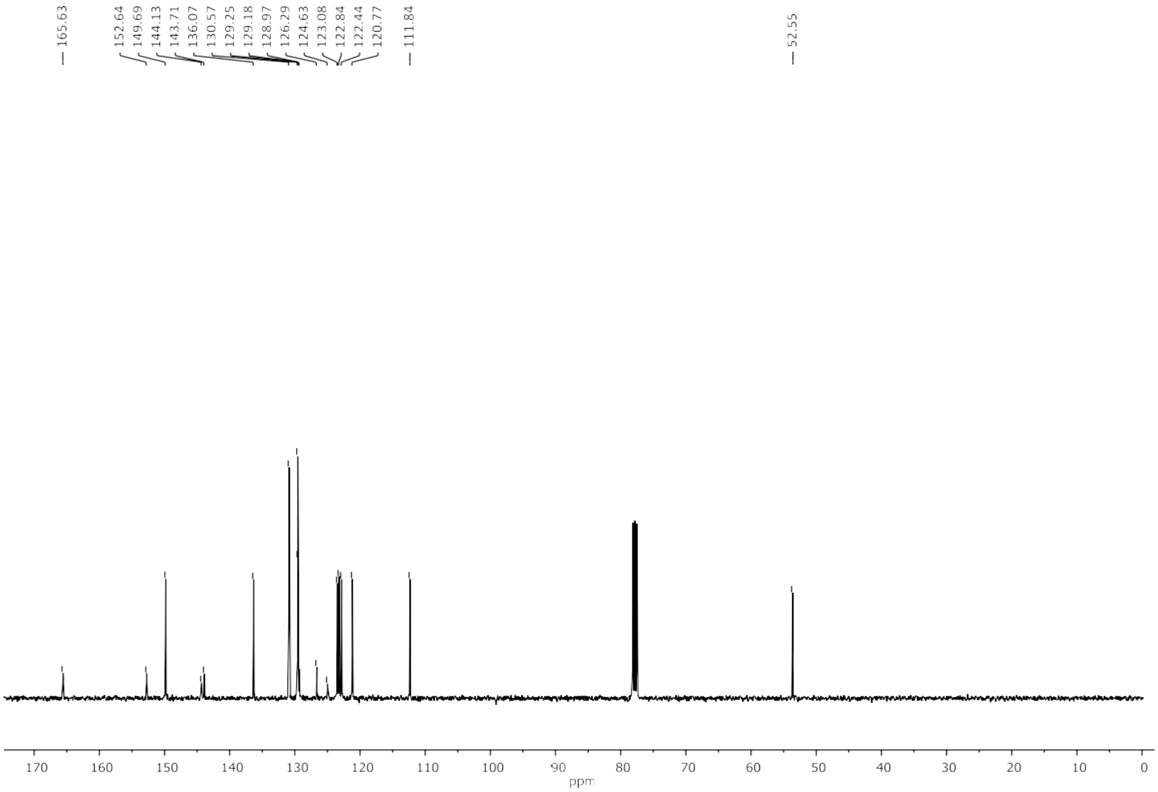

g-COSY ($CDCl_3$)

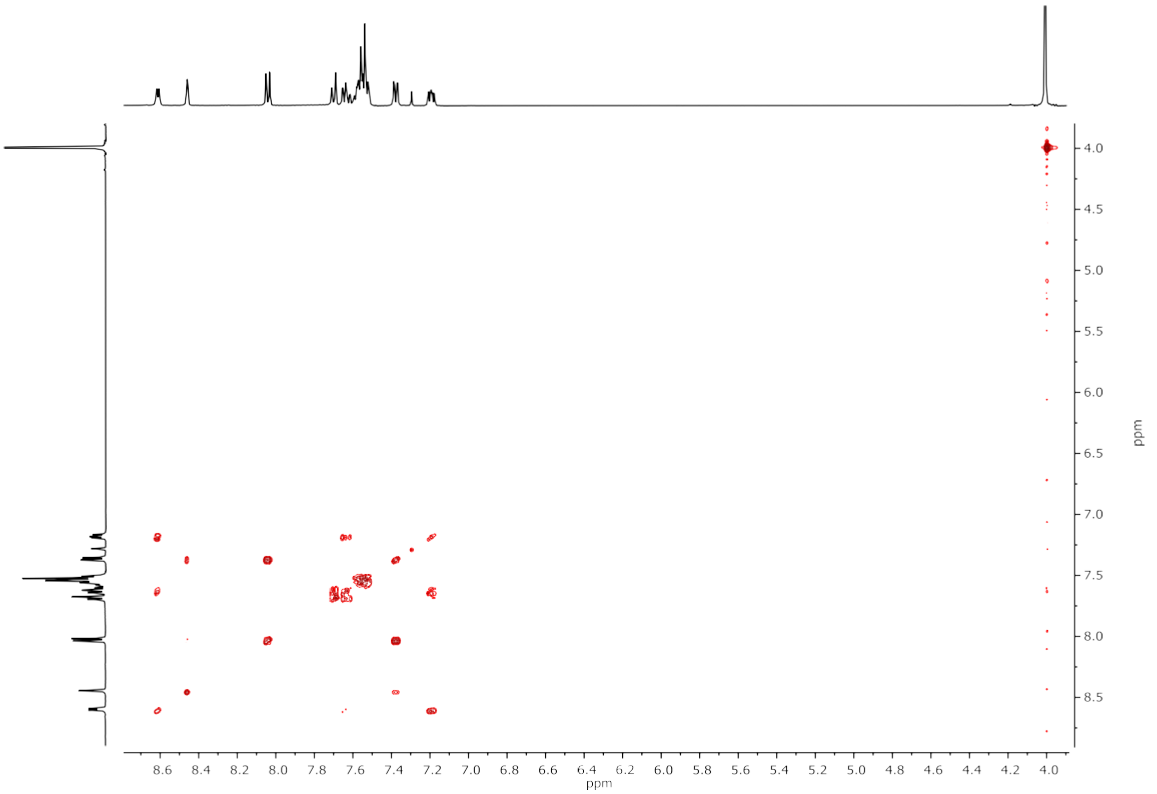

g-HSQC ($CDCl_3$)

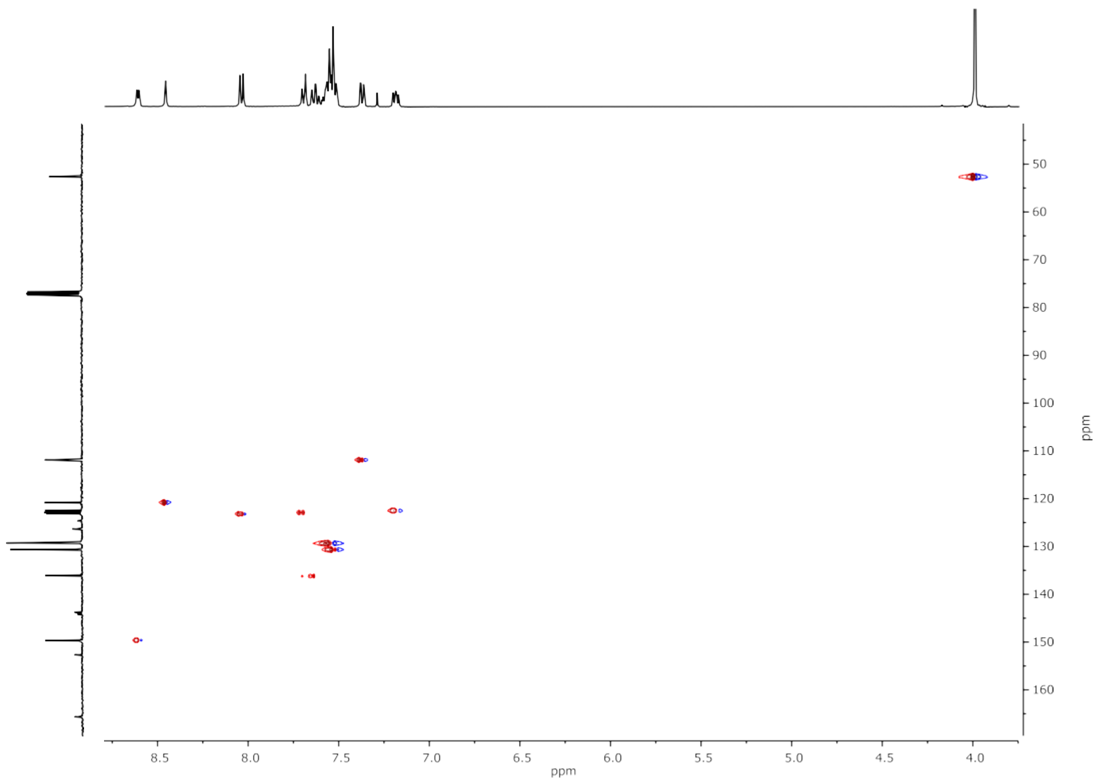

50
60
70
80
90
100
110
120
130
140
150
160
ppm
8.5
8.0
7.5
7.0
6.5
6.0
5.5
5.0
4.5
4.0
ppm

Compound **7.** ^{1}**H NMR (CDCl$_3$)**

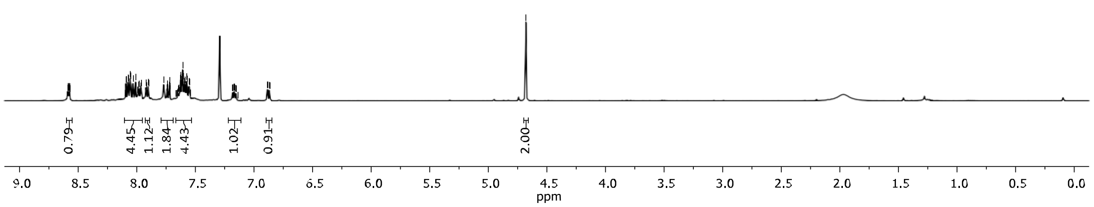

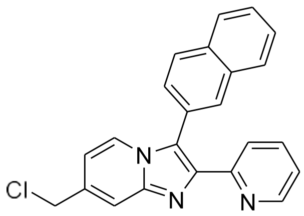

^{13}**C NMR (CDCl$_3$)**

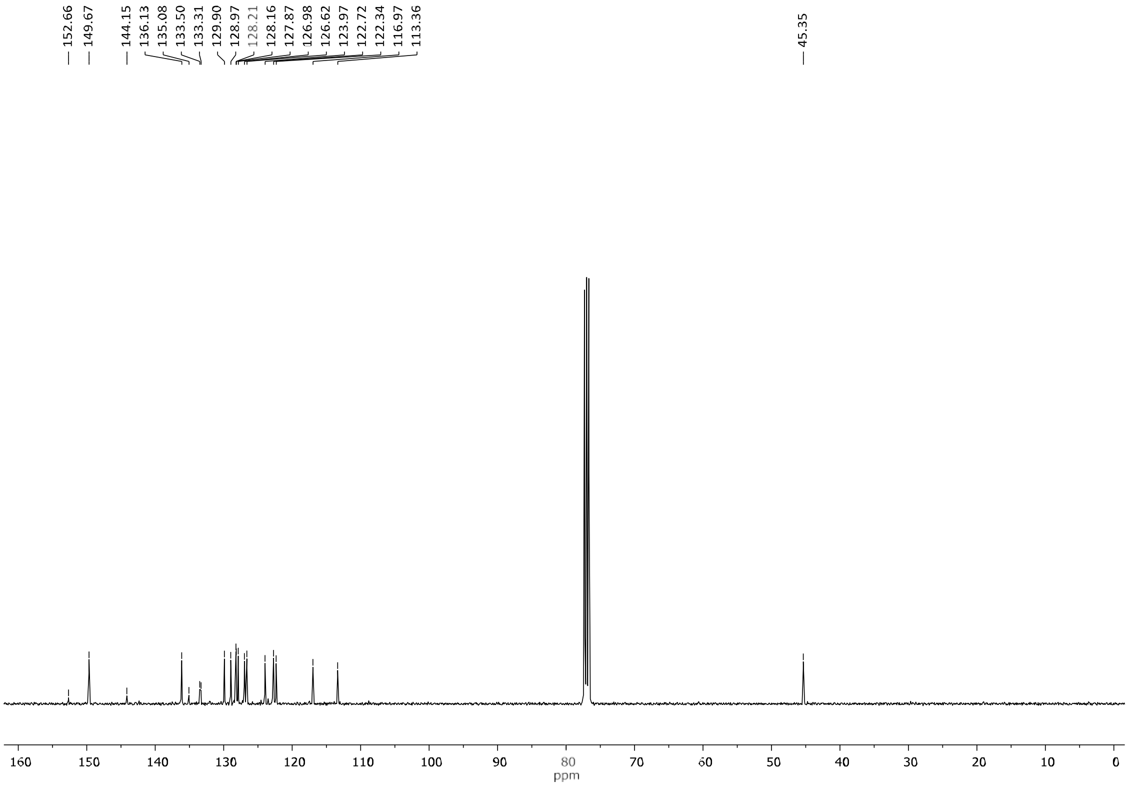

DEPT 135 (CDCl₃)

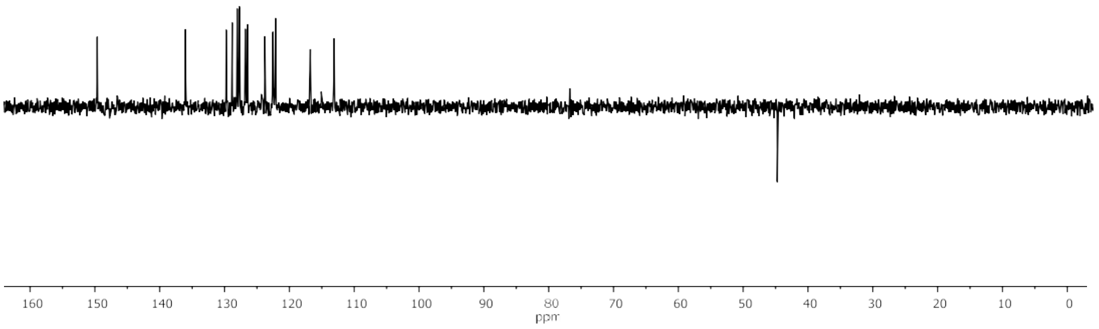

g-COSY (CDCl₃)

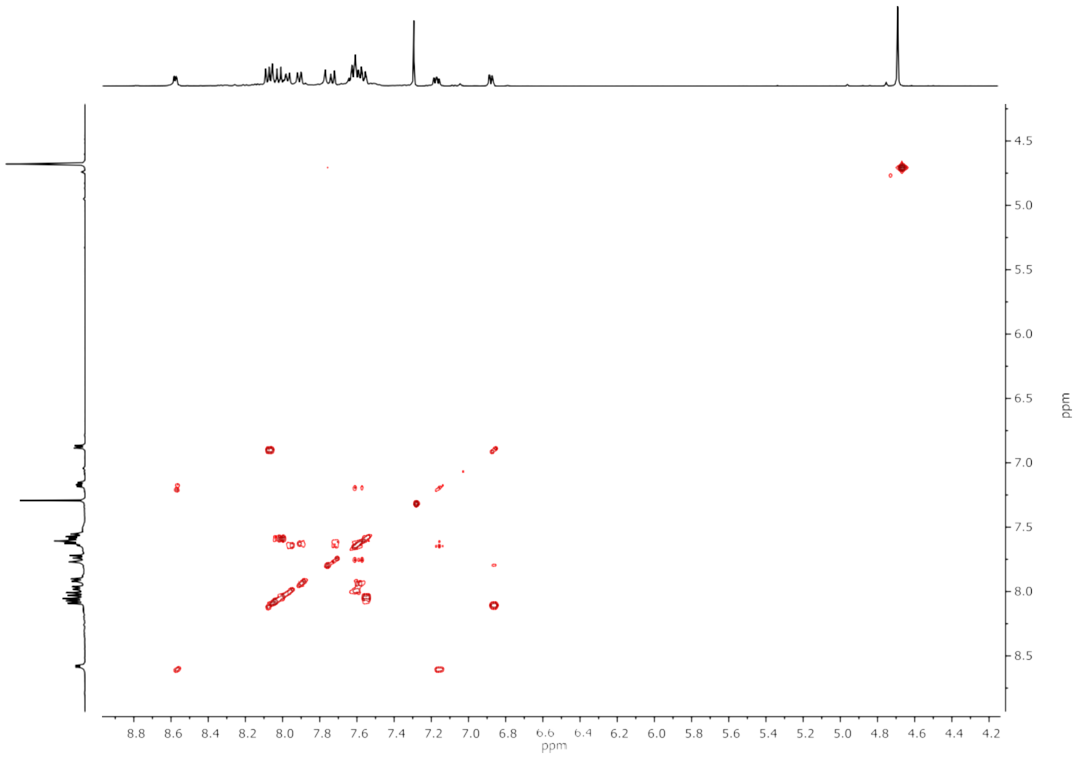

g-HSQC ($CDCl_3$)

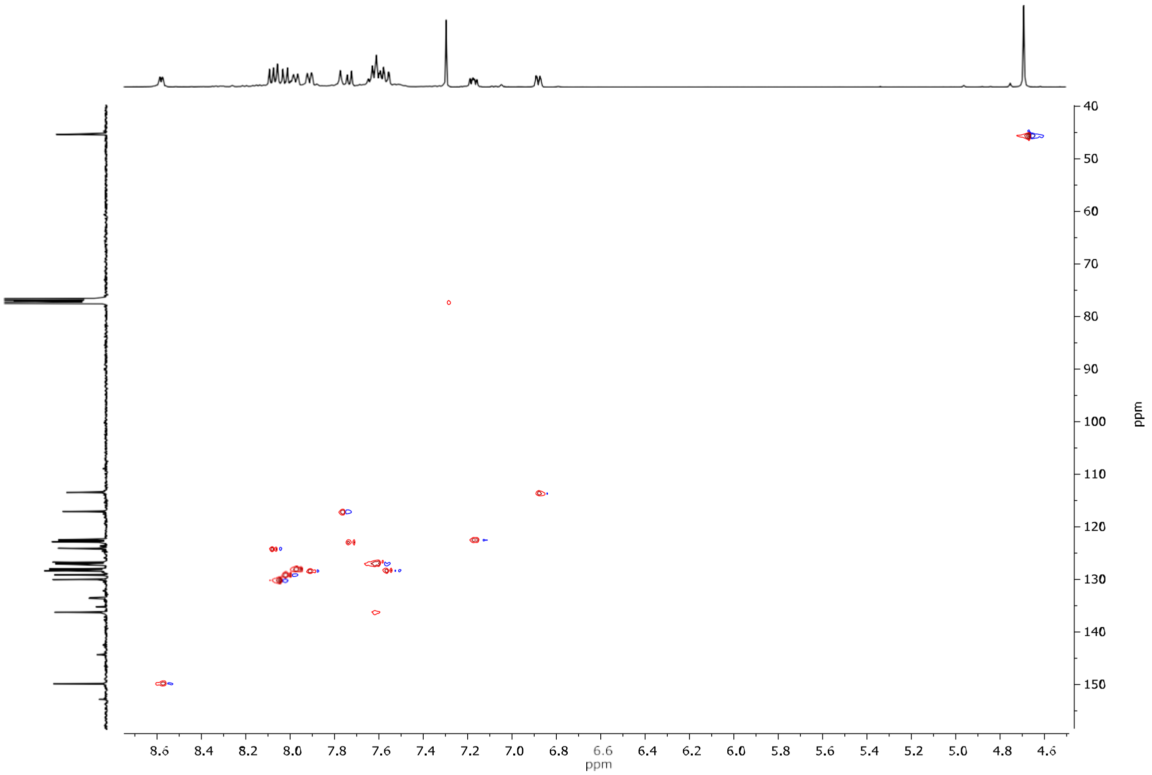

40
50
60
70
80
90
100
110
120
130
140
150
ppm
8.6
8.4
8.2
8.0
7.8
7.6
7.4
7.2
7.0
6.8
6.6
6.4
6.2
6.0
5.8
5.6
5.4
5.2
5.0
4.8
4.6
ppm

Compound **8**. **^{1}H NMR (CDCl$_3$)**

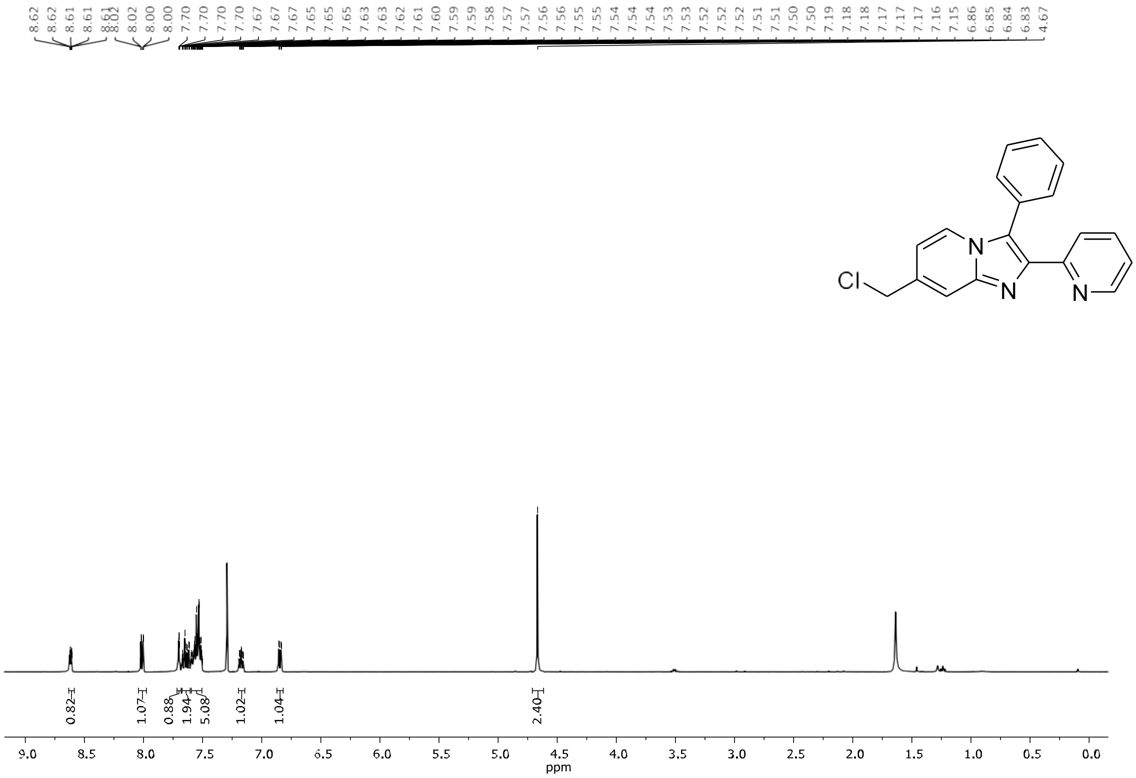

^{13}C NMR (CDCl$_3$)

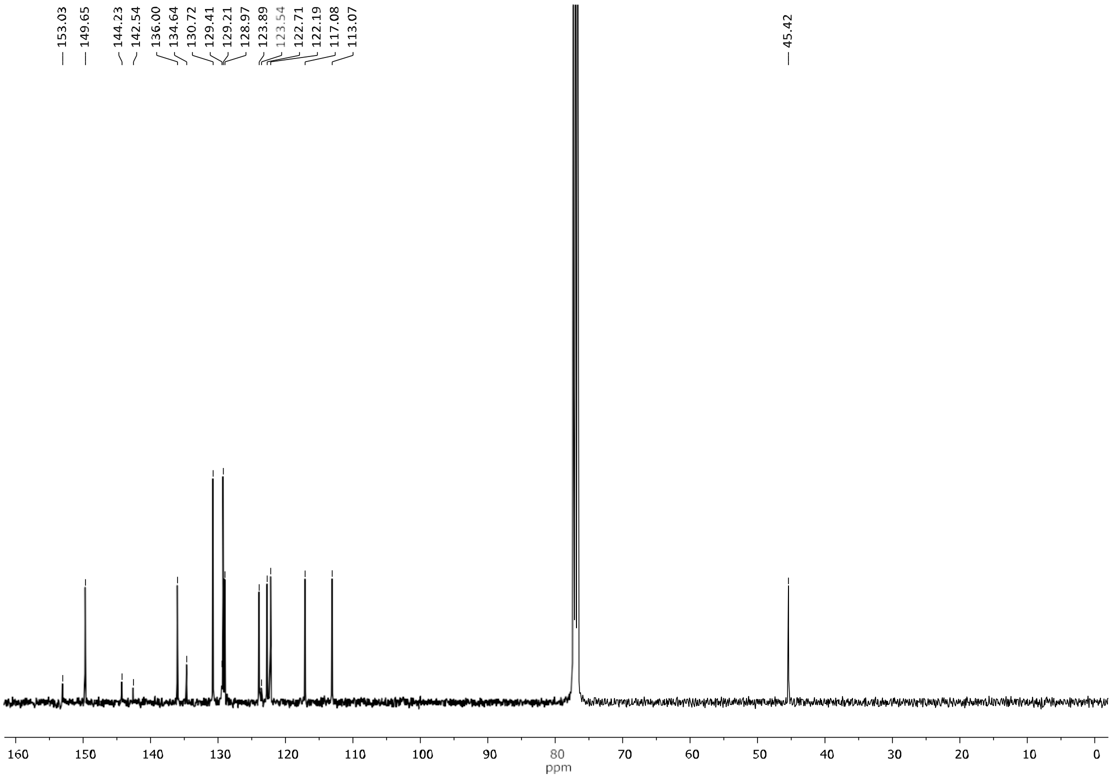

DEPT 135 ($CDCl_3$)

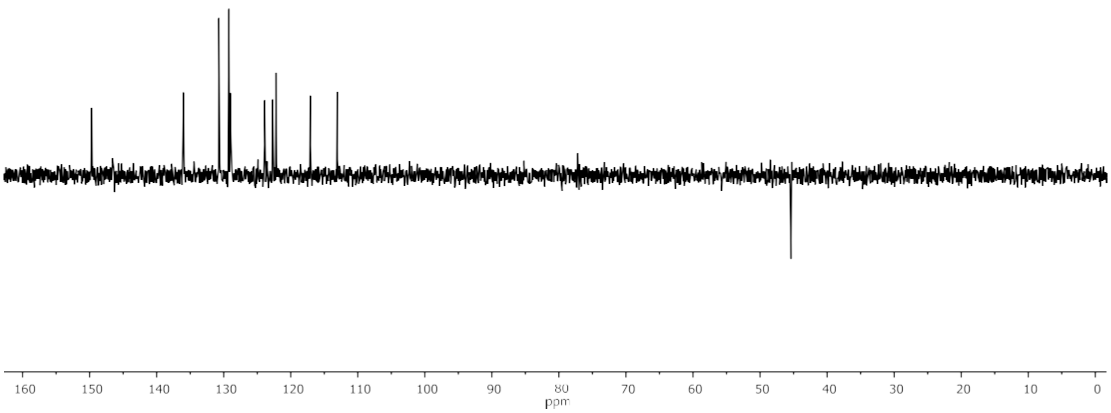

g-COSY ($CDCl_3$)

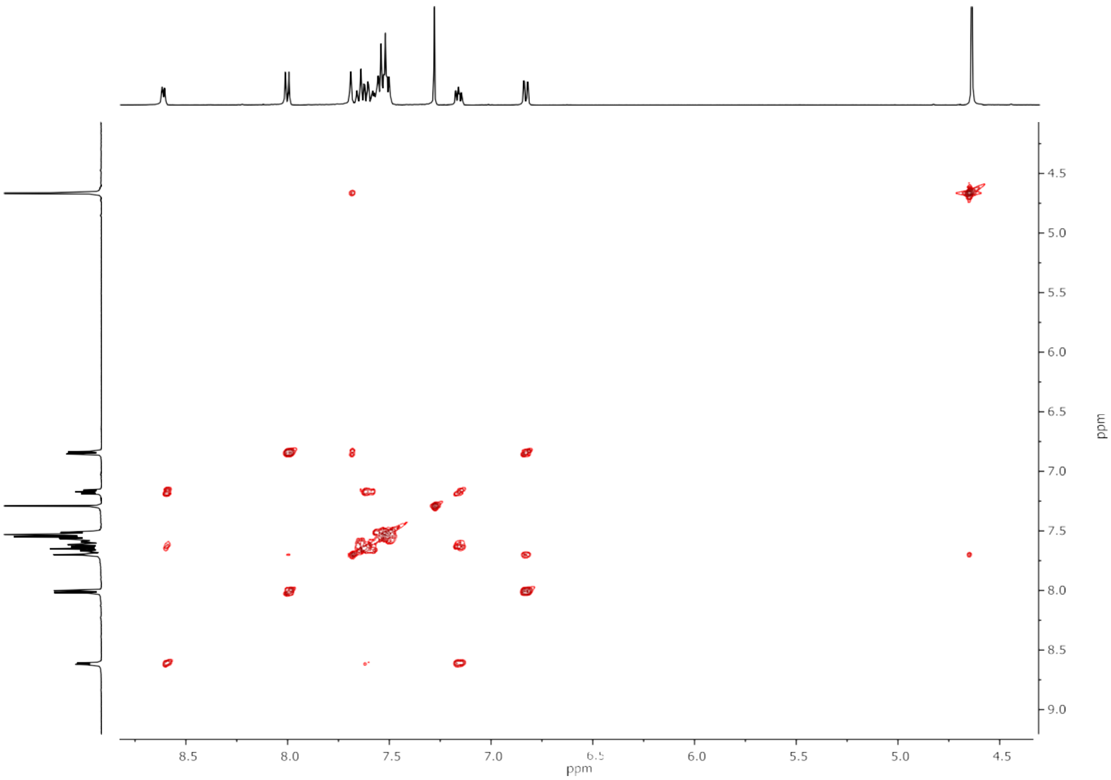

g-HSQC ($CDCl_3$)

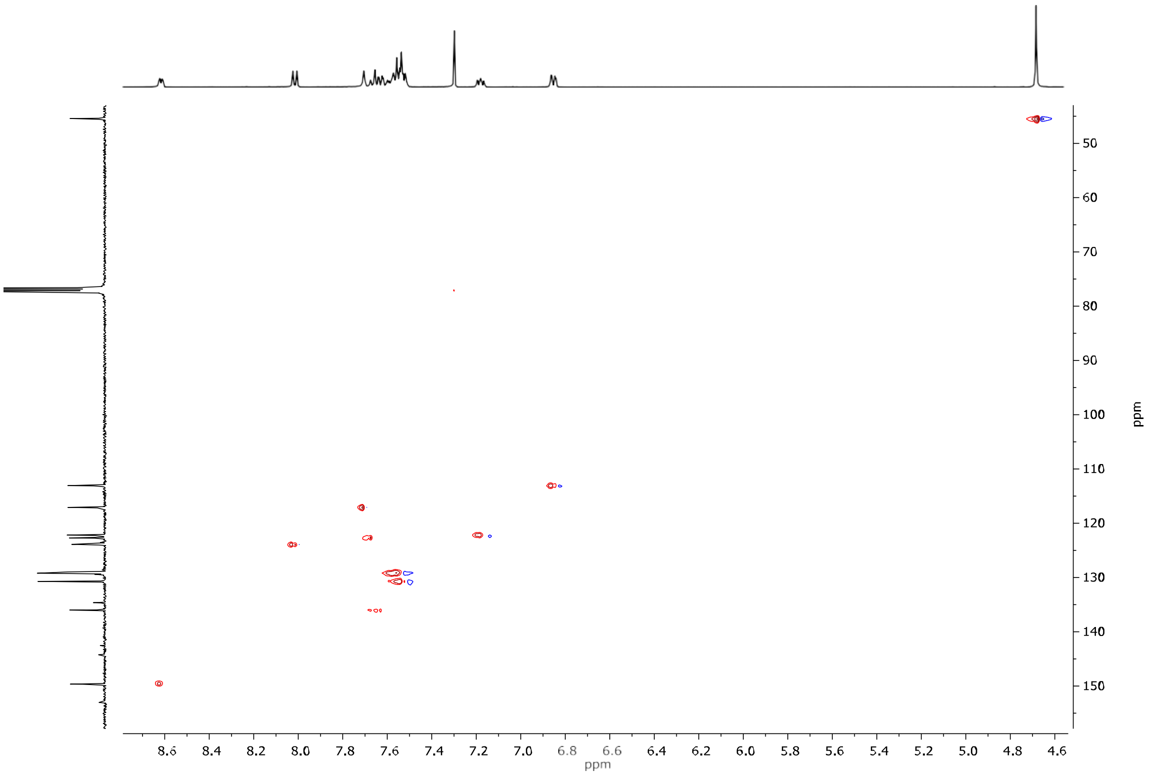

8.6
8.4
8.2
8.0
7.8
7.6
7.4
7.2
7.0
6.8
6.6
6.4
6.2
6.0
5.8
5.6
5.4
5.2
5.0
4.8
4.6
ppm
50
60
70
80
90
100
110
120
130
140
150
ppm

Platinum(II) complex **Aurkine 16**. **^{1}H NMR (DMF-d7)**

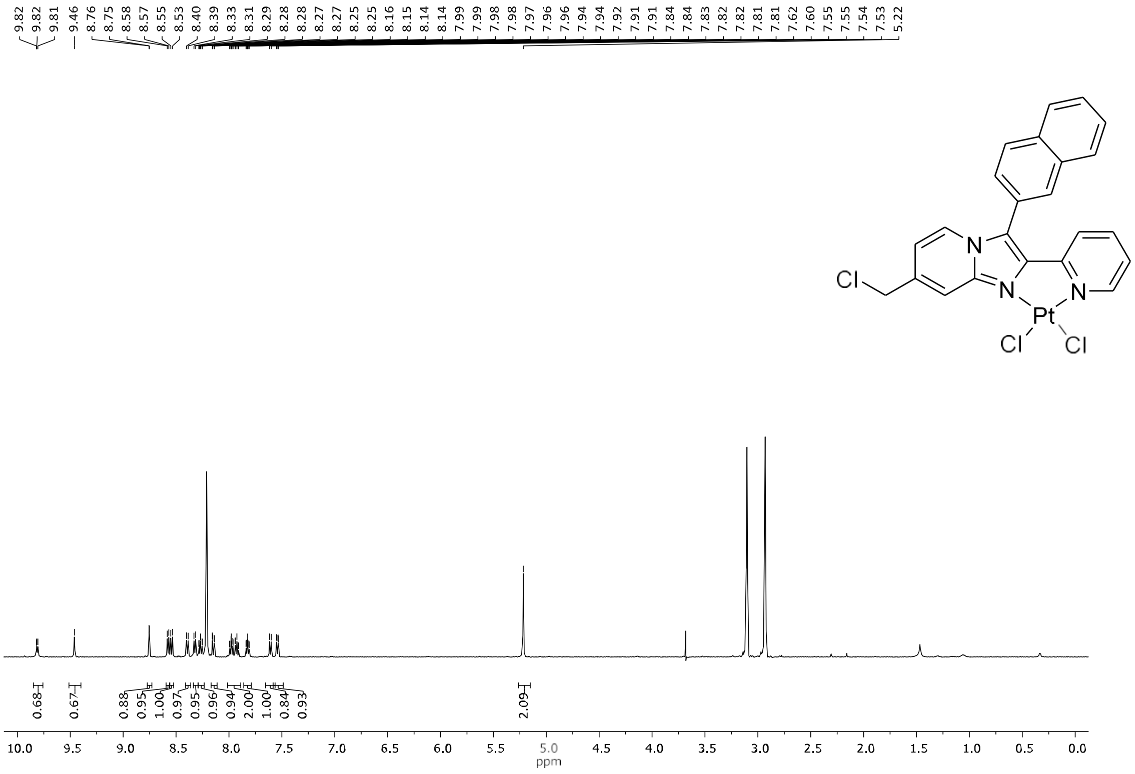

^{13}C NMR (DMF-d7)

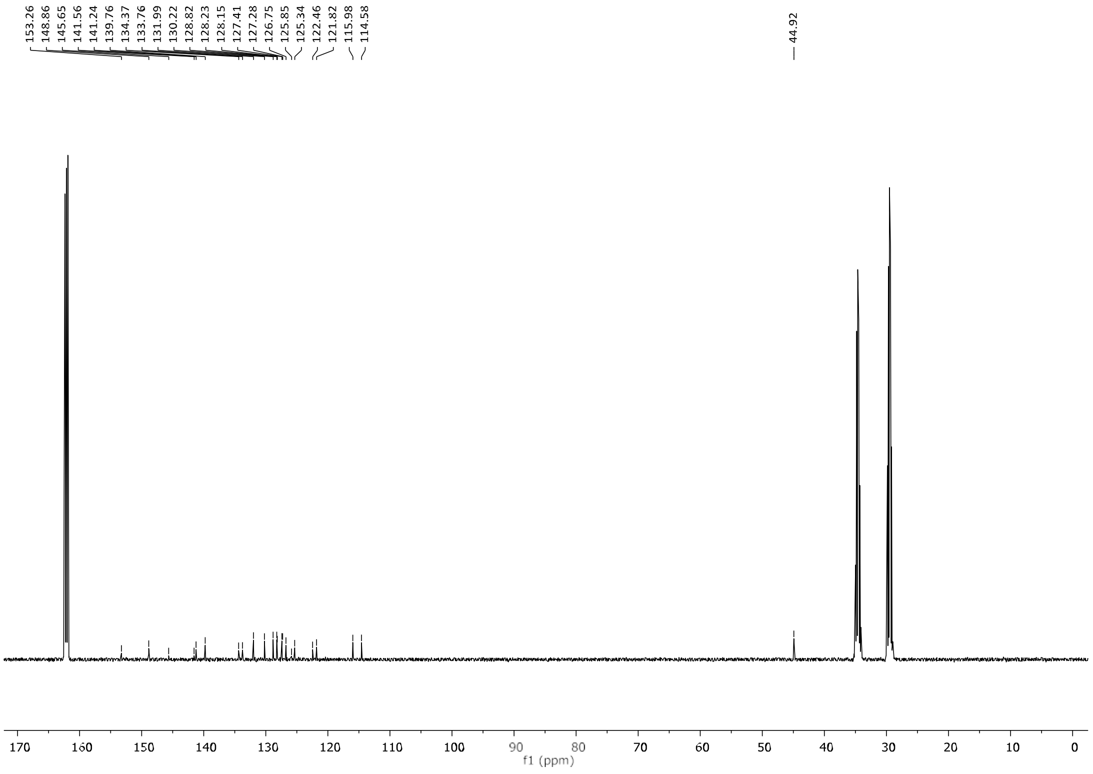

^{195}Pt-NMR (DMF-d7)

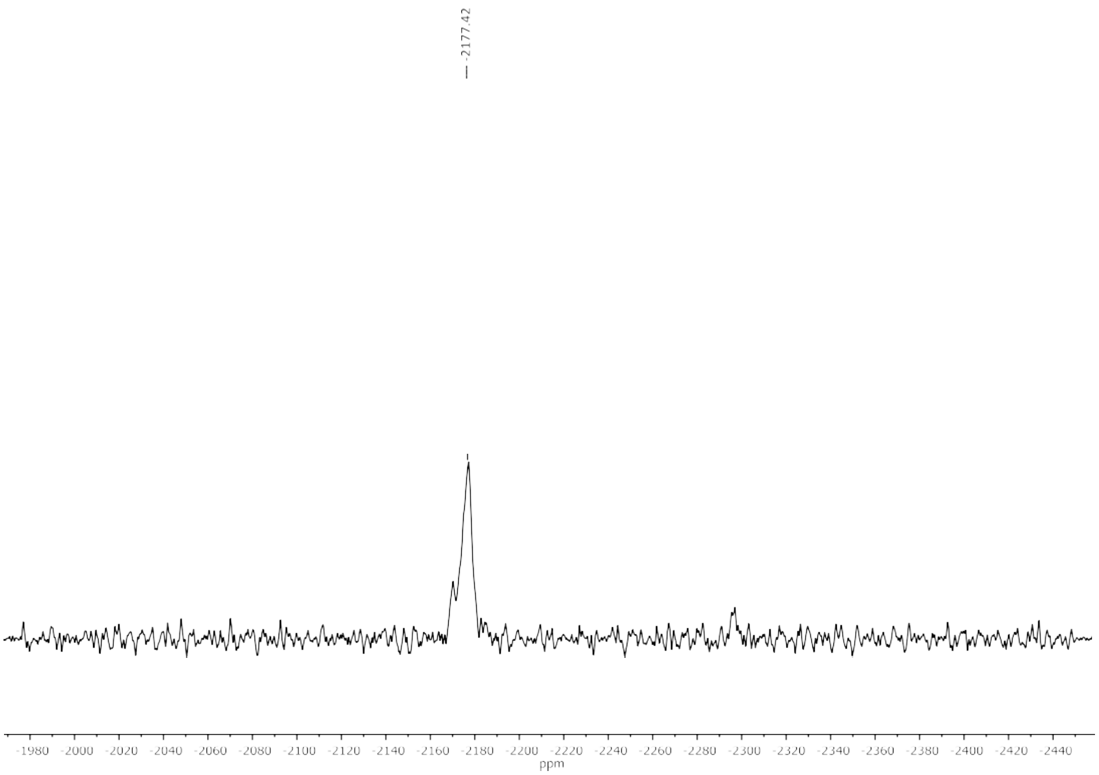

g-COSY (DMF-d7)

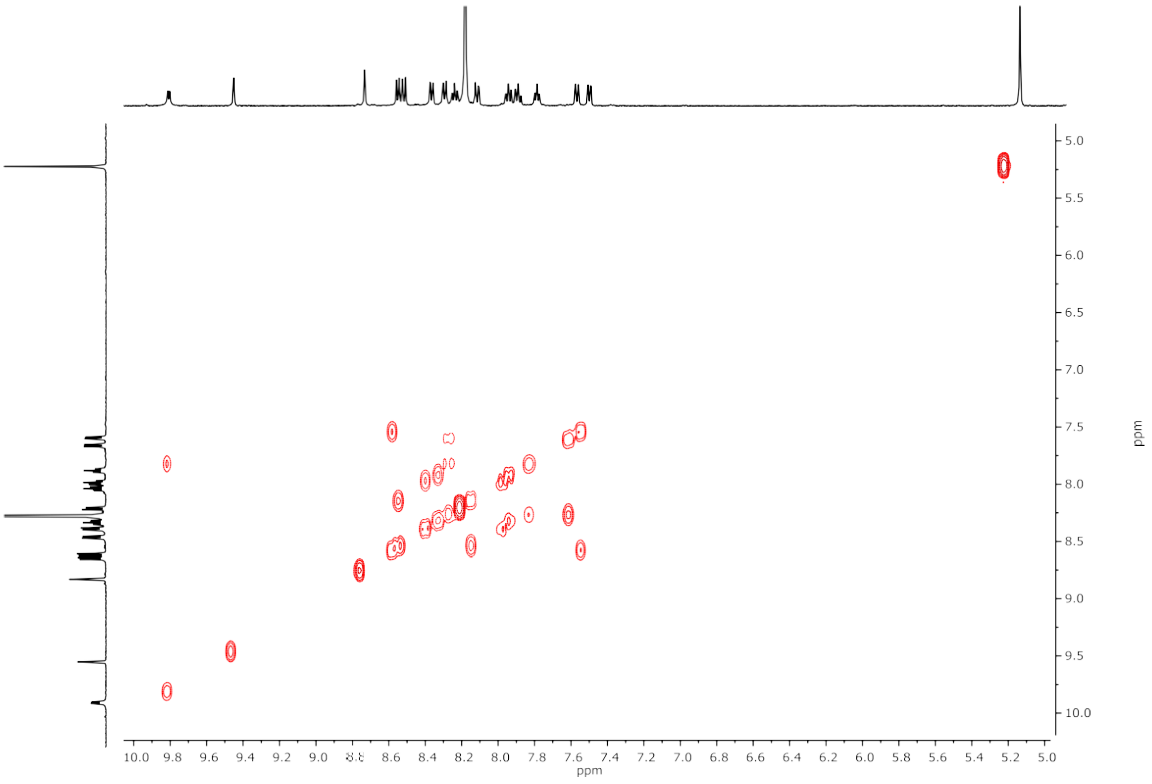

g-HSQC (DMF-d7)

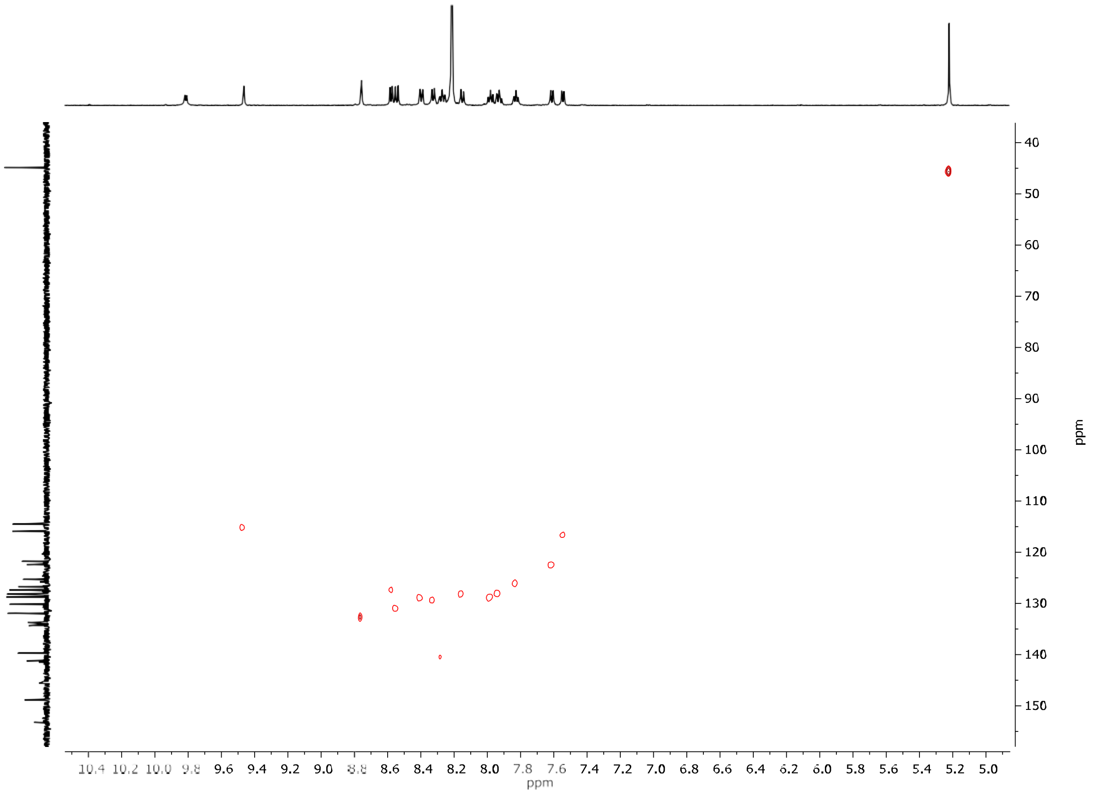

Platinum(II) complex **Aurkine 18**. ^{1}H NMR

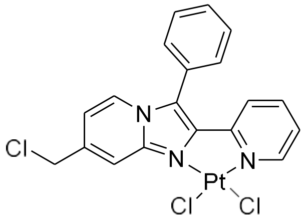

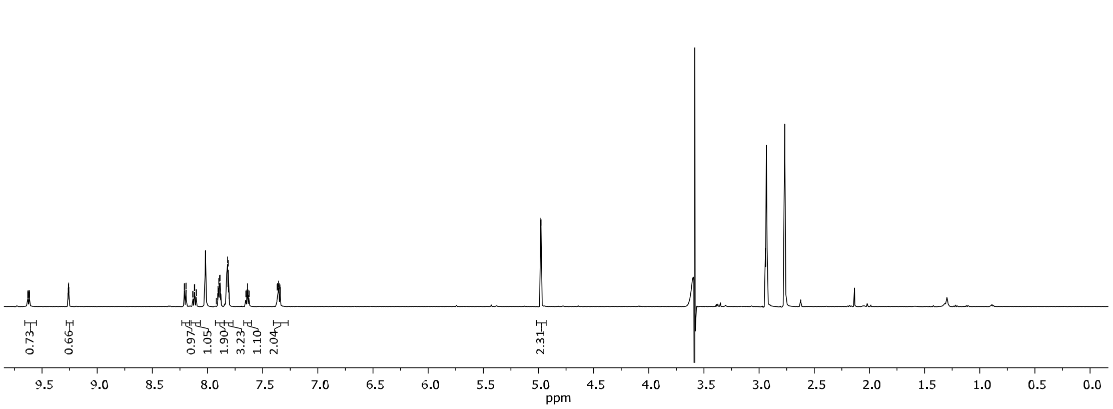

^{13}C NMR (DMF-d7)

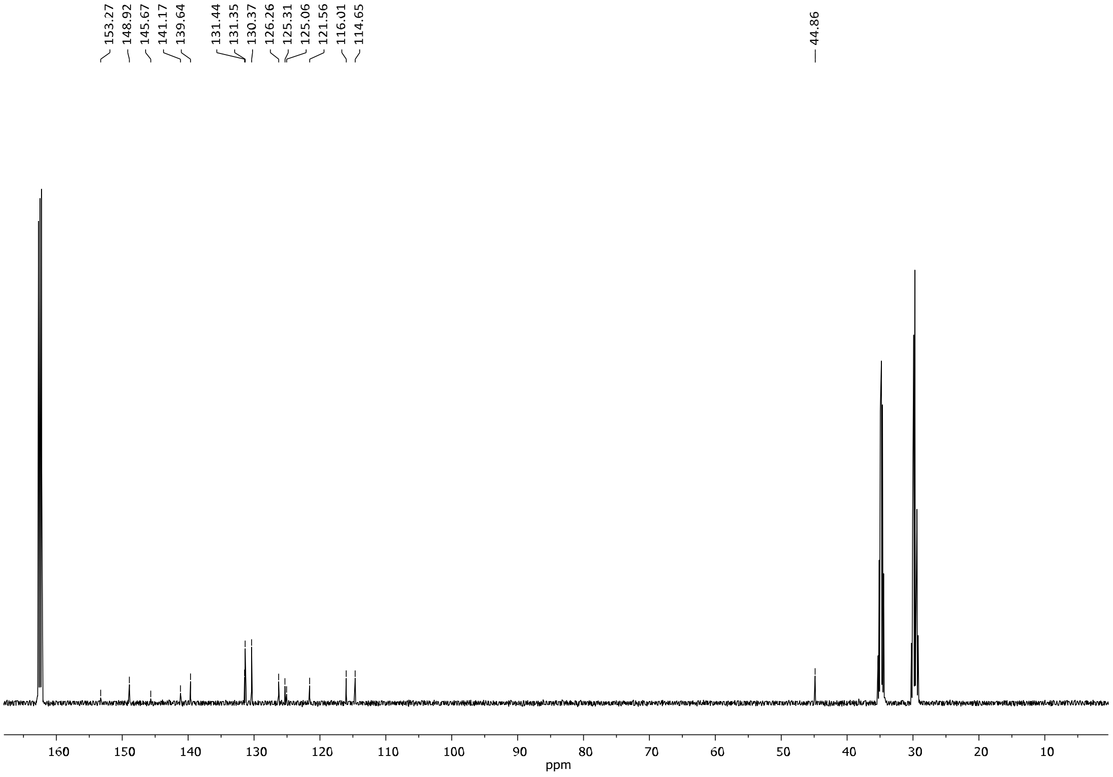

^{195}Pt-NMR (DMF-d7)

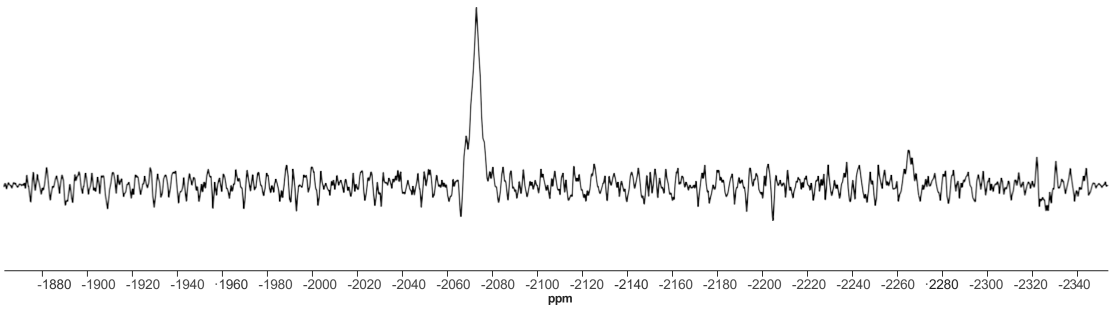

g-COSY (DMF-d7)

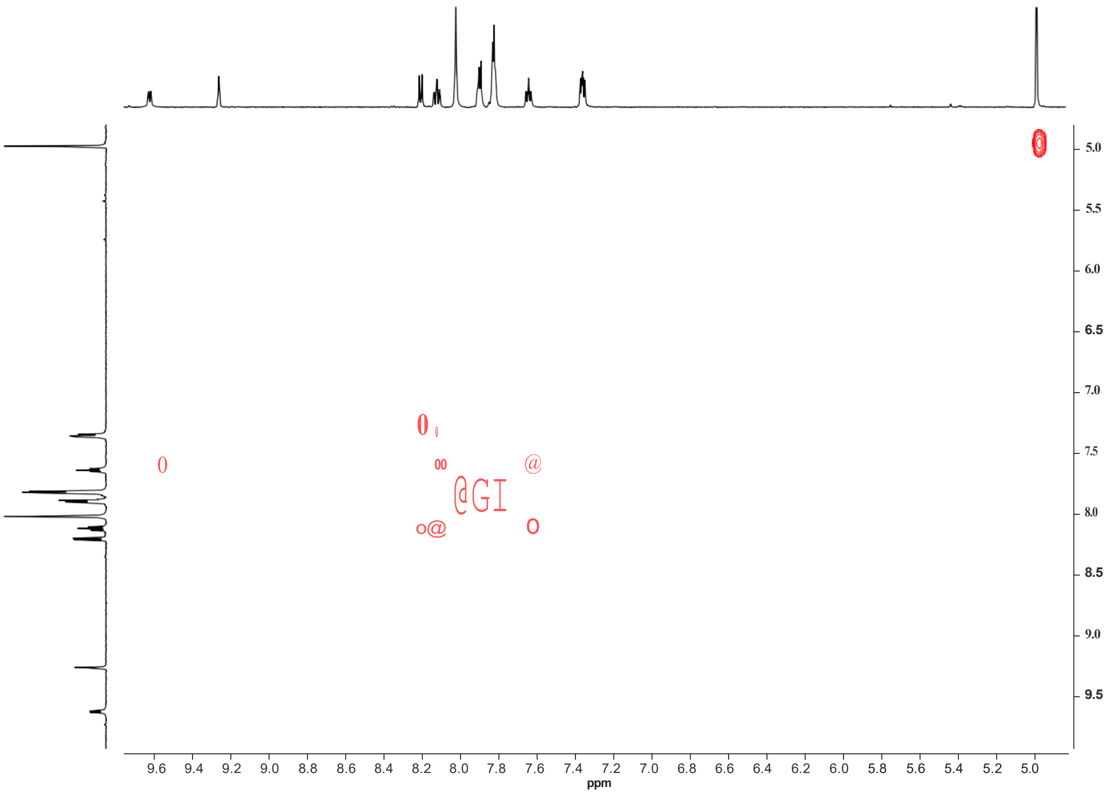

g-HSQC (DMF-d7)

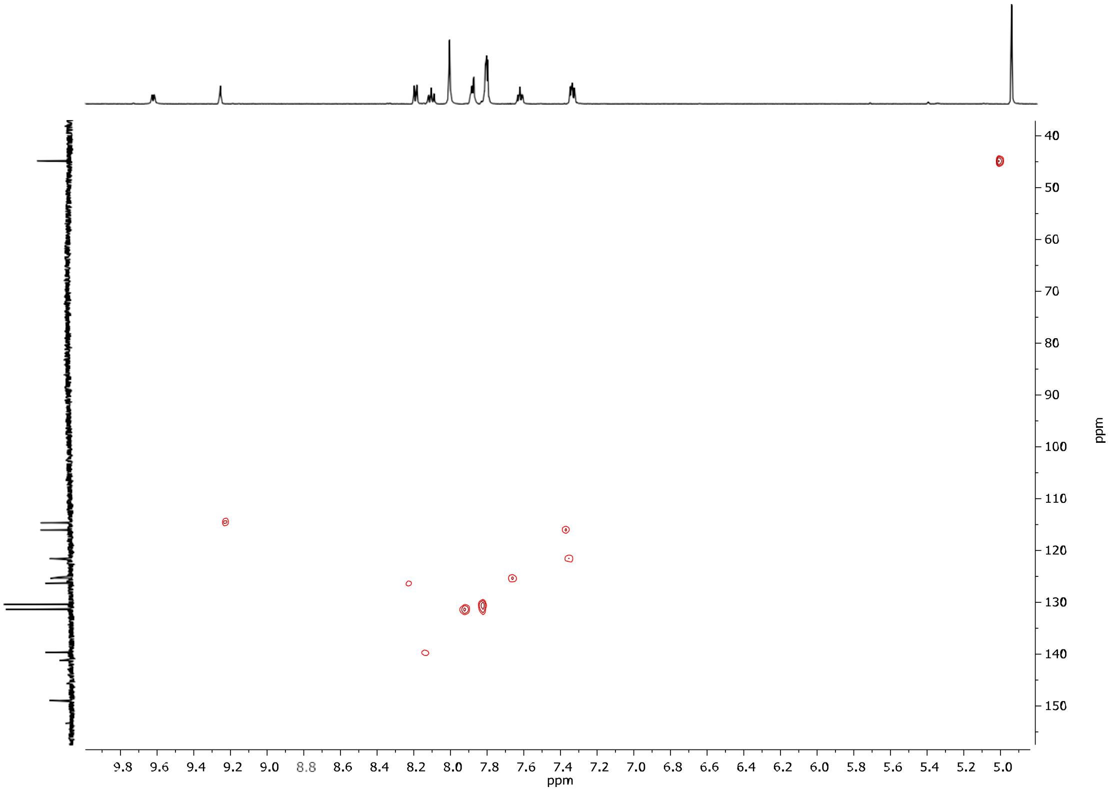
9.8 9.6 9.4 9.2 9.0 8.8 8.6 8.4 8.2 8.0 7.8 7.6 7.4 7.2 7.0 6.8 6.6 6.4 6.2 6.0 5.8 5.6 5.4 5.2 5.0
ppm
40 50 60 70 80 90 100 110 120 130 140 150
ppm

Computational Details

Molecular Dynamics calculations

We have run classical MD simulations of B-DNA in presence of the Aurki-Pt to identify potential binding sites. The starting structure contains three molecules of Aurki-Pt placed 15 Å from the DNA helical axes.[5,6] The B-DNA duplex, d(GCACGAACGGACGAACGC), was chosen as a model system. The topologies and coordinate files were generated by means of tleap in Amber using the OL15[7] and General Amber Force Field (GAFF) [8] together with the Metal Center Parameter Builder, MCPB.py.[9] The ligands were optimized and their charges were computed via the restrained electrostatic potential (RESP) using B3LYP/def2svp[10] as model chemistry. The required quantum mechanics calculations were carried out with the Gaussian16[11] suite of programs. The TIP3P water model was used to fill an octahedral box with solvent with 10 Å buffer distance, in each direction, around the platinum-DNA complex, and sodium counterions were added to neutralise the system. An excess of ~150 mM of NaCl was added to reach the physiological conditions. In the MD simulations, the SHAKE[12] algorithm was applied to constrain all bonds involving hydrogens. Initial minimisation was performed for 1000 steps, applying the harmonic positional restraint from 10 to 1 kcal mol^{-1} $Å^{-2}$ at the residues that correspond to the DNA, gradually decreasing the positional restraint in 2 kcal mol^{-1} $Å^{-2}$. Final minimization was performed for 2500 steps with no restrains. The system was then heated up from 0 to 300 K for 1000 ps. Finally, 10 independent production simulations were run at constant temperature of 300 K for 1μs. In one of the replicates, one of the Aurki-Pt bound to the B-DNA at ~800 ns in the configuration shown in Supplementary Fig. 1a and remained in this pose until the end of the simulation. This simulation was then extended to 5μs. As shown in Supplementary Fig. 1b, binding is concomitant with distorsions in the B-DNA structure resulting from the ligand.

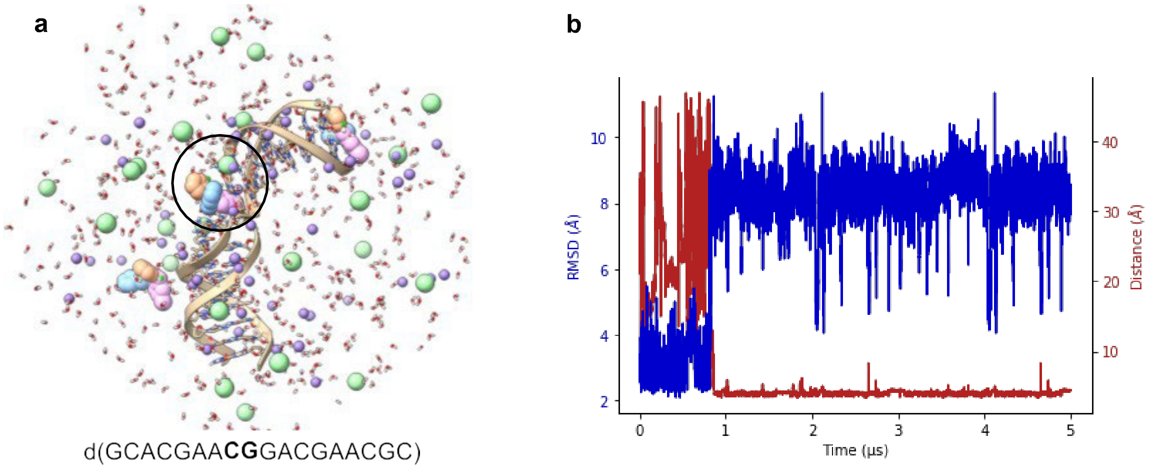

Snapshot from the Molecular Dynamacs trajectory, RMSD and Distance plots. (A) One molecule of Aurkine 16 intercalated between CpG base step, highlighted in black. Sodium and chloride ions are represented by purple and light green spheres, respectively. Some water molecules have been removed for clarity. **(B)** Time series data for the RMSD (blue) with respect to the first simulation frame and the distance (red) between the Pt in Aurkine 16 and the N7 atom of the guanine residue in the intercalation site.

<u>Calculation of the QM reaction profiles</u>

QM calculations of the stationary points were carried out within DFT framework. Hybrid exchange–correlation functional, B3LYP[13] was chosen. 6-31+G**[14,15] diffuse polarized basis set and LANL2DZ[16]] basis set and effective core potential (ECP) were applied to represent the main-group elements and platinum metallic centre, respectively. Continuum QM treatment of the solvent was applied within the Polarisation Continuum Model.[17] Grimme's D3[18] dispersion and Baker & Johnson (BJ) damping functions[19]] were considered.

Stationary Points

Total electronic energies (E, in hartree), zero point vibrational energies (ZPVE, in hartree), Thermal corrections to Gibbs Free Energies (TCGFE, in hartree), and number of imaginary frequencies (NIMAG) of all the stationary points. When NIMAG=1 (transition structures, TSs), the corresponding imaginary frequencies are given in cm^{-1}.

	E	ZPVE	TCGFE	NIMAG(ϖ)
**Aurkine 16**	-2703.3525391	0.388676	0.320759	0
**TS associated with aqua activation 1**	-2779.7756843	0.413692	0.344135	1(-159.6085)
**9**	-2319.3994416	0.413969	0.345818	0
**TS1**	-2862.0164682	0.530986	0.452404	1(-136.9456)
**10**	-2785.6266258	0.505976	0.427429	0
**TS associated with aqua activation 2**	-2862.0513489	0.531466	0.454203	1(-151.9801)
**11**	-2401.6690934	0.532375	0.457127	0
**TS2**	-2944.2829486	0.649964	0.562754	1(-116.7874)
**12**	-2867.8996496	0.625421	0.539416	0
**TS3**	-3410.5370465	0.742966	0.648797	1(-331.4051)
**13**	-2950.2972708	0.740203	0.644934	0

Optimized Cartesian coordinates of all the stationary points.

H_2O

```
---------------------------------------------------------------------
 Center     Atomic      Atomic             Coordinates (Angstroms)
 Number     Number       Type             X           Y           Z
---------------------------------------------------------------------
   1          8           0        0.000000   -0.000000    0.117678
   2          1           0       -0.000000    0.766834   -0.470711
   3          1           0       -0.000000   -0.766834   -0.470711
---------------------------------------------------------------------
```

Guanine

```
---------------------------------------------------------------------
 Center     Atomic      Atomic             Coordinates (Angstroms)
 Number     Number       Type             X           Y           Z
---------------------------------------------------------------------
   1          6           0        0.227363    1.448219    0.000688
   2          6           0       -0.844022    0.503689    0.000272
   3          6           0       -0.532415   -0.860502   -0.000393
   4          7           0        0.688687   -1.442216    0.003579
```

5	6	0	1.680502	-0.569706	-0.000440
6	7	0	1.480669	0.792356	-0.000428
7	7	0	-1.743838	-1.499615	0.000157
8	6	0	-2.720274	-0.522397	0.001508
9	7	0	-2.217988	0.692498	0.001146
10	7	0	2.966516	-1.007773	-0.054962
11	8	0	0.183691	2.688464	0.000773
12	1	0	-3.771366	-0.772816	0.002026
13	1	0	-1.892522	-2.500171	0.002752
14	1	0	2.283463	1.412098	-0.019011
15	1	0	3.105251	-1.992184	0.125603
16	1	0	3.720397	-0.397210	0.226194

Aurkine 16

Center Number	Atomic Number	Atomic Type	Coordinates (Angstroms) X	Y	Z
1	78	0	2.148327	-0.991952	-0.424958
2	7	0	0.563853	0.269887	-0.281071
3	7	0	0.651024	-2.331275	-0.005609
4	6	0	-0.598994	-1.798750	0.142562
5	6	0	-0.645056	-0.359555	-0.037691
6	8	0	3.776742	-2.029809	2.669937
7	8	0	3.595157	0.792196	2.458183
8	6	0	-1.689370	-2.608536	0.463608
9	6	0	-1.661963	0.573150	0.030002
10	6	0	-1.499529	-3.977723	0.624855
11	6	0	-0.220332	-4.509435	0.459640
12	6	0	0.831771	-3.656709	0.147949
13	7	0	-1.034348	1.800470	-0.165343
14	6	0	-1.589798	3.059112	-0.220235
15	6	0	0.331537	1.591742	-0.354125
16	6	0	-0.778197	4.132068	-0.436095
17	6	0	0.628861	3.959234	-0.610977
18	6	0	1.177221	2.695319	-0.572516
19	6	0	1.502552	5.151860	-0.839448
20	6	0	-3.110590	0.449957	0.244960
21	6	0	-3.707869	1.007710	1.412949
22	6	0	-3.895461	-0.214395	-0.680876
23	6	0	-5.060797	0.880218	1.628238
24	6	0	-5.291385	-0.366978	-0.478714
25	6	0	-5.888644	0.192449	0.698371
26	6	0	-7.287150	0.038994	0.899121
27	6	0	-6.113175	-1.057831	-1.411467
28	6	0	-8.058613	-0.637459	-0.022493
29	6	0	-7.466973	-1.190626	-1.187625
30	17	0	1.762688	6.078251	0.729378
31	1	0	3.841151	-2.296878	1.732610
32	1	0	4.583784	-2.357342	3.089462
33	1	0	3.642572	0.901531	1.494174
34	1	0	3.650140	-0.177025	2.586209
35	1	0	2.234477	2.512969	-0.708457

36	1	0	-1.216986	5.121828	-0.476527
37	1	0	-2.661255	3.116328	-0.089176
38	1	0	-2.338496	-4.617177	0.875978
39	1	0	-2.667360	-2.163239	0.584474
40	1	0	1.844143	-4.012943	0.015580
41	1	0	-0.026712	-5.569365	0.572630
42	1	0	-5.511951	1.300396	2.522365
43	1	0	-3.083537	1.518762	2.138899
44	1	0	-3.443146	-0.630539	-1.576334
45	1	0	-5.655523	-1.479550	-2.301805
46	1	0	-8.087409	-1.720391	-1.903753
47	1	0	-9.126023	-0.749774	0.140613
48	1	0	-7.738250	0.462898	1.791758
49	1	0	2.494221	4.871850	-1.188467
50	1	0	1.050269	5.868820	-1.523657
51	17	0	3.873669	-2.653117	-0.530370
52	17	0	3.831830	0.638134	-0.898455

TS associated with aqua activation 1

Center Number	Atomic Number	Atomic Type	Coordinates (Angstroms) X	Y	Z
1	78	0	1.885739	-1.036378	-0.031946
2	17	0	3.637847	-2.673302	0.006281
3	7	0	0.312916	0.213014	-0.039606
4	7	0	0.370360	-2.364018	0.249589
5	6	0	-0.891522	-1.831003	0.300452
6	6	0	-0.919199	-0.392869	0.122225
7	17	0	3.309975	0.416121	-1.979462
8	8	0	3.366607	0.675801	1.006576
9	8	0	5.546628	-0.882948	1.664170
10	8	0	6.423842	-0.028615	-0.970866
11	6	0	-2.001759	-2.646078	0.522307
12	6	0	-1.918228	0.562634	0.114860
13	6	0	-1.824918	-4.016828	0.682989
14	6	0	-0.535491	-4.546004	0.615130
15	6	0	0.539602	-3.692904	0.400201
16	7	0	-1.250948	1.775342	-0.048502
17	6	0	-1.768654	3.047298	-0.156672
18	6	0	0.118759	1.536383	-0.144990
19	6	0	-0.916400	4.095436	-0.335365
20	6	0	0.494555	3.887007	-0.425219
21	6	0	1.006134	2.610867	-0.333434
22	6	0	1.408259	5.053919	-0.627079
23	6	0	-3.379031	0.470250	0.239747
24	6	0	-4.038559	1.073568	1.350385
25	6	0	-4.115979	-0.211367	-0.712649
26	6	0	-5.404128	0.973684	1.483879
27	6	0	-5.524242	-0.336597	-0.593809
28	6	0	-6.184121	0.269078	0.525593
29	6	0	-7.594801	0.143511	0.642726
30	6	0	-6.298141	-1.044094	-1.554641
31	6	0	-8.318884	-0.550083	-0.304266
32	6	0	-7.665368	-1.148962	-1.412277

33	17	0	1.611103	5.999765	0.938374
34	1	0	4.116705	0.198185	1.423531
35	1	0	3.710060	0.974833	0.143194
36	1	0	5.100101	-1.708292	1.387206
37	1	0	5.996650	-1.060234	2.501158
38	1	0	5.528471	0.035672	-1.352028
39	1	0	6.280439	-0.316871	-0.051860
40	1	0	2.062130	2.403935	-0.424521
41	1	0	-1.326563	5.094865	-0.416978
42	1	0	-2.844359	3.134272	-0.096359
43	1	0	-2.680562	-4.659516	0.857140
44	1	0	-2.986205	-2.200123	0.566911
45	1	0	1.558683	-4.049355	0.347776
46	1	0	-0.350357	-5.607306	0.729689
47	1	0	-5.902595	1.428806	2.334716
48	1	0	-3.452297	1.596990	2.098877
49	1	0	-3.616139	-0.660728	-1.565798
50	1	0	-5.793484	-1.500965	-2.401127
51	1	0	-8.248984	-1.691616	-2.149396
52	1	0	-9.396159	-0.640782	-0.205191
53	1	0	-8.093420	0.602408	1.491715
54	1	0	2.409210	4.744452	-0.920405
55	1	0	1.009058	5.769627	-1.344705

9

Center Number	Atomic Number	Atomic Type	Coordinates (Angstroms) X	Y	Z
1	78	0	1.998458	-1.072398	-0.171743
2	7	0	0.453789	0.213765	-0.077455
3	7	0	0.497185	-2.359206	0.253741
4	6	0	-0.755516	-1.810649	0.346482
5	6	0	-0.778489	-0.376330	0.127819
6	8	0	5.450704	-0.331658	0.935248
7	8	0	8.050453	0.148142	-0.161786
8	6	0	-1.859479	-2.605345	0.652650
9	6	0	-1.768849	0.587597	0.117965
10	6	0	-1.682884	-3.970505	0.859571
11	6	0	-0.402805	-4.515270	0.754461
12	6	0	0.666284	-3.680872	0.452066
13	7	0	-1.093311	1.791132	-0.086510
14	6	0	-1.597735	3.066258	-0.214487
15	6	0	0.271184	1.536266	-0.203814
16	6	0	-0.735687	4.099713	-0.430222
17	6	0	0.672189	3.874899	-0.530307
18	6	0	1.171747	2.595001	-0.420300
19	6	0	1.594970	5.030172	-0.759801
20	6	0	-3.229168	0.508269	0.250798
21	6	0	-3.887324	1.159918	1.334422
22	6	0	-3.968230	-0.204036	-0.677648
23	6	0	-5.254644	1.079257	1.463700
24	6	0	-5.378091	-0.309222	-0.562497
25	6	0	-6.037328	0.347719	0.528001
26	6	0	-7.450328	0.245140	0.639229
27	6	0	-6.154375	-1.043436	-1.501155
28	6	0	-8.176870	-0.474656	-0.286090
29	6	0	-7.523785	-1.124574	-1.365244
30	17	0	1.807527	6.007647	0.783143
31	1	0	5.187725	-1.272408	0.879001

32	1	0	5.383862	-0.079006	1.867934
33	1	0	7.903501	0.545151	-1.029820
34	1	0	7.160038	-0.014392	0.202683
35	1	0	2.226899	2.367688	-0.495789
36	1	0	-1.135783	5.101753	-0.528011
37	1	0	-2.671092	3.168617	-0.138420
38	1	0	-2.533790	-4.597819	1.100508
39	1	0	-2.837836	-2.150105	0.727524
40	1	0	1.679437	-4.047539	0.363311
41	1	0	-0.221289	-5.572381	0.905723
42	1	0	-5.752730	1.572073	2.293453
43	1	0	-3.299700	1.705300	2.065932
44	1	0	-3.469115	-0.690108	-1.510827
45	1	0	-5.649889	-1.538847	-2.325775
46	1	0	-8.109526	-1.687247	-2.085461
47	1	0	-9.255992	-0.546806	-0.192182
48	1	0	-7.948922	0.743188	1.465828
49	1	0	2.592568	4.706450	-1.049099
50	1	0	1.198936	5.731793	-1.493053
51	17	0	3.759020	-2.699863	-0.274801
52	8	0	3.452444	0.408293	-0.620554
53	1	0	4.252144	0.255682	-0.035777
54	1	0	3.748375	0.303735	-1.538812

TS1

Center Number	Atomic Number	Atomic Type	Coordinates (Angstroms) X	Y	Z
1	6	0	-1.314004	0.664287	2.555140
2	6	0	-2.548192	0.528451	1.844100
3	6	0	-3.494288	1.552834	1.948034
4	7	0	-3.401374	2.715745	2.625754
5	6	0	-2.248383	2.860342	3.258691
6	7	0	-1.258804	1.905024	3.231844
7	7	0	-4.552348	1.145199	1.172555
8	6	0	-4.219677	-0.067170	0.617637
9	7	0	-3.023142	-0.465159	0.995736
10	78	0	-1.479168	-1.940390	-0.235506
11	7	0	-2.027116	3.968255	4.006829
12	8	0	-0.357838	-0.117604	2.637367
13	7	0	-0.182293	-0.433511	-0.389193
14	6	0	1.132260	-0.736834	-0.104788
15	6	0	1.881132	0.426761	-0.076246
16	7	0	0.968727	1.447085	-0.344827
17	6	0	-0.292196	0.890422	-0.542465
18	6	0	1.169439	2.806422	-0.444420
19	6	0	0.104943	3.614081	-0.715281
20	6	0	-1.201654	3.070164	-0.919801
21	6	0	-1.393990	1.708466	-0.844779
22	6	0	3.301468	0.684155	0.191079
23	6	0	1.392749	-2.136478	0.171851
24	7	0	0.266138	-2.919496	0.118158
25	6	0	0.361785	-4.242307	0.357640

26	6	0	1.576350	-4.842972	0.665214
27	6	0	2.728671	-4.058198	0.735344
28	6	0	2.633191	-2.692442	0.483747
29	6	0	-2.340992	3.975323	-1.254822
30	17	0	-2.262513	4.472105	-3.035878
31	17	0	-2.953338	-3.812795	-0.078491
32	6	0	4.273829	0.161775	-0.643156
33	6	0	5.651823	0.378962	-0.383910
34	6	0	6.666700	-0.157674	-1.223197
35	6	0	6.029795	1.165644	0.753420
36	6	0	5.009859	1.698040	1.589930
37	6	0	3.680850	1.465306	1.322091
38	6	0	7.997668	0.073063	-0.947575
39	6	0	8.372326	0.851115	0.178294
40	6	0	7.410161	1.385367	1.009642
41	8	0	-3.016293	-1.059478	-1.884313
42	1	0	-0.563427	-4.799743	0.299542
43	1	0	1.605615	-5.910623	0.847622
44	1	0	3.687484	-4.501265	0.980239
45	1	0	3.505101	-2.053353	0.527238
46	1	0	2.181073	3.156476	-0.295267
47	1	0	-2.352996	1.239521	-1.025900
48	1	0	0.266445	4.682982	-0.786567
49	1	0	-4.869507	-0.589389	-0.065076
50	1	0	-5.414214	1.654903	1.025399
51	1	0	-0.405602	2.056288	3.759363
52	1	0	-2.685578	4.726115	3.896424
53	1	0	-1.093698	4.218003	4.299489
54	1	0	-3.307241	3.492179	-1.130463
55	1	0	-2.308255	4.913030	-0.702857
56	1	0	3.985806	-0.422203	-1.512520
57	1	0	2.912669	1.858427	1.980450
58	1	0	5.295582	2.288330	2.455651
59	1	0	7.694991	1.980871	1.872360
60	1	0	9.424264	1.024619	0.383010
61	1	0	6.375171	-0.752833	-2.083876
62	1	0	8.766385	-0.340806	-1.592708
63	8	0	-0.309570	1.944323	-4.091698
64	1	0	-0.801934	2.746831	-3.851956
65	1	0	0.633298	2.106136	-3.953931
66	8	0	-1.483741	-0.427290	-3.897859
67	1	0	-1.029431	0.449973	-3.980365
68	1	0	-1.505271	-0.864321	-4.758125
69	1	0	-2.461761	-0.846116	-2.690612
70	1	0	-3.609139	-1.794944	-2.097307

10

Center Number	Atomic Number	Atomic Type	Coordinates (Angstroms) X	Y	Z
1	6	0	6.581047	1.135461	0.119775
2	6	0	5.928492	0.422754	1.178857
3	6	0	7.994229	1.281244	0.157840
4	6	0	8.727497	0.746001	1.196042
5	6	0	6.711547	-0.116737	2.236365

6	6	0	8.081048	0.041145	2.244243
7	6	0	5.792613	1.671613	-0.935714
8	6	0	4.517494	0.277324	1.142931
9	6	0	4.426337	1.510612	-0.951220
10	6	0	3.775347	0.799935	0.098934
11	6	0	2.317230	0.618285	0.065330
12	6	0	1.516937	-0.499740	-0.075932
13	7	0	1.427331	1.684223	0.181001
14	7	0	0.183743	-0.132428	-0.041794
15	6	0	0.123135	1.197083	0.122895
16	6	0	1.691523	3.023233	0.352766
17	6	0	0.649750	3.898493	0.441563
18	6	0	-0.698604	3.434680	0.380955
19	6	0	-0.955872	2.088613	0.239893
20	6	0	-1.830525	4.409263	0.406928
21	17	0	-2.162239	5.009420	-1.309961
22	6	0	1.786413	-1.913922	-0.275353
23	6	0	2.002381	-4.636397	-0.646874
24	6	0	3.053048	-2.492365	-0.360211
25	6	0	3.162488	-3.867565	-0.549269
26	6	0	0.765813	-4.006160	-0.565396
27	7	0	0.657027	-2.677004	-0.387851
28	78	0	-1.126001	-1.645245	-0.338606
29	17	0	-2.534441	-3.533875	-0.723816
30	7	0	-4.471485	0.741401	-1.104423
31	6	0	-3.328988	0.068922	-1.429626
32	7	0	-2.821582	-0.512089	-0.352811
33	6	0	-3.664204	-0.216330	0.711891
34	6	0	-3.606399	-0.546395	2.103945
35	8	0	-2.771515	-1.211348	2.719199
36	7	0	-4.706250	0.022537	2.786948
37	6	0	-5.697987	0.798003	2.229398
38	7	0	-6.644694	1.288593	3.056672
39	7	0	-5.735487	1.099762	0.938935
40	6	0	-4.708771	0.579891	0.241308
41	1	0	8.487571	1.820752	-0.645626
42	1	0	9.806670	0.862475	1.215444
43	1	0	6.212333	-0.655579	3.036599
44	1	0	8.671894	-0.374402	3.054577
45	1	0	6.286877	2.207891	-1.740459
46	1	0	4.020080	-0.254576	1.948777
47	1	0	3.836245	1.908696	-1.770563
48	1	0	2.734148	3.303382	0.407674
49	1	0	0.860222	4.954125	0.563734
50	1	0	-1.964039	1.706590	0.207555
51	1	0	-1.607574	5.303363	0.985940
52	1	0	-2.762473	3.958151	0.741351
53	1	0	3.932733	-1.868610	-0.277458
54	1	0	4.140965	-4.329987	-0.617603
55	1	0	2.043315	-5.709601	-0.789020
56	1	0	-0.167429	-4.547889	-0.647002
57	1	0	-2.874391	0.064164	-2.414075
58	1	0	-4.726397	-0.170061	3.782812
59	1	0	-6.749879	0.951213	4.001698
60	1	0	-7.436210	1.751804	2.634601
61	1	0	-5.040924	1.275992	-1.748425
62	8	0	-0.051472	2.636520	-2.855933
63	1	0	0.541522	3.073857	-3.482304
64	1	0	-0.648019	3.332069	-2.536306
65	8	0	-1.347978	0.347052	-3.802844
66	1	0	-0.888292	1.162898	-3.508325
67	1	0	-1.567936	0.493674	-4.731815

TS associated with aqua activation 2

Center Number	Atomic Number	Atomic Type	X	Y	Z
1	8	0	0.694013	4.923089	1.314687
2	8	0	-0.691204	2.565186	2.067627
3	8	0	2.358611	-3.181395	1.621420
4	17	0	2.327582	-4.381482	-1.071598
5	7	0	4.873346	-0.359302	-0.976402
6	6	0	3.861258	-1.259533	-1.134304
7	7	0	2.863686	-0.982175	-0.309835
8	6	0	3.242480	0.138426	0.423946
9	6	0	2.571155	0.901103	1.427704
10	7	0	3.355427	1.999699	1.825890
11	6	0	4.607336	2.316996	1.346502
12	7	0	5.227384	1.593440	0.423871
13	6	0	4.507950	0.542387	-0.002445
14	8	0	1.459100	0.712144	1.934832
15	7	0	5.197303	3.428063	1.828745
16	1	0	1.155456	5.332689	2.059149
17	1	0	1.380102	4.756970	0.647465
18	1	0	-0.244046	3.389884	1.794071
19	1	0	0.017420	1.895992	2.066952
20	1	0	2.583226	-3.952400	1.058638
21	1	0	1.795962	-3.504017	2.340357
22	1	0	3.879826	-2.087102	-1.825287
23	1	0	5.744159	-0.356540	-1.492949
24	1	0	4.849773	3.905085	2.646904
25	1	0	6.153115	3.603053	1.554857
26	1	0	2.918617	2.600033	2.517990
27	78	0	1.110372	-2.004346	-0.113864
28	7	0	-0.124790	-0.504871	-0.629877
29	7	0	-0.699221	-2.879354	0.222294
30	6	0	-1.776222	-2.035064	0.203064
31	6	0	-1.442959	-0.695783	-0.250281
32	6	0	-3.042028	-2.490430	0.570776
33	6	0	-2.144923	0.486378	-0.395703
34	6	0	-3.204432	-3.824425	0.937360
35	6	0	-2.102873	-4.681561	0.915087
36	6	0	-0.860717	-4.172984	0.553469
37	7	0	-1.214521	1.399569	-0.885432
38	6	0	-1.382113	2.725411	-1.205953
39	6	0	0.015772	0.764801	-1.037339
40	6	0	-0.318704	3.432692	-1.685026
41	6	0	0.946857	2.803788	-1.885491
42	6	0	1.103855	1.469663	-1.575780
43	6	0	2.101385	3.587166	-2.421902
44	6	0	-3.531544	0.864268	-0.089293
45	6	0	-3.776037	1.809377	0.950702
46	6	0	-4.587221	0.307956	-0.788248
47	6	0	-5.067811	2.160505	1.268817
48	6	0	-5.928059	0.649950	-0.472297
49	6	0	-6.175159	1.595859	0.576518

50	6	0	-7.518462	1.936678	0.891531
51	6	0	-7.031203	0.082382	-1.167619
52	6	0	-8.568448	1.368337	0.201009
53	6	0	-8.323062	0.433293	-0.837649
54	17	0	3.052848	4.361282	-1.040387
55	1	0	2.035253	0.951808	-1.748645
56	1	0	-0.453306	4.479773	-1.927888
57	1	0	-2.371257	3.133974	-1.055933
58	1	0	-4.182753	-4.191152	1.227638
59	1	0	-3.880401	-1.806993	0.562747
60	1	0	0.030131	-4.786865	0.510053
61	1	0	-2.193905	-5.729130	1.175176
62	1	0	-5.257205	2.874417	2.065501
63	1	0	-2.927147	2.224530	1.486691
64	1	0	-4.397616	-0.399532	-1.590367
65	1	0	-6.838495	-0.633051	-1.962067
66	1	0	-9.159574	-0.005510	-1.372603
67	1	0	-9.590724	1.635498	0.450560
68	1	0	-7.704526	2.652707	1.687043
69	1	0	2.822051	2.959730	-2.942152
70	1	0	1.788684	4.415066	-3.054985

11

Center Number	Atomic Number	Atomic Type	Coordinates (Angstroms) X	Y	Z
1	6	0	-6.601469	0.834888	0.050361
2	6	0	-6.000970	0.075768	-1.007384
3	6	0	-8.018668	0.913808	0.120538
4	6	0	-8.804975	0.271218	-0.812649
5	6	0	-6.837948	-0.573695	-1.956097
6	6	0	-8.209926	-0.478643	-1.860053
7	6	0	-5.759046	1.483036	0.995639
8	6	0	-4.586112	-0.006600	-1.077596
9	6	0	-4.389428	1.383323	0.912040
10	6	0	-3.792518	0.621067	-0.134540
11	6	0	-2.329133	0.498447	-0.193945
12	6	0	-1.475871	-0.574820	-0.036656
13	7	0	-1.492801	1.589708	-0.430597
14	7	0	-0.160880	-0.151289	-0.155277
15	6	0	-0.167830	1.163409	-0.432957
16	6	0	-1.827932	2.902169	-0.669759
17	6	0	-0.835772	3.806908	-0.910938
18	6	0	0.529883	3.395843	-0.963201
19	6	0	0.855081	2.074415	-0.743709
20	6	0	1.607550	4.393627	-1.248089
21	17	0	2.116387	5.232568	0.306509
22	6	0	-1.658722	-2.000096	0.169246
23	6	0	-1.693535	-4.748213	0.383531
24	6	0	-2.882076	-2.659227	0.284276
25	6	0	-2.899562	-4.046561	0.399182
26	6	0	-0.504173	-4.036282	0.288647

27	7	0	-0.480828	-2.694899	0.201099
28	78	0	1.217233	-1.530560	0.283941
29	7	0	4.010850	1.481667	1.134907
30	6	0	2.842138	0.800482	1.301547
31	7	0	2.817810	-0.271846	0.521402
32	6	0	4.032410	-0.304204	-0.158970
33	6	0	4.637956	-1.287729	-0.987029
34	8	0	4.210471	-2.423737	-1.284069
35	7	0	5.885351	-0.868620	-1.453007
36	6	0	6.523098	0.301949	-1.090164
37	7	0	7.729843	0.544631	-1.627718
38	7	0	6.001807	1.162865	-0.223764
39	6	0	4.783613	0.812795	0.212865
40	8	0	2.678818	-3.005929	0.716730
41	1	0	-8.472465	1.487968	0.923274
42	1	0	-9.886730	0.337049	-0.749107
43	1	0	-6.377915	-1.147197	-2.755755
44	1	0	-8.842113	-0.978538	-2.587249
45	1	0	-6.214510	2.057967	1.796814
46	1	0	-4.129661	-0.573699	-1.883588
47	1	0	-3.748331	1.869558	1.640209
48	1	0	-2.882653	3.137820	-0.656424
49	1	0	-1.103099	4.841393	-1.089781
50	1	0	1.870908	1.721085	-0.833616
51	1	0	1.274005	5.186841	-1.914811
52	1	0	2.507519	3.923385	-1.639396
53	1	0	-3.800237	-2.087797	0.275788
54	1	0	-3.843638	-4.571989	0.489447
55	1	0	-1.662658	-5.828815	0.448761
56	1	0	0.449213	-4.545726	0.274754
57	1	0	2.032737	1.101811	1.959386
58	1	0	6.366074	-1.537787	-2.046238
59	1	0	8.124612	-0.025262	-2.360419
60	1	0	8.182922	1.418196	-1.403191
61	1	0	4.259609	2.340316	1.610951
62	1	0	3.283213	-2.988352	-0.092535
63	1	0	2.398330	-3.915045	0.888785
64	8	0	-1.708954	2.757882	2.598017
65	1	0	-2.096541	3.042156	3.438149
66	1	0	-1.353138	3.564035	2.197090
67	8	0	0.294721	0.854404	3.017801
68	1	0	-0.436763	1.489382	2.865548
69	1	0	-0.016365	0.002331	2.683139

TS2

Center Number	Atomic Number	Atomic Type	Coordinates (Angstroms) X	Y	Z
1	6	0	3.924239	-3.443529	-0.507953
2	6	0	3.365185	-2.852324	0.658481
3	6	0	3.703733	-3.411553	1.899400

4	7	0	4.541751	-4.434308	2.153395
5	6	0	5.076407	-4.962531	1.064379
6	7	0	4.786539	-4.502577	-0.201118
7	7	0	2.989741	-2.695827	2.823500
8	6	0	2.252730	-1.751717	2.149054
9	7	0	2.456750	-1.811127	0.847484
10	78	0	1.003466	-0.278685	-0.423048
11	7	0	5.910807	-6.021537	1.165415
12	8	0	3.728353	-3.152598	-1.710664
13	7	0	-0.829920	0.541327	-0.376003
14	6	0	-1.870102	-0.353696	-0.566124
15	6	0	-3.081131	0.297288	-0.419654
16	7	0	-2.746802	1.621462	-0.150311
17	6	0	-1.358932	1.749895	-0.134886
18	6	0	-3.570002	2.686785	0.136882
19	6	0	-3.014949	3.903200	0.403444
20	6	0	-1.597753	4.082073	0.376406
21	6	0	-0.779880	3.007541	0.105667
22	6	0	-4.468996	-0.173675	-0.499506
23	6	0	-1.482133	-1.697847	-0.951697
24	7	0	-0.127006	-1.872777	-1.002986
25	6	0	0.385136	-3.047345	-1.412135
26	6	0	-0.431684	-4.112503	-1.771820
27	6	0	-1.817299	-3.954942	-1.710797
28	6	0	-2.347925	-2.734865	-1.300812
29	6	0	-1.002557	5.423348	0.664325
30	17	0	-0.889148	5.696234	2.481380
31	6	0	-4.919142	-1.156222	0.365152
32	6	0	-6.248391	-1.645212	0.285954
33	6	0	-6.726479	-2.661952	1.157843
34	6	0	-7.134042	-1.095857	-0.698508
35	6	0	-6.648885	-0.080369	-1.568344
36	6	0	-5.352760	0.371123	-1.476856
37	6	0	-8.024226	-3.116261	1.057962
38	6	0	-8.900974	-2.573347	0.083304
39	6	0	-8.465849	-1.585346	-0.775068
40	1	0	1.463537	-3.113054	-1.460249
41	1	0	0.020706	-5.042056	-2.095435
42	1	0	-2.478716	-4.768870	-1.986237
43	1	0	-3.417115	-2.577955	-1.256360
44	1	0	-4.632257	2.487156	0.132183
45	1	0	0.292580	3.111584	0.067318
46	1	0	-3.666990	4.738047	0.630324
47	1	0	1.589041	-1.060051	2.657237
48	1	0	2.998728	-2.840144	3.825141
49	1	0	5.199357	-4.960475	-1.007231
50	1	0	6.228040	-6.264091	2.093096
51	1	0	6.503100	-6.293610	0.394400
52	1	0	0.014936	5.510847	0.288170
53	1	0	-1.616778	6.239197	0.286865
54	1	0	-4.251289	-1.565739	1.117522
55	1	0	-4.989144	1.131585	-2.160587
56	1	0	-7.317085	0.331996	-2.318636
57	1	0	-9.135530	-1.168789	-1.522032
58	1	0	-9.920259	-2.940892	0.016275
59	1	0	-6.051638	-3.074357	1.902473
60	1	0	-8.381487	-3.893307	1.726497
61	6	0	2.779232	2.501143	-2.032328

62	6	0	2.630857	2.356798	-0.618387
63	6	0	3.126771	3.366160	0.212644
64	7	0	3.761872	4.496095	-0.139693
65	6	0	3.891642	4.641458	-1.451276
66	7	0	3.425573	3.707899	-2.350454
67	7	0	2.817417	2.975089	1.493729
68	6	0	2.152116	1.785575	1.438899
69	7	0	2.027191	1.386731	0.181705
70	7	0	4.473174	5.750688	-1.947567
71	8	0	2.430970	1.736209	-2.948131
72	1	0	1.727551	1.286009	2.304489
73	1	0	3.027132	3.491417	2.338956
74	1	0	3.548597	3.866824	-3.345231
75	1	0	4.906651	6.383705	-1.291323
76	1	0	4.738183	5.826329	-2.918315
77	8	0	3.011074	-0.616225	-1.740009
78	1	0	2.859270	-0.036776	-2.509148
79	1	0	3.272551	-1.539802	-1.962980
80	8	0	-2.046609	1.086417	2.877752
81	8	0	0.464876	0.393620	3.581161
82	1	0	0.562351	0.435455	4.541728
83	1	0	-0.465507	0.635435	3.354184
84	1	0	-2.383243	1.968508	3.088790
85	1	0	-2.782766	0.462241	2.940045

12

Center Number	Atomic Number	Atomic Type	Coordinates (Angstroms) X	Y	Z
1	6	0	7.127114	-0.951191	0.236858
2	6	0	6.163158	-1.463940	1.166014
3	6	0	8.481702	-1.364770	0.353212
4	6	0	8.864136	-2.245507	1.343209
5	6	0	6.588056	-2.369803	2.176674
6	6	0	7.909643	-2.752450	2.262666
7	6	0	6.695637	-0.046291	-0.772182
8	6	0	4.811029	-1.049461	1.053548
9	6	0	5.377046	0.334892	-0.864985
10	6	0	4.416489	-0.174469	0.056987
11	6	0	3.008209	0.228042	-0.055449
12	6	0	1.853239	-0.476784	-0.333459
13	7	0	2.588664	1.545900	0.127517
14	7	0	0.762714	0.376291	-0.311135
15	6	0	1.207603	1.611514	-0.034081
16	6	0	3.332001	2.658497	0.450076
17	6	0	2.701246	3.859494	0.591030
18	6	0	1.288928	3.965806	0.415600
19	6	0	0.550298	2.844985	0.104459
20	6	0	0.608056	5.289459	0.555156
21	17	0	0.742204	6.247569	-1.016289
22	6	0	1.577279	-1.859055	-0.689717
23	6	0	0.775688	-4.387295	-1.444042

24	6	0	2.535027	-2.861575	-0.842598
25	6	0	2.130398	-4.138687	-1.221447
26	6	0	-0.134335	-3.350223	-1.281737
27	7	0	0.252521	-2.117126	-0.908938
28	78	0	-1.019165	-0.524053	-0.651117
29	7	0	-2.740540	-1.591572	-0.977840
30	6	0	-3.318625	-2.537408	-0.134209
31	6	0	-3.563511	-1.442510	-2.005338
32	6	0	-4.526377	-2.953385	-0.693892
33	6	0	-2.864311	-3.149287	1.079410
34	7	0	-4.653180	-2.247032	-1.867585
35	7	0	-5.399977	-3.853811	-0.210530
36	7	0	-3.820842	-4.079662	1.546271
37	8	0	-1.811870	-2.979325	1.694096
38	6	0	-5.010154	-4.401523	0.932572
39	7	0	-5.780645	-5.341947	1.517517
40	7	0	-2.190346	1.138514	-0.389029
41	6	0	-2.596532	1.717167	0.811829
42	6	0	-2.720710	1.861483	-1.363838
43	6	0	-3.395868	2.823345	0.520772
44	6	0	-2.272321	1.431283	2.178223
45	7	0	-3.457770	2.884521	-0.852811
46	7	0	-3.976182	3.679112	1.379507
47	7	0	-2.918313	2.350797	3.036925
48	8	0	-1.543795	0.556520	2.646032
49	6	0	-3.717310	3.402984	2.651171
50	7	0	-4.269778	4.159256	3.620251
51	1	0	9.211104	-0.975737	-0.351430
52	1	0	9.901486	-2.555375	1.423366
53	1	0	5.853822	-2.754818	2.878528
54	1	0	8.226293	-3.444836	3.036430
55	1	0	7.424465	0.338366	-1.479511
56	1	0	4.081634	-1.428263	1.763485
57	1	0	5.056397	1.012248	-1.650082
58	1	0	4.394433	2.505345	0.577486
59	1	0	3.289049	4.734146	0.842487
60	1	0	-0.516178	2.895600	-0.043628
61	1	0	1.070440	5.919857	1.312561
62	1	0	-0.458475	5.186275	0.745068
63	1	0	3.578359	-2.635404	-0.669270
64	1	0	2.865131	-4.926526	-1.344590
65	1	0	0.418601	-5.365211	-1.743104
66	1	0	-1.191425	-3.493626	-1.453641
67	1	0	-3.409585	-0.789908	-2.848773
68	1	0	-3.555444	-4.560209	2.399858
69	1	0	-5.624581	-5.635059	2.470513
70	1	0	-6.701788	-5.489542	1.131045
71	1	0	-2.577716	1.698171	-2.418410
72	1	0	-2.740334	2.194582	4.023728
73	1	0	-3.981594	4.090060	4.584554
74	1	0	-4.766186	4.991321	3.336712
75	1	0	-3.939924	3.588997	-1.397070
76	1	0	-5.418703	-2.319908	-2.526551
77	8	0	-0.760376	3.814674	-2.828756
78	1	0	-1.204637	4.175511	-3.608604
79	1	0	-0.290295	4.565732	-2.430021
80	8	0	0.761272	1.527008	-3.457460
81	1	0	0.252875	2.327950	-3.215263

82	1	0	0.402450	0.824013	-2.898954

TS3

Center Number	Atomic Number	Atomic Type	Coordinates (Angstroms) X	Y	Z
1	7	0	-2.669937	2.450475	3.690859
2	7	0	-2.172149	0.567330	4.942766
3	7	0	-2.836123	4.406929	2.269329
4	6	0	-1.811960	1.499285	4.030211
5	6	0	-2.170076	3.326026	2.795401
6	6	0	-1.996511	5.022331	1.382063
7	7	0	-0.534577	1.443923	3.531685
8	6	0	-0.907703	3.331947	2.192162
9	7	0	-0.828407	4.413755	1.317587
10	6	0	0.041456	2.331643	2.593037
11	8	0	1.212548	2.171106	2.246837
12	8	0	2.115432	2.789100	-3.804425
13	8	0	0.591044	0.495019	-4.186728
14	7	0	-5.727460	3.222010	1.203365
15	7	0	-4.216820	1.448308	1.270035
16	6	0	-4.830820	2.442847	0.547300
17	8	0	-2.661931	-0.212642	1.572230
18	6	0	-3.176425	0.607188	0.813739
19	7	0	-4.540198	2.700546	-0.718169
20	6	0	-2.873659	0.893662	-0.556401
21	6	0	-3.566695	1.911133	-1.210681
22	7	0	-1.940901	0.357977	-1.435794
23	7	0	-3.039116	1.959471	-2.481542
24	6	0	-2.067168	1.010411	-2.585306
25	8	0	-1.265950	-3.304786	1.575712
26	7	0	-3.253229	-4.447918	1.642210
27	7	0	-5.172724	-5.752404	1.851789
28	6	0	-2.316057	-3.600024	1.007097
29	6	0	-4.429408	-4.913837	1.100514
30	6	0	-2.786429	-3.233895	-0.297283
31	7	0	-4.831081	-4.598801	-0.123289
32	6	0	-3.982675	-3.775908	-0.765508
33	7	0	-2.243891	-2.430437	-1.297839
34	7	0	-4.135652	-3.288970	-2.043896
35	6	0	-3.074954	-2.484125	-2.327767
36	17	0	0.996454	5.600086	-2.761450
37	1	0	-3.155047	0.529739	5.174128
38	1	0	-3.798247	4.663934	2.456863
39	1	0	-1.668999	-0.306578	5.000182
40	1	0	-2.290259	5.885914	0.803926
41	1	0	0.091543	0.711216	3.848536
42	1	0	3.033840	2.746592	-3.508236
43	1	0	1.762237	3.638652	-3.465091
44	1	0	1.141964	1.285588	-3.997859

45	1	0	0.832914	-0.157797	-3.515355
46	1	0	-6.181919	2.874898	2.036828
47	1	0	-3.315570	2.598527	-3.216874
48	1	0	-1.440215	0.856309	-3.454943
49	1	0	-6.279848	3.841560	0.625971
50	1	0	-4.406814	1.369566	2.265396
51	1	0	-4.997915	-5.875084	2.838016
52	1	0	-4.902268	-3.496409	-2.671853
53	1	0	-2.950746	-1.962850	-3.262941
54	1	0	-6.086969	-6.002163	1.503873
55	1	0	-2.977512	-4.755034	2.569110
56	78	0	-0.627346	-1.185720	-1.118651
57	7	0	1.032362	-0.067842	-0.830038
58	7	0	0.787680	-2.660290	-0.913123
59	6	0	2.064795	-2.232636	-0.674804
60	6	0	2.187171	-0.787132	-0.586050
61	6	0	3.114052	-3.143935	-0.557526
62	6	0	3.231730	0.080251	-0.316854
63	6	0	2.850440	-4.505369	-0.683108
64	6	0	1.543369	-4.927497	-0.927903
65	6	0	0.537987	-3.975103	-1.041124
66	7	0	2.674130	1.352982	-0.402193
67	6	0	3.257057	2.578995	-0.165258
68	6	0	1.322333	1.236790	-0.707464
69	6	0	2.487534	3.699883	-0.221647
70	6	0	1.097278	3.609125	-0.533126
71	6	0	0.525944	2.381686	-0.794105
72	6	0	0.249921	4.789913	-0.534811
73	6	0	4.645551	-0.142276	0.012324
74	6	0	5.661859	0.321428	-0.872935
75	6	0	4.991253	-0.802128	1.178078
76	6	0	6.988104	0.108803	-0.575768
77	6	0	6.351303	-1.040520	1.503674
78	6	0	7.372001	-0.574398	0.611242
79	6	0	8.733877	-0.811396	0.940720
80	6	0	6.728824	-1.724332	2.692043
81	6	0	9.069720	-1.478596	2.100092
82	6	0	8.059059	-1.938941	2.983336
83	1	0	-0.525154	2.285277	-1.007736
84	1	0	2.940842	4.664005	-0.029068
85	1	0	4.310883	2.570427	0.073963
86	1	0	3.656640	-5.225045	-0.594806
87	1	0	4.117363	-2.783667	-0.374095
88	1	0	-0.486421	-4.254662	-1.241686
89	1	0	1.295699	-5.976492	-1.035429
90	1	0	7.760855	0.457539	-1.254478
91	1	0	5.379405	0.828131	-1.790285
92	1	0	4.217056	-1.144034	1.858698
93	1	0	5.951448	-2.074427	3.365118
94	1	0	8.339397	-2.462151	3.892200
95	1	0	10.113171	-1.654490	2.342614
96	1	0	9.506212	-0.458307	0.263395
97	1	0	0.607050	5.728527	-0.143694
98	1	0	-0.726287	4.748031	-0.985768

13

Center Number	Atomic Number	Atomic Type	Coordinates (Angstroms) X	Y	Z
1	6	0	-7.673624	-0.546097	0.482049
2	6	0	-6.780972	0.003299	1.460299
3	6	0	-9.073300	-0.494411	0.722034
4	6	0	-9.566923	0.074872	1.877180
5	6	0	-7.320012	0.582330	2.642133
6	6	0	-8.683101	0.617762	2.845443
7	6	0	-7.128306	-1.124667	-0.697273
8	6	0	-5.383048	-0.050205	1.223688
9	6	0	-5.769189	-1.157394	-0.906620
10	6	0	-4.879104	-0.608518	0.062391
11	6	0	-3.430674	-0.634345	-0.178969
12	6	0	-2.490304	0.363044	-0.339119
13	7	0	-2.715050	-1.823297	-0.305642
14	7	0	-1.239474	-0.194284	-0.553046
15	6	0	-1.374253	-1.527453	-0.527053
16	6	0	-3.159079	-3.119431	-0.209368
17	6	0	-2.261978	-4.139543	-0.325237
18	6	0	-0.877568	-3.870061	-0.533082
19	6	0	-0.439120	-2.571070	-0.638937
20	6	0	0.061997	-5.050950	-0.617187
21	6	0	-2.556462	1.813638	-0.358471
22	6	0	-2.385772	4.563993	-0.458099
23	6	0	-3.721523	2.574793	-0.254353
24	6	0	-3.635754	3.962974	-0.303315
25	6	0	-1.260468	3.757276	-0.566399
26	7	0	-1.339397	2.414924	-0.516508
27	78	0	0.260834	1.154026	-0.772967
28	7	0	1.803532	-0.135043	-1.211509
29	6	0	2.997739	-0.335269	-0.523432
30	6	0	1.924561	-0.737268	-2.388584
31	6	0	3.850551	-1.089084	-1.328510
32	6	0	3.450279	0.081913	0.770985
33	7	0	3.145021	-1.337260	-2.484665
34	7	0	5.097896	-1.512950	-1.052856
35	7	0	4.784929	-0.336060	0.982514
36	8	0	2.853366	0.713671	1.640947
37	6	0	5.545734	-1.095899	0.125858
38	7	0	6.809083	-1.384938	0.495543
39	7	0	1.675221	2.631230	-0.913525
40	6	0	2.128878	3.458339	0.110597
41	6	0	2.442003	2.867001	-1.968042
42	6	0	3.207789	4.200777	-0.365684
43	6	0	1.671515	3.669849	1.452990
44	7	0	3.378404	3.810761	-1.674959
45	7	0	3.947139	5.111131	0.293754
46	7	0	2.498573	4.613836	2.105722
47	8	0	0.706988	3.183096	2.040778
48	6	0	3.563518	5.285794	1.550709
49	7	0	4.250665	6.133485	2.345292
50	7	0	1.455634	-4.679485	-0.349828
51	6	0	1.952001	-4.194196	0.859122
52	6	0	2.460978	-4.727854	-1.223362
53	6	0	3.306988	-3.948109	0.683797

54	6	0	1.332235	-3.936260	2.125472
55	7	0	3.588729	-4.293993	-0.626792
56	7	0	4.173532	-3.466726	1.581252
57	7	0	2.284581	-3.416530	3.026767
58	8	0	0.163047	-4.112419	2.457717
59	6	0	3.620151	-3.204377	2.763026
60	7	0	4.378751	-2.686153	3.741055
61	1	0	-9.748317	-0.910642	-0.020308
62	1	0	-10.637983	0.109538	2.050855
63	1	0	-6.639694	0.996424	3.380779
64	1	0	-9.086782	1.062257	3.749797
65	1	0	-7.803684	-1.538188	-1.440509
66	1	0	-4.705248	0.355250	1.969277
67	1	0	-5.364510	-1.587654	-1.816539
68	1	0	-4.215657	-3.254840	-0.026470
69	1	0	-2.615916	-5.159372	-0.234210
70	1	0	0.599632	-2.314232	-0.771298
71	1	0	-4.675728	2.078804	-0.140343
72	1	0	-4.533677	4.565745	-0.224916
73	1	0	-2.274031	5.640484	-0.501559
74	1	0	-0.275281	4.178736	-0.703113
75	1	0	1.164063	-0.751397	-3.164533
76	1	0	5.172137	-0.056406	1.877651
77	1	0	7.119650	-1.264568	1.448168
78	1	0	7.313565	-2.049256	-0.073606
79	1	0	2.357471	2.381725	-2.926831
80	1	0	2.239336	4.800591	3.068937
81	1	0	3.885177	6.436195	3.235776
82	1	0	4.945185	6.719000	1.904551
83	1	0	2.384033	-5.052931	-2.249104
84	1	0	1.917047	-3.202796	3.949133
85	1	0	4.038604	-2.576262	4.684628
86	1	0	5.376003	-2.641132	3.590318
87	1	0	3.482887	-1.851470	-3.288813
88	1	0	4.077920	4.165333	-2.315290
89	1	0	4.490730	-4.194883	-1.081764
90	1	0	-0.224700	-5.811950	0.110914
91	1	0	0.031481	-5.505358	-1.610348
92	8	0	-2.957085	-1.109796	-3.316363
93	1	0	-3.548975	-0.356176	-3.451216
94	1	0	-3.364257	-1.843618	-3.798115
95	8	0	-0.431178	-0.487724	-4.337114
96	1	0	-1.315992	-0.719069	-3.981093
97	1	0	-0.433375	-0.788236	-5.255136

References

[1] Su H, Wang L, Rao H, et al. Iron-Catalyzed Dehydrogenative sp3–sp2 Coupling via Direct Oxidative C–H Activation of Acetonitrile. Org Lett 2017;19:2226–9.

[2] Selwood DL, Brummell DG, Budworth J, et al. Synthesis and Biological Evaluation of Novel Pyrazoles and Indazoles as Activators of the Nitric Oxide Receptor, Soluble Guanylate Cyclase. J Med Chem 2001;44:78–93.

[3] Abdelfattah MAO, Lehmann J, Abadi AH. Discovery of highly potent and selective D4 ligands by interactive SAR study. Bioorg Med Chem Lett 2013;23:5077–81.

[4] Bar-Nahum I, Khenkin AM, Neumann R. Mild, Aqueous, Aerobic, Catalytic Oxidation of Methane to Methanol and Acetaldehyde Catalyzed by a Supported Bipyrimidinylplatinum−Polyoxometalate Hybrid Compound. J Am Chem Soc 2004;126:10236–7.

[5] Galindo-Murillo R, Cheatham III TE. Ethidium bromide interactions with DNA: an exploration of a classic DNA–ligand complex with unbiased molecular dynamics simulations. Nucleic Acids Res 2021;49:3735–47.

[6] Galindo-Murillo R, Winkler L, García-Ramos JC, et al. Ancillary Ligand in Ternary CuII Complexes Guides Binding Selectivity toward Minor-Groove DNA. J Phys Chem B 2020;124:11648–58.

[7] Zgarbová M, Šponer J, Otyepka M, et al. Refinement of the Sugar–Phosphate Backbone Torsion Beta for AMBER Force Fields Improves the Description of Z- and B-DNA. J Chem Theory Comput 2015;11:5723–36.

[8] Wang J, Wolf RM, Caldwell JW, et al. Development and testing of a general amber force field. J Comput Chem 2004;25:1157–74.

[9] Li P, Merz KMJr. MCPB.py: A Python Based Metal Center Parameter Builder. J Chem Inf Model 2016;56:599–604.

[10] Weigend F, Ahlrichs R. Balanced basis sets of split valence{,} triple zeta valence and quadruple zeta valence quality for H to Rn: Design and assessment of accuracy. Phys Chem Chem Phys 2005;7:3297–305.

[11] Frisch MJ, Trucks GW, Schlegel HB, et al. Gaussian16 Revision C.01 2016.

[12] Ryckaert J-P, Ciccotti G, Berendsen HJC. Numerical integration of the cartesian equations of motion of a system with constraints: molecular dynamics of n-alkanes. J Comput Phys 1977;23:327–41.

[13] Becke AD. Density-functional thermochemistry. III. The role of exact exchange. J Chem Phys 1993;98:5648.

[14] Hariharan PC, Pople JA. The influence of polarization functions on molecular orbital hydrogenation energies. Theor Chim Acta 1973;28:213–22.

[15] W. J. Hehre, R. Ditchfield and JAP. Self—Consistent Molecular Orbital Methods. XII. Further Extensions of Gaussian—Type Basis Sets for Use in Molecular Orbital Studies of Organic Molecules. J Chem Phys 1972;56:2257.

[16] Hay PJ, Wadt WR. *Ab initio* effective core potentials for molecular calculations. Potentials for K to Au including the outermost core orbitals. J Chem Phys 1985;82:299–310.

[17] Tomasi J, Mennucci B, Cammi R. Quantum Mechanical Continuum Solvation Models. Chem Rev 2005;105:2999–3094.

[18] Grimme S, Antony J, Ehrlich S, et al. A consistent and accurate ab initio parametrization of density functional dispersion correction (DFT-D) for the 94 elements H-Pu. J Chem Phys 2010;132:154104.

[19] Grimme S, Ehrlich S, Goerigk L. Effect of the Damping Function in Dispersion Corrected Density Functional Theory. J Comput Chem 2011;32:1456–65.
