# Supplementary material for: New platinum derivatives selectively cause double-strand DNA breaks and death in naïve and cisplatin-resistant cholangiocarcinomas
Source: J Hepatol. 2025 Nov;83(5):1077–91. doi: 10.1016/j.jhep.2025.04.034 (PMC12547501; doi:10.1016/j.jhep.2025.04.034)
Supplement: Multimedia component 3 [file mmc3.pdf]

JOURNAL
OF HEPATOLOGY

Journal of Hepatology

CTAT methods

Tables for a "Complete, Transparent, Accurate and Timely account" (CTAT) are now mandatory for all revised submissions. The aim is to enhance the reproducibility of methods.

- Only include the parts relevant to your study
- Refer to the CTAT in the main text as 'Supplementary CTAT Table'
- Do not add subheadings
- Add as many rows as needed to include all information
- Only include one item per row

If the CTAT form is not relevant to your study, please outline the reasons why:

1.1 Antibodies

Name	Citation	Supplier	Cat no.	Clone no.
Mouse monoclonal anti-ATR	n/a	Santa Cruz Biotechnology	sc-515173	C-1
Rabbit monoclonal anti-p-ATR	n/a	Abcam	ab223258	T1989
Mouse monoclonal anti-CHK1	n/a	Cell Signaling	2360S	2G1D5
Rabbit monoclonal anti-p-CHK1 (Ser345)	n/a	Cell Signaling	2348T	133D3
Mouse monoclonal anti-CDC2	n/a	Santa Cruz Biotechnology	sc-54	n/a
Mouse monoclonal anti-p-CDC2	n/a	Santa Cruz Biotechnology	sc-136014	pY15.44
Mouse monoclonal anti β-actin	n/a	Sigma-Aldrich	A5316	AC-74
Rabbit monoclonal anti-CK19	n/a	Abcam	ab52625	EP1580Y
Rabbit monoclonal anti-CD4	n/a	Abcam	ab183685	EPR19514
Rabbit monoclonal anti-CD8	n/a	Cell Signaling	98941	D4W2Z
Rabbit monoclonal anti-KI67	n/a	Abcam	ab16667	SP6
Rabbit polyclonal anti-PCNA	n/a	Abcam	ab18197	n/a
Rabbit monoclonal anti-Cleaved Caspase-3 (Asp175)	n/a	Cell Signaling	9664	5A1E
Rabbit polyclonal anti-phospho-Histone H3 (Ser28)	n/a	Cell Signaling	9713	n/a
Anti-mouse IgG, HRP-linked Antibody	n/a	Cell Signaling Technology	#7076	n/a
Anti-rabbit IgG, HRP-linked Antibody	n/a	Cell Signaling Technology	#7074	n/a

Biotin-conjugated polyclonal pig anti-rabbit antibody	n/a	Dako	E0353	n/a

1.2 Cell lines

Name	Citation	Supplier	Cat no.	Passage no.	Authentication test method
Normal human cholangiocytes (NHC)	Merino-Azpitarte M. et al. JHEP.2017; Erice O. *et al.* Biochim Biophys Acta Mol Basis Dis. 2018.	Isolated	n/a	5-10	Biliary markers expression (CK-7, CK-19, AQP, AE2)
EGI-1	Scherdin G *et al.* Immunobiology 1987.	Leibniz Institute DSMZ-German Collection of Microorganisms and Cell Cultures GmbH	ACC 385	P8-P16	Biliary markers expression (CK-7, CK-19)
HUCCT1	Miyagiwa M *et al.* In Vitro Cell Dev Biol Anim 1989.	Accegen	ABC-0433	P8-P16	Biliary markers expression (CK-7, CK-19)
EGI-1R	n/a	Developed	n/a	>120 (from initial EGI-1 culture)	Biliary markers expression (CK-7, CK-19) and viability assay in the presence of cisplatin

1.3 Organisms

Name	Citation	Supplier	Strain	Sex	Age	Overall n number
Crl:CD1-Foxn1nu mice	Merino-Azpitarte M. *et al.* JHEP.2017	Charles River Laboratories	Crl:CD1-Foxn1nu; strain 086	Male	7 weeks	62
C57BL6/J	Rizvi S. *et al.* Oncotarget. 2017	Charles River Laboratories	C57Bl6/J	Male	8 weeks	48
C57BL6/J	n/a	Charles River Laboratories	C57Bl6/J	Male	8 weeks	56

1.4 Sequence based reagents

Name	Sequence	Supplier
SLC22A1	Forward 5'-GTCGCTTTGCCAGAGACCAT-3'<br>Reverse 5'-CTTCATCCCTCCAACATGACA-3'	
SLC22A3	Forward 5'-ATCGTCAGCGAGTTTGACCTT-3'<br>Reverse 5'-ACCTGTCTGCTGCATAGCCTA-3'	
SLC31A1	Forward 5'-TGCGTAAGTCACAAGTCAGC-3'<br>Reverse 5'-CTGCTACTGCAATGCAGAGG-3'	
SLC51A1	Forward 5'- TTCCAGGTTCTCCTCATCCTGAC-3'<br>Reverse 5'- CAATTCATCACTTGAGACCTGGTTT-3'	
GADPH	Forward 5'-CCAAGGTCATCCATGACAAC-3'<br>Reverse 5'-TGTCATACCAGGAAATGAGC-3'	

1.5 Biological samples

Description	Source	Identifier

1.6 Deposited data

Name of repository	Identifier	Link
PRIDE	PXD061935	

1.7 Software

Software name	Manufacturer	Version
R	n/a	4.3.3. and 4.2.1
QuPath	Bankhead, P. et al.	V0.5.1
DIA-NN software	Aptila	v1.8.1.
MSFragger	Nesvilab	V4.1
IonQuant	Nesvilab	V1.10.27
FragPipe	Nesvilab	V22.0
QIAGEN IPA	QIAGEN	December 14, 2024
Bio-Rad CFX MaestroTM 1.0	Bio-Rad	4.0.2325.0418
Microsoft Excel for Windows	Microsoft Corporation	16.32
GraphPad Prism	GraphPad Software, Inc	8.0.2
Zen (blue edition)	ZEISS	3.1
CytExpert	Beckman Coulter Inc.	2.4.0.28
Image J	National Institutes of Health-Bethesda	1.50

1.8 Other (e.g. drugs, proteins, vectors etc.)

Aurkines	Synthesized in at the University of the Basque Country	
Cisplatin	Synthesized in at the University of the Basque Country	
Rifampicin	MCE	
Taurocholic acid	Sigma-Aldrich	
Mifepristone	MCE	
Quinine	MCE	
AKT plasmid	Kindly provided by Dr. Calvisi	
YAP plasmid	Kindly provided by Dr. Calvisi	
Sleeping beauty transposase plasmid	Kindly provided by Dr. Calvisi	
pUC18 plasmid	Thermo Fisher, ref. SD0051	

1.9 Please provide the details of the corresponding methods author for the manuscript:

Jesus M. Banales, Prof, Department of Liver and Gastrointestinal Diseases, Biodonostia Health Research Institute – Donostia University Hospital, Paseo del Dr. Begiristain s/n, E-20014, San Sebastian, Spain. e-mail: jesus.banales@biodonostia.org / Phone: +34 943006067; Fax: +34 943006250

2.0 Please confirm for randomised controlled trials all versions of the clinical protocol are included in the submission. These will be published online as supplementary information.

n/a
