# Supplementary material for: New platinum derivatives selectively cause double-strand DNA breaks and death in naïve and cisplatin-resistant cholangiocarcinomas
Source: J Hepatol. 2025 Nov;83(5):1077–91. doi: 10.1016/j.jhep.2025.04.034 (PMC12547501; doi:10.1016/j.jhep.2025.04.034)
Supplement: Multimedia component 5 [file mmc5.pdf]

Research Article
Hepatic and Biliary Cancer

JOURNAL OF HEPATOLOGY

New platinum derivatives selectively cause double-strand DNA breaks and death in naïve and cisplatin-resistant cholangiocarcinomas

Authors

Irene Olaizola, Mikel Odriozola-Gimeno, Paula Olaizola, …, Jose J.G. Marin, Fernando P. Cossío, Jesus M. Banales

Correspondence

jesusmaria.banalesasurmendi@bio-gipuzkoa.eus (J.M. Banales), fp.cossio@ehu.es (F.P. Cossío).

Graphical abstract

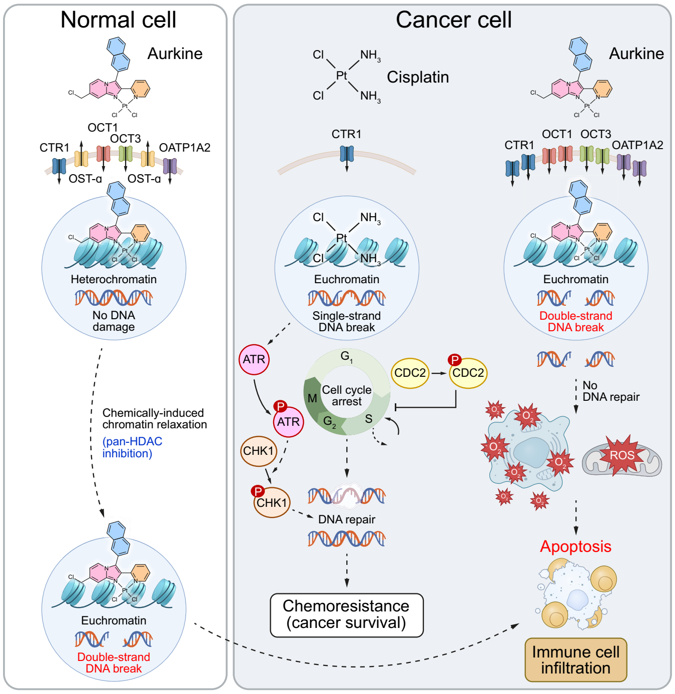

Highlights

- Aurkines represent a new generation of chemotherapeutic agents with distinctive polielectrophilic properties.
- Aurkines selectively target DNA in cancer cells, including cholangiocarcinoma.
- Aurkines demonstrate greater efficacy than cisplatin in experimental models of both naïve and cisplatin-resistant CCA.
- Aurkines show no evident systemic toxicity at therapeutic doses.
- Aurkines impact the tumor microenvironment, targeting cancer-associated fibroblasts and boosting immunogenicity.

Impact and implications

This study introduces a novel therapeutic strategy designed to induce frequent double-strand DNA breaks selectively in both naïve and cisplatin-resistant cancer cells, without evident toxic side effects at therapeutic doses. This approach may form the basis for new strategies to overcome the critical challenge of drug resistance in cancer treatment and has the potential to be a breakthrough not only for the treatment of biliary tumors but also for other cancers.

https://doi.org/10.1016/j.jhep.2025.04.034
© 2025 The Author(s). Published by Elsevier B.V. on behalf of European Association for the Study of the Liver. This is an open access article under the CC BY license (http://creativecommons.org/licenses/by/4.0/). J. Hepatol. 2025, 83, 1077–1091

Research Article
Hepatic and Biliary Cancer

JOURNAL OF HEPATOLOGY

New platinum derivatives selectively cause double-strand DNA breaks and death in naïve and cisplatin-resistant cholangiocarcinomas

Irene Olaizola[1], Mikel Odriozola-Gimeno[2], Paula Olaizola[1,3], Francisco J. Caballero-Camino[1,3], Noelia Pastor-Toyos[1], Mireia Tena-Garitaonaindia[1], Ainhoa Lapitz[1,3,4], Beatriz Val[1,3], Amanda R. Guimaraes[2], Maitane Asensio[3,5], Maider Huici-Izagirre[1], Colin Rae[6], David de Sancho[7], Xabier Lopez[7], Pedro M. Rodrigues[1,3,8], Elisa Herraez[3,5], Oscar Briz[3,5], Laura Izquierdo-Sanchez[1,3], Aitziber Eleta-Lopez[9,10], Alexander M. Bittner[8,10], Ana Martinez-Amesti[11], Teresa Miranda[11], Sumera I. Ilyas[12], Chiara Braconi[6,13], Maria J. Perugorria[1,3,14], Luis Bujanda[1,3,14], Iván Rivilla[2,8], Jose J.G. Marin[3,5], Fernando P. Cossío[2,*,‡], Jesus M. Banales[1,3,8,15,*,‡]

Journal of Hepatology **2025**. vol. 83 | 1077–1091

Check for updates

Background & Aims: Patients with cholangiocarcinoma (CCA) have poor prognosis. Current cisplatin-based first-line chemotherapy offers limited survival benefit. Cisplatin induces single-strand DNA breaks, activating DNA repair mechanisms that diminish its effectiveness. Here, we present the design, chemical synthesis, and therapeutic evaluation of a new generation of chemotherapeutic agents (Aurkines) with unique polyelectrophilic properties. These agents cause a high frequency of double-strand DNA breaks, bypassing DNA repair, and promoting cancer cell death.

Methods: Two novel compounds, Aurkine 16 and Aurkine 18, were designed and evaluated for their antitumor effects in both naïve and cisplatin-resistant CCA cells, cancer-associated fibroblasts, healthy cholangiocytes, and *in vivo* models.

Results: Aurkines effectively induced double-strand DNA breaks, leading to increased DNA damage and elevated levels of reactive oxygen species, resulting in greater cytotoxicity than cisplatin in CCA cells. Phosphoproteomic and molecular analysis revealed that cisplatin activates DNA repair pathways, while Aurkines primarily induce apoptosis. Importantly, Aurkines also triggered apoptosis in cisplatin-resistant CCA cells and cancer-associated fibroblasts without harming healthy cholangiocytes. Additionally, Aurkines demonstrated cytotoxicity in other cisplatin-resistant cancers, such as breast and ovarian cancer. This tumor selectivity results from reduced uptake, increased efflux, and compact chromatin structure in normal cells, limiting Aurkine-DNA interactions. *In vivo*, Aurkines inhibited the growth of subcutaneous naïve and cisplatin-resistant CCA tumors, as well as orthotopic tumors in immunocompetent mice, promoting antitumor immune cell recruitment without any adverse events. Transport studies revealed that Aurkines were selectively taken up by OCT1, OCT3, CTR1, and OATP1A2, whereas only CTR1 transported cisplatin.

Conclusions: Aurkines represent promising therapeutic drugs for both naïve and cisplatin-resistant cancers due to their unique polyelectrophilic properties and selective targeting of malignant cells.

© 2025 The Author(s). Published by Elsevier B.V. on behalf of European Association for the Study of the Liver. This is an open access article under the CC BY license (http://creativecommons.org/licenses/by/4.0/).

Introduction

Cholangiocarcinoma (CCA) comprises a heterogeneous group of malignant tumors arising along the biliary tree.[1] Ranked as the second most frequent primary liver cancer, CCA contributes to 3% of all gastrointestinal malignancies and accounts for 2% of all cancer-related deaths.[2] However, these statistics are likely underestimated due to errors in disease coding, diagnosis, and data retrieval. Globally, the incidence (0.3-6 cases per 100,000 inhabitants annually) and mortality (1-6 per 100,000 inhabitants annually) of CCA are on the rise, representing a significant health challenge.[1] Patients with early stage CCA typically lack symptoms, leading to the majority of diagnoses at advanced stages (70%), often when the cancer has already spread.[2] This delayed diagnosis, coupled with the tumor chemoresistance, contributes to unfavorable outcomes. Current curative strategies for CCA include surgical resection or liver transplantation, although less than 30% of patients are eligible for these interventions and the risk of tumor recurrence is still high.[1,2] In unresectable cases, palliative treatment based on systemic therapy remains the only feasible option. Until

* Corresponding authors. Addresses: Department of Liver and Gastrointestinal Diseases, Biogipuzkoa Health Research Institute – Donostia University Hospital, Paseo Dr. Begiristain s/n, E-20014, San Sebastian, Spain. (J.M. Banales), or Department of Organic Chemistry I, Faculty of Chemistry, University of the Basque Country (UPV/EHU), Paseo Manuel Lardizabal, 3, 20018, San Sebastian, Spain. (F.P. Cossío).
E-mail addresses: jesusmaria.banalesasurmendi@bio-gipuzkoa.eus (J.M. Banales), fp.cossio@ehu.es (F.P. Cossío).
‡ Share senior authorship
https://doi.org/10.1016/j.jhep.2025.04.034

recently, the first-line treatment for advanced CCA involved a combination of gemcitabine + cisplatin (GemCis),[3] which evolved into a triple combination with the inclusion of the monoclonal antibodies durvalumab (anti-PD-L1)[4] or pembrolizumab (anti-PD1).[5] Nevertheless, the median overall survival achieved with this combined therapy remains modest, with a one-year estimate, and this regimen is still considered palliative.[4,5]

Cisplatin (CisPt) is a fundamental therapeutic agent in the fight against various cancer types.[6] Whether used as monotherapy or in combination with other therapies, CisPt currently stands as the primary treatment for different types of solid cancers, including CCA, breast, ovarian, lung, and testicular cancers, among others. However, the use of CisPt is associated with potential side effects, such as myelotoxicity, nephrotoxicity, neuropathy, ototoxicity, and the tendency to induce nausea and vomiting.[7] Resistance to CisPt still remains a challenge in cancer treatment, with certain tumors exhibiting intrinsic resistance while others gradually acquire resistance over time, despite an initial positive response.[8]

CisPt, cis-$[Pt(NH_3)_2Cl_2)]$, is a platinum (Pt) compound that binds to DNA, inducing mainly single-strand DNA breaks and subsequently causing cancer cell death.[9,10] This process includes various steps: (i) cellular uptake through diffusion and facilitated transport; (ii) intracellular activation through interaction with water molecules in the presence of low concentrations (10-20 mM) of chloride ions; this leads to the generation of aqua complexes cis-$[Pt(NH_3)_2Cl(OH_2)]^+$ and cis-$[Pt(NH_3)_2(OH_2)_2]^{2+}$, electrophilic substances that interact with DNA; (iii) nuclear uptake and interaction with nucleophilic centers N7 of the DNA nitrogen bases guanine (G) and, to a lesser extent, adenine (A), forming covalent bonds, preferably with two guanosine residues.[11] In this context, *in vitro* assays have revealed that CisPt generates 47-50% of adducts on a single strand (*i.e.* intrastrand), comprising two contiguous G units of the cis-$[Pt(NH_3)_2$\{d(GpG)\}] (or cis-GG) type. Additionally, 23-28% of intrastrand adducts integrate two consecutive A and G units of the cis-$[Pt(NH_3)_2$\{d(ApG)\}] (or cis-AG) type, while 8-10% of intrastrand adducts are composed of two Pt-G linkages separated by another base X in the cis-$[Pt(NH_3)_2$\{d(GpXpG)\}] (or cis-GXG) structure. Finally, 2-3% of CisPt-DNA adducts are single-base G linkages.[12] A parallel *in vitro* exploration involving a CisPt analogue yielded similar outcomes.[13] *Ex vivo* analyses with human cancer samples mirrored these findings, with cis-GG, cis-AG, and cis-GXG proportions being 65%, 22%, and 13%, respectively.[14] These data highlight that interstrand crosslinks, leading to double-strand DNA breaks, which are more challenging for cancer cells to repair,[15] represent a relatively rare event, accounting for less than 5% of crosslinks.[13,14] This allows cancer cells to activate their DNA repair mechanisms against single-strand DNA breaks, thereby enabling them to survive and develop resistance to CisPt.[16]

Considering the activity of Pt(II) derivatives and the resistance mechanisms developed by cancer cells, we hypothesize that novel chemotherapeutic agents with marked polyelectrophilic properties could induce interstrand DNA crosslinks. This mechanism would inhibit DNA repair mechanisms, thereby promoting cancer cell death. Considering the interstrand interactions between DNA and dielectrophilic chemotherapeutic agents like CisPt (Fig. 1A, 1st row), we reasoned that adding an extra electrophilic site would create a trielectrophilic agent capable of generating higher rates of double-strand DNA breaks (Fig. 1A, 2nd row, highlighted in green). Thus, we formulated and synthesized a new family of chemotherapeutic agents with pronounced polyelectrophilic characteristics, named Aurkines. Among the synthesized compounds, Aurkine 16 and Aurkine 18 (Fig.1B), emerged as the most promising, exhibiting IC_{50} values for cell viability lower than those of CisPt and showing remarkable selectivity for cancer cells (Fig. S1). These compounds were designed to interact with DNA at multiple sites and were tested therapeutically for CCA. This design included a Pt(II) electrophilic center capable of substituting two chloride groups with two G (or A) units, along with an additional 4-chlorobenzyl carbon-based electrophilic center. Additionally, aryl groups (highlighted in pale blue in Fig. 1B,C) were incorporated to bind and intercalate with the minor and major grooves of DNA, as illustrated in Fig. 1C.

Materials and methods

Chemical synthesis of Aurkines 16 and 18

Aurkines 16 and 18 were synthesized as described in the supplementary information - chemical synthesis file.

Cell cultures

Normal human cholangiocytes (NHCs) were isolated from healthy liver tissue. Three human CCA cell lines were used: HUCCT1 (intrahepatic CCA), EGI-1 (extrahepatic CCA sensitive to CisPt) and EGI-1R (extrahepatic CCA resistant to CisPt). EGI-1R was generated as detailed in the supplementary information. Cancer-associated fibroblasts (CAFs) were isolated from resected intrahepatic CCA. Details of cell isolation, culture, and characterization are provided in the supplementary information.

Patient-derived organoids (PDOs) from CCA tumors

PDOs from patients with CCA were generated as described in the supplementary information.

Transcriptomic analysis of human samples

Transcriptomic analyses were performed on CCA tumors and adjacent non-tumor liver tissues using datasets from five distinct publicly available patient cohorts: AHN (dataset: GSE107943), The Thailand Initiative in Genomics and Expression Research (TIGER-LC; dataset: GSE76297), Copenhagen (dataset: GSE26566), Cancer Genome Atlas (TCGA-CHOL) and JOB (dataset: E-MATB-6389). To assess the association between gene expression and tumor mutational profiles, data from the publicly available DONG cohort (OEP001105) was used.

scRNA-seq analysis of human CCA tissues

Publicly available single-cell RNA sequencing (scRNA-seq) data from human CCA tumors (GSE151530) were analyzed as described in the supplementary information.

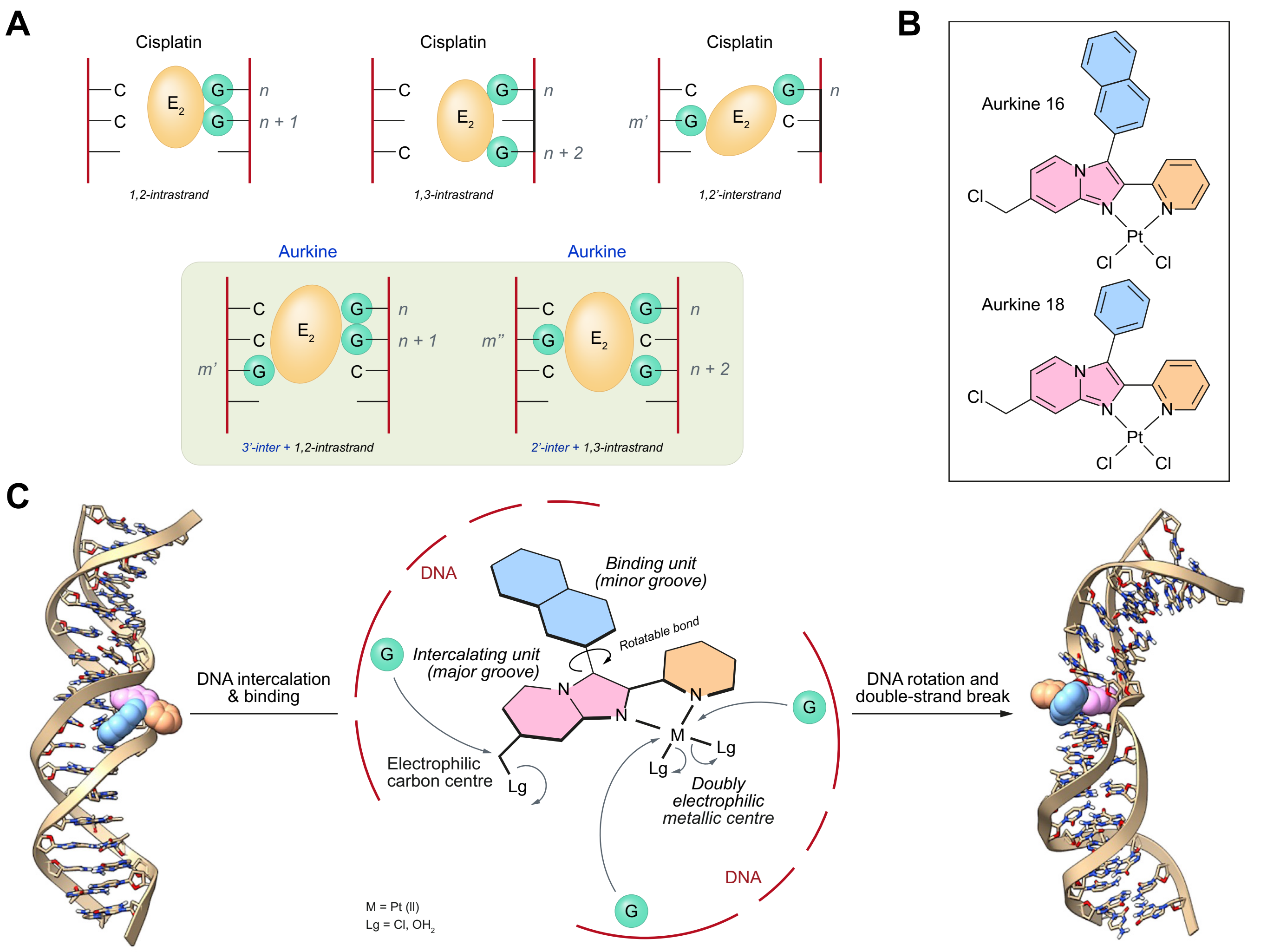

Fig. 1. Design, structure, and interaction modes of Aurkines. (A) Interaction of dielectrophilic (E_2) and trielectrophilic (E_3) platinum reagents with guanine (G) units. (B) Chemical structures of Aurkines 16 and 18. (C) Molecular dynamics simulations illustrating the interaction of Aurkine 16 with DNA, highlighting both the initial minor groove binding associated with the β-naphthyl moiety (pale blue) and the subsequent major groove intercalation of the heterocyclic groups (pink and orange).

RNA isolation and gene expression

Total RNA was extracted from cultured cells using TRI Reagent® and analyzed by reverse transcription and quantitative real-time PCR, as outlined in the supplementary information. Primer sequences are listed in Table S1.

Histological analyses

H&E staining was performed to assess tissue morphology, as previously described.[17] Immunohistochemistry (IHC) was used to evaluate markers of proliferation (Ki67), apoptosis (cleaved caspase-3), DNA damage (p-H2AX) and immune cell populations (CD4 and CD8), as previously detailed.[17] The antibodies used are listed in Table S2.

Immunoblotting

Protein expression in CCA cells and NHCs was analyzed using immunoblotting, with antibodies listed in Table S2. Methodological details are provided in the supplementary information.

ROS detection, cell viability, proliferation, cell cycle, and apoptosis assays

Assays for reactive oxygen species (ROS) detection, cell viability, proliferation, cell cycle, and apoptosis were conducted on NHCs, CCA cells, and CAFs in the presence or absence of CisPt or Aurkines (10 μM or 20 μM), following protocols described in the supplementary information.

3D spheroids

3D spheroids were generated and monitored as described in the supplementary information.

Microscopy and molecular analyses

Atomic force microscopy (AFM), transmission electron microscopy (TEM), pUC18 plasmid mobility, and comet assays were conducted to evaluate DNA interactions and damage, as detailed in the supplementary information.

Experimental overexpression of human transporters in cells and transport assays

Overexpression of human transporters, along with direct and indirect transport assays, were performed using flow cytometry and HPLC-MS/MS, respectively.

Mass spectrometry-based high-throughput phosphoproteomic analysis

Mass spectrometry-based high-throughput phosphoproteomic analysis was performed in the EGI-1 CCA cell line following treatment with CisPt, Aurkine 16, or Aurkine 18, as described in the supplementary information.

Experimental animal models of CCA

Subcutaneous CCA xenografts in immunodeficient mice and orthotopic CCA models in immunocompetent mice were generated as described in the supplementary information.

Statistical analyses

Statistical analyses are outlined in the supplementary information.

Results

Chemical synthesis of Aurkines 16 and 18

These syntheses were planned based on our previous experience on 3-arylimidazo-[1,2-*a*]pyridines.[18] Basically, a double addition of 2-bromo-1-(pyridine-2-yl)ethan-1-one **(2)** with methyl 2-aminoisonicotinate **(3)** yielded adduct **(4)** in good yield. From this intermediate, both β-naphthyl and phenyl groups were added *via* palladium-catalysed C–C coupling with the corresponding aryl bromides. Then, the electrophilic carbon centers were generated by reduction of the ester group of intermediates **(5)** and **(6)**, followed by chloro-substitution of the alcohol groups. Finally, Aurkines 16 and 18 were obtained in good yields by means of the reaction of chlorides **(7)** and **(8)** with platinum dichloride complexed to two equivalents of dimethyl sulfoxide (Fig. S2).

Assessment of the triple electrophilicity of Aurkine 16

Molecular dynamics simulations (see supplementary information for further details) of Aurkine 16 on the model sequence 5'-GCACGAACGGACGAACGC-3' show that aryl groups highlighted in pale blue in Fig. 1C (2-naphthyl in the case of Aurkine 16) promote an initial interaction at the minor groove of DNA. The 2-pyridyl and imidazo-[1,2-*a*]pyridine groups, represented in orange and pink, respectively, induce an additional intercalation step that promotes a severe distortion of the double helix with base-pair eversion. These interactions may constitute the previous stage for the respective substitution reactions.

Computational analysis using density-functional theory on the steps involved in the nucleophilic substitution of the three chlorine atoms of Aurkine 16 by three G residues in the presence of at least two molecules of water show a complex sequential mechanism whose main features are shown in Fig. S3. Previous work from our group on CisPt[19] and electrophilic carbon atoms of nitrogen mustards[20] showed that this approach captures the essentials of this process in terms of selectivity and kinetics. Our calculations provide a complex stepwise mechanism that starts with a nucleophilic attack of one molecule of surrounding water to generate a mono-aqua complex **(9)**. This aqua ligand acts as a good leaving group in the subsequent nucleophilic attack by one equivalent of G to yield a mono-G substituted intermediate **(10)**. This latter intermediate is transformed into an activated dication **(11)** *via* nucleophilic substitution by another water molecule. The nucleophilic attack by a second G base yields intermediate **(12)**, whose carbon-centered substitution reaction by the third equivalent of G gives rise to the trisubstituted dicationic species **(13)**, with an overall exergonic reaction profile. The geometries of the critical transition structures **TS1-3** (Fig. S3) are coherent with the expected mechanism, the corresponding activation free energies lying in the range of 10-23 kcal mol^{-1}. These results demonstrate that the design criteria of Aurkine 16 and 18 are kinetically and thermodynamically compatible with the mechanisms associated with these transformations.

Characterization of the binding of Aurkines 16 and 18 with DNA

To evaluate the interaction between Aurkine 16 and isolated DNA from *Escherichia coli*, AFM and TEM were employed. AFM images showed that, after 10 min of incubation, CisPt induced some DNA bending, although the main structure of the DNA strand was preserved. In contrast, Aurkine 16 exerted a considerably different effect on DNA, disrupting its strand structure, as shown in Fig. 2A. TEM images further supported these observations, showing a progressive decrease in DNA density over time, consistent with DNA breakage (Fig. 2B). Electrophoretic mobility analysis of the pUC18 plasmid revealed that both Aurkine 16 and 18 induced linear fragments of multiple sizes, which appeared as a diffuse pattern. In contrast, CisPt left the plasmid's supercoiled form unaltered (Fig. 2C). At the molecular level, phosphoproteomic analysis of CCA cells exposed to Aurkines or CisPt for 3 h revealed that CisPt incubation activated DNA repair pathways, while Aurkine primarily promoted apoptotic pathways (Fig. 2D). Consistent with these observations, exposure of CCA cells to CisPt triggered the phosphorylation and activation of key proteins involved in single-strand DNA repair pathways, such as ATR and CHK1 (Fig. S4). This is consistent with the fact that the DNA damage induced by Aurkines exceeds a critical threshold, leading to direct cell death without activating DNA repair mechanisms. These results are in line with the computational data and underscore that Aurkines 16 and 18 operate through a distinct mechanism compared to CisPt.

Evaluation of the antitumor effect of Aurkines 16 and 18 on human CCA cells

Having confirmed the disruptive impact of Aurkines 16 and 18 on DNA structure, including the generation of double-strand DNA breaks, we analyzed their genotoxic impact *in vitro* using the comet assay. This analysis was conducted on two human CCA cell lines (EGI-1 and HUCCT1) and NHCs. Interestingly, CisPt did not induce significant DNA damage, as evidenced by the "tail moment", in either NHCs or CCA cells at tested concentrations. In contrast, Aurkines 16 and 18 induced remarkable DNA damage, specifically in human CCA cells. This was characterized by an increased relative amount of DNA in

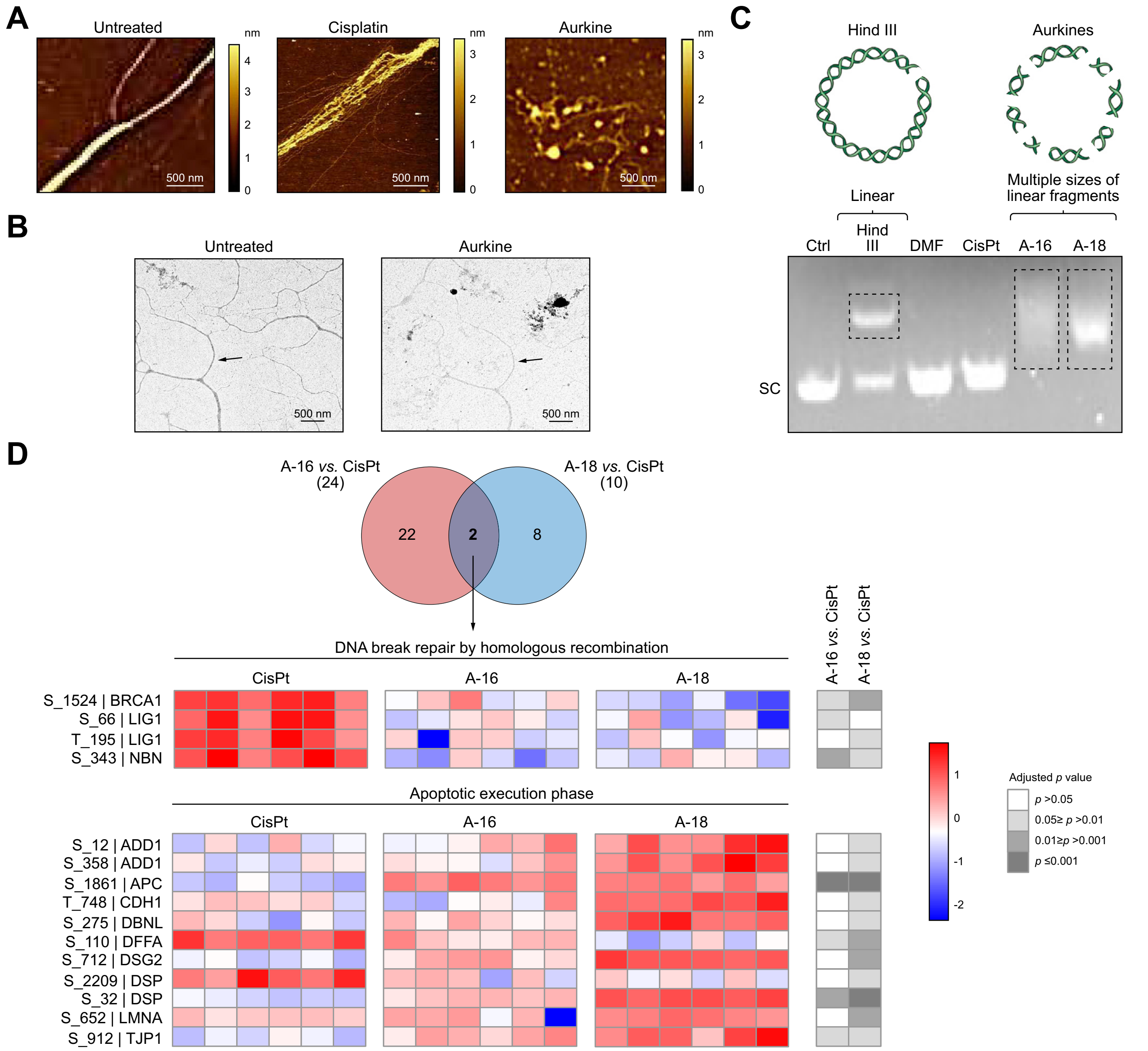

Fig. 2. Effect of Aurkine on isolated DNA. (A) Atomic force microscopy (AFM) images of untreated DNA and DNA incubated with CisPt or Aurkine 16 for 10 min. (B) Transmission electron microscopy (TEM) images of untreated DNA, and DNA incubated with Aurkine 16 for 10 min. (C) Agarose gel analysis of pUC18 plasmid after 1 h incubation with vehicle, CisPt, Aurkine 16 or 18. HindIII digestion was used as control. (D) Venn diagram showing the number of canonical pathways with significant adjusted p values in both comparisons and a heatmap illustrating the altered protein phosphorylation in EGI-1 cells after a 3-hour exposure to CisPt or Aurkines. Enriched proteins are coloured in red and proteins with lower abundance are displayed in blue. CisPt, cisplatin; DMF, dimethylformamide; SC, supercoiled.

the comet tail and a greater migration of the genetic material from the nucleus. Importantly, no DNA damage was observed in NHCs, as they remained entirely unaffected by Aurkine compounds (Fig. 3A).

In agreement with the increased DNA damage, flow cytometry analysis demonstrated that both Aurkines 16 and 18 increased total and mitochondrial ROS levels specifically in CCA cells (Fig. 3B,C). This increased oxidative stress correlated with a marked reduction in CCA cell viability following treatment with Aurkines 16 and 18, compared to the milder effects observed with CisPt (Fig. 3D). On the other hand, in contrast to Aurkine 16, Aurkine 18 induced a slight increase in mitochondrial ROS in NHCs, similar to the effect of CisPt (Fig. 3C), all of which resulted in a mild decrease in cell viability (Fig. 3D). To further investigate these antitumor effects, we examined whether the observed reduction in CCA cell viability could be attributed to a decrease in proliferation and/or an increase in apoptosis. Flow cytometry revealed that both Aurkines 16 and 18 selectively targeted cancer cells, promoting greater apoptosis in CCA cells compared to CisPt, while leaving NHCs unaffected (Fig. 4A,B). Of note, while the addition of gemcitabine significantly enhanced the apoptotic effect of CisPt, it

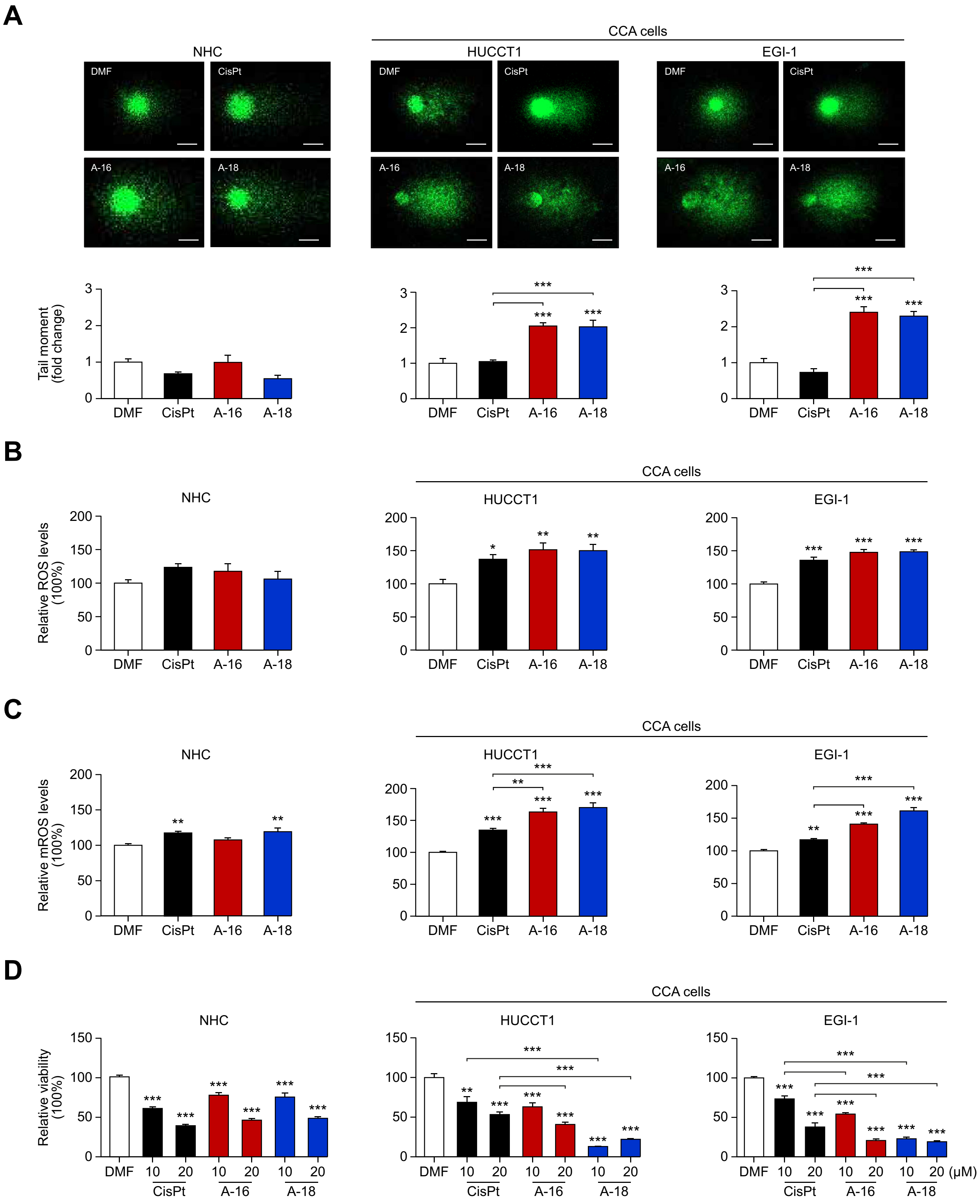

Fig. 3. Genotoxic effects of Aurkines 16 and 18 on human CCA cells. (A) DNA damage, (B) ROS and (C) mROS in NHCs and CCA cell lines (HUCCT1 and EGI-1) after 24-hour incubation with vehicle, CisPt, Aurkines 16 or 18 (10 μM). (D) Cell viability of NHCs and CCA cell lines (HUCCT1 and EGI-1) after 48-hour incubation with vehicle, CisPt, Aurkines 16 or 18 (10 μM and 20 μM). One-way ANOVA test or Student's *t* tests were used. Data are shown as mean ± SEM. *p ≤0.05, **p ≤0.01, ***p ≤0.001). CCA, cholangiocarcinoma; CisPt, cisplatin; DMF, dimethylformamide; NHCs, normal human cholangiocytes; mROS, mitochondrial ROS; ROS, reactive oxygen species.

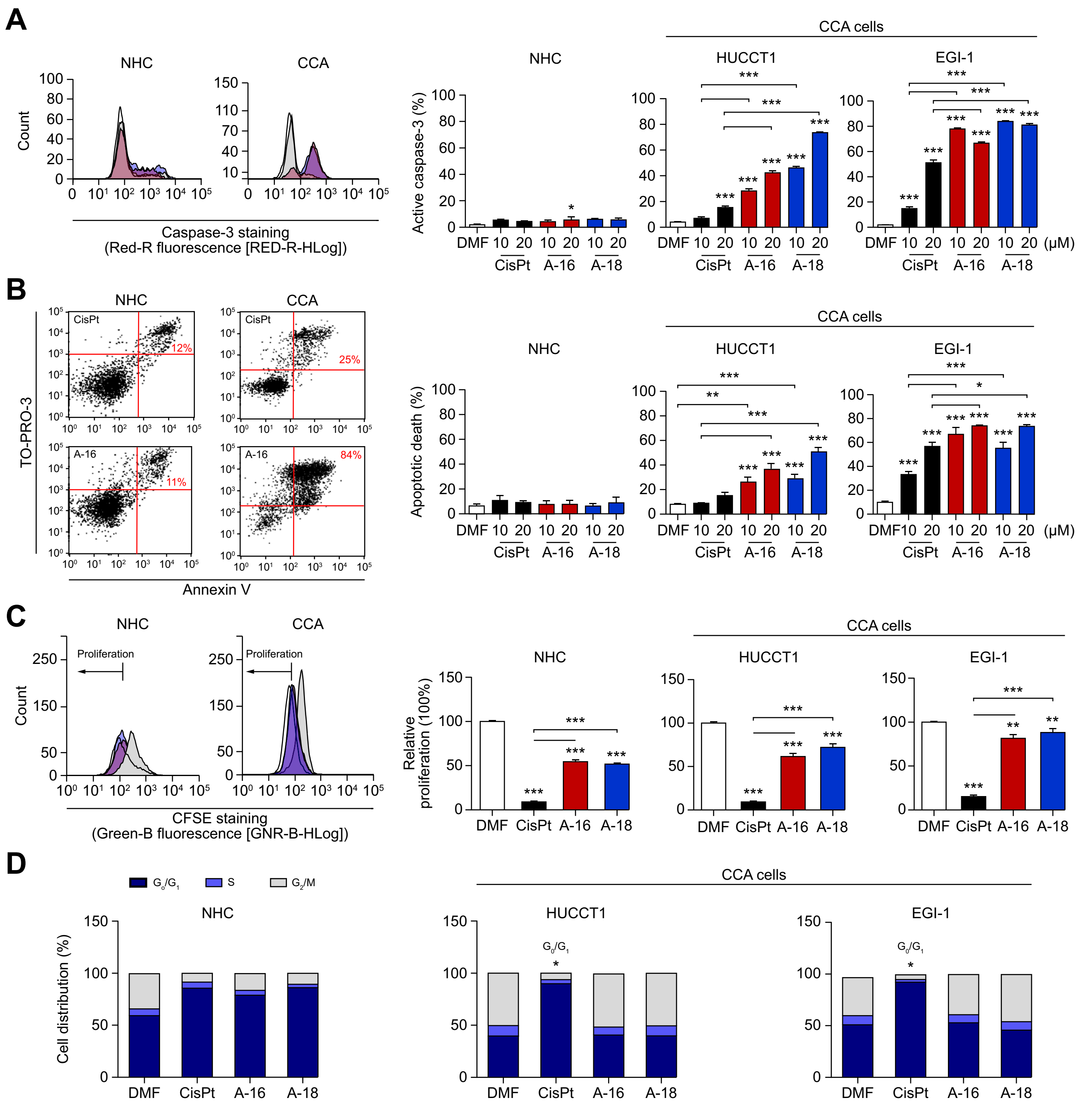

Fig. 4. Pro-apoptotic effects of Aurkines 16 and 18 on human CCA cells. (A) % of cleaved caspase-3^+ cells and (B) Annexin V/TO-PRO^{TM}-3 dual staining of NHCs and CCA cell lines (HUCCT1 and EGI-1) after 48-hour incubation with vehicle, CisPt, Aurkines 16 or 18 (10 µM and 20 µM). (C) Cell proliferation and (D) cell cycle analysis in NHCs and CCA cell lines (HUCCT1 and EGI-1) after 24-hour incubation with vehicle, CisPt, Aurkines 16 or 18 (10 µM). One-way ANOVA test or Student's *t* tests were used. Data are shown as mean ± SEM. *p ≤0.05, **p ≤0.01, ***p ≤0.001. CCA, cholangiocarcinoma; CisPt, cisplatin; DMF, dimethylformamide; NHCs, normal human cholangiocytes.

provided little additional benefit to the pronounced effect of Aurkines (Fig. S5). Besides, treatment with Aurkines 16 and 18 led to a reduction in the proliferation of surviving CCA cells, although this effect was less pronounced than that of CisPt (Fig. 4C). Moreover, CisPt induced cell cycle arrest in the G_0/G_1 phase in both CCA cell lines, associated with p-CDC2 activation, explaining its robust antiproliferative effect on CCA cells.

In contrast, Aurkines 16 and 18 did not induce cell cycle arrest in CCA cells, aligning with their milder effect on CCA cell proliferation. In NHCs, both CisPt and Aurkine compounds induced cell cycle arrest in the G1 phase through p-CDC2 activation (Figs. 4D and S6). Nevertheless, the antiproliferative effect of Aurkines 16 and 18 on NHCs was significantly lower than that of CisPt, suggesting milder pharmacodynamic

interaction of Aurkines on normal cells compared to CisPt (Fig. 4C). Additionally, Aurkines 16 or 18 also reduced the growth of 3D CCA spheroids (Fig. S7A) and cell viability in PDOs (from two patients with CCA) compared to both vehicle and CisPt (Fig. S7B).

To understand the selective toxicity of Aurkines against tumor cells, we investigated the potential role of organic solute export transporters. We observed that the mRNA expression levels of organic solute transporter alpha (*SLC51A*), which encodes the alpha subunit of OST-α/β, a well-characterized exporter of different organic substrates in epithelial cells, were higher in NHCs compared to CCA cells (Fig. 5A). Analysis of publicly available scRNA-seq data from 12 patients with CCA (GSE151530) confirmed the almost complete absence of *SLC51A* (OST-α) expression in CCA cells and other cell types within the tumor (Fig. 5B). Similarly, the expression of *SLC51A* was higher in surrounding liver tissues than in CCA samples (Fig. 5C). Importantly, the role of OST-α/β in NHC resistance to Aurkines was further demonstrated by co-incubating cells with clofazimine (CFZ), an OST-α/β inhibitor, which significantly reduced NHC viability when combined with Aurkines (Fig. 5D). Moreover, differences in DNA packaging between normal and cancer cells may also influence Aurkine efficacy. In fact, the combination of SAHA (suberanilohydroxamic acid), a histone deacetylase pan-inhibitor that induces DNA unpacking, with Aurkines 16 and 18, promoted apoptosis in normal cells (Fig. 5E).

Evaluation of the antitumor effect of Aurkines 16 and 18 on CisPt-resistant human CCA cells

Considering that resistance to CisPt remains a major obstacle in chemotherapy response, our focus shifted to evaluating the therapeutic potential of these novel compounds in overcoming

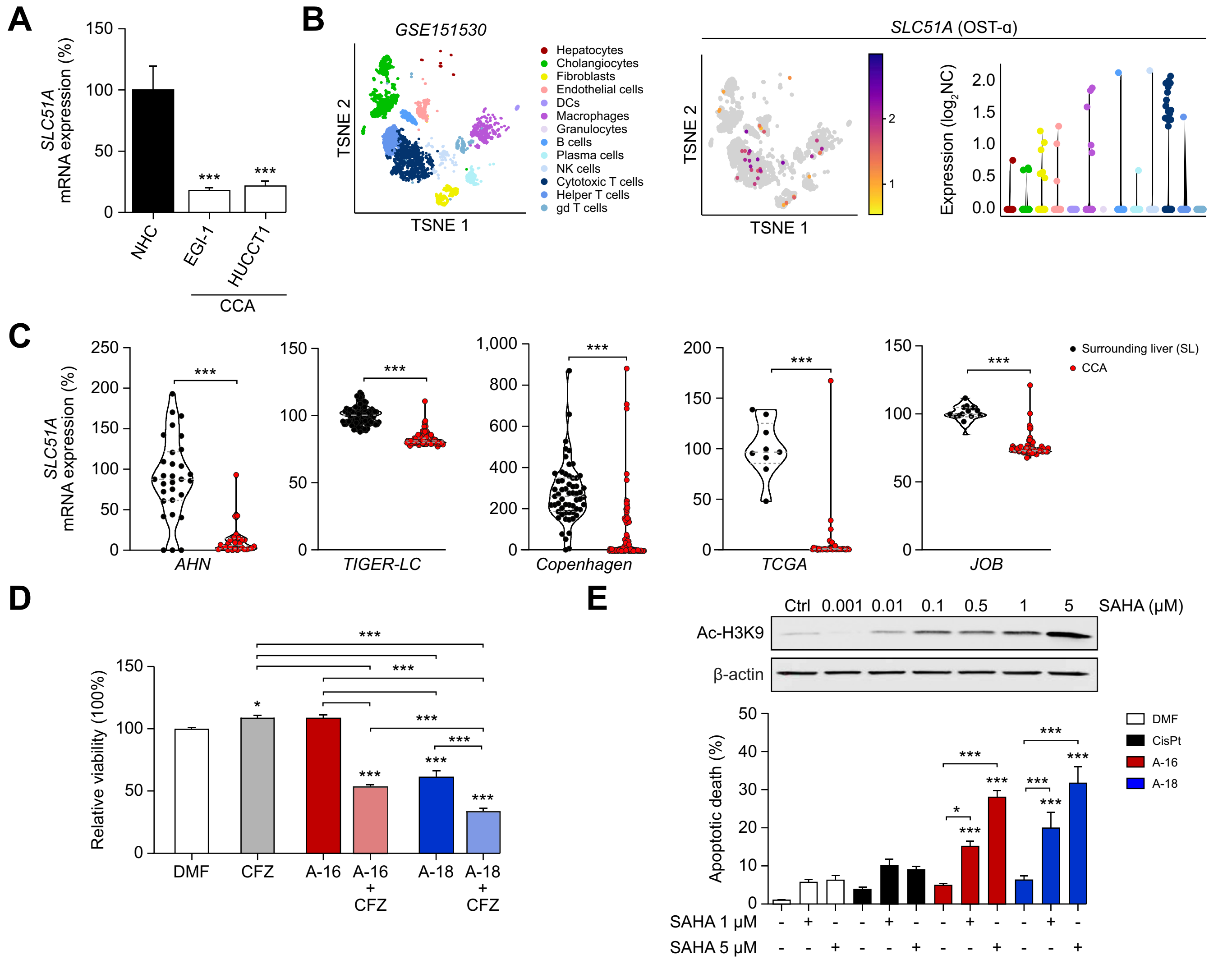

Fig. 5. Mechanisms of NHC resistance to Aurkines. (A) *SLC51A* mRNA expression in NHC and CCA cell lines (HUCCT1 and EGI-1). (B) t-SNE plot clustering all the cell populations detected by scRNA sequencing in the GSE151530 dataset, which includes samples from 12 patients with CCA. (C) *SLC51A* mRNA expression levels in CCA tumors compared to SL tissue from the AHN, TIGER-LC, Copenhagen, TCGA and JOB human cohorts. (D) NHC viability after 48-hour incubation with Aurkines 16 and 18 (20 µM), or their combination with the OST-α/β inhibitor (CFZ, 10 µM). (E) Immunoblot of Ac–H3K9 in NHCs after 48-hour incubation with SAHA, with β-actin as loading control. Apoptosis of NHCs after 48-hour incubation with CisPt, Aurkines 16 and 18 (10 µM), or their combination with SAHA (1 µM and 5 µM). One-way ANOVA test or Student's *t* tests were used. Data are shown as mean ± SEM. *$p \leq 0.05$, **$p \leq 0.01$, ***$p \leq 0.001$. CCA, cholangiocarcinoma; CFZ, clofazimine; CisPt, cisplatin; DMF, dimethylformamide; NHCs, normal human cholangiocytes; OST, organic solute transporter; SAHA, suberoylanilide hydroxamic acid; SL, surrounding liver.

CisPt resistance. To accomplish this, we generated a CCA cell line resistant to CisPt (EGI-1R) by subjecting EGI-1 cells to increasing doses of CisPt, until resistance was validated (Fig. 6A). In contrast to CisPt, both Aurkines 16 and 18 induced pronounced DNA damage in EGI-1R cells (Fig. 6B). Additionally, these compounds led to an increase in intracellular ROS levels compared to CisPt (Fig. 6C). Although Aurkines 16 and 18 did not initiate ROS formation in the mitochondria (Fig. 6D), this could be due to high baseline oxidative stress arising as a consequence of repetitive cycles of CisPt exposure. Consistent with these results, Aurkines 16 and 18 markedly reduced EGI-1R cell viability (Fig. 6E), inducing apoptosis, a response not triggered by CisPt (Fig. 6F,G). In the cells that remained alive, both Aurkines 16 and 18 slightly reduced cell proliferation to the same extent as CisPt (Fig. 6H), despite not causing any cell cycle arrest (Fig. 6I).

Notably, Aurkines 16 and 18 also exhibited a dose-dependent reduction in cell viability in an ovarian cancer cell line and two breast cancer cell lines, all resistant to CisPt (Fig. S8A). Moreover, Aurkines 16 and 18 induced apoptosis in the CisPt-resistant ovarian cancer cell line, a response not observed with CisPt (Fig. S8B).

Evaluation of the effect of Aurkines 16 and 18 on CCA tumor microenvironment

CCA tumors are characterized by an extensive desmoplastic stroma that supports tumor growth and dissemination, contributing to unfavorable patient outcomes and limited treatment response. To explore the potential antitumor effects of Aurkine compounds on the CCA tumor microenvironment (TME), we investigated their impact on CAFs isolated from human CCA tumors – a pivotal component of the TME and a potential mediator of drug resistance. Strikingly, Aurkines 16 and 18 reduced CAF viability and increased their cell death rate (Fig. 6J), while no discernible effects were observed after incubation with CisPt. These findings provide insights into the cytotoxic potential of Aurkine compounds, suggesting their capacity to extend beyond direct effects on cancer cells and influence components of the TME, particularly CAFs.

Analysis of the activity of Aurkines 16 and 18 on CCA tumors *in vivo*

To further investigate the therapeutic potential of Aurkines 16 and 18 in CCA, both compounds were tested in immunodeficient subcutaneous and immunocompetent orthotopic murine CCA models. Initially, Aurkines were administered at a 2 mg/kg dose, a dose known to inhibit CCA tumor growth by CisPt. Although both Aurkines 16 and 18, along with CisPt, noticeably delayed tumor growth compared to the vehicle control group, no statistically significant differences were observed among the treatment groups (Fig. S9A and B). Subsequently, the treatment dose of all compounds was reduced to 0.5 mg/kg, a dosage considered ineffective for CisPt (Fig. 7A). Remarkably, while CisPt showed no therapeutic effect on tumor growth at this reduced dosage, Aurkine 16 halted tumor progression over time (Fig. 7B,C). This was accompanied by a decrease in Ki67$^+$ proliferative cells and an increase in cleaved caspase-3$^+$ apoptotic cells (Fig. 7C). Importantly, no evidence of systemic toxicity was observed (Fig. S9C and D). Notably, in mice subcutaneously injected with CisPt-resistant CCA cells, both Aurkines 16 and 18, at a 2 mg/kg dose, effectively arrested tumor growth, in contrast to vehicle- or CisPt-treated animals (Fig. 7D, E). Furthermore, in an orthotopic CCA model, Aurkine 16 significantly reduced tumor volumes compared to controls (Fig. 7F–H), along with an increase in p-H2AX$^+$ cells (Fig. 7I) and enhanced infiltration of CD8$^+$ and CD4$^+$ T cells into tumors (Fig. 7J). Importantly, no signs of hematological, renal, or hepatic toxicity were observed after treatment with Aurkine (Figs. S10 and S11), a finding further confirmed by toxicological studies with escalating doses in healthy C57BL/6J mice (Figs. S12–14).

Evaluation of the main transporters involved in the uptake of Aurkines 16 and 18 by cancer cells

To elucidate the mechanisms accounting for Aurkines 16 and 18 uptake by cancer cells, cell models with forced expression of plasma membrane transporters involved in the uptake of anionic and cationic drugs by liver cells were used to carry out indirect and direct transport assays. Our observations indicated that Aurkine compounds were transported into the cells by the organic cation transporters, OCT1 and OCT3, the organic anion transporting polypeptide, OATP1A2, and the copper transporter, CTR1 (Figs. 8A,B and S15A). Importantly, none of these transporters, except for CTR1, were found to be involved in the uptake of CisPt (Figs. 8A and S15B). In contrast, OATP1B3 and OATP2B1 did not seem to participate in the transport of Aurkine compounds (Fig. S15A and C). Of note, the expression (mRNA) of *SLC22A3* (OCT3), *SLC22A1* (OCT1), and *SLC31A1* (CTR1) was increased in CCA cell lines compared to NHCs (Figs. 8C and S15D). Among these, scRNA-seq data confirmed that *SLC22A3* (OCT3) is specifically expressed in cholangiocytes within human CCA tumors (Figs. 8D and S16A), regardless of their underlying mutation status (*IDH1, KRAS, TP53*, or wild-type) (Figs. 8E and S16B) or anatomical origin (Figs. 8F,G and S16C,D). Moreover, *SLC22A1* was also found upregulated in CAFs compared to NHCs, which could explain, at least in part, the cytotoxic effect of Aurkines on CAFs (Fig. S15D).

Discussion

Since CisPt was approved in 1978, the range of platinum (Pt)-based cancer therapies has expanded to include seven additional agents, opening alternative treatment options. These drugs have become foundational in oncology, used in approximately 50% of cancer cases, depending on the type and stage of the cancer.[21] However, their clinical utility is often limited by potential side effects and the rapid development of drug resistance.[7] Up to 70% of patients either have intrinsic resistance to CisPt or develop it quickly during treatment, significantly reducing its therapeutic effectiveness.[8] This challenge has spurred extensive research, evidenced by over 12,000 publications in 2023 alone, underscoring the urgent need for new strategies to overcome CisPt resistance (Source: SCOPUS).

All existing Pt-based chemotherapeutic agents, such as CisPt, oxaliplatin, and carboplatin, operate through a well-

understood mechanism.[8,22] These compounds primarily bind to DNA, inducing single-strand DNA breaks in about 95% of cases.[12,13] However, these breaks are relatively easy for cancer cells to repair, enabling survival and drug resistance. In contrast, interstrand crosslinks that result in double-strand DNA breaks are much more challenging for cancer cells to repair,[15] though they occur in less than 5% of cases.[12,13] To address these limitations, we have designed and synthesized

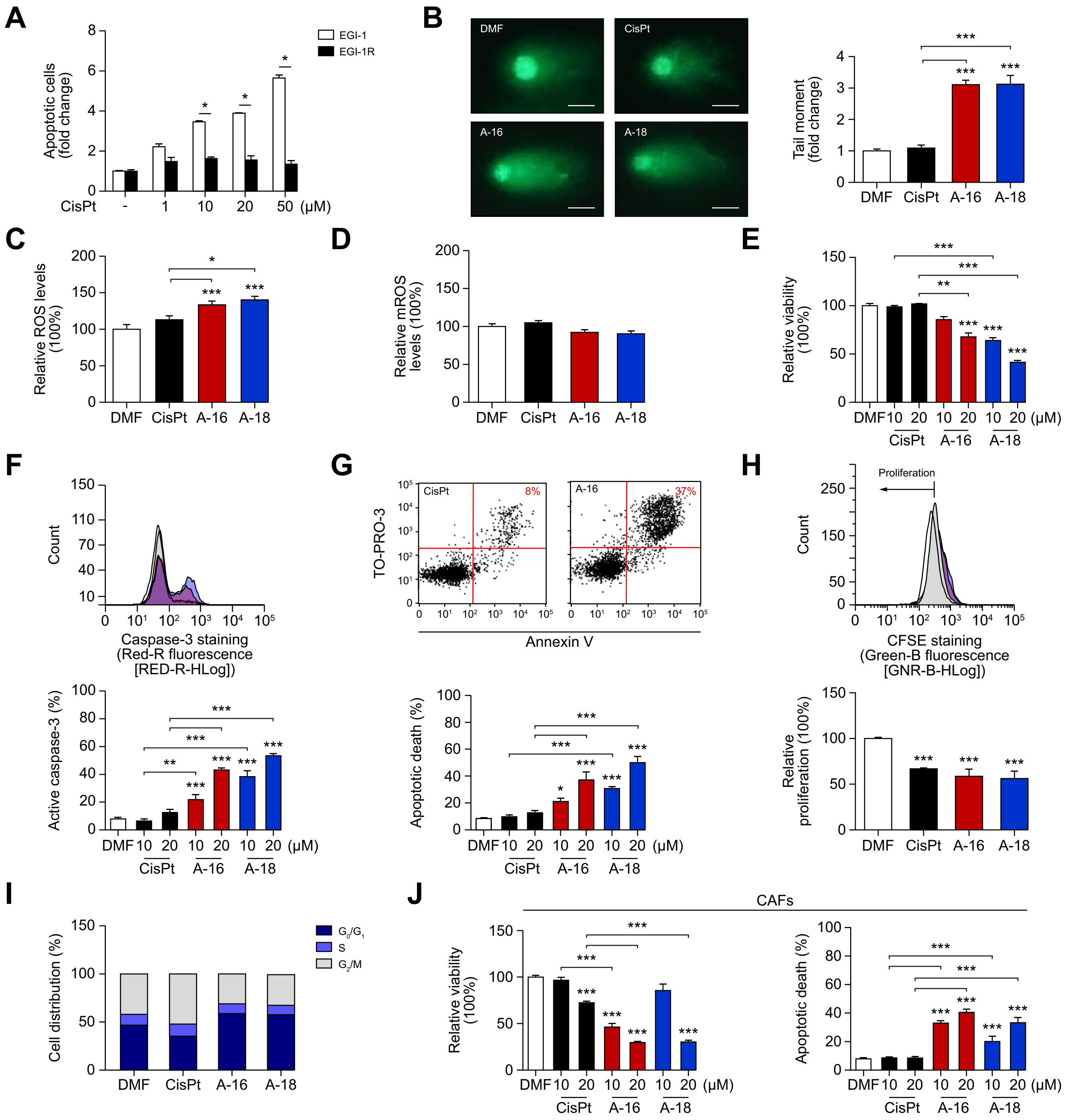

Fig. 6. Antitumor effect of Aurkine 16 and 18 on CisPt-resistant CCA cells (EGI-1R) and CAFs. (A) Apoptosis in EGI-1 and EGI-1R cells after 48-hour incubation with increasing CisPt doses. (B) DNA damage, (C) ROS and (D) mROS in EGI-1R cells after 24-hour incubation with vehicle, CisPt, Aurkines 16 or 18 (10 µM). (E) Cell viability, (F) % of cleaved caspase-3$^+$ cells and (G) Annexin V/TO-PRO^{TM}-3 dual staining of EGI-1R cells after 48-hour incubation with vehicle, CisPt, Aurkines 16 or 18 (10 µM and 20 µM). (H) Cell proliferation and (I) cell cycle analysis in EGI-1R cells after 24-hour incubation with vehicle, CisPt, Aurkines 16 or 18 (10 µM). (J) Cell viability and apoptosis of CAFs after 48-hour incubation with vehicle, CisPt, Aurkines 16 or 18 (10 and 20 µM). One-way ANOVA test or Student's *t* tests were used. Data are shown as mean ± SEM. *p ≤0.05, **p ≤0.01, ***p ≤0.001. CAFs, cancer-associated fibroblasts; CCA, cholangiocarcinoma; CisPt, cisplatin; DMF, dimethylformamide; mROS, mitochondrial ROS; ROS, reactive oxygen species.

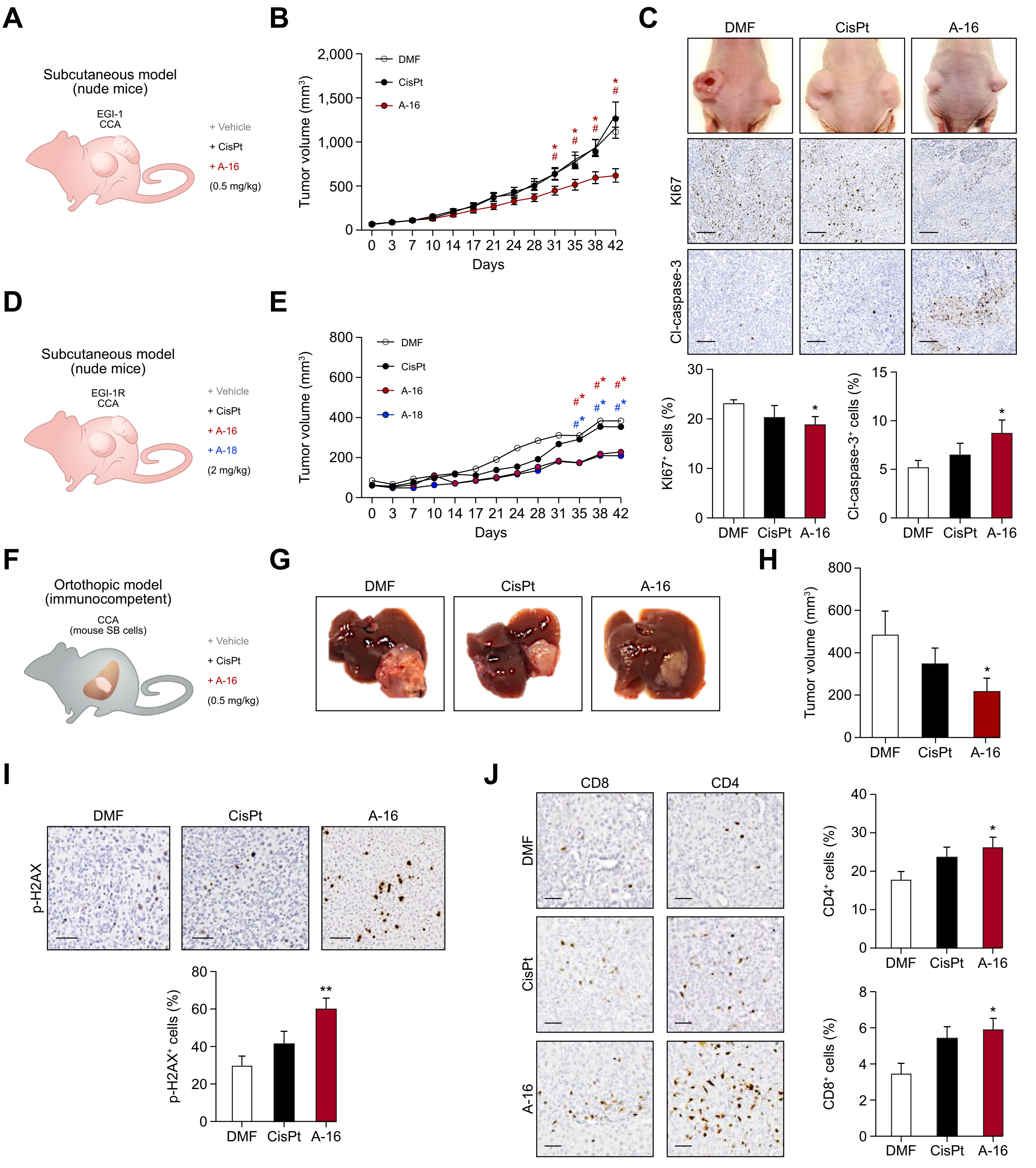

Fig. 7. ***In vivo*** **antitumor activity of Aurkines 16 and 18 in experimental murine models of CCA.** (A) Schematic representation of the subcutaneous CCA model. (B) Tumor volume growth during treatment with CisPt or Aurkine 16 (0.5 mg/kg) in the subcutaneous CCA model. Group sizes: vehicle control (n = 12), CisPt (n = 11), Aurkine 16 (n = 13). (C) Representative tumor images, along with Ki67 and cleaved caspase-3 staining images and quantification in the subcutaneous CCA model. (D) Schematic representation of the subcutaneous CisPt-resistant CCA model. (E) Tumor volume growth during treatment with CisPt, Aurkines 16 and 18 (2 mg/kg) in the subcutaneous CisPt-resistant CCA model. Group sizes: vehicle control (n = 13), CisPt (n = 10), Aurkine 16 (n = 10), Aurkine 18 (n = 16). (F) Schematic representation of the orthotopic CCA model. (G) Representative macroscopic images of liver tumors from vehicle-, CisPt-, and Aurkine 16-treated animals. (H) Tumor volume at sacrifice following treatment with CisPt and Aurkine 16 (0.5 mg/kg) in the orthotopic CCA model. Group sizes: vehicle control (n = 16), CisPt (n = 16), Aurkine 16 (n = 16). (I) Representative images and quantification of p-H2AX staining in the orthotopic CCA model. (J) Representative images and quantification of CD4 and CD8 staining in the orthotopic CCA model. One-way ANOVA test or Student's *t* test were used. Data are shown as mean ± SEM. *p* values (B, E): #*p* <0.05, compared to vehicle-treated; **p* <0.05, compared to CisPt-treated. *p* values (C, H-J): **p* ≤0.05, ***p* ≤0.01, ****p* ≤0.001. Scale bar: 100 μm. CCA, cholangiocarcinoma; CisPt, cisplatin; DMF, dimethylformamide.

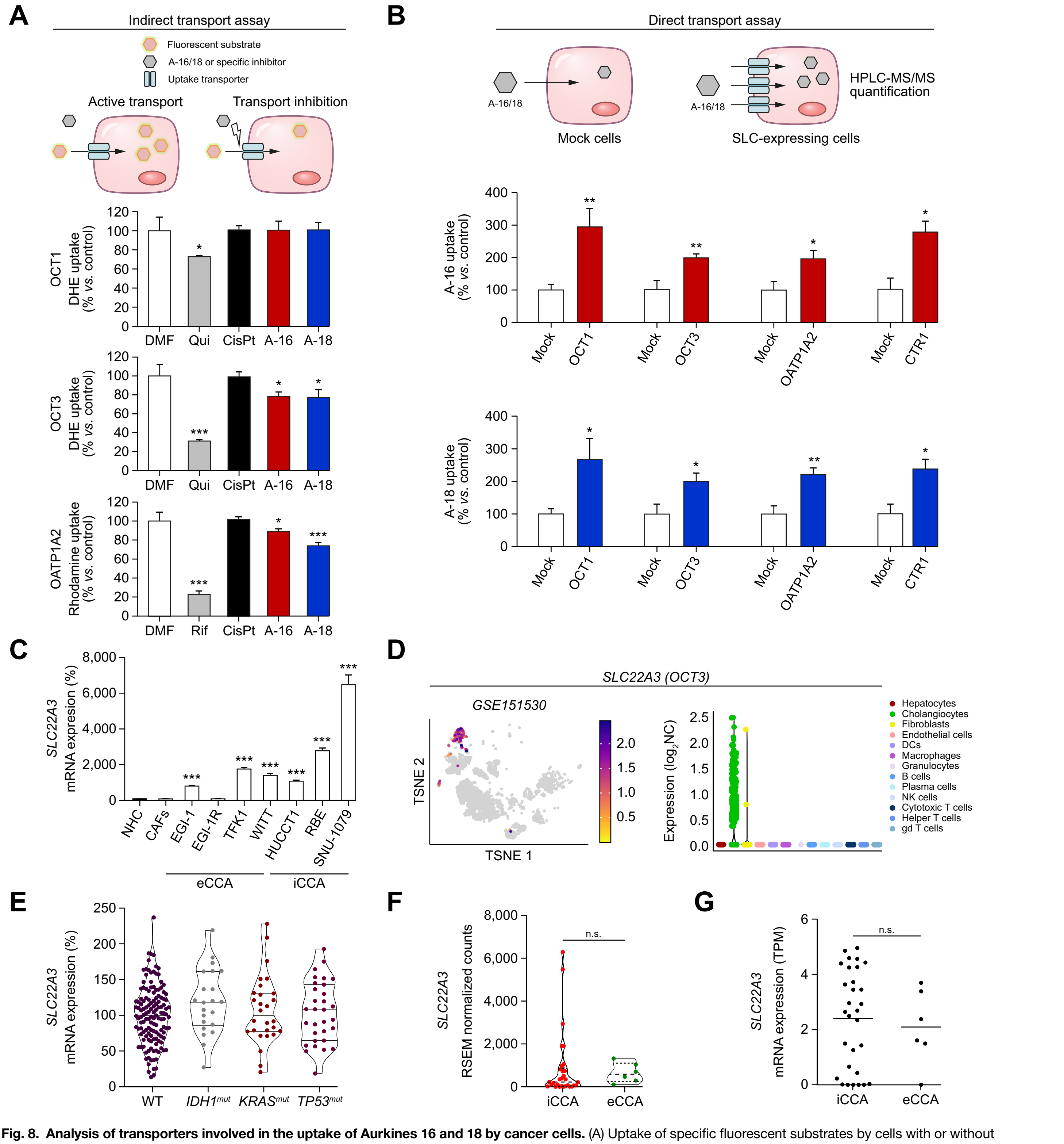

Fig. 8. Analysis of transporters involved in the uptake of Aurkines 16 and 18 by cancer cells. (A) Uptake of specific fluorescent substrates by cells with or without experimental overexpression of each transporter, measured by flow cytometry. (B) Intracellular accumulation of Aurkine 16 and 18 in cells with or without overexpression of each transporter, measured by HPLC-MS/MS. (C) Relative mRNA expression (qPCR) of *SLC22A3* in NHCs, CAFs, eCCA (*i.e.* EGI-1, EGI-1R, TFK1, WITT) and iCCA (*i.e.* HUCCT1, RBE and SNU-1079) cells. (D) Expression levels of *SLC22A3* at the single-cell level in human CCA tumors. (E) mRNA expression of *SLC22A3* in CCA tissues, stratified by mutational status (*IDH1, KRAS, TP53* or WT). (F) mRNA expression of *SLC22A3* in CCA tissues, stratified by anatomical origin (iCCA *vs.* eCCA). (G) mRNA expression of *SLC22A3* in CCA cell lines, stratified by anatomical origin (iCCA *vs.* eCCA). One-way ANOVA test or Student's *t* tests were used. Data are shown as mean ± SEM. *$p \le 0.05$, **$p \le 0.01$, ***$p \le 0.001$. CisPt, cisplatin; CTR, copper transporter; DCs, dendritic cells; DMF, dimethylformamide; eCCA, extrahepatic cholangiocarcinoma; iCCA, intrahepatic cholangiocarcinoma; NHCs, normal human cholangiocytes; OATP, organic anion transporting polypeptide; OCT, organic cation transporter; Qui, quinine; Rif, rifampicine; WT, wild-type. This figure was partly generated using Servier Medical Art, provided by Servier, licensed under a Creative Commons Attribution 3.0 unported license.

two novel chemotherapeutic agents, Aurkine 16 and Aurkine 18. These innovative compounds feature marked poly-electrophilic properties that promote up to three nucleophilic substitution reactions, thus enhancing formation of interstrand DNA crosslinks, leading to increased double-strand DNA breaks. This approach hampers the activation of DNA repair mechanisms, resulting in greater cancer cell death and a reduced likelihood of resistance. The synthesis of Aurkines 16 and 18 is efficient and cost-effective, starting with readily available reagents and producing high yields, ensuring scalability and resource efficiency. The synthesis process is robust, supporting further exploration and optimization of these compounds. The purification of the final products is straightforward, avoiding complex and costly procedures, enabling synthesis on a gram scale and beyond.

Our molecular studies, coupled with phosphoproteomic analysis, demonstrate that Aurkines and CisPt operate through distinct mechanisms of action. CisPt mainly induces single-strand DNA breaks, which activate DNA repair pathways, leading to cell cycle arrest and reduced cell proliferation. However, as these single-strand breaks are relatively easy to repair, cells often restore their DNA structure, allowing them to survive and avoid cell death. In contrast, Aurkines induce double-strand DNA breaks, a more challenging form of damage for cancer cells to repair. This results in elevated oxidative stress within the cell and mitochondria, triggering caspase activation and ultimately leading to cell death. Notably, Aurkines have shown cytotoxic effects not only in CisPt-sensitive CCA cells but also in CisPt-resistant cancer cells from CCA, ovarian, and breast cancers. This demonstrates their therapeutic potential in overcoming CisPt resistance, a significant challenge in cancer treatment.[23] These findings have been further validated in various subcutaneous and orthotopic cancer mouse models, demonstrating the superior efficacy of Aurkines compared to CisPt.

The lack of selectivity of chemotherapies for tumor cells remains a major problem, often leading to significant side effects. However, our study highlights the pronounced selectivity of Aurkine compounds for cancer cells and CAFs. These compounds demonstrate minimal toxicity to normal cells, likely due to a combination of different mechanisms, including reduced uptake via OCT1, OCT3 and CTR1, increased efflux via OST-α/β, and the presence of a compact chromatin structure that limits Aurkine-DNA interactions. Additionally, toxicological studies in healthy C57BL/6J mice, along with stable body weight in treated animals, showed no apparent signs of hematological, hepatic, or renal toxicity after Aurkine administration. These findings reinforce the potential safety and efficacy of Aurkines for treating CCA. Pending further translational and clinical research, they could offer a safe alternative to conventional chemotherapy with a potentially wider therapeutic window.

Our research also addresses the critical interaction between tumor cells and the stroma, especially in CCA, where the extensive TME can impact cancer progression and chemoresistance.[24] Aurkines have demonstrated superior effectiveness compared to CisPt in reducing the viability of CAFs and inducing their apoptosis. This represents a significant advance, as CAFs are known to be resistant to conventional chemotherapy.[25] By targeting both cancer and stromal cells, Aurkines could disrupt the signals that promote tumor growth, thereby creating a more hostile environment for cancer progression. Furthermore, Aurkines promote the recruitment of $CD4^+$ and $CD8^+$ T cells into the TME, which could potentially enhance the effectiveness of immunotherapy, which warrants further investigation.

In conclusion, this study provides compelling evidence of the significant therapeutic potential of Aurkines 16 and 18, highlighting their importance not only in treating CCA but also in addressing various other cancers. The research reveals their unique mechanism of action, distinguishing them from CisPt. These compounds show promise in treating both naïve and CisPt-resistant tumors – a major challenge in cancer treatment – and have substantial effects on stromal and immune cells within the tumor microenvironment. The notable absence of toxicity further underscores their safety profile, reinforcing their potential as effective treatments for CCA. Collectively, these findings position Aurkine compounds as an innovative therapeutic approach, supporting further scientific and clinical investigation.

Affiliations

[1]*Department of Liver and Gastrointestinal Diseases, Biogipuzkoa Health Research Institute - Donostia University Hospital -, University of the Basque Country (UPV/EHU), Donostia-San Sebastian, Spain;* [2]*Department of Organic Chemistry I, Center of Innovation in Advanced Chemistry (ORFEO-CINQA), Faculty of Chemistry, University of the Basque Country (UPV/EHU) & Donostia International Physics Center (DIPC), Donostia-San Sebastian, Spain;* [3]*National Institute for the Study of Liver and Gastrointestinal Diseases (CIBERehd, "Instituto de Salud Carlos III"), Spain;* [4]*Department of Biochemistry and Molecular Biology, Faculty of Science and Technology, University of the Basque Country (UPV/EHU), Leioa, Spain;* [5]*Experimental Hepatology and Drug Targeting (HEVEPHARM), Institute of Biomedical Research of Salamanca (IBSAL), University of Salamanca, Salamanca, Spain;* [6]*School of Cancer Sciences, University of Glasgow, Glasgow, UK;* [7]*Polimero eta Material Aurreratuak: Fisika, Kimika eta Teknologia & Donostia International Physics Center (DIPC), Donostia-San Sebastian, Spain;* [8]*IKERBASQUE, Basque Foundation for Science, Bilbao, Spain;* [9]*Didactics of Mathematics, Experimental and Social Science, Faculty of Education, Philosophy and Anthropology, University of the Basque Country (UPV/EHU), Donostia-San Sebastian, Spain;* [10]*CIC nanoGUNE (BRTA), Donostia-San Sebastian, Spain;* [11]*SGIker, Advanced Research Facilities, University of the Basque Country (UPV/EHU), Donostia-San Sebastian, Spain;* [12]*Division of Gastroenterology and Hepatology, Mayo Clinic College of Medicine and Science, Rochester, MN, USA;* [13]*Beatson West of Scotland Cancer Centre, Glasgow, UK;* [14]*Department of Medicine, Faculty of Medicine and Nursing, University of the Basque Country (UPV/EHU), Donostia-San Sebastian, Spain;* [15]*Department of Biochemistry and Genetics, School of Sciences, University of Navarra, Pamplona, Spain*

Abbreviations

A, adenine; AFM, atomic force microscopy; CAFs, cancer-associated fibroblasts; CCA, cholangiocarcinoma; CFZ, clofazimine; CisPt, cisplatin; CTR1, copper transporter 1; DCs, dendritic cells; DMF, dimethylformamide; eCCA, extrahepatic CCA; G, guanine; GemCis, gemcitabine + cisplatin; iCCA, intrahepatic CCA; IHC, immunohistochemistry; mROS, mitochondrial ROS; NHCs, normal human cholangiocytes; OATP, organic anion transporting polypeptide; OCT, organic cation transporter; OST, organic solute transporter; PDO, patient-derived organoids; Pt, platinum; Qui, quinine; Rif, rifampicine; ROS, reactive oxygen species; SAHA, suberanilohydroxamic acid; SC, supercoiled; scRNA-seq, single-cell RNA sequencing; SL, surrounding liver; TEM, transmission electron microscopy; TME, tumor microenvironment; WT, wild type.

Financial support

This study was competitively funded by the Department of Health of the Basque Government "Euskadi RIS3" [2019222054, 2020333010, and 2021333003 to JMB and FPC] and Instituto de Salud Carlos III (ISCIII) "PI24/00148" to JMB, co-funded by the European Union. Other funding sources not involved in study design and analysis, decision to publish, or preparation of the article include: Instituto de Salud Carlos III (ISCIII) "PI21/00922" to JMB, "PI23/01565 and PI20/00186" to MJP, and "PI23/01850" to PMR, co-funded by the European Union; ISCIII-FIS grant "PI18/01075" to JMB, co-financed by ERDF (FEDER) Funds from the European Commission; ISCIII "PMP22/00054" to JMB, co-funded by the European Union NextGenerationEU/PRTR; ISCIII "FORT23/00026" to JMB; Sara Borrell CD19/00254 to PMR and Miguel Servet CP22/00073 to PMR; CIBERehd (ISCIII). Spanish Ministry of Economy and Competitiveness (MINECO) "Ramón y Cajal" Program "RYC-2015-17755" to MJP; "Diputación Foral Gipuzkoa" (2020-CIEN-000067-01, 2021-CIEN-000029-04-01 and 2023-CIEN-000008-01 to PMR; 2023-CIEN-000045-01 to MJP; 2024-CIE4-000015-01 to LI-S); Department of Education of the Basque Government [IT-1761-22 to LB]; Department of Health of the Basque Country [2023111017 to MJP; 2022111070 to PMR; 2024111044 and 2020111077 to JMB; 2017111010 to JMB], "Euskadi RIS3" [2022333032, 2023333005 and 2024333004 to JMB], and Elkartek (KK-2020/00008 to JMB; KK-2023/00099 to JMB, MJP); La Caixa Scientific Foundation [HR17-00601 to JMB]; Scientific Foundation of the Spanish Association Against Cancer (AECC23/502 to PMR, POSTD246369IZQU to LI-S, PRYGN246692BANA to JMB); AMMF-The Cholangiocarcinoma Charity (EU/2019/AMMFt/001 to JMB and PMR). PSC Partners US (to JMB) and PSC Supports UK (06119JB to JMB). European Research Council, [FPC (ERC-2020-SyG 951281)]; European Commission [ARG (MSCA 101026616 – SN2DNA)]; Spanish Ministry of Science, Innovation and Universities [FPC, IR. MO-G (MCIN/AEI/PID2019-104772GB-I00 and MCIN/AEI/RED2018-102387-T)]; Basque Government [FPC, IR, ARG,MO-G (IT-1553-22)]; Junta de Castilla y Leon (SA074P20 and SA113/P23 to JJGM); Instituto de Salud Carlos III (ISCIII) "PI22/00526" to JJGM; Department of Education of the Basque Country (IT1584-22 to XL and DdS); Spanish Ministry of Science and Universities (MINECO/FEDER): PID2021-127907NB-I00 to XL and DdS; EU MSCA (101072645 - "nanoremedi") to AMB; Spanish Ministry of Science and Innovation (CEX2020-001038-M/MCIN/AEI/10.13039/501100011033, PID2019-104650GB-C22 and PID2023-147987OB-C32 to AMB) and Basque Government - AZPITEK grant to AMB.

Conflict of interest

FPC is scientific advisor of Quimatryx Ltd (quimatryx.com). Remaining authors have no conflicts of interest to declare related to this manuscript.

Please refer to the accompanying ICMJE disclosure forms for further details.

Authors' contributions

FPC and JMB conceived and coordinated the project. IR and MOG completed the chemical synthesis of the compounds. ARG, DdS and XL performed the computational studies. AEL, AMB, AMA and TM performed the AFM and TEM experiments. IR and IO arranged the organization of chemical and biological data. IO, PO, FJCC, NPT, MTG, MHI, CR and PMR completed the functional experiments. MA, OB, EH and JJGM performed the transport assays. AL and BV performed the bioinformatic analysis. IO, MOG, PO, FJCC, NPT, MTG, AL, BV, MA, ARG, MHI, CR, OB, LIS, PMR, MJP, LB, JJGM, FPC, and JMB completed the experiments as well as the collection, analysis and interpretation of data. IO, FPC and JMB wrote the paper with contributions of all the authors. All authors read and critically revised the manuscript and approved the final version. PMR, MJP, LB, JJGM, FPC, and JMB obtained funding.

Data availability

Phosphoproteomic data have been deposited in the ProteomeXchange Consortium via the PRIDE (PRoteomics IDEntifications Database) repository under the identifier PXD061935. Single-cell transcriptome data from cholangiocarcinoma tumors were downloaded from the Gene Expression Omnibus (GEO) dataset under accession number GSE151530. Data from the DONG database (OEP001105) were retrieved from the Biosino NODE repository (https://www.biosino.org/node/project/detail/OEP001105). Batch-corrected gene expression data from the DepMap 24Q4 dataset (https://depmap.org/portal) were analyzed for intraductal papillary neoplasm of the bile duct CCA cell lines. Transcriptomic analyses were performed on CCA tumors and adjacent non-tumor liver tissues using publicly available datasets from five independent patient cohorts: AHN (GSE107943), The Thailand Initiative in Genomics and Expression Research (TIGER-LC; GSE76297), Copenhagen (GSE26566), Cancer Genome Atlas (TCGA-CHOL) and JOB (E-MATB-6389). All datasets used in this study are publicly available and can be accessed via the corresponding repositories.

Acknowledgements

This article stems from collaborations within the COST Actions CA18122 ***European Cholangiocarcinoma Network*** and CA22125 ***Precision-Biliary Tract Cancer-Network***, both supported by COST (European Cooperation in Science and Technology: www.cost.eu). We extend our gratitude to Dr. M. Muñoz Caffarel for providing the commercial breast cancer cell lines. We also thank the Histology Platform of IIS Biogipuzkoa for their technical support in histological analysis and the Hematology Platform of Hospital Donostia for their assistance with the hematological studies in the toxicological assessment. Additionally, we are grateful to Dr. Felix Elortza and Dr. Mikel Azkargorta from the Proteomics Platform of CIC bioGUNE for their help and support with the phosphoproteomics analyses.

Supplementary data

Supplementary data to this article can be found online at https://doi.org/10.1016/j.jhep.2025.04.034.

References

Author names in bold designate shared co-first authorship

[1] Banales JM, Marin JJG, Lamarca A, et al. Cholangiocarcinoma 2020: the next horizon in mechanisms and management. Nat Rev Gastroenterol Hepatol 2020;17:557–588.

[2] Izquierdo-Sanchez L, Lamarca A, La Casta A, et al. Cholangiocarcinoma landscape in Europe: diagnostic, prognostic and therapeutic insights from the ENSCCA Registry. J Hepatol 2022;76:1109–1121.

[3] Valle J, Wasan H, Palmer DH, et al. Cisplatin plus gemcitabine versus gemcitabine for biliary tract cancer. N Engl J Med 2010;362:1273–1281.

[4] Oh D-Y, Ruth He A, Qin S, et al. Durvalumab plus gemcitabine and cisplatin in advanced biliary tract cancer. NEJM Evid 2022;1:1–11.

[5] **Kelley RK**, **Ueno M**, Yoo C, et al. Pembrolizumab in combination with gemcitabine and cisplatin compared with gemcitabine and cisplatin alone for patients with advanced biliary tract cancer (KEYNOTE-966): a randomised, double-blind, placebo-controlled, phase 3 trial. The Lancet 2023;401:1853–1865.

[6] Dasari S, Bernard Tchounwou P. Cisplatin in cancer therapy: molecular mechanisms of action. Eur J Pharmacol 2014;740:364–378.

[7] Oun R, Moussa YE, Wheate NJ. Correction: the side effects of platinum-based chemotherapy drugs: a review for chemists. Dalton Trans 2018;47:7848.

[8] Galluzzi L, Senovilla L, Vitale I, et al. Molecular mechanisms of cisplatin resistance. Oncogene 2012;31:1869–1883.

[9] Kelland L. The resurgence of platinum-based cancer chemotherapy. Nat Rev Cancer 2007;7:573–584.

[10] Rosenberg B, VanCamp L, Trosko JE, et al. Platinum compounds: a new class of potent antitumour agents. Nature 1969;222:385–386.

[11] Lippard SJ. New chemistry of an old molecule: cis-[Pt(NH3) 2Cl2]. Science 1982;218:1075–1082. 1979.

[12] Fichtinger-Schepman AMJ, Lohman PHM, van der Veer JL, et al. Adducts of the antitumor drug cis-diamminedichloroplatinum(II) with DNA: formation, identification, and quantitation. Biochemistry 1985;24:707–713.

[13] Eastman A. Reevaluation of interaction of cis-dichloro(ethylenediamine)platinum(II) with DNA. Biochemistry 1986;25:3912–3915.

[14] Fichtinger-Schepman AM, van Oosterom AT, Lohman PH, et al. cis-Diamminedichloroplatinum(II)-induced DNA adducts in peripheral leukocytes from seven cancer patients: quantitative immunochemical detection of the adduct induction and removal after a single dose of cis-diamminedichloroplatinum(II). Cancer Res 1987;47:3000–3004.

[15] Deans AJ, West SC. DNA interstrand crosslink repair and cancer. Nat Rev Cancer 2011;11:467–480.

[16] Rocha CRR, Silva MM, Quinet A, et al. DNA repair pathways and cisplatin resistance: an intimate relationship. Clinics 2018;73:1–10.

[17] Olaizola P, Lee-Law PY, Fernandez-Barrena MG, et al. Targeting NAE1-mediated protein hyper-NEDDylation halts cholangiocarcinogenesis and impacts on tumor-stroma crosstalk in experimental models. J Hepatol 2022;77:177–190.

[18] Rivilla I, Aparicio B, Bueno JM, et al. Fluorescent bicolour sensor for low-background neutrinoless double β decay experiments. Nature 2020;583:48–54.
[19] de Cózar A, Larrañaga O, Bickelhaupt FM, et al. New insights into the reactivity of cisplatin with free and restrained nucleophiles: microsolvation effects and base selectivity in cisplatin–DNA interactions. ChemPhysChem 2016;17:3932–3947.
[20] Larrañaga O, de Cózar A, Cossío FP. Mono- and Di-Alkylation processes of DNA bases by nitrogen mustard mechlorethamine. ChemPhysChem 2017;18:3390–3401.
[21] Gandin V, Hoeschele JD, Margiotta N. Special issue "cisplatin in cancer therapy: molecular mechanisms of action 3.0.". Int J Mol Sci 2023;24:7917.
[22] Romani AMP. Cisplatin in cancer treatment. Biochem Pharmacol 2022;206.
[23] Marin JJG, Lozano E, Herraez E, et al. Chemoresistance and chemosensitization in cholangiocarcinoma. Biochim Biophys Acta Mol Basis Dis 2018;1864:1444–1453.
[24] **Rodrigues PM**, **Olaizola P**, Paiva NA, et al. Pathogenesis of cholangiocarcinoma. Annu Rev Pathol 2021;16:433–463.
[25] **Vita F**, **Olaizola I**, Amato F, et al. Heterogeneity of cholangiocarcinoma immune biology. Cells 2023;12.

Keywords: Cancer; Chemotherapy; Chemoresistance; DNA damage.
Received 11 October 2024; received in revised form 27 March 2025; accepted 22 April 2025; available online 3 May 2025

Journal of Hepatology, Volume *83*

Supplemental information

New platinum derivatives selectively cause double-strand DNA breaks and death in naïve and cisplatin-resistant cholangiocarcinomas

Irene Olaizola, Mikel Odriozola-Gimeno, Paula Olaizola, Francisco J. Caballero-Camino, Noelia Pastor-Toyos, Mireia Tena-Garitaonaindia, Ainhoa Lapitz, Beatriz Val, Amanda R. Guimaraes, Maitane Asensio, Maider Huici-Izagirre, Colin Rae, David de Sancho, Xabier Lopez, Pedro M. Rodrigues, Elisa Herraez, Oscar Briz, Laura Izquierdo-Sanchez, Aitziber Eleta-Lopez, Alexander M. Bittner, Ana Martinez-Amesti, Teresa Miranda, Sumera I. Ilyas, Chiara Braconi, Maria J. Perugorria, Luis Bujanda, Iván Rivilla, Jose J.G. Marin, Fernando P. Cossío, and Jesus M. Banales

Supplementary information – materials and methods

New platinum derivatives selectively cause double-strand DNA breaks and death in naïve and cisplatin-resistant cholangiocarcinomas

Irene Olaizola, Mikel Odriozola-Gimeno, Paula Olaizola, Francisco J. Caballero-Camino, Noelia Pastor-Toyos, Mireia Tena-Garitaonandia, Ainhoa Lapitz, Beatriz Val, Amanda R. Guimaraes, Maitane Asensio, Maider Huici-Izagirre, Colin Rae, David de Sancho, Xabier Lopez, Pedro M. Rodrigues, Elisa Herraez, Oscar Briz, Laura Izquierdo-Sanchez, Aitziber Eleta-Lopez, Alexander M. Bittner, Ana Martinez-Amesti, Teresa Miranda, Sumera I. Ilyas, Chiara Braconi, Maria J. Perugorria, Luis Bujanda, Iván Rivilla, Jose J.G. Marin, Fernando P. Cossio, Jesus M. Banales

Table of contents

Supplementary materials and methods 2

Supplementary figures 19

Supplementary tables 39

Supplementary references 42

Supplementary materials and methods

Cell cultures

Normal human cholangiocytes (NHC) were isolated from healthy liver tissue as previously detailed by our group.[1,2] Three human CCA cell lines were used: HUCCT1 (intrahepatic CCA sensitive to CisPt), EGI-1 (extrahepatic CCA sensitive to CisPt), and EGI-1R (extrahepatic CCA resistant to CisPt). The EGI-1R cell line was developed at the Biogipuzkoa Health Research Institute (BHRI, Spain) by subjecting EGI-1 cells to increasing CisPt concentrations, ranging from 5 μM to 30 μM, over a 48-hour period until resistance was effectively acquired and confirmed. Notably, to sustain the acquired resistance, CisPt (30 μM) was added to cells every two days. NHC, EGI-1 and EGI-1R cells were grown in fully-supplemented DMEM/F-12 medium as previously described,[2] whereas HUCCT1 cells were cultured in RPMI medium supplemented with 10% fetal bovine serum (FBS; Gibco) and 1% penicillin/streptomycin (P/S; Gibco). Cancer-associated fibroblasts (CAFs) were isolated from resected intrahepatic CCA at BHRI (Spain), purified, and cultured in DMEM 1X supplemented with 10% FBS and 1% P/S as previously described.[3] CAF phenotype was characterized analyzing the expression of the positive markers platelet derived growth factor receptor β (PDGFRβ), fibroblast activated protein 1 (FAP1), α-smooth muscle actin (α-SMA) and vimentin. Cytokeratin 19 (CK19) was used as a negative control (positive marker of cholangiocytes).[3] CCA cells (*i.e.,* EGI-1) and hepatic stellate cells (*i.e.,* LX2), were used as controls. Liver tissue was obtained according to the guidelines approved by the Ethics Committee of the hospital and prior signature of the pertinent written informed patient consent. Besides, Chinese hamster ovary (CHO) cells and a liver cancer cell line (HepG2) were used for the transport assays. CHO cells were grown in DMEM medium supplemented with 1% GlutaMAX™ and 0.43 mM L-proline, while HepG2 cells were cultured in DMEM medium supplemented with 2.2 g/L

sodium bicarbonate and 110 mg/L sodium pyruvate. All cell lines were tested for mycoplasma and were negative all along the experiments.

Establishment of Patient-derived Organoids (PDOs) from tissues

Human tissues were obtained from patients undergoing surgical resection at Glasgow Royal Infirmary, collected under approval of the Ethical Committee. All tissues were collected with informed consent and pathology reports confirmed the presence of cancer. Patient BB220682 was diagnosed with distal cholangiocarcinoma (T3N2R0) and underwent surgical resection without prior neo-adjuvant systemic treatment. This tumor stage indicates a locally advanced disease with regional lymph node involvement but negative surgical margins. Patient BB220452 had a duodenal adenocarcinoma (T4N2R1) and also underwent surgical resection without prior systemic therapy. This case represents a more aggressive disease with deeper invasion (T4), regional lymph node metastases (N2), and microscopically positive surgical margins (R1), indicating residual tumor presence.

For establishment of organoids, tissue digestion was adapted these two protocols. [4,5] Briefly, tissue was minced on ice using a scalpel and then incubated in digestion solution (collagenase, dispase and Y-27632 in basal medium (Advanced DMEM/F12, supplemented with HEPES buffer, Glutamax, Penicillin/Streptomycin, Amphotericin). Tissue was washed twice with cold basal medium then incubated in TrypLe solution (ThermoFisher Scientific, UK) and DNase I (Merck Life Sciences, UK). After washing in cold basal medium, cells were resuspended in growth factor-reduced Matrigel (Corning, UK) and domes placed in 24-well plates, then covered with organoid medium when Matrigel solidified. Medium was replaced every 3-4 days and organoids passaged by mechanical disaggregation. Presence of tumor cells was confirmed by hematoxylin and eosin (H&E) staining of fixed organoids. For drug screening, organoids were dissociated to single cells by TrypLe, then 10^4 cells in organoid medium containing 2% Matrigel added to Matrigel-coated wells of black-sided clear-bottom 96-well plates. After re-formation of organoids,

drugs were added and 72 hours later cell viability was measured by CellTiter-Blue® Assay (Promega, UK). The cell viability was normalized to that of the controls containing only vehicle. Each drug was tested at least in triplicate and statistical significance ($p<0.05$) was tested using student's t-test.

Transcriptomic analysis of human samples

Transcriptomic analyses were performed on CCA tumors and adjacent non-tumor liver tissues using datasets from five distinct publicly available patient cohorts: AHN (dataset: GSE107943),[6] The Thailand Initiative in Genomics and Expression Research (TIGER-LC; dataset: GSE76297),[7] Copenhagen (dataset: GSE26566),[8] Cancer Genome Atlas (TCGA-CHOL)[9] and JOB (dataset: E-MATB-6389).[10]

GSE107943 provided expression profiling by high-throughput sequencing using Illumina NextSeq 500 (GPL18573) and included 27 paired intrahepatic CCA (iCCA) tumor and adjacent normal liver samples. The TIGER-LC cohort (GSE76297) used microarray expression profiling with the [HTA-2_0] Affymetrix Human Transcriptome Array 2.0 (GPL17586) and contained 90 paired iCCA and adjacent normal liver samples. GSE26566 utilized microarray expression profiling with the Illumina humanRef-8 v2.0 expression beadchip (GPL6104) and included 104 CCA tumor samples and 59 surrounding normal liver samples. RNA sequencing data from TCGA-CHOL were obtained from the FireBrowse portal (Broad Institute of MIT & Harvard, USA; https://gdac.broadinstitute.org/) and comprised 36 CCA tumor samples, including 30 iCCA and 6 extrahepatic CCA (eCCA), which were further classified into 4 perihilar CCA (pCCA) and 2 distal CCA (dCCA). The E-MTAB-6389 dataset, retrieved from https://www.ebi.ac.uk/biostudies/arrayexpress/studies/E-MTAB-6389, contained 78 iCCA tumor samples and 31 non-tumor liver samples.

Data from the Gene Expression Omnibus (GEO) datasets (GSE107943, GSE76297, and GSE26566) were analyzed using GEO2R. The datasets from TCGA and E-MTAB-6389 were exported as batch-corrected, normalized versions for further analysis.

Gene expression and tumor mutational profile analysis

To assess the association between gene expression and tumor mutational profiles, data from the DONG database (OEP001105)[11] available in the Biosino NODE repository (https://www.biosino.org/node/project/detail/OEP001105) were analyzed. Specifically, RNA-seq transcript per million (TPM) values (Analysis ID: OEZ00008243) and whole exome sequencing (WES) data (Analysis ID: OEZ00008242) were used. Gene expression data were compared across four patient subgroups: iCCA tumors with *KRAS* mutation (*$KRAS^{mut}$, $TP53^{wt}$, $IDH1^{wt}$*), iCCA tumors with *TP53* mutation (*$KRAS^{wt}$, $TP53^{mut}$, $IDH1^{wt}$*), iCCA tumors with *IDH1* mutation (*$KRAS^{wt}$, $TP53^{wt}$, $IDH1^{mut}$*), and iCCA tumors wild-type for *KRAS, TP53* and *IDH1* (*$KRAS^{wt}$, $TP53^{wt}$, $IDH1^{wt}$*).

CCA cell line analysis

Batch-corrected gene expression data from the DepMap[12] Public 24Q4 dataset (https://depmap.org/portal) were analyzed for intraductal papillary neoplasm of the bile duct CCA cell lines. Intrahepatic CCA cell lines included SNU1079, HUH28, HUCCT1, KKU055, KKU213, ICC10, ICC106, ICC108, ICC12, ICC137, ICC15, ICC2, ICC3, ICC4, ICC5, ICC6, ICC7, ICC8, ICC9, HKGZCC, OZ, RBE, SG231, SSP25, TKKK, YSCCC, CCLP1, CCSW1, ICC16 and ICC18. Extrahepatic CCA cell lines analyzed were SNU245, SNU1196, EGI1, KKU100, TFK1 and ECC2.

Single-cell RNA sequencing analysis (scRNA-seq) of human iCCA tissue

Single-cell transcriptome profiling from CCA tumors was downloaded from Gene Expression Omnibus (GEO) dataset under accession number GSE151530.[13] This CCA tumor dataset

comprises the transcriptional profile of single cells obtained from 14 fresh liver tumor biopsies from 12 patients with iCCA.

Regarding data processing, the dataset was processed with Seurat R package version 5.1.0 and R version 4.3.3. Quality control metrics were computed, and cells with fewer than 500 detected genes, more than 4,000 detected genes, or over 20% mitochondrial gene expression were excluded. Normalization was performed using the SCTransform method, which regresses out the effects of mitochondrial content and RNA counts to minimize technical variability. Dimensionality reduction was applied to the analyzed data using Principal Component Analysis (PCA). For this, the top 2,000 variable genes were selected using the FindVariableFeatures function (vst method) in Seurat. Based on an elbow plot, the first 30 principal components (PCs) were retained for downstream analyses, including clustering and visualization. Unsupervised clustering was performed using the Leiden algorithm at multiple resolutions. For subsequent analyses, clusters generated at a resolution of 0.4 were selected as optimal. Dimensionality reduction techniques, including UMAP and t-SNE, were applied for visualizing the clusters.

Cell type annotation of clusters was based on the expression of canonical marker genes as follows: Hepatocytes (HPX, LBP, SERPINA10); Cholangiocytes (*KRT19*, *KRT7, FXYD2*); Fibroblasts (*COL1A1, DCN*); Endothelial cells (*FCN2, VWF, CDH5*); Dendritic Cells (DCs) (*IRF8*); Macrophages (*CD68, CD163, CSFIR*); Granulocytes (*FXYD2, IRF8*); B cells (*MS4A1, CD79A*); Plasma cells (*FCRL5, IGHM*); Natural Killer (NK) cells (*KLRD1, GNLY*); Cytotoxic T Cells (*CD3D, TRAC*); Helper T Cells (*IL7R, MAL*); γδ (gd) T cells (*TYMS*).

RNA isolation and gene expression

RNA was extracted from cell cultures using TRI Reagent® (Sigma-Aldrich). Reverse transcription of 1 µg RNA from cell samples was performed utilizing a mixture containing DNAse I Amplification Grade (Invitrogen – Thermo Fisher Scientific), M-MLV Reverse Transcriptase (Invitrogen),

RNaseOUT Recombinant Ribonuclease Inhibitor (Invitrogen – Thermo Fisher Scientific), Random Primers (Invitrogen – Thermo Fisher Scientific), and dNTPs (GE Life Sciences). The gene expression (mRNA) of specific primers sequences (Table S1) was determined by real-time quantitative polymerase chain reaction (qPCR) using iQ SYBR Green Supermix (Bio- Rad) in a CFX96 Touch Real-Time PCR Detection as previously described. Expression of *Glyceraldehyde-3-phosphate dehydrogenase (GAPDH)* was used as a housekeeping control for data normalization and gene expression was determined using the ΔCT method.

Histological analyses

Tissue samples were collected and fixed in 4% paraformaldehyde for 24 hours. Next, tissues were processed using the MTM tissue processor (Slee Medical GmbH), embedded in paraffin (Gibco – Thermo Fisher Scientific) and cut using the HM355S microtome (Gibco – Thermo Fisher Scientific) in sections at a thickness of 4-5 µm.

Immunohistochemistry (IHC) was performed in paraffin-embedded mouse liver tissue or tumoral sections. In order to remove the paraffin, slides were incubated in xylene and rehydrated in graded series of ethanol as previously described. Next, sections were placed on a 0.6% H2O2 (Sigma-Aldrich) in methanol (Applichem Panreac) solution for 15 minutes to block endogenous peroxidases. Following antigen retrieval with antigen unmasking solution (Vector Laboratories), slides were blocked using first the Avidin/Biotin Blocking Kit (Vector Laboratories) and later, a 20% swine bovine serum in DPBS 1X blocking buffer. Primary antibodies (Table S2) were incubated overnight at 4ºC. After washing the antibodies with DPBS 1X, slides were incubated with the appropriate biotinylated secondary antibodies. Vectastain ABC Reagent (Vector Laboratories) followed by 3,3 diaminobenzidine (DAB) peroxidase substrate Kit (Vector Laboratories) was used for antigen visualization. Slides were counterstained with Mayer’s hematoxylin (Sigma-Aldrich), dehydrated and mounted as previously described. Representative pictures were taken in an Axioscan 7 (Zeiss) and images were analysed with QuPath software.

Immunoblotting

Whole cell lysates of cultured human cholangiocytes were extracted using radioimmunoprecipitation (RIPA) lysis buffer. Alterations in protein expression were evaluated through immunoblotting, using 10-30 µg of protein from the cellular extracts, which were separated using 7-12.5% SDS-PAGE and subsequently transferred to a nitrocellulose membrane (Bio-Rad). These membranes were blocked either with 5% BSA/TBS-0.1%Tween for phosphorylated proteins or 5% skim milk powder/TBS-0.1%Tween for non-phosphorylated forms. Membranes were then incubated with the relevant primary antibody (Table S2) overnight at 4ºC. Subsequently, horseradish peroxidase-conjugated secondary antibody was applied to the membranes and incubated for 1 hour at room temperature. Next, antibodies were exposed using the Novex® ECL HRP Chemiluminescent Substrate Reagent Kit (Invitrogen), and the emitted chemiluminescence was visualized and captured in the iBright CL1500 Imaging System (Invitrogen – Thermo Fisher Scientific). Protein signal quantification was performed using ImageJ (National Institutes of Health, USA).[14] β-actin protein levels were utilized to normalize protein loading.

Cell viability, proliferation, cell cycle and apoptosis

Cell viability was assessed using the Cell Proliferation WST-1 Assay (Roche) according to the manufacturer's instructions. Cells were seeded at a density of $2.5x10^3$ cells per well in a collagen-coated 96-well plate in their respective culture media and incubated overnight at 37ºC. The next day, cells were exposed to two different concentrations (10 µM and 20 µM) of CisPt, Aurkines, or the vehicle solution for 48 hours. Finally, 10 µL of WST-1 reagent were added to each well, incubated at 37ºC for 1 hour and the signal was measured at 450 nm in a Halo LED 96® multiplate reader (Dynamic Scientific Ltd., UK).

Early apoptosis was evaluated by measuring caspase-3 activity (PhiPhiLux®-G_2D_2 kit, Oncoimmunin) through flow cytometry. Cells were seeded at a density of $2.5x10^4$ cells per well in a collagen-coated 24-well plate. After 24 hours, cells were incubated with two concentrations (10 μM and 20 μM) of CisPt, Aurkines, or the vehicle solution for 48 hours. Subsequently, cells were collected and stained with a substrate solution (10 μM) for 45 minutes at 37ºC. Fluorescence was then measured by flow cytometry using the *Guava easyCyte 8HT* Flow Cytometer (Merck Millipore).

Late apoptosis was evaluated using FITC Annexin V (BioLegend) and TO-PRO™-3 iodide (Invitrogen Thermo Fisher Scientific) by flow cytometry. Cells were seeded at a density of $2.5x10^4$ cells per well in a collagen-coated 24-well plate. After 24 hours, cells were incubated with two concentrations (10 μM and 20 μM) of CisPt, Aurkines, or the vehicle solution for 48 hours. Subsequently, cells were collected and stained with FITC Annexin V (BioLegend) for 15 minutes at room temperature and TO-PRO™-3 iodide (Invitrogen – Thermo Fisher Scientific) for 15 minutes at 4ºC. Fluorescence was measured by flow cytometry using the *Guava easyCyte 8HT* Flow Cytometer (Merck Millipore). Puromycin (2 μg/mL) was used as a positive control for cell death.

Cell proliferation rates were determined through flow cytometry, using the CellTrace™ CFSE Cell Proliferation Kit (Invitrogen – Thermo Fisher Scientific) in accordance with the manufacturer's instructions. Briefly, $2x10^4$ cells were collected, suspended in a 0.1% BSA in PBS solution, and labelled with CFSE at a 5 mM concentration. Afterwards, 5 volumes of cold medium were added, and the tubes were incubated for 5 minutes on ice. Subsequently, cells were washed three times with cold media, resuspended in their respective culture media, and seeded in a collagen-coated 12-well plate. The cells were allowed to attach overnight, followed by incubation with CisPt, Aurkines or vehicle solution (10 μM) for 24 hours (the vehicle solution was used as control). The cells were then trypsinized, centrifuged, and resuspended in DPBS 1X (Gibco – Thermo Fisher

Scientific) before being placed in a U-bottom 96-well plate, following the flow cytometer manufacturer's instructions. Fluorescence was measured using the *Guava easyCyte 8HT* Flow Cytometer (Merck Millipore).

The cell cycle distribution was assessed through flow cytometry utilizing TO-PRO™-3 iodide (Invitrogen – Thermo Fisher Scientific). Approximately $1x10^5$ cells were harvested, washed with DPBS 1X, and fixed with ice-cold 70% ethanol overnight at -20ºC. After centrifugation at 8,000 rpm for 5 minutes, the cell pellets were stained with a DPBS 1X solution containing TO-PRO™-3 iodide (Invitrogen – Thermo Fisher Scientific) and RNAse A (Sigma), followed by a 30-minute incubation at 37ºC in the dark. Subsequently, the samples were transferred to a U-bottom 96-well plate following the manufacturer's instructions and analyzed using the *Guava easyCyte 8HT* Flow Cytometer (Merck Millipore).

Reactive oxygen species (ROS) detection

To study cellular oxidative stress, the CellROX™ Deep Red Reagent (Invitrogen – Thermo Fisher Scientific) was utilized following the manufacturer's guidelines. Briefly, $2x10^5$ cells were seeded overnight in thin collagen-coated 12-well plates with their corresponding culture media. The next day, cells were incubated with CisPt, Aurkines, or the vehicle solution (10 µM) for 24 hours. Subsequently, cells were trypsinized and incubated with 25 µM CellROX™ Deep Red Reagent (Invitrogen) for 1 hour at 37ºC. Fluorescence was then measured using the *Guava easyCyte 8HT* Flow Cytometer (Merck Millipore).

To evaluate mitochondrial oxidative stress, the MitoSOX™ Red Reagent (Invitrogen) was employed following the manufacturer's instructions. Initially, $2x10^5$ cells were seeded overnight in thin collagen-coated 12-well plates with their corresponding culture media. The next day, cells were incubated with CisPt, Aurkines, or the vehicle solution (10 µM) for 24 hours. After this incubation period, the medium was removed, and cells were incubated with 1 µL of MitoSOX

reagent per well in 250 µL of their respective culture media for 20 minutes at 37ºC. Subsequently, cells were trypsinized, washed twice with DPBS 1X, and finally transferred to a U-bottom 96-well plate for analysis using the *Guava easyCyte 8HT* Flow Cytometer (Merck Millipore).

3D Spheroids

To generate CCA spheroids, $3x10^3$ human CCA cells were seeded in 50 µL of complete spheroid medium (DMEM F12 + Glutamax + P/S + EGF + B27 + Insulin) in a 96-well U-bottom plate (Greiner bio-one). The plate was then centrifuged at 400 g for 10 minutes and maintained at 37ºC with 5% CO2 for 24 hours to allow spheroid formation. The following day, once the spheroids had formed, 50 µL of normal medium (DMEM-F12 + P/S + Glutamax) was added to each well. Subsequently, CisPt or Aurkines at a concentration of 10 µM (or vehicle solution as control) were added in 100 µL of medium to each well. Calcein was used to stain the spheroids. The size of the CCA spheroids was measured at baseline (0 hours) and 48 hours after treatment using ImageJ software version 1.50 (NIH, Bethesda, MA, USA), and photographs were taken during the process using the Axio Observer 7 fluorescent microscope.

Atomic Force Microscopy and Transmission Electron Microscopy

DNA samples were prepared from lyophilized lambda phage DNA, methylated, from *E. coli* host strain W3110 (Mw. 31.5 x 10^3 kDa, 48 kb, from Sigma-Aldrich). DNA solutions (0.167 mg mL^{-1} in (N-morpholino)propanesulfonic acid, MOPS, 10 mM) were prepared. These samples were deposited on a freshly cleaned silicon oxide surface for AFM, and on a freshly cleaned and glow discharged carbon grid for TEM. The silicon wafer (100 orientation and thickness 0.52 mm) was cut into pieces by a wafer dicing saw (Disco DAD321). The pieces were thoroughly cleaned by sonication in an ultrasonicator (VWR Ultrasonic Cleaner) for 5 min in a sequence of four solvents: isopropanol (LC-MS chromasolv®, Sigma-Aldrich), acetone (ACS reagent > 99.5 %, Sigma-Aldrich), ethanol (absolute, Sigma-Aldrich) and water (18 M *Ohm* cm, < 5 ppb total organic content,

Millipore). Afterwards, the wafer was freshly cleaned with an oxygen plasma (Femto plasma system, Diener).

A droplet of DNA solution was deposited on the substrate (2 μL for carbon grid and 5 μL for silicon wafer), softly blown with a nitrogen stream (focused to the solid-liquid air line) and dragged along until it evaporated. Then, a droplet between 2-5 μL of a 10 μM Pt (II) compound solution in 10 mM MOPS was deposited on the DNA/carbon grid or DNA/silicon sample. The Pt(II) incubation time varied between 2 and 10 min. The samples were dried with a nitrogen stream. DNA surface topographies were imaged with an atomic force microscopy (Agilent AFM 5500) in air, in AC mode with an oscillation frequency of 63 kHz. The images were obtained at 512 points per lines at 0.5-1 lines/second. Topography images were flattened, and profiles were analyzed with Gwyddion 2.55 (www.gwyddion.net). For TEM analysis, DNA was imaged by high-angle annular dark-field scanning transmission electron microscopy (HAADF-STEM), with a TECNAI G2 20 TWIN apparatus, equipped with a LaB6 filament, operated at 120 kV.

pUC18 plasmid mobility

The pUC18 plasmid (Thermo Fisher, ref. SD0051) was obtained from a transfected *E. coli* clone and purified using Miniprep (Qiagen). A total of 300 ng of DNA was incubated in 25 μL of 10 mM Tris pH 8.0 with 50 μM of the drug at 37°C for 1 hour. Subsequently, agarose gel electrophoresis was performed using a 1% agarose gel containing SYBR™ Safe DNA Gel Stain (Thermo Fisher, ref. S33111) in TAE buffer (40 mM Tris-HCl, 1 mM EDTA, 20 mM acetic acid, pH 8.0) at 80 V for 1 hour.

Comet assay

Comet slides (Abcam) were prepared using the following procedure. Initially, comet agarose (Abcam) was heated in a water bath at 90-95ºC until liquefied, and then cooled down to 37ºC for 20 minutes. Subsequently, 75 μL of comet agarose was added per well onto the 3-well comet slide

to create a base layer and incubated at 4ºC for 15 minutes. Cells, whether incubated with vehicle solution, CisPt, or Aurkines at 10 μM, were then trypsinized, collected, and centrifuged at 700 *g* for 2 minutes. The supernatant was then discarded, and cells were resuspended to $1x10^5$ cells/mL in ice-cold PBS. Cell samples were then mixed with comet agarose (1/10 ratio) and 75 μL of this mixture was added per well onto the comet agarose base layer. After a 15-minute incubation at 4ºC, the slide was first placed in Lysis Buffer for 45 minutes at 4ºC in the dark, and then in Alkaline Solution for 30 minutes at 4ºC in the dark. The slide was then placed within an electrophoresis chamber with cold alkaline electrophoresis solution, at 300 mA and 1 volt/cm for 30 minutes. After the electrophoresis step, the slide was rinsed in dH_2O three times, treated with cold 70% ethanol for 5 minutes, and air-dried. Finally, the slide was stained with Vista Green DNA Dye for 15 minutes at room temperature. The stained slide was then observed using a confocal microscope, specifically the ZEISS LSM 900 model.

Sample preparation for phosphoproteomic analysis

Approximately 500 μg of protein per sample was processed using the FASP protocol described in the previous section. Phosphopeptides were enriched using the High-Select™ TiO_2 Phosphopeptide Enrichment Kit (Thermo) according to the manufacturer's instructions. The enriched samples were then directly loaded onto the mass spectrometer for analysis.

Mass spectrometry-based high throughput phosphoproteomic analysis

Samples were analyzed using a timsTOF Pro mass spectrometer with PASEF (Bruker Daltonics), coupled online to an Evosep ONE liquid chromatograph (Evosep). Samples were directly loaded onto the Evosep ONE in a solution containing approximately 5% trifluoroacetic acid (TFA) and resolved using the 30 samples-per-day protocol (44-minute runs).

Total protein identification and quantification were performed using DIA-NN software v1.8.1.[15] Database searches were conducted against *Homo sapiens* entries from

UniProt/SwissProt, with precursor and fragment mass tolerances of 20 ppm and 0.05 Da, respectively. Carbamidomethylation of cysteines was set as a fixed modification, while oxidation of methionines and phosphorylation of serine, threonine and tyrosine were considered variable modifications. A false discovery rate (FDR) of <1% at the peptide level was applied as a significance threshold. Proteins not detected with at least two peptides and in at least 75% of samples within at least one experimental group were excluded from further analysis. Protein abundances were log_2-transformed, and missing values were imputed using quantile regression-based imputation (QRILC). The imputed protein quantification data were normalized by quantile normalization in R studio (R version 4.2.1). Group comparisons were conducted by first assessing normality using the Shapiro-Wilk test. If data followed a normal distribution, a *t*-test was performed; otherwise, the Wilcoxon rank-sum test was used. P-values were adjusted for multiple testing using the FDR method.

Phosphopeptide samples were analyzed using MSFragger v4.1 and IonQuant v1.10.27 via FragPipe v22.0,[16] with default settings optimized for phosphoproteomic analysis. Phosphosites not detected in at least 75% of samples within at least one experimental group were excluded. Phosphosite abundances were log_2-transformed, and missing values were imputed using QRILC. Normalization was performed by quantile normalization in R Studio (R version 4.2.1). Phosphorylation site expression levels were compared following the same statistical workflow as total protein analysis: normality was assessed using the Shapiro-Wilk test, followed by a *t*-test for normally distributed data or a Wilcoxon rank-sum test otherwise. P-values were adjusted for multiple testing using the FDR method. Phospho log ratios from comparisons, along with adjusted p-values for all identified phosphosites, were analyzed using QIAGEN Ingenuity Pathway Analysis (QIAGEN IPA).[17]

The mass spectrometry data have been deposited in the ProteomeXchange Consortium via the PRIDE (PRoteomics IDEntifications Database) repository[18] under the identifier PXD061935.

Experimental overexpression of human transporters in cells

To conduct transport assays, the CHO cell line and a human liver cancer cell line (HepG2) were employed. To establish stable monoclonal cell lines expressing human drug transporters, lentiviral transduction was carried out for both CHO and HepG2 cell lines, and cells were subjected to monoclonal selection. Specifically, CHO cells were transduced to express OATP1A2, OATP2B1, OATP1B3, and OCT3, while HepG2 cells were transduced to express OCT1. Single cell clones were obtained by the limited dilution method. As controls, CHO-mock and HepG2-mock cells were utilized.

Indirect transport assay

Cells were plated in a 12-well plate at a density of $2x10^4$ cells per well and cultured for four days. After this period, the cells were trypsinized and suspended first in culture medium and then in PBS. Subsequently, 50 µL of cells were combined with 50 µL of a "transport" medium (containing 96 mM NaCl, 5.3 mM KCl, 1.1 mM KH_2PO_4, 0.8 mM $MgSO_4$, 1.8 mM $CaCl_2$, 11 mM glucose, and 50 mM HEPES at pH 7.40). Within this medium, a specific known fluorescent substrate at a concentration of 1 µM was introduced along with the corresponding specific inhibitor or the Aurkine compound at 10 µM, and the mixture was incubated at 37ºC for 15 minutes (Table S3). The loading phase was concluded by diluting the mixture with 900 µL of ice-cold uptake medium, and the level of intracellular fluorescence was assessed using a FACSCalibur flow cytometer. Propidium iodide staining (5 µg/mL) was applied to identify and exclude dead cells from the data analysis.

Direct transport assay

Cells were initially seeded into 12-well plates at a density of $2x10^4$ cells per well, and the experiments were conducted the following day. The culture medium was replaced with fresh medium containing either Aurkines 16 or 18. After a 60-minute incubation period, the uptake process was halted by washing the plates four times with 1 mL of ice-cold culture medium devoid of FBS. Subsequently, cells were lysed using distilled water supplemented with 5 µM prednisolone (utilized as an internal standard). The concentration of Aurkines in the lysates was quantified using HPLC-MS/MS on a 6420 Triple Quad LC/MS system from Agilent Technologies, Santa Clara, CA, USA. The results were adjusted based on the protein content.

Experimental animal models of CCA

The therapeutic efficacy of Aurkines 16 and 18 was assessed *in vivo*, in subcutaneous xenograft CCA mouse models. All experimental protocols received approval from the Ethical Committee for Animal Experimentation of the BHRI (CEEA18/020, CEEA21/011 and CEEA24/08) and were conducted in accordance with the institution's regulations for the ethical treatment of laboratory animals.

Xenograft CCA models

CCA (EGI-1) cells (1×10^6 cells) and CCA (EGI-1R) cells resistant to CisPt ($5x10^6$)were subcutaneously injected in the dorsal flanks of seven-week-old male immunodeficient CD-1 nude mice (Crl:CD1-Foxn1nu; strain 086, homozygous) (Charles River). Once tumors were well-established (average size: ~50 mm3), mice were homogenously distributed into control and treatment groups (CisPt, Aurkine 16 and Aurkine 18). All of the treatments were intraperitoneally administered once a week during 5 weeks, at 2 mg/kg or 0.5 mg/kg dose. Tumor size were measured using a caliper every 2-3 days during 5 weeks. The formula V = (D x d2)/2 was

employed to calculate tumor volume (V), where "D" means the largest diameter measured and "d" represents the shortest diameter.

Orthotopic CCA model

Orthotopic xenografts were established by injecting 5 × 10^5 CCA (SB) mouse cells, resuspended in 50% Matrigel/saline, into the left lobule of the liver of immunocompetent C57BL6/J mice following laparotomy, as previously described.[19] One week later, mice were randomized into control and treatment groups (CisPt or Aurkine 16). Treatments were administered intraperitoneally (0.5 mg/kg) once per week for one month. After 28 days, mice were sacrificed by exanguination. After sacrifice, tumor size was measured with a caliper and tumor volume (V) was calculated using the following formula: $V = (D \times d^2)/2$ (where “D” represents the largest diameter measured and “d” the shortest”.

Toxicological study

Healthy C57BL/6J mice were treated once per week for one month with either vehicle (DMF), CisPt, Aurkine 16, or Aurkine 18 at doses of 0.5 mg/kg (used in the subcutaneous model with CisPt-sensitive CCA cells) and 2 mg/kg (used in the subcutaneous model with CisPt-resistant CCA cells).

A comprehensive panel of hematological markers was measured, including:

- Red blood cell parameters: erythrocytes, hemoglobin, hematocrit, mean corpuscular volume (MCV), mean corpuscular hemoglobin (MCH), mean corpuscular hemoglobin concentration (MCHC), and red cell distribution width (RDW).
- Platelet parameters: platelet count and immature platelet fraction (IPF).

- White blood cell parameters: total white blood cells (WBCs), neutrophils, lymphocytes, monocytes, eosinophils, basophils, immature granulocytes, nucleated red blood cells (NRBCs), reticulocytes, and immature reticulocyte fraction (IRF).

Additionally, to further assess potential nephrotoxicity or hepatotoxicity, several biochemical parameters were measured in serum, including alanine aminotransferase (ALT), aspartate aminotransferase (AST), gamma-glutamyl transferase (GGT), alkaline phosphatase (ALP), creatinine, urea, albumin, glucose, triglycerides, and bilirubin.

Statistical analysis

Statistical analyses were performed using the GraphPad Prism 9.2.0 software (GraphPad Software). Once the normality assessment with Shapiro-Wilk test was carried out, the statistical difference between two data sets was determined using the parametric paired or unpaired Student´s *t*-test or the non-parametric Mann-Whitney test. For comparison between more than two data sets, one-way analysis of variance (ANOVA) with Tukey´s *post hoc* test or Kruskal-Wallis with Dunn´s *post hoc* test was implemented for the analysis of normally and non-normally distributed data, respectively. Data are indicated as mean ± standard error of the mean (SEM), and differences of $p < 0.05$ were considered statistically significant.

Supplementary figures

CisPt

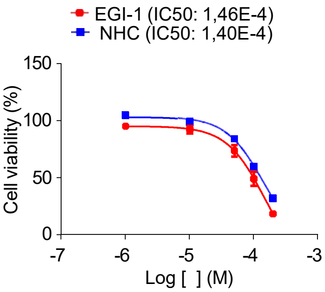

Aurkine-10

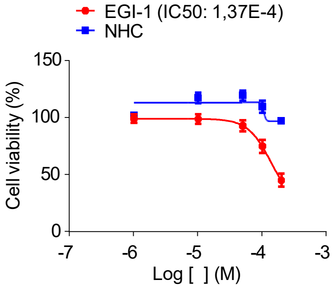

Aurkine-14

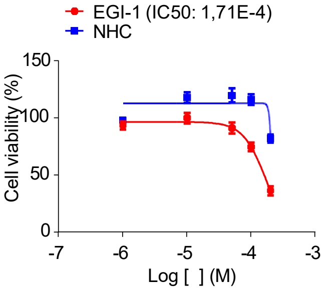

Aurkine-15

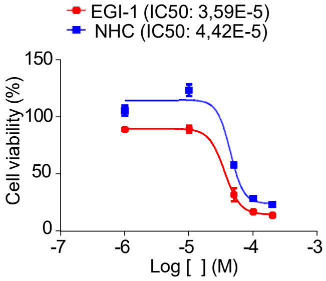

Aurkine-16

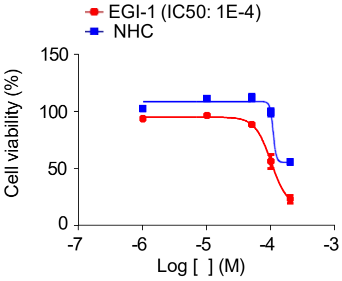

Aurkine-17

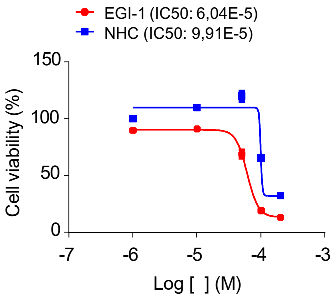

Aurkine-18

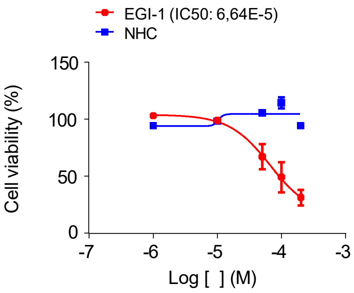

Fig. S1. Dose-response curves and IC50 values of Aurkines on eCCA (EGI- 1) cells and NHCs. Dose-response curves (0.1 µM, 1 µM, 5 µM, 10 µM, 20 µM) and IC50 values of the Aurkine compounds were determined for the eCCA cell line (EGI-1) and NHCs. Curve fitting and IC50 calculation were performed using the log(inhibitor) vs. normalized response with variable slope equation in GraphPad 9.2.0. Abbreviations: CisPt, cisplatin; NHCs, normal human cholangiocytes.

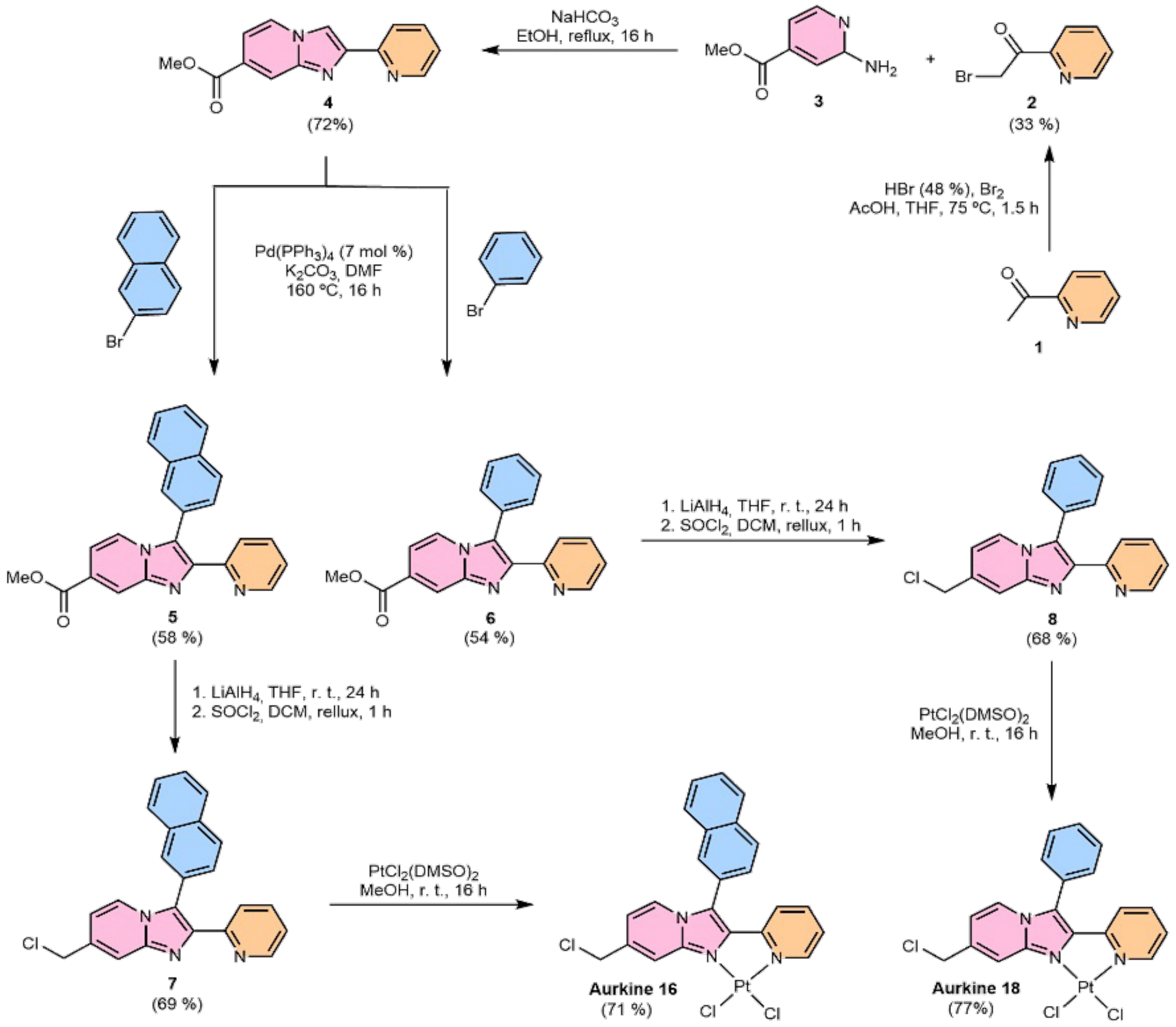

Fig. S2. Chemical synthesis of Aurkines 16 and 18. THF: tetrahydrofuran; DMSO: dimethylsulfoxide; AcOH: acetic acid; EtOH: ethanol.

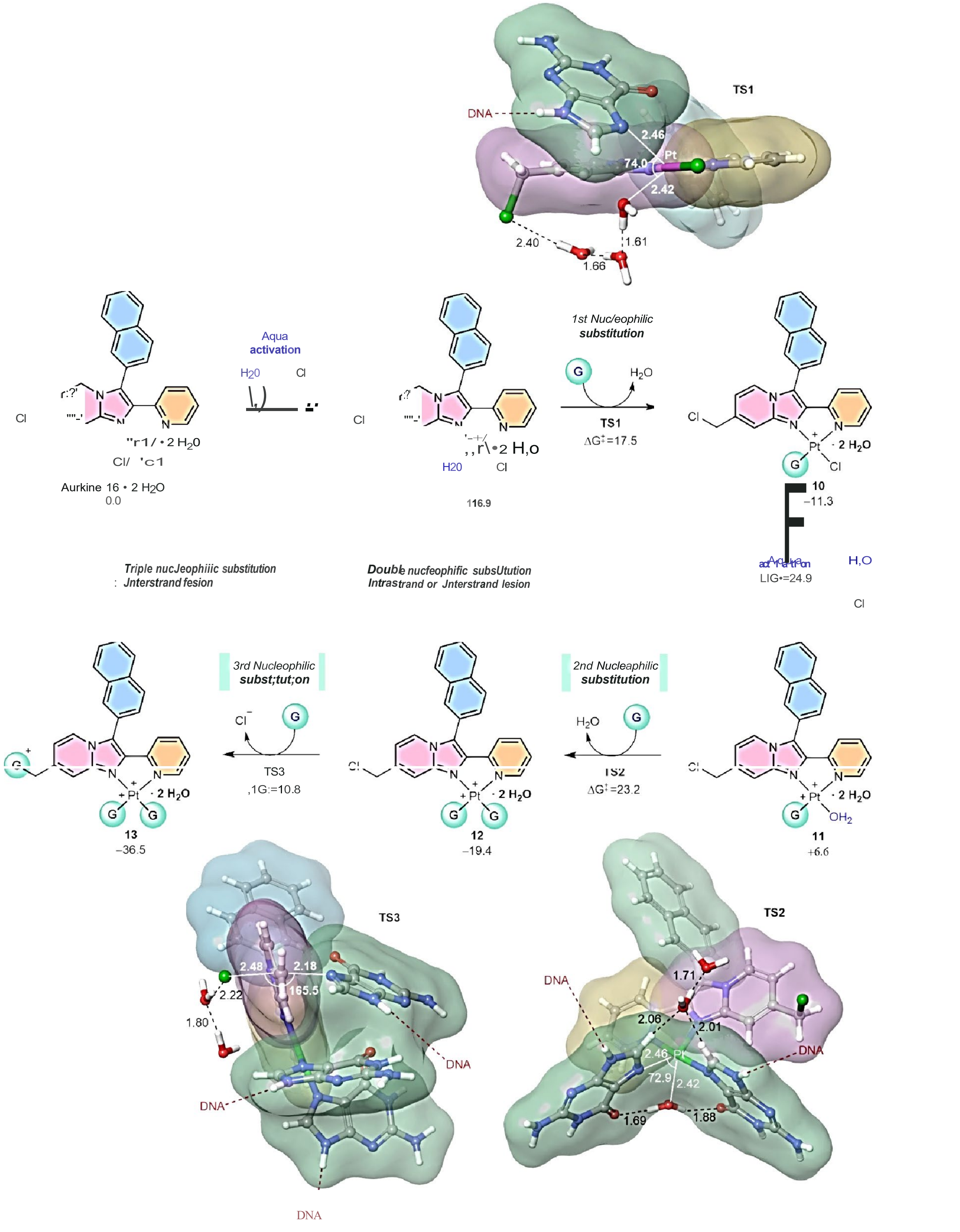
TS1
DNA
2.46
Pt
74.0
2.42
2.40
1.61
1.66
Aqua
activation
Cl
Aurkine 16 • 2 H2O
0.0
1st Nuc/eophilic
substitution
G
H2O
TS1
ΔG‡=17.5
116.9
10
−11.3
H,O
LlG•=24.9
Triple nucJeophiiic substitution
Jnterstrand fesion
Double nucfeophific subsUtution
Intrastrand or Jnterstrand lesion
3rd Nucleophilic
subst;tut;on
2nd Nucleaphilic
substitution
TS3
TS2
ΔG‡=23.2
2 H2O
OH2
13
−36.5
12
−19.4
11
+6.6
TS3
2.48
2.18
165.5
2.22
1.80
DNA
TS2
1.71
2.06
2.01
2.46
72.9
2.42
1.69
1.88

Fig. S3. DFT analysis of Aurkine 16 activation and electrophilicity with DNA. All calculations were performed at the B3LYP/D3-BJ(PCM=water)/6-31+G(d,p)&LANL2DZ(Pt) level of theory**.** The figure displays the formation of aqua intermediates and substitution adducts with up to three guanine molecules, with either none or two water molecules. The numbers beneath the intermediates indicate the relative Gibbs free energies, while those under the reaction arrows denote activation energies ($\Delta G^{\ddagger}$) in kcal/mol calculated at 298 K. Transition structures (TSs) illustrate to the nucleophilic substitution reactions by guanine units, with bond distances and angles in Å and degrees, respectively.

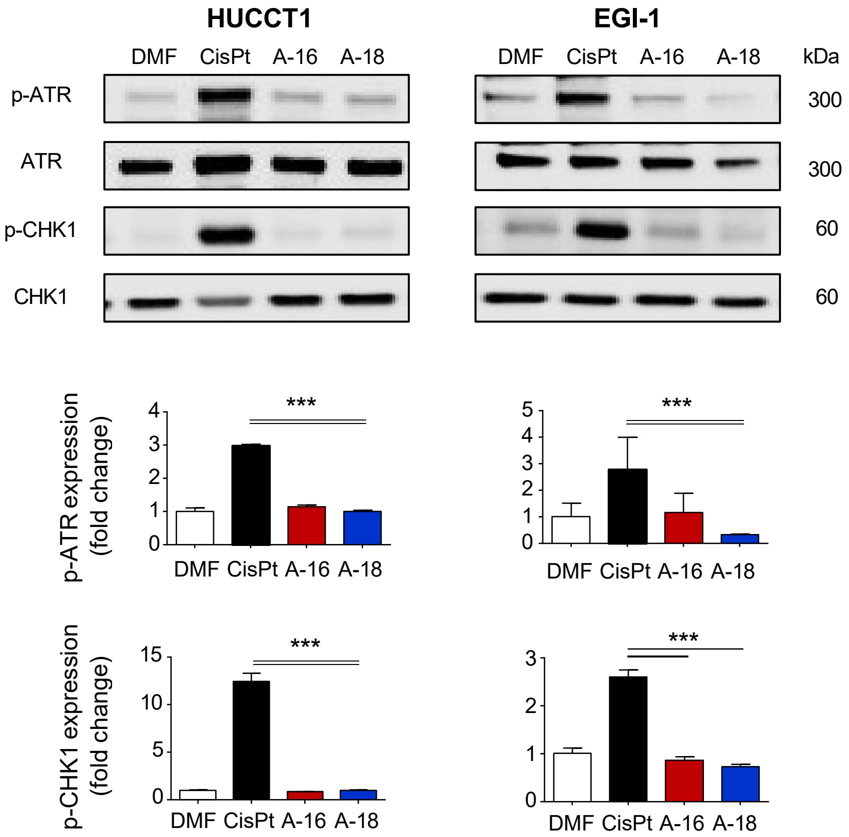

Fig. S4. Single-strand DNA repair pathway activation. Immunoblots and quantification of single-strand DNA repair proteins after 48-hour incubation with vehicle, CisPt, Aurkine 16 or 18 in CCA cell lines (HUCCT1 and EGI-1). β-actin served as a loading control. Student's t-test was used. Data are shown as mean ± SEM. p-values: * ($p \leq 0.05$), ** ($p \leq 0.01$), *** ($p \leq 0.001$). Abbreviations: ATR, ataxia telangiectasia and RAD3-related protein; CisPt, Cisplatin; CHK1, checkpoint kinase 1; CCA, cholangiocarcinoma; DMF, dimethylformamide.

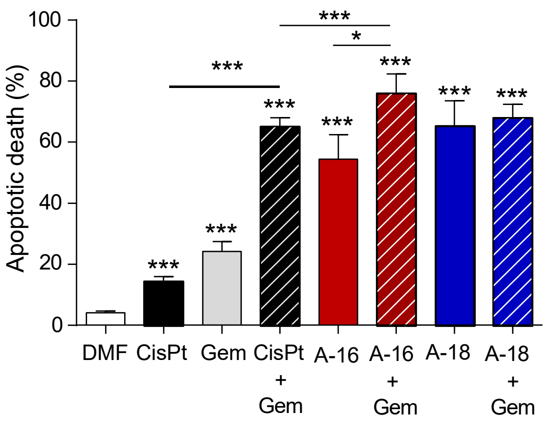

Fig. S5. Antitumor effect of Aurkines combined with Gemcitabine on EGI-1 CCA cells. Annexin V/TO-PRO™-3 dual staining of EGI-1 CCA cells after 48-hour incubation with CisPt, Aurkines 16 or 18 (10μM) alone or in combination with Gemcitabine (1 μM). Student's t-test was used. Data are shown as mean ± SEM. p-values: * ($p \le 0.05$), ** ($p \le 0.01$), *** ($p \le 0.001$). Abbreviations: CCA, cholangiocarcinoma; CisPt, Cisplatin; DMF, dimethylformamide; Gem, gemcitabine; NHC.

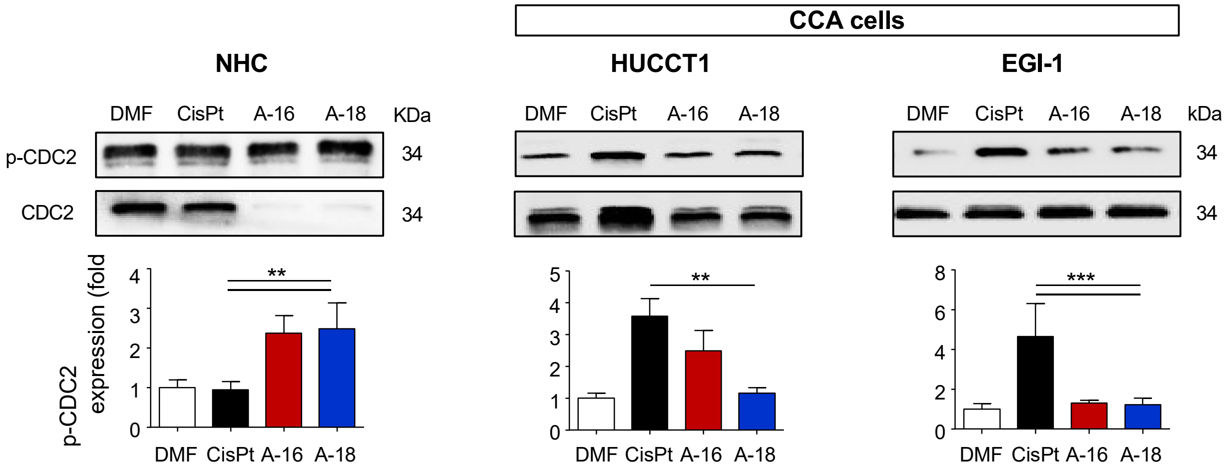

Fig. S6. p-CDC2 quantification. Immunoblot and quantification of p-CDC2 in NHC and CCA cell lines (HUCCT1 and EGI-1) following treatment with vehicle, CisPt, Aurkines 16 or 18 (10 µM), with β-actin as loading control. Student's t-test was used. Data are shown as mean ± SEM. p-values: * ($p \le 0.05$), ** ($p \le 0.01$), *** ($p \le 0.001$). Abbreviations: CisPt, Cisplatin; CCA, cholangiocarcinoma; DMF, dimethylformamide; NHC, normal human cholangiocytes.

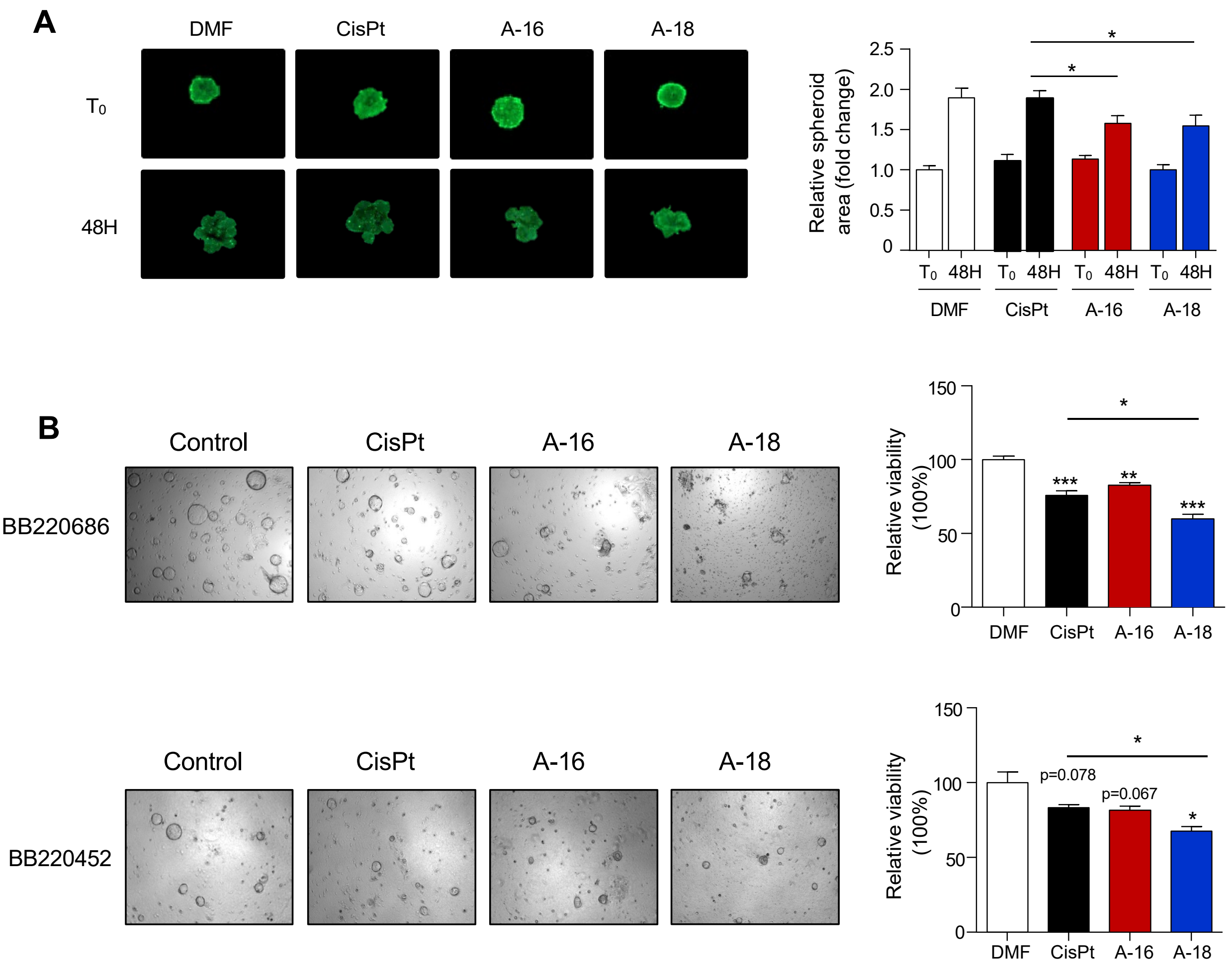

Fig. S7. Antitumoral effects of Aurkines on 3D models. (A) 3D spheroid formation of EGI-1 CCA cells after 48-hour incubation with vehicle, CisPt, Aurkines 16 or 18 (10 μM). (B) Cell viability of patient-derived organoids (PDOs) of two CCA patients after 72-hour treatment with CisPt, Aurkine 16 or Aurkine 18 (20 μM). Student's t-test was used. Data are shown as mean ± SEM. p-values: * ($p \leq 0.05$), ** ($p \leq 0.01$), *** ($p \leq 0.001$). Abbreviations: CisPt, Cisplatin; CCA, cholangiocarcinoma; DMF, dimethylformamide; PDO, patient-derived organoids.

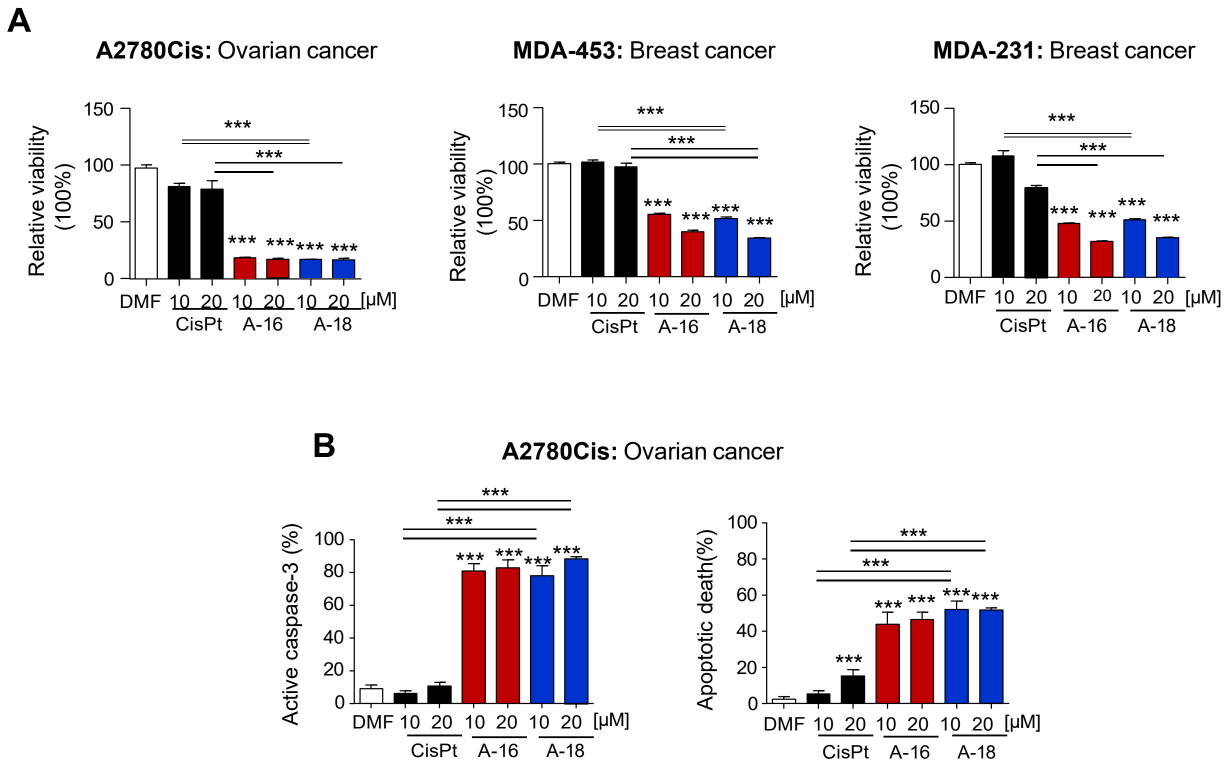

Fig. S8. Antitumor effect of Aurkines 16 and 18 on CisPt-resistant human cancer cell lines. (A) Cell viability of the ovarian cancer cell line (A2780Cis) and the breast cancer cell lines (MDA-453 and MDA-231) after 48-hour incubation with vehicle (DMF), CisPt, Aurkines 16 or 18 (10 μM and 20 μM). (B) % of cleaved caspase-3$^+$ cells and Annexin V/ TO-PRO^{TM}-3 dual staining of A2780Cis cells after 48-hour incubation with vehicle, CisPt, Aurkine 16 or 18 (10 μM and 20 μM). Student's t-test was used. Data are shown as mean ± SEM. p-values: * ($p \leq 0.05$), ** ($p \leq 0.01$), *** ($p \leq 0.001$). Abbreviations: CisPt, cisplatin; DMF, dimethylformamide.

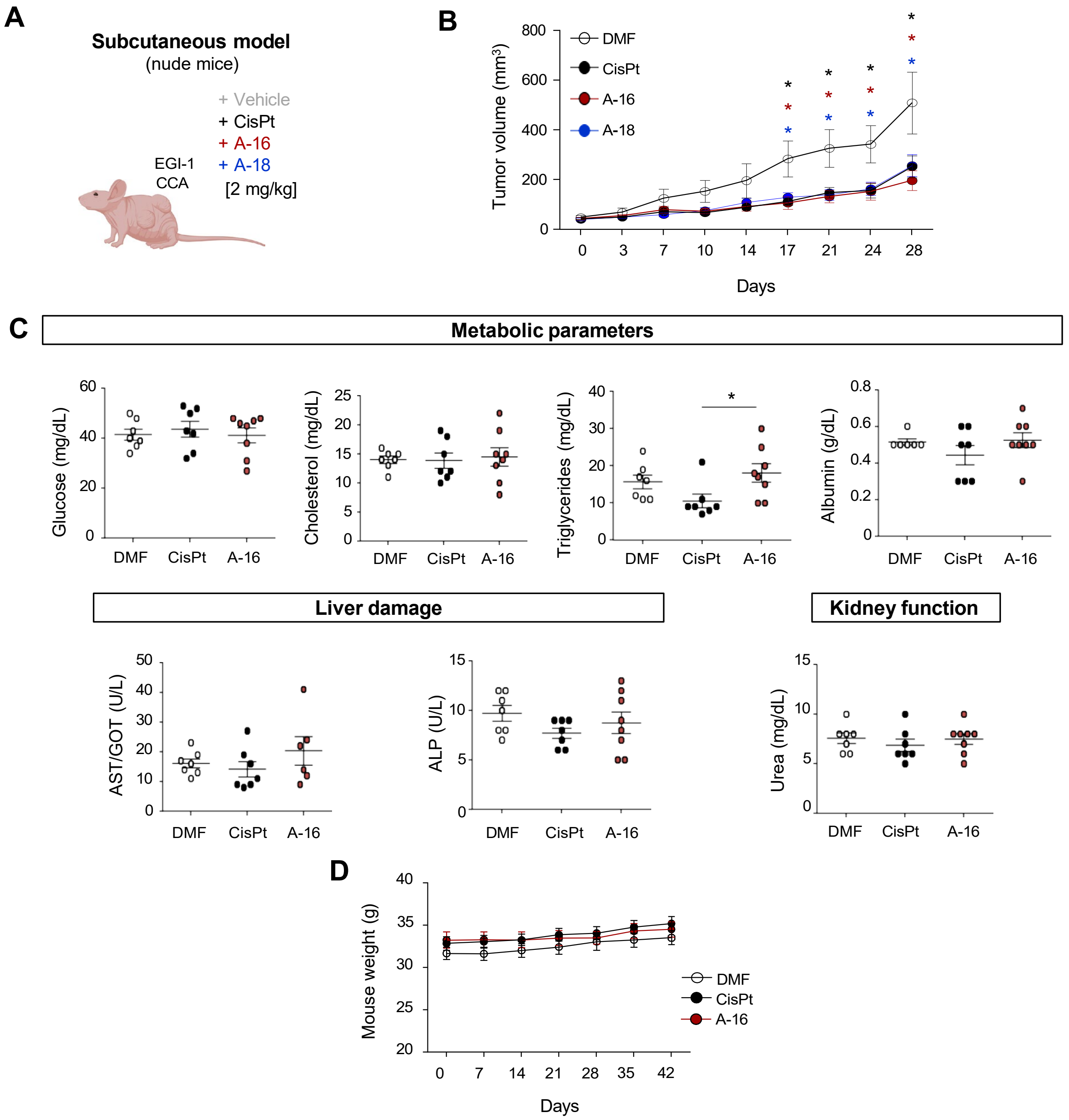

Fig. S9. *In vivo* antitumor activity of Aurkines 16 and 18 on CCA. (A) Schematic representation of the subcutaneous CCA model. (B) Tumor volume growth during treatment with CisPt, Aurkine 16 or Aurkine 18 (2 mg/kg). Group sizes: vehicle control (n = 8), CisPt (n = 8), Aurkine 16 (n = 8), and Aurkine 18 (n = 12). (C) Biochemical parameters in serum of mice treated with CisPt, Aurkine 16 or vehicle (DMF) (0.5 mg/kg). (D) Mouse weight over time. One-way ANOVA test or Student's t-tests were used. Data are shown as mean ± SEM. p-values: * ($p \leq 0.05$), ** ($p \leq 0.01$), *** ($p \leq 0.001$). Abbreviations: ALP, alkaline phosphatase; AST, aspartate aminotransferase; CisPt, cisplatin; DMF, dimethylformamide.

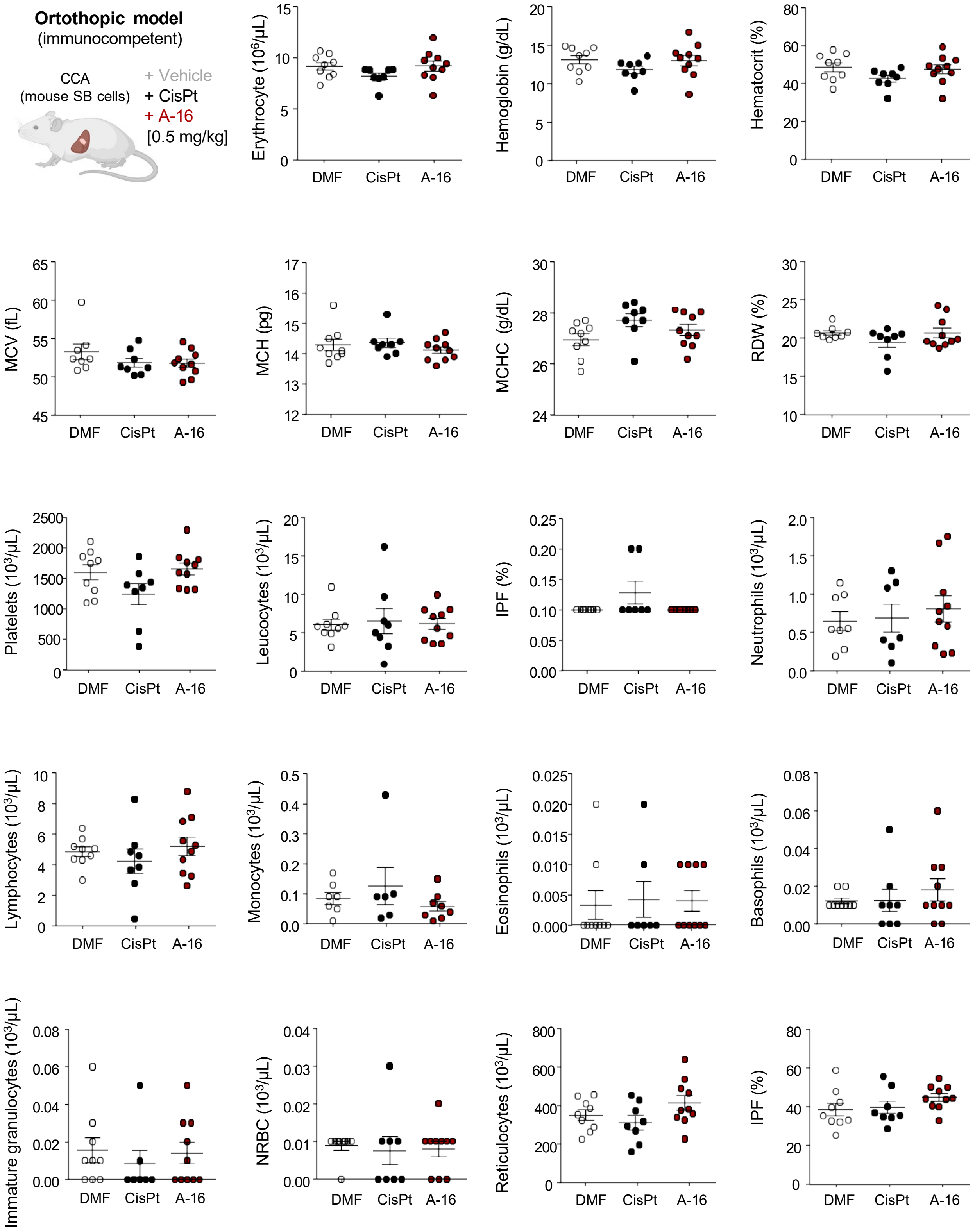
Ortothopic model
(immunocompetent)
CCA
(mouse SB cells)
+ Vehicle
+ CisPt
+ A-16
[0.5 mg/kg]
Erythrocyte (10^6/µL)
Hemoglobin (g/dL)
Hematocrit (%)
MCV (fL)
MCH (pg)
MCHC (g/dL)
RDW (%)
Platelets (10^3/µL)
Leucocytes (10^3/µL)
IPF (%)
Neutrophils (10^3/µL)
Lymphocytes (10^3/µL)
Monocytes (10^3/µL)
Eosinophils (10^3/µL)
Basophils (10^3/µL)
Immature granulocytes (10^3/µL)
NRBC (10^3/µL)
Reticulocytes (10^3/µL)
IPF (%)
DMF
CisPt
A-16

Fig. S10. Red and white blood cell parameters in the orthotopic CCA model after treatment with vehicle solution, CisPt or Aurkine 16. A comprehensive panel of hematological markers was evaluated in orthotopic xenografts after treatment with vehicle solution (n=9), CisPt (n=8) or Aurkine 16 (n=10) at a dose of 0.5 mg/kg, administered once per week for one month. Student's t-test were used. Data are shown as mean ± SEM. p-values: * ($p \leq 0.05$), ** ($p \leq 0.01$), *** ($p \leq 0.001$). Abbreviations: IPF: immature platelet fraction; IRF, immature reticulocyte fraction; MCH, mean corpuscular hemoglobin; MCHC, mean corpuscular hemoglobin concentration; MCV, mean corpuscular volume; NRBCs, nucleated red blood cells; RDW: red cell distribution width.

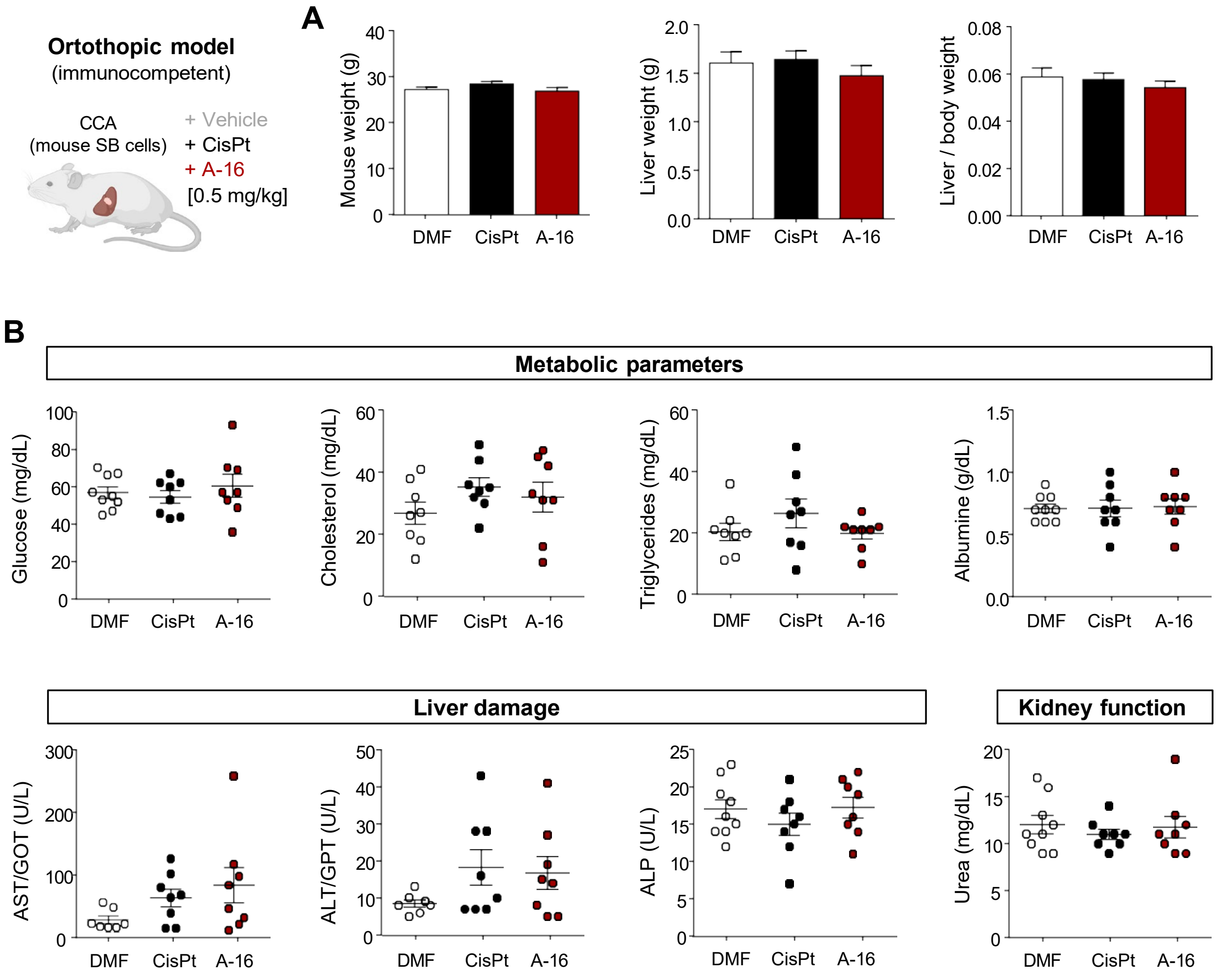

Fig. S11. Biochemical parameters in the orthotopic CCA model after treatment with vehicle solution, CisPt or Aurkine 16. (A) Mouse and liver weight at sacrifice.

(B) A comprehensive panel of biochemical markers was evaluated in orthotopic xenografts after treatment with vehicle solution (n=9), CisPt (n=8) or Aurkine 16 (n=10) at a dose of 0.5 mg/kg, administered once per week for one month. Student's t-test were used. Data are shown as mean ± SEM. p-values: * ($p \leq 0.05$), ** ($p \leq 0.01$), *** ($p \leq 0.001$). Abbreviations: ALP, alkaline phosphatase; ALT, alanine aminotransferase; AST, aspartate aminotransferase; GGT, gamma-glutamyl transferase.

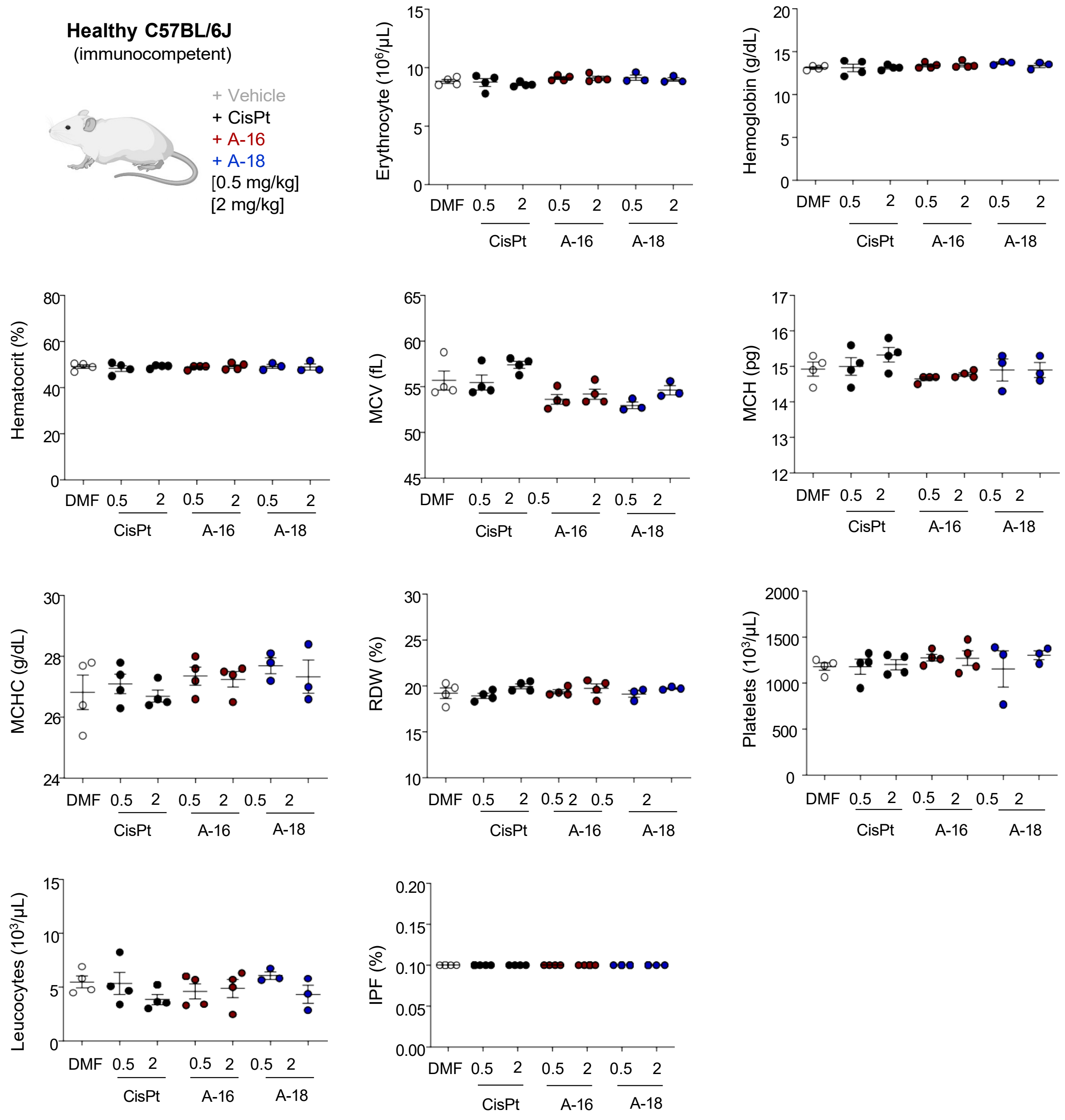

Fig. S12. Red blood cell parameters in healthy mice after treatment with CisPt, Aurkines or vehicle. Healthy C57BL/6J mice were treated once per week with either vehicle (DMF) (n=4), CisPt (n=4), Aurkine 16 (n=4), or Aurkine 18 (n=3) at doses of 0.5 mg/kg and 2 mg/kg for one month. A comprehensive panel of hematological markers was evaluated. Student's t-test were used. Data are shown as mean ± SEM. p-values: * ($p \leq 0.05$), ** ($p \leq 0.01$), *** ($p \leq 0.001$). Abbreviations: IPF: immature platelet fraction; MCH, mean corpuscular hemoglobin; MCHC, mean corpuscular hemoglobin concentration; MCV, mean corpuscular volume.

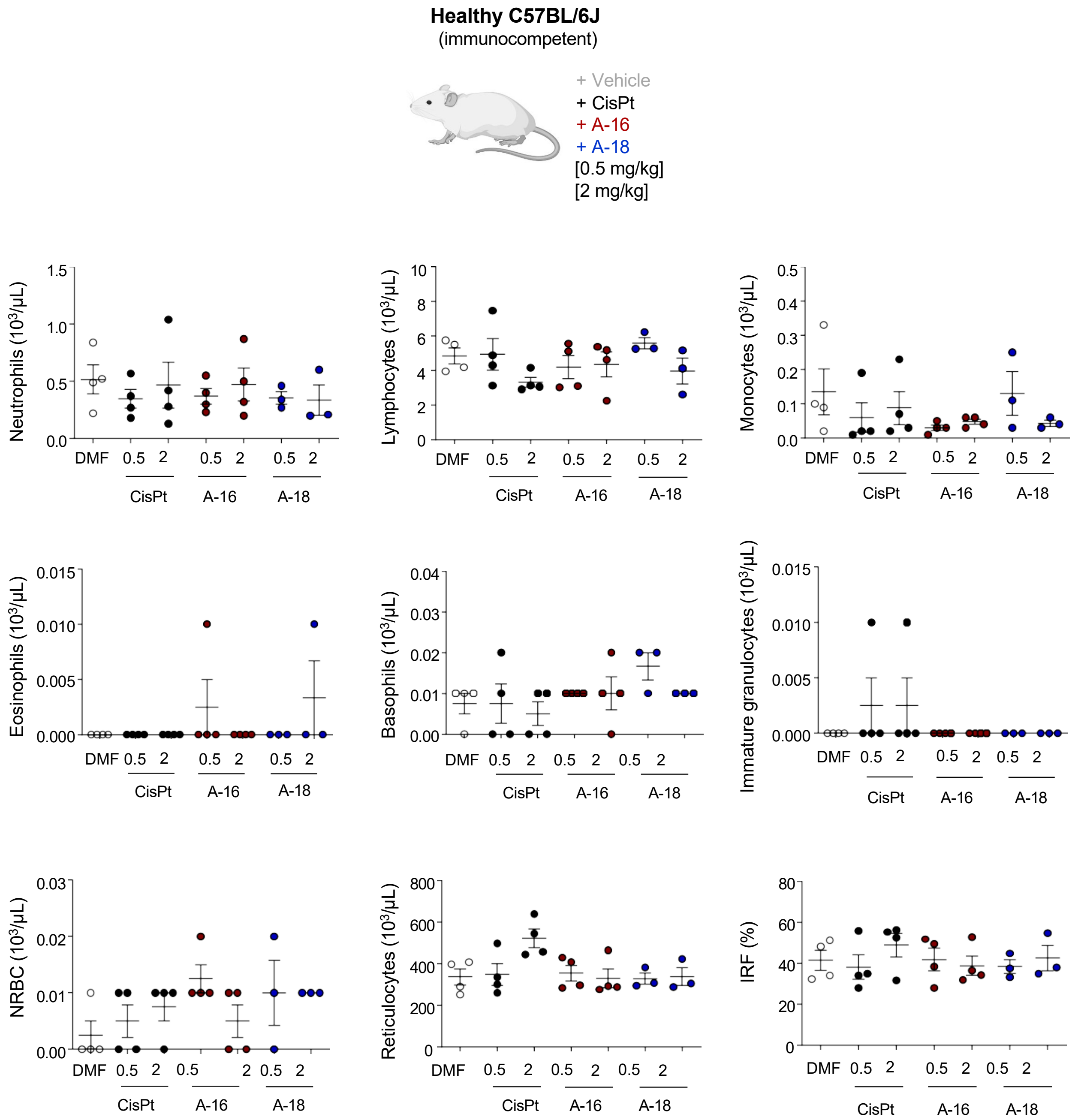

Fig. S13. White blood cell parameters in healthy mice after treatment with CisPt, Aurkines or vehicle. Healthy C57BL/6J mice were treated once per week with either vehicle (DMF) (n=4), CisPt (n=4), Aurkine 16 (n=4), or Aurkine 18 (n=3) at doses of 0.5 mg/kg and 2 mg/kg for one month. A comprehensive panel of hematological markers was evaluated. Student's t-test were used. Data are shown as mean ± SEM. p-values: * ($p \leq 0.05$), ** ($p \leq 0.01$), *** ($p \leq 0.001$). Abbreviations: Abbreviations: IRF, immature reticulocyte fraction; NRBCs, nucleated red blood cells.

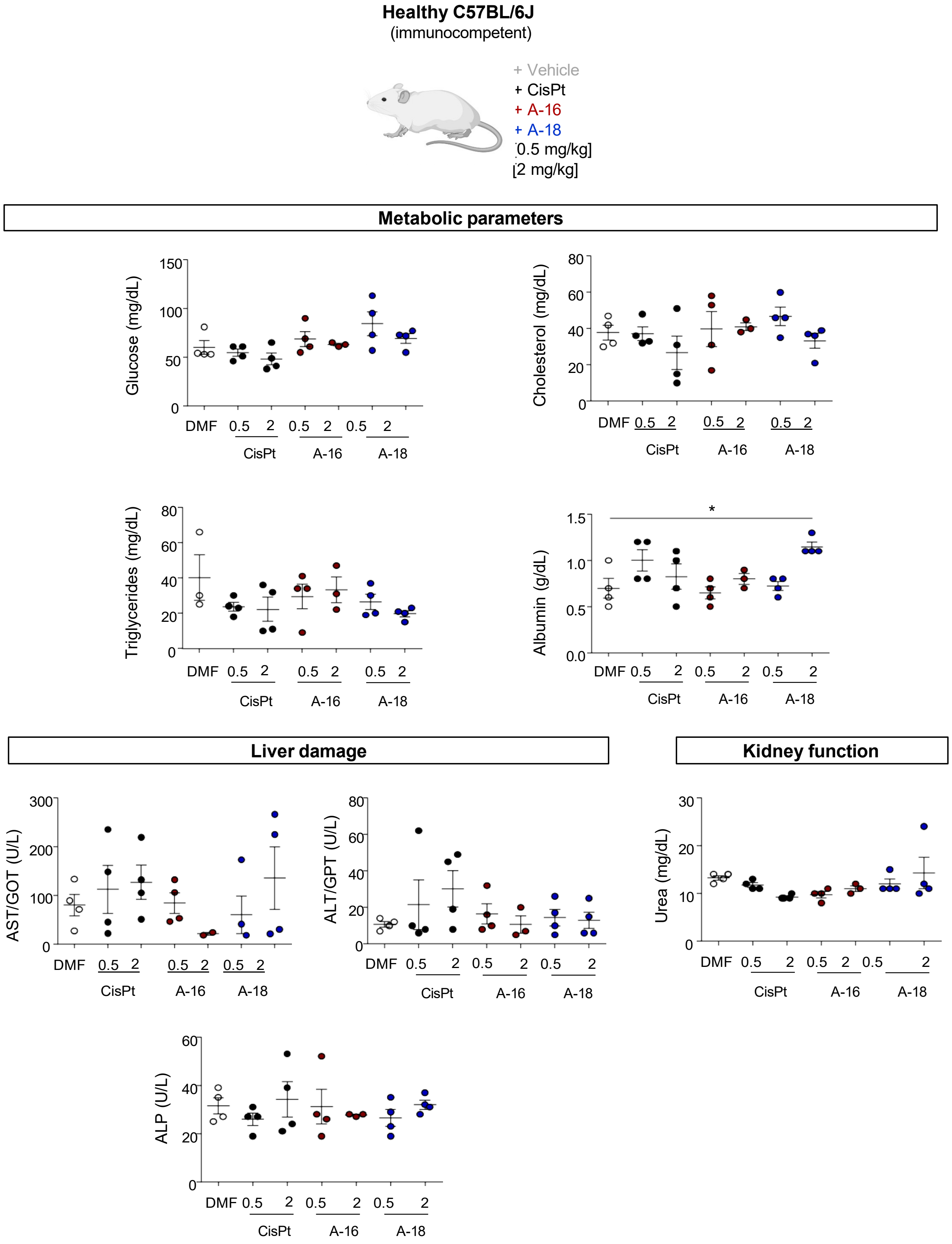

Healthy C57BL/6J
(immunocompetent)
+ Vehicle
+ CisPt
+ A-16
+ A-18
[0.5 mg/kg]
[2 mg/kg]
Metabolic parameters
Glucose (mg/dL)
Cholesterol (mg/dL)
Triglycerides (mg/dL)
Albumin (g/dL)
*
DMF
0.5
2
CisPt
A-16
A-18
Liver damage
AST/GOT (U/L)
ALT/GPT (U/L)
ALP (U/L)
Kidney function
Urea (mg/dL)

Fig. S14. Biochemical parameters in healthy mice after treatment with CisPt, Aurkines, or vehicle. Healthy C57BL/6J mice were treated once per week with either vehicle (DMF) (n=4), CisPt (n=4), Aurkine 16 (n=4), or Aurkine 18 (n=4) at doses of 0.5 mg/kg and 2 mg/kg for one month. A comprehensive panel of biochemical parameters was evaluated in serum. Student's t-test were used. Data are shown as mean ± SEM. p-values: * ($p \leq 0.05$), ** ($p \leq 0.01$), *** ($p \leq 0.001$). Abbreviations: ALP, alkaline phosphatase; ALT, alanine aminotransferase; AST, aspartate aminotransferase; GGT, gamma-glutamyl transferase.

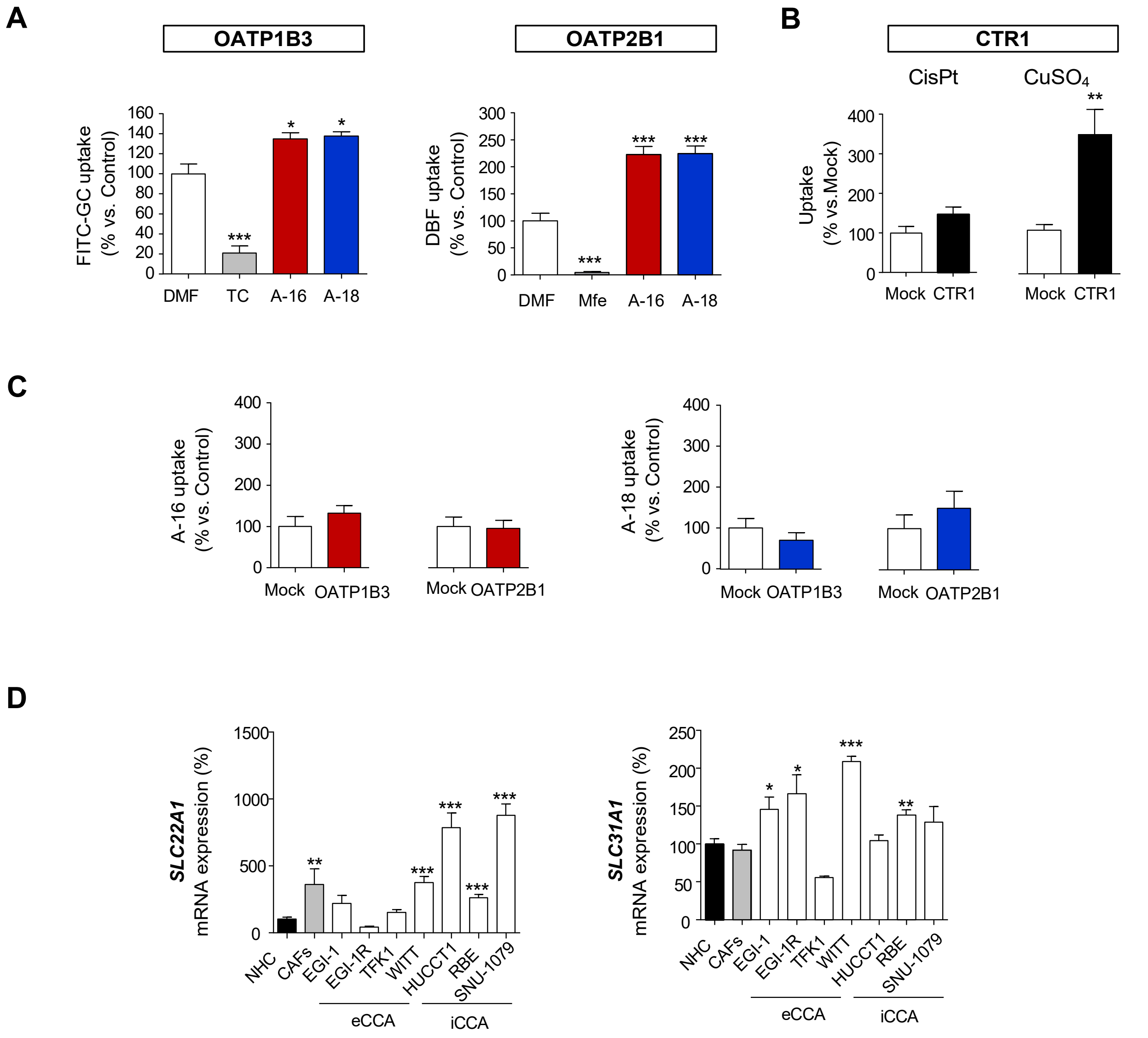

Fig. S15. Analysis of transporters involved in Aurkines 16 and 18 uptake by cancer cells. (A) Uptake of specific fluorescent substrates by cells with or without experimental overexpression of upstream transporters measured by flow cytometry. (B) Atomic absorption spectroscopy (AAS) was employed to determine the accumulation of Pt and Cu after incubating the cells with CisPt or CuSO4. (C) Intracellular accumulation of Aurkine 16 and 18 in cells with or without overexpression of different transporters measured by HPLC-MS/MS. (D) Relative mRNA expression (qPCR) of *SLC22A1* and *SLC31A1* in NHC, CAFs, eCCA (i.e., EGI-1, EGI-1R, TFK1, WITT) and iCCA (i.e., HUCCT1, RBE and SNU-1079) cells. Student's t-test was used. Data are shown as mean ± SEM. p-values: * (p ≤ 0.05), ** (p ≤ 0.01), *** (p ≤ 0.001). Abbreviations: CTR1, copper transport 1; Mfe, mifepristone; NHC, normal human cholangiocytes; OATP, organic-anion-transporting polypeptide; OCT, organic cation transporter; TC, taurocholate.

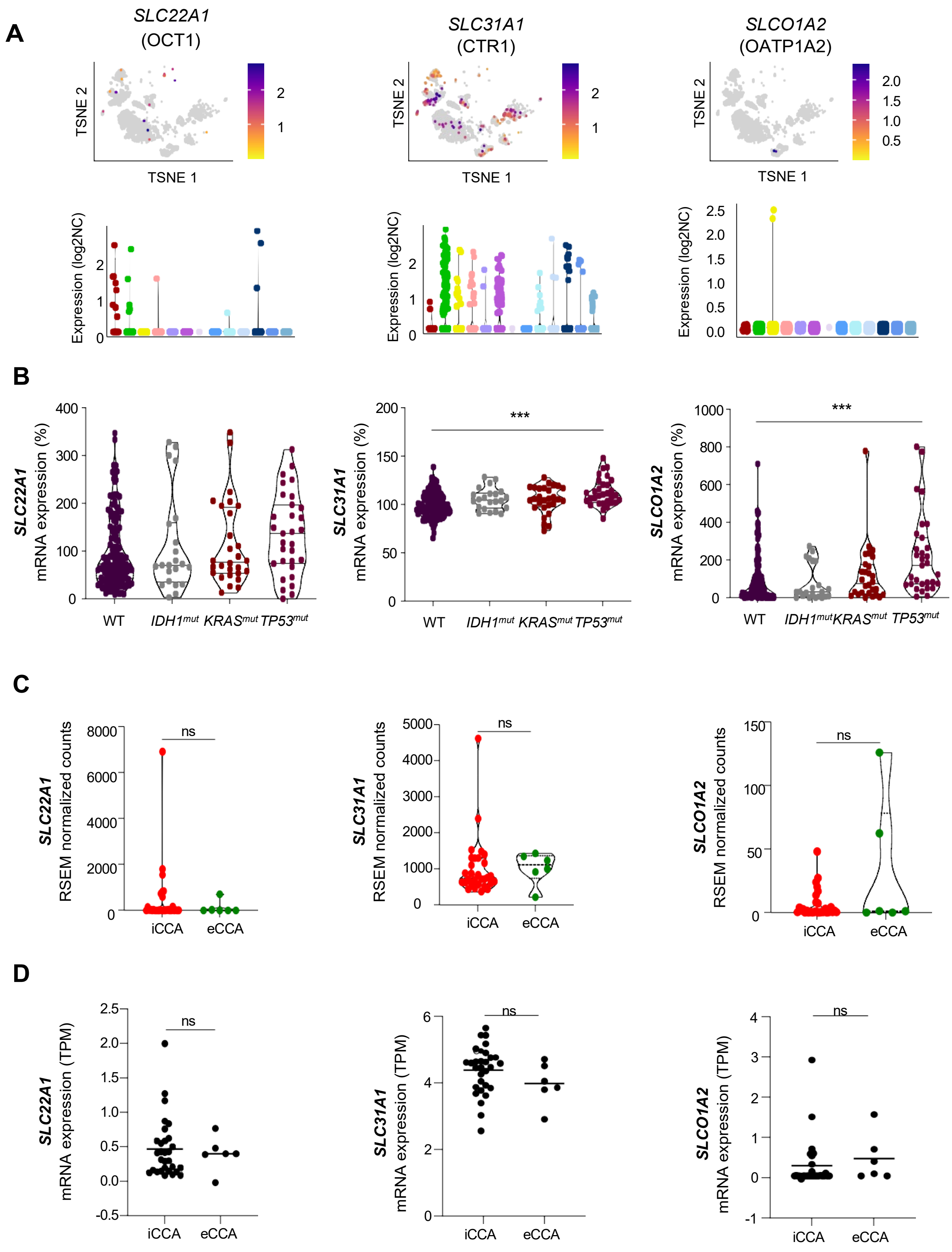
A
SLC22A1
(OCT1)
SLC31A1
(CTR1)
SLCO1A2
(OATP1A2)
TSNE 2
TSNE 1
Expression (log2NC)
B
SLC22A1
mRNA expression (%)
SLC31A1
SLCO1A2
WT
IDH1mut
KRASmut
TP53mut
***
C
RSEM normalized counts
ns
iCCA
eCCA
D
mRNA expression (TPM)

Fig. S16. Expression levels of *SLC22A1, SLC31A1 and SLCO1A2*. (A) Expression levels of *SLC22A1, SLC31A1* and *SLCO1A2* across all the cell populations of human CCA tumors detected by scRNA-seq in the GSE151530 dataset, which includes samples from 12 CCA patients. (B) Expression of *SLC22A1, SLC31A1* and *SLCO1A2* in CCA tissues, stratified by mutational status (*IDH1, KRAS, TP53* or wild type type for these genetic alterations). (C) mRNA expression of *SLC22A1, SLC31A1* and *SLCO1A2* in CCA tissues, stratified by anatomical origin (iCCA vs eCCA). (D) mRNA expression of *SLC22A1, SLC31A1* and *SLCO1A2* in CCA cell lines, stratified by anatomical origin (iCCA vs eCCA). One-way ANOVA test or Student's t-test were used. Data are shown as mean ± SEM. p-values: * ($p \leq 0.05$), ** ($p \leq 0.01$), *** ($p \leq 0.001$). Abbreviations: eCCA, extrahepatic cholangiocarcinoma; iCCA, intrahepatic cholangiocarcinoma; WT, wild type.

Supplementary tables

Table S1. Human primers sequences employed for qPCR (all from Sigma-Aldrich).

Gene	Sequence
SLC22A1 (OCT1)	Forward 5'-GTCGCTTTGCCAGAGACCAT-3'<br>Reverse 5'-CTTCATCCCTCCAACATGACA-3'
SLC22A3 (OCT3)	Forward 5'-ATCGTCAGCGAGTTTGACCTT-3'<br>Reverse 5'-ACCTGTCTGCTGCATAGCCTA-3'
SLC31A1 (CTR1)	Forward 5’-TGCGTAAGTCACAAGTCAGC-3’<br>Reverse 5’-CTGCTACTGCAATGCAGAGG-3’
SLC51A (OST-α)	Forward 5’- TTCCAGGTTCTCCTCATCCTGAC -3’<br>Reverse 5’- CAATTCATCACTTGAGACCTGGTTT-3’
GADPH	Forward 5’-CCAAGGTCATCCATGACAAC-3’<br>Reverse 5’-TGTCATACCAGGAAATGAGC-3’

Abbreviations: CTR1, copper transporter 1; GAPDH, glyceraldehyde-3-phosphate dehydrogenase; OCT1, organic cation transporter 1; OCT3, organic cation transporter 3; OST, organic solute transporter.

Table S2. Antibodies employed for WB and IHC assays.

Antibody	Clone	Company	Reference	Application
Mouse monoclonal anti-ATR	C-1	Santa Cruz Biotechnology	sc-515173	WB
Rabbit monoclonal anti-p-ATR	T1989	Abcam	ab223258	WB
Mouse monoclonal anti-CHK1	2G1D5	Cell Signaling	2360S	WB
Rabbit monoclonal anti-p-CHK1 (Ser345)	133D3	Cell Signaling	2348T	WB
Mouse monoclonal anti-CDC2	n/a	Santa Cruz Biotechnology	sc-54	WB
Mouse monoclonal anti-p-CDC2	pY15.44	Santa Cruz Biotechnology	sc-136014	WB
Mouse monoclonal anti β-actin	AC-74	Sigma-Aldrich	A5316	WB
Rabbit monoclonal anti-CK19	EP1580Y	Abcam	ab52625	IHC
Rabbit monoclonal anti-CD4	EPR19514	Abcam	ab183685	IHC
Rabbit monoclonal anti-CD8	D4W2Z	Cell Signaling	98941	IHC
Rabbit monoclonal anti-KI67	SP6	Abcam	ab16667	IHC
Rabbit polyclonal anti-PCNA	n/a	Abcam	ab18197	IHC
Rabbit monoclonal anti-Cleaved Caspase-3 (Asp175)	5A1E	Cell Signaling	9664	IHC
Rabbit polyclonal anti-phospho-Histone H3 (Ser28)	n/a	Cell Signaling	9713	IHC

n/a: not applicable. Abbreviations: CK19, cytokeratin 19; IHC, immunohistochemistry; p-ATR, phosphorylated ataxia telangiectasia and RAD3-related protein; p-CHK1, phosphorylated chekpoint kinase 1; PCNA, proliferating cell nuclear antigen; WB, western blot.

Table S3. Inhibitor and known fluorescent substrate of each transporter.

Transporter	Fluorescent substrate	Inhibitor
OATP1A2	Rhodamine-123 1 μM	Rifampicin 100 μM
OATP1B3	FITC-GC 1 μM	Taurocholic acid 100 μM
OATP2B1	Dibromofluorescein 1 μM	Mifepristone 20 μM
OCT1	Dihydroethidium 1 μM	Quinine 50 μM
OCT3	Dihydroethidium 1 μM	Quinine 10 μM

Abbreviations: DBF, dibenzylfluorescein; DHE, dihydroethidium; FITC-GC, fluorescent bile acid derivative cholylglycylamido-fluorescein; OATP, organic anion transporting polypeptide; OCT, organic cation transporter

Supplementary references

Author names in bold designate shared co-first authorships.

[1] Merino-Azpitarte M, Lozano E, Perugorria MJ, et al. SOX17 regulates cholangiocyte differentiation and acts as a tumor suppressor in cholangiocarcinoma. J Hepatol 2017;67:72–83.

[2] Erice O, Labiano I, Arbelaiz A, et al. Differential effects of FXR or TGR5 activation in cholangiocarcinoma progression. Biochim Biophys Acta Mol Basis Dis 2018;1864:1335–44.

[3] Olaizola P, Lee-Law PY, Fernandez-Barrena MG, et al. Targeting NAE1-mediated protein hyper-NEDDylation halts cholangiocarcinogenesis and impacts on tumor-stroma crosstalk in experimental models. J Hepatol 2022;77:177–90.

[4] Vlachogiannis G, Hedayat S, Vatsiou A, et al. Patient-derived organoids model treatment response of metastatic gastrointestinal cancers. Science 2018;359:920–6.

[5] **Boj SF, Hwang C II, Baker LA,** et al. Organoid models of human and mouse ductal pancreatic cancer. Cell 2015;160:324–38.

[6] Ahn KS, O'Brien D, Kang YN, et al. Prognostic subclass of intrahepatic cholangiocarcinoma by integrative molecular-clinical analysis and potential targeted approach. Hepatol Int 2019;13:490–500.

[7] **Chaisaingmongkol J, Budhu A,** Dang H, et al. Common Molecular Subtypes Among Asian Hepatocellular Carcinoma and Cholangiocarcinoma. Cancer Cell 2017;32:57-70.e3.

[8] Andersen JB, Spee B, Blechacz BR, et al. Genomic and genetic characterization of cholangiocarcinoma identifies therapeutic targets for tyrosine kinase inhibitors. Gastroenterology 2012;142.

[9] Farshidfar F, Zheng S, Gingras MC, et al. Integrative Genomic Analysis of Cholangiocarcinoma Identifies Distinct IDH-Mutant Molecular Profiles. Cell Rep 2017;18:2780–94.

[10] **Job S, Rapoud D**, Dos Santos A, et al. Identification of Four Immune Subtypes Characterized by Distinct Composition and Functions of Tumor Microenvironment in Intrahepatic Cholangiocarcinoma. Hepatology 2020;72:965–81.

[11] Dong L, Lu D, Chen R, et al. Proteogenomic characterization identifies clinically relevant subgroups of intrahepatic cholangiocarcinoma. Cancer Cell 2022;40:70-87.e15.

[12] **Tsherniak A, Vazquez F**, Montgomery PG, et al. Defining a Cancer Dependency Map. Cell 2017;170:564-576.e16.

[13] **Ma L, Wang L, Khatib SA,** et al. Single-cell atlas of tumor cell evolution in response to therapy in hepatocellular carcinoma and intrahepatic cholangiocarcinoma. J Hepatol 2021;75:1397–408.

[14] Schneider CA, Rasband WS, Eliceiri KW. NIH Image to ImageJ: 25 years of image analysis. Nat Methods 2012;9:671–5.

[15] Demichev V, Messner CB, Vernardis SI, et al. DIA-NN: neural networks and interference correction enable deep proteome coverage in high throughput. Nature Methods 2019 17:1 2019;17:41–4.

[16] **Yu F, Haynes SE,** Teo GC, et al. Fast Quantitative Analysis of timsTOF PASEF Data with MSFragger and IonQuant. Mol Cell Proteomics 2020;19:1575–85.

[17] Krämer A, Green J, Pollard J, et al. Causal analysis approaches in Ingenuity Pathway Analysis. Bioinformatics 2014;30:523–30.

[18] Perez-Riverol Y, Bandla C, Kundu DJ, et al. The PRIDE database at 20 years: 2025 update. Nucleic Acids Res 2025;53:D543–53.

[19] Rizvi S, Fischbach SR, Bronk SF, et al. YAP-associated chromosomal instability and cholangiocarcinoma in mice. Oncotarget 2017;9:5892–905.
